# Supplementary material for: Cross-sectional study of association between psychosocial stressors with chronic kidney disease among migrant and non-migrant Ghanaians living in Europe and Ghana: the RODAM study
Source: BMJ Open. 2019 Aug 1;9(8):e027931. doi: 10.1136/bmjopen-2018-027931 (PMC6688695; doi:10.1136/bmjopen-2018-027931)
Supplement: Supplementary data [file bmjopen-2018-027931supp001.pdf]

Supplementary Tables

S1: Association of psychosocial stressors (PS) indicators (negative life events, discrimination, stress at work/home and depression) with albuminuria, reduced eGFR and CKD risk for Ghanaians living in Ghana and those living in Europe

	Albuminuria (ACR ≥ 3 mg/mmol)		eGFR < 60 mL/min/1.73 m2		High to very high CKD risk (KDIGO, 2012)	
		OR (95% CI)		OR (95% CI)		OR (95% CI)
	n (%)	Model 3	n (%)	Model 3	n cases (%)	Model 3
**Negative events**						
**Europe**						
No	1128 (8.2)	1.00 (Reference)	1106 (2.6)	1.00 (Reference)	1090 (8.5)	1.00 (Reference)
Yes	1615 (8.4)	0.95 (0.70-1.30)	1587 (2.5)	0.77 (0.44-1.37)	1557 (8.6)	0.92 (0.67-1.26)
**Ghana**						
No	732 (10.4)	1.00 (Reference)	736 (4.5)	1.00 (Reference)	732 (10.9)	1.00 (Reference)
Yes	1595 (9.2)	0.79 (0.58-1.07)	1601 (3.4)	0.69 (0.43-1.09)	1590 (9.9)	0.79 (0.58-1.06)
**Discrimination**						
**Europe**						
No	1899 (8.5)	1.00 (Reference)	1867 (2.6)	1.00 (Reference)	1832 (8.9)	1.00 (Reference)
Yes	810 (7.4)	0.87 (0.61-1.21)	791 (2.2)	0.87 (0.45-1.65)	782 (7.3)	0.79 (0.55-1.12)
**Ghana**						
No	2034 (10.0)	1.00 (Reference)	2047 (3.9)	1.00 (Reference)	2031 (10.6)	1.00 (Reference)
Yes	104 (7.7)	0.92 (0.43-1.98)	104 (1.9)	0.65 (0.15-2.79)	104 (6.7)	0.74 (0.33-1.66)
**Stress at home/work**						
**Europe**						
Never	1330 (8.2)	1.00 (Reference)	1305 (3.4)	1.00 (Reference)	1282 (8.5)	1.00 (Reference)
Some stress	1002 (7.9)	1.01 (0.72-1.41)	984 (1.4)	0.51 (0.25-1.04)	968 (8.1)	1.01 (0.72-1.42)
Several/Permanent stresses	397 (9.1)	1.00 (0.64-1.59)	390 (2.3)	0.91 (0.40-2.03)	383 (9.7)	1.15 (0.74-1.79)
**Ghana**						

Never	682 (10.3)	1.00 (Reference)	688 (3.3)	1.00 (Reference)	682 (9.9)	1.00 (Reference)
Some stress	1279 (9.5)	0.78 (0.56-1.09)	1279 (3.9)	1.14 (0.67-1.93)	1274 (10.3)	0.93 (0.67-1.29)
Several/Permanent stresses	365 (8.5)	0.70 (0.43-1.13)	369 (4.1)	1.32 (0.65-1.68)	365 (10.4)	0.97 (0.62-1.52)
**Depressive symptoms**						
**Europe**						
No	2505 (8.5)	1.00 (Reference)	2457 (2.7)	1.00 (Reference)	2416 (8.7)	1.00 (Reference)
Yes	206 (6.3)	0.65 (0.33-1.28)	202 (1.5)	0.94 (0.28-3.14)	199 (7.1)	0.78 (0.41-1.47)
**Ghana**						
No	2212 (9.9)	1.00 (Reference)	2222 (3.8)	1.00 (Reference)	2207 (10.4)	1.00 (Reference)
Yes	114 (5.3)	0.41 (0.16-1.04)	114 (2.6)	0.58 (0.17-1.95)	114 (7.9)	0.62 (0.28-1.31)

Model 3, adjusted for age, sex educational level, hypertension, diabetes, hypercholesterolemia, BMI, physical activity and smoking; Abbreviations: CI, confidence interval; ACR, albumin creatinine ration; eGFR, estimated glomerular filtration rate; CKD, chronic kidney disease; OR, odds ratio, n= total number of Ghanaians living in Ghana and Europe among the various levels of PS constructs; %, proportion of individuals with CKD among the various levels of PS constructs in Ghana and Europe.

S2: Association of psychosocial stressors (PS) indicators (negative life events, discrimination, stress at work/home and depression) with albuminuria, reduced eGFR and CKD risk for Ghanaians living in Ghana and those living in Europe stratified by obesity status

	Albuminuria (ACR ≥ 3 mg/mmol)			eGFR < 60 mL/min/1.73 m2			High to very high CKD risk (KDIGO, 2012)		
		OR (95% CI)			OR (95% CI)			OR (95% CI)	
	n (%)	Model 1	Model 2	n (%)	Model 1	Model 2	n cases (%)	Model 1	Model 2
**Negative events**									
**Europe/Not obese**									
No	736 (7.4)	1.00 (Reference)	1.00 (Reference)	729 (2.5)	1.00 (Reference)	1.00 (Reference)	719 (8.5)	1.00 (Reference)	1.00 (Reference)
Yes	1003 (7.7)	0.98 (0.67-1.40)	0.99 (0.69-1.43)	992 (2.0)	0.77 (0.40-1.48)	0.71 (0.36-1.42)	971 (7.9)	0.91 (0.64-1.29)	0.89 (0.62-1.29)
**Europe/Obese**									
No	390 (9.2)	1.00 (Reference)	1.00 (Reference)	375 (2.9)	1.00 (Reference)	1.00 (Reference)	369 (8.7)	1.00 (Reference)	1.00 (Reference)
Yes	607 (9.6)	1.05 (0.67-1.63)	1.14 (0.72-1.82)	590 (3.2)	0.97 (0.44-2.11)	1.01 (0.44-2.26)	581 (9.6)	1.15 (0.73-1.82)	1.15 (0.71-1.85)
**Ghana/Not obese**									
No	588 (9.5)	1.00 (Reference)	1.00 (Reference)	592 (4.7)	1.00 (Reference)	1.00 (Reference)	588 (10.0)	1.00 (Reference)	1.00 (Reference)
Yes	1324 (8.5)	0.86 (0.61-1.21)	0.83 (0.59-1.17)	1331 (3.4)	0.64 (0.39-1.05)	0.65 (0.39-1.83)	1321 (9.0)	0.86 (0.61-1.20)	0.84 (0.60-1.18)
**Ghana/Obese**									
No	144 (13.9)	1.00 (Reference)	1.00 (Reference)	144 (3.5)	1.00 (Reference)	1.00 (Reference)	144 (14.6)	1.00 (Reference)	1.00 (Reference)
Yes	268 (13.1)	0.91 (0.50-1.64)	0.91 (0.49-1.65)	267 (3.8)	1.04 (0.34-3.16)	1.09 (0.35-3.37)	266 (14.3)	0.94 (0.52-1.68)	0.94 (0.52-1.67)
**Discrimination**									
**Europe/Not obese**									
No	1186 (8.4)	1.00 (Reference)	1.00 (Reference)	1172 (2.4)	1.00 (Reference)	1.00 (Reference)	1150 (9.4)	1.00 (Reference)	1.00 (Reference)
Yes	538 (5.9)	0.69 (0.46-1.05)	0.71 (0.46-1.11)	532 (1.5)	0.62 (0.28-1.37)	0.57 (0.24-1.34)	525 (5.9)	**0.63 (0.42-0.96)**	**0.63 (0.41-0.97)**
**Europe/Obese**									
No	709 (8.7)	1.00 (Reference)	1.00 (Reference)	691 (3.0)	1.00 (Reference)	1.00 (Reference)	678 (8.9)	1.00 (Reference)	1.00 (Reference)
Yes	269 (10.0)	1.14 (0.71-1.85)	1.29 (0.78-2.12)	256 (3.5)	1.15 (0.50-2.63)	1.27 (0.54-2.99)	254 (9.8)	1.13 (0.69-1.86)	1.22 (0.73-2.13)
**Ghana/Not obese**									
No	1648 (9.1)	1.00 (Reference)	1.00 (Reference)	1661 (3.9)	1.00 (Reference)	1.00 (Reference)	1646 (9.6)	1.00 (Reference)	1.00 (Reference)
Yes	81 (8.6)	1.06 (0.48-2.38)	1.04 (0.47-2.32)	81 (2.5)	1.04 (0.48-2.32)	0.86 (0.20-3.69)	81 (7.4)	0.90 (0.38-2.12)	0.89 (0.38-2.11)
**Ghana/Obese**									

No	383 (13.8)	1.00 (Reference)	1.00 (Reference)	383 (3.7)	1.00 (Reference)	1.00 (Reference)	382 (14.9)	1.00 (Reference)	1.00 (Reference)
Yes	23 (4.4)	0.31 (0.04-2.36)	0.40 (0.05-3.11)	23 (0.0)	**** (***-***)	**** (***-***)	23 (4.4)	0.29 (0.04-2.22)	0.34 (0.04-2.66)
**Stress at home/work**									
**Europe/Not obese**									
Never	855 (7.4)	1.00 (Reference)	1.00 (Reference)	847 (3.4)	1.00 (Reference)	1.00 (Reference)	831 (8.2)	1.00 (Reference)	1.00 (Reference)
Some stress	629 (8.3)	1.16 (0.79-1.70)	1.20 (0.81-1.79)	627 (1.4)	0.52 (0.24-1.12)	0.47 (0.21-1.07)	615 (7.9)	0.98 (0.66-1.43)	0.98 (0.65-1.46)
Several/Permanent stresses	247 (6.9)	0.93 (0.53-1.62)	1.15 (0.77-1.72)	239 (2.3)	0.45 (0.13-1.52)	0.49 (0.15-1.67)	236 (9.1)	0.97 (0.57-1.65)	1.04 (0.61-1.78)
**Europe/Obese**									
Never	472 (9.8)	1.00 (Reference)	1.00 (Reference)	455 (3.9)	1.00 (Reference)	1.00 (Reference)	448 (10.3)	1.00 (Reference)	1.00 (Reference)
Some stress	372 (7.5)	0.72 (0.44-1.18)	0.79 (0.47-1.32)	356 (1.4)	0.41 (0.15-1.16)	0.43 (0.15-1.21)	352 (9.5)	0.94 (0.57-1.53)	1.05 (0.62-1.76)
Several/Permanent stresses	147 (12.2)	1.32 (0.74-2.38)	1.31 (0.72-2.42)	148 (4.1)	1.05 (0.40-2.79)	1.09 (0.40-2.91)	144 (8.5)	1.36 (0.74-2.49)	1.41 (0.75-2.64)
**Ghana/Not obese**									
Never	537 (9.7)	1.00 (Reference)	1.00 (Reference)	542 (3.1)	1.00 (Reference)	1.00 (Reference)	537 (8.9)	1.00 (Reference)	1.00 (Reference)
Some stress	1055 (8.3)	0.79 (0.56-1.15)	0.74 (0.52-1.07)	1057 (3.9)	1.16 (0.65-2.09)	1.21 (0.67-2.18)	1052 (9.0)	0.92 (0.64-1.35)	0.90 (0.62-1.32)
Several/permanent stresses	319 (8.5)	0.81 (0.49-1.32)	0.73 (0.44-1.20)	323 (4.3)	1.34 (0.64-2.83)	1.43 (0.67-3.04)	319 (10.7)	1.12 (0.69-1.79)	1.09 (0.68-1.76)
**Ghana/Obese**									
Never	145 (12.4)	1.00 (Reference)	1.00 (Reference)	146 (4.1)	1.00 (Reference)	1.00 (Reference)	145 (13.8)	1.00 (Reference)	1.00 (Reference)
Some stress	221 (14.9)	1.21 (0.64-2.26)	1.05 (0.55-1.99)	219 (3.7)	0.74 (0.25-2.22)	0.83 (0.26-2.06)	219 (15.9)	1.13 (0.62-2.07)	1.02 (0.55-1.89)
Several/Permanent stresses	46 (8.7)	0.64 (0.20-2.01)	0.52 (0.16-1.65)	46 (2.2)	0.41 (0.05-3.61)	0.45 (0.05-4.01)	46 (8.8)	0.55 (0.17-1.71)	0.47 (0.14-1.50)
**Depressive symptoms**									
**Europe/Not obese**									
No	1601 (7.9)	1.00 (Reference)	1.00 (Reference)	1586 (2.2)	1.00 (Reference)	1.00 (Reference)	1558 (8.3)	1.00 (Reference)	1.00 (Reference)
Yes	121 (4.9)	0.59 (0.25-1.36)	0.65 (0.27-1.48)	117 (1.7)	0.79 (0.18-2.35)	0.86 (0.21-3.80)	115 (5.22)	0.58 (0.25-1.35)	0.64 (0.27-1.49)
**Europe/Obese**									
No	899 (9.7)	1.00 (Reference)	1.00 (Reference)	866 (3.4)	1.00 (Reference)	1.00 (Reference)	853 (9.4)	1.00 (Reference)	1.00 (Reference)
Yes	83 (7.2)	0.71 (0.29-1.67)	0.72 (0.30-1.74)	83 (1.2)	0.46 (0.06-3.52)	0.46 (0.06-3.57)	82 (8.5)	0.89 (0.40-2.01)	0.90 (0.40-2.04)

**Ghana/Not obese**									
No	1811 (8.9)	1.00 (Reference)	1.00 (Reference)	1822 (3.9)	1.00 (Reference)	1.00 (Reference)	1808 (9.4)	1.00 (Reference)	1.00 (Reference)
Yes	100 (6.0)	0.57 (0.24-1.33)	0.56 (0.24-1.32)	100 (2.0)	0.38 (0.09-1.63)	0.38 (0.09-1.63)	100 (8.0)	0.69 (0.32-1.46)	0.69 (0.32-1.47)
**Ghana/Obese**									
No	398 (13.8)	1.00 (Reference)	1.00 (Reference)	397 (3.5)	1.00 (Reference)	1.00 (Reference)	396 (14.7)	1.00 (Reference)	1.00 (Reference)
Yes	14 (0.0)	*** (****-****)	*** (****-****)	14 (7.1)	1.76 (0.21-14.89)	2.14 (0.25-8.78)	14 (7.1)	0.42 (0.05-3.32)	0.38 (0.05-3.07)

Model 1, adjusted for age and sex; Model 2, adjusted for age, sex, and educational level for Ghanaians (SSA) and length of stay for those in Europe; Abbreviations: CI, confidence interval; ACR, albumin creatinine ration; eGFR, estimated glomerular filtration rate; CKD, chronic kidney disease; OR, odds ratio, n= total number of Ghanaians living in Ghana and Europe among the various levels of PS constructs; %, proportion of individuals with CKD among the various levels of PS constructs in Ghana and Europe.

S3: Association of psychosocial stressors (PS) indicators (negative life events, discrimination, stress at work/home and depression) with albuminuria, reduced eGFR and CKD risk for Ghanaians living in Ghana and those living in Europe stratified by diabetes status

	Albuminuria (ACR ≥ 3 mg/mmol)			eGFR < 60 mL/min/1.73 m2			High to very high CKD risk (KDIGO, 2012)		
		OR (95% CI)			OR (95% CI)			OR (95% CI)	
	n (%)	Model 1	Model 2	n (%)	Model 1	Model 2	n cases (%)	Model 1	Model 2
**Negative events**									
**Europe/No diabetes**									
No	986 (8.0)	1.00 (Reference)	1.00 (Reference)	971 (2.1)	1.00 (Reference)	1.00 (Reference)	957 (7.9)	1.00 (Reference)	1.00 (Reference)
Yes	1359 (7.7)	0.95 (0.70-1.29)	0.97 (0.71-1.34)	1340 (2.0)	0.90 (0.50-1.63)	0.90 (0.48-1.68)	1315 (8.1)	1.01 (0.75-1.37)	0.99 (0.72-1.36)
**Europe/Diabetes**									
No	142 (9.9)	1.00 (Reference)	1.00 (Reference)	135 (6.7)	1.00 (Reference)	1.00 (Reference)	133 (12.8)	1.00 (Reference)	1.00 (Reference)
Yes	256 (12.5)	1.28 (0.66-2.50)	1.41 (0.71-2.82)	247 (4.9)	0.75 (0.29-1.89)	10.68 (0.27-1.77)	242 (11.2)	0.87 (0.46-1.69)	0.96 (0.49-1.88)
**Ghana/No diabetes**									
No	683 (9.1)	1.00 (Reference)	1.00 (Reference)	687 (4.1)	1.00 (Reference)	1.00 (Reference)	683 (9.8)	1.00 (Reference)	1.00 (Reference)
Yes	1447 (7.7)	0.84 (0.60-1.16)	0.82 (0.59-1.14)	1451 (3.1)	0.69 (0.42-1.13)	0.69 (0.42-1.13)	1442 (8.3)	0.82 (0.59-1.12)	0.80 (0.58-1.10)
**Ghana/Diabetes**									
No	49 (28.3)	1.00 (Reference)	1.00 (Reference)	49 (10.2)	1.00 (Reference)	1.00 (Reference)	49 (26.4)	1.00 (Reference)	1.00 (Reference)
Yes	148 (24.3)	0.78 (0.37-1.61)	0.72 (0.34-1.52)	150 (7.3)	0.55 (0.16-1.81)	0.57 (0.17-1.88)	148 (26.4)	0.93 (0.45-1.97)	0.90 (0.43-1.92)
**Discrimination**									
**Europe/No diabetes**									
No	1629 (7.9)	1.00 (Reference)	1.00 (Reference)	1609 (2.2)	1.00 (Reference)	1.00 (Reference)	1580 (8.3)	1.00 (Reference)	1.00 (Reference)
Yes	684 (6.7)	0.85 (0.60-1.21)	0.89 (0.62-1.28)	669 (1.5)	0.68 (0.33-1.40)	0.69 (0.32-1.47)	661 (7.0)	0.84 (0.59-1.20)	0.86 (0.59-1.25)
**Europe/Diabetes**									
No	270 (11.9)	1.00 (Reference)	1.00 (Reference)	258 (5.4)	1.00 (Reference)	1.00 (Reference)	252 (13.1)	1.00 (Reference)	1.00 (Reference)
Yes	126 (11.1)	0.87 (0.44-1.71)	0.89 (0.45-1.76)	122 (5.7)	1.29 (0.48-3.45)	1.25 (0.46-3.37)	121 (9.1)	0.68 (0.32-1.40)	0.69 (0.33-1.42)
**Ghana/No diabetes**									
No	1854 (8.4)	1.00 (Reference)	1.00 (Reference)	1865 (3.5)	1.00 (Reference)	1.00 (Reference)	1851 (9.0)	1.00 (Reference)	1.00 (Reference)
Yes	99 (8.1)	1.03 (0.49-2.17)	1.04 (0.49-2.19)	99 (2.0)	0.79 (0.19-3.33)	0.78 (0.18-3.29)	99 (7.1)	0.87 (0.39-1.93)	0.88 (0.39-1.95)

**Ghana/Diabetes**									
No	180 (26.7)	1.00 (Reference)	1.00 (Reference)	182 (7.7)	1.00 (Reference)	1.00 (Reference)	180 (27.2)	1.00 (Reference)	1.00 (Reference)
Yes	5 (0.0)	**** (***-***)	0.40 (0.05-3.11)	5 (0.0)	**** (***-***)	**** (***-***)	5 (0.0)	**** (***-***)	**** (***-***)
**Stress at home/work**									
**Europe/No diabetes**									
Never	1137 (7.9)	1.00 (Reference)	1.00 (Reference)	1127 (2.3)	1.00 (Reference)	1.00 (Reference)	1102 (8.0)	1.00 (Reference)	1.00 (Reference)
Some stress	860 (7.2)	0.90 (0.65-1.27)	0.96 (0.68-1.36)	850 (1.2)	0.49 (0.24-1.02)	0.49 (0.22-1.07)	835 (7.4)	0.94 (0.67-1.31)	0.99 (0.69-1.41)
Several/Permanent stresses	335 (8.4)	1.04 (0.67-1.62)	1.07 (0.68-1.70)	327 (2.1)	0.84 (0.36-1.95)	0.92 (0.39-2.17)	322 (9.6)	1.20 (0.77-1.85)	1.28 (0.82-1.99)
**Europe/Diabetes**									
Never	193 (9.8)	1.00 (Reference)	1.00 (Reference)	184 (8.2)	1.00 (Reference)	1.00 (Reference)	180 (11.7)	1.00 (Reference)	1.00 (Reference)
Some stress	142 (12.7)	1.22 (0.61-2.45)	1.38 (0.67-2.81)	134 (3.0)	0.43 (0.13-1.39)	0.40 (0.12-1.29)	133 (12.0)	1.04 (0.52-2.09)	1.13 (0.56-2.32)
Several/Permanent stresses	62 (12.9)	1.31 (0.54-3.16)	1.38 (0.56-3.41)	63 (3.2)	0.49 (0.10-2.29)	0.46 (0.10-2.23)	61 (9.8)	0.82 (0.31-2.13)	0.89 (0.34-2.35)
**Ghana/No diabetes**									
Never	628 (7.8)	1.00 (Reference)	1.00 (Reference)	634 (2.7)	1.00 (Reference)	1.00 (Reference)	628 (7.6)	1.00 (Reference)	1.00 (Reference)
Some stress	1163 (8.6)	1.06 (0.74-1.51)	0.97 (0.67-1.39)	1163 (3.7)	1.27 (0.71-2.27)	1.32 (0.74-2.34)	1158 (9.3)	1.15 (0.80-1.65)	1.10 (0.77-1.58)
Several/permanent stresses	339 (7.4)	0.88 (0.53-1.46)	0.76 (0.46-1.27)	341 (3.5)	1.24 (0.58-2.67)	1.35 (0.62-2.95)	339 (8.9)	1.07 (0.66-1.73)	1.00 (0.61-1.64)
**Ghana/Diabetes**									
Never	54 (38.9)	1.00 (Reference)	1.00 (Reference)	54 (11.1)	1.00 (Reference)	1.00 (Reference)	54 (37.0)	1.00 (Reference)	1.00 (Reference)
Some stress	116 (18.9)	**0.37 (0.37-0.77)**	**0.35 (0.17-0.75)**	116 (6.0)	0.47 (0.14-1.60)	0.45 (0.12-1.55)	116 (19.8)	**0.43 (0.21-0.89)**	**0.40 (0.19-0.86)**
Several/Permanent stresses	26 (25.0)	0.44 (0.44-1.29)	0.39 (0.13-1.19)	28 (10.7)	0.65 (0.13-3.21)	0.62 (0.12-3.23)	26 (30.8)	0.69 (0.25-1.93)	0.64 (0.22-1.87)
**Depressive symptoms**									
**Europe/No diabetes**									
No	2154 (8.0)	1.00 (Reference)	1.00 (Reference)	2122 (2.1)	1.00 (Reference)	1.00 (Reference)	2087 (8.1)	1.00 (Reference)	1.00 (Reference)
Yes	167 (5.4)	0.63 (0.32-1.25)	0.69 (0.34-1.38)	163 (2.0)	0.65 (0.15-2.72)	0.72 (0.17-3.04)	161 (6.8)	0.58 (0.25-1.35)	0.86 (0.46-1.64)
**Europe/Diabetes**									

No	351 (12.0)	1.00 (Reference)	1.00 (Reference)	335 (35.9)	1.00 (Reference)	1.00 (Reference)	329 (12.5)	1.00 (Reference)	1.00 (Reference)
Yes	39 (10.3)	0.79 (0.26-1.35)	0.73 (0.24-2.19)	39 (2.6)	0.49 (0.06-3.97)	0.49 (0.06-3.99)	38 (7.9)	0.89 (0.40-2.01)	0.60 (0.17-2.08)
**Ghana/No diabetes**									
No	2027 (8.3)	1.00 (Reference)	1.00 (Reference)	2035 (3.4)	1.00 (Reference)	1.00 (Reference)	2022 (8.8)	1.00 (Reference)	1.00 (Reference)
Yes	102 (4.9)	0.50 (0.20-1.25)	0.48 (0.19-1.19)	102 (2.9)	0.64 (0.19-2.13)	0.67 (0.20-2.21)	102 (7.8)	0.69 (0.32-1.46)	0.71 (0.34-1.51)
**Ghana/Diabetes**									
No	185 (26.5)	1.00 (Reference)	1.00 (Reference)	187 (8.7)	1.00 (Reference)	1.00 (Reference)	185 27.6)	1.00 (Reference)	1.00 (Reference)
Yes	12 (8.3)	0.27 (0.03-2.13)	0.25 (0.03-2.01)	12 (0.0)	*** (****-****)	*** (***-***)	12 (8.3)	0.42 (0.05-3.32)	0.27 (0.03-2.19)

Model 1, adjusted for age and sex; Model 2, adjusted for age, sex, and educational level for Ghanaians (SSA) and length of stay for those in Europe; Abbreviations: CI, confidence interval; ACR, albumin creatinine ration; eGFR, estimated glomerular filtration rate; CKD, chronic kidney disease; OR, odds ratio, n= total number of Ghanaians living in Ghana and Europe among the various levels of PS constructs; %, proportion of individuals with CKD among the various levels of PS constructs in Ghana and Europe.

S4: Association of psychosocial stressors (PS) indicators (negative life events, discrimination, stress at work/home and depression) with albuminuria, reduced eGFR and CKD risk for Ghanaians living in Ghana and those living in Europe stratified by hypertensive status

	Albuminuria (ACR ≥ 3 mg/mmol)			eGFR < 60 mL/min/1.73 m2			High to very high CKD risk (KDIGO, 2012)		
		OR (95% CI)			OR (95% CI)			OR (95% CI)	
	n (%)	Model 1	Model 2	n (%)	Model 1	Model 2	n cases (%)	Model 1	Model 2
**Negative events**									
**Europe/No hypertension**									
No	568 (6.9)	1.00 (Reference)	1.00 (Reference)	562 (1.3)	1.00 (Reference)	1.00 (Reference)	551 (7.1)	1.00 (Reference)	1.00 (Reference)
Yes	743 (5.0)	0.72 (0.45-1.15)	0.78 (0.48-1.27)	740 (0.8)	0.59 (0.19-1.77)	0.60 (0.20-1.83)	724 (5.1)	0.71 (0.44-1.13)	0.75 (0.46-1.22)
**Europe/Hypertension**									
No	560 (9.6)	1.00 (Reference)	1.00 (Reference)	544 (4.0)	1.00 (Reference)	1.00 (Reference)	539 (10.0)	1.00 (Reference)	1.00 (Reference)
Yes	872 (11.4)	1.22 (0.86-1.73)	1.20 (0.83-1.73)	847 (3.9)	0.95 (0.54-1.67)	0.89 (0.49-1.62)	833 (11.6)	1.19 (0.83-1.69)	1.13 (0.78-1.62)
**Ghana/No Hypertension**									
No	540 (6.3)	1.00 (Reference)	1.00 (Reference)	543 (2.2)	1.00 (Reference)	1.00 (Reference)	540 (6.9)	1.00 (Reference)	1.00 (Reference)
Yes	1118 (5.6)	0.88 (0.57-1.36)	0.84 (0.54-1.31)	1124 (1.9)	0.82 (0.39-1.69)	0.86 (0.41-1.79)	1115 (6.4)	0.93 (0.61-1.40)	0.90 (0.59-1.36)
**Ghana/Hypertension**									
No	192 (21.9)	1.00 (Reference)	1.00 (Reference)	193 (10.9)	1.00 (Reference)	1.00 (Reference)	192 (22.4)	1.00 (Reference)	1.00 (Reference)
Yes	476 (18.1)	0.78 (0.52-1.18)	0.79 (0.52-1.20)	476 (6.9)	0.58 (0.32-1.04)	0.58 (0.33-1.05)	474 (18.4)	0.77 (0.50-1.16)	0.76 (0.51-1.16)
**Discrimination**									
**Europe/No hypertension**									
No	916 (6.3)	1.00 (Reference)	1.00 (Reference)	907 (0.8)	1.00 (Reference)	1.00 (Reference)	885 (6.8)	1.00 (Reference)	1.00 (Reference)
Yes	379 (3.7)	0.60 (0.33-1.09)	0.65 (0.36-1.21)	378 (1.1)	1.35 (0.39-4.74)	1.37 (0.38-4.89)	374 (3.2)	**0.47 (0.25-0.89)**	**0.51 (0.27-0.97)**
**Europe/Hypertension**									
No	983 (10.5)	1.00 (Reference)	1.00 (Reference)	960 (4.4)	1.00 (Reference)	1.00 (Reference)	947 (10.9)	1.00 (Reference)	1.00 (Reference)
Yes	431 (10.7)	1.03 (0.71-1.49)	1.05 (0.72-1.54)	413 (3.2)	0.73 (0.38-1.39)	0.75 (0.38-1.48)	408 (11.0)	1.03 (0.71-1.49)	1.04 (0.71-1.53)
**Ghana/No hypertension**									
No	1427 (5.7)	1.00 (Reference)	1.00 (Reference)	1437 (2.2)	1.00 (Reference)	1.00 (Reference)	1424 (6.5)	1.00 (Reference)	1.00 (Reference)

Yes	81 (7.4)	1.30 (0.55-3.09)	1.30 (0.55-3.10)	81 (0.0)	**** (***-***)	**** (***-***)	81 (7.4)	1.21 (0.51-2.88)	1.22 (0.51-2.89)
**Ghana/Hypertension**									
No	606 (20.3)	1.00 (Reference)	1.00 (Reference)	609 (7.9)	1.00 (Reference)	1.00 (Reference)	606 (20.3)	1.00 (Reference)	1.00 (Reference)
Yes	23 (8.7)	0.38 (0.09-1.68)	0.40 (0.09-1.74)	23 (8.7)	1.27 (0.28-5.70)	1.20 (0.26-5.44)	23 (4.4)	0.19 (0.03-1.44)	0.19 (0.03-1.45)
**Stress at home/work**									
**Europe/No hypertension**									
Never	644 (6.1)	1.00 (Reference)	1.00 (Reference)	640 (1.3)	1.00 (Reference)	1.00 (Reference)	625 (5.8)	1.00 (Reference)	1.00 (Reference)
Some stress	468 (4.5)	0.74 (0.43-1.27)	0.81 (0.46-1.43)	464 (0.4)	0.37 (0.07-1.74)	0.41 (0.09-2.01)	457 (4.8)	0.84 (0.49-1.45)	0.91 (0.52-1.60)
Several/Permanent stresses	192 (6.8)	1.07 (0.56-2.05)	1.19 (0.61-2.29)	191 (1.6)	1.49 (0.38-5.78)	1.52 (0.39-5.98)	186 (8.1)	1.38 (0.74-2.60)	1.49 (0.79-2.84)
**Europe/Hypertension**									
Never	686 (10.2)	1.00 (Reference)	1.00 (Reference)	665 (5.4)	1.00 (Reference)	1.00 (Reference)	657 (11.1)	1.00 (Reference)	1.00 (Reference)
Some stress	534 (11.1)	1.03 (0.71-1.49)	1.05 (0.72-1.54)	520 (2.3)	**0.49 (0.25-0.96)**	**0.47 (0.23-0.95)**	511 (10.9)	0.97 (0.67-1.41)	0.99 (0.67-0.47)
Several/Permanent stresses	205 (11.2)	1.11 (0.67-1.82)	1.12 (0.67-1.87)	199 (3.0)	0.56 (0.23-1.37)	0.58 (0.24-1.43)	197 (11.2)	0.99 (0.59-1.64)	1.03 (0.61-1.74)
**Ghana/No hypertension**									
Never	495 (5.5)	1.00 (Reference)	1.00 (Reference)	498 (1.4)	1.00 (Reference)	1.00 (Reference)	495 (5.9)	1.00 (Reference)	1.00 (Reference)
Some stress	899 (5.9)	1.07 (0.66-1.73)	0.99 (0.61-1.60)	901 (2.2)	1.30 (0.54-3.15)	1.39 (0.57-3.37)	896 (6.5)	1.04 (0.65-1.65)	1.00 (0.63-1.59)
Several/permanent stresses	263 (5.7)	1.01 (0.53-1.95)	0.89 (0.46-1.72)	267 (2.6)	1.69 (0.58-5.01)	1.89 (0.63-5.65)	263 (7.6)	1.21 (0.67-2.20)	1.15 (0.63-2.11)
**Ghana/Hypertension**									
Never	187 (22.9)	1.00 (Reference)	1.00 (Reference)	190 (8.4)	1.00 (Reference)	1.00 (Reference)	187 (20.9)	1.00 (Reference)	1.00 (Reference)
Some stress	379 (18.2)	0.74 (0.48-1.14)	0.68 (0.43-1.05)	377 (7.9)	0.95 (0.50-1.80)	0.99 (0.51-1.91)	377 (19.4)	0.90 (0.58-1.40)	0.87 (0.55-1.36)
Several/Permanent stresses	102 (15.7)	0.62 (0.33-1.17)	0.54 (0.28-1.04)	102 (7.8)	0.92 (0.37-2.26)	0.97 (0.39-2.43)	102 (17.7)	0.79 (0.42-1.49)	0.76 (0.40-1.43)
**Depressive symptoms**									
**Europe/No hypertension**									

No	1195 (5.9)	1.00 (Reference)	1.00 (Reference)	1187 (1.1)	1.00 (Reference)	1.00 (Reference)	1162 (6.0)	1.00 (Reference)	1.00 (Reference)
Yes	101 (3.9)	0.59 (0.21-1.65)	0.64 (0.23-1.82)	99 (1.0)	1.18 (0.15-2.72)	1.27 (0.16-10.17)	98 (5.1)	0.77 (0.30-1.97)	0.84 (0.33-2.17)
**Europe/Hypertension**									
No	1310(10.9)	1.00 (Reference)	1.00 (Reference)	1270 (4.1)	1.00 (Reference)	1.00 (Reference)	1254 (11.2)	1.00 (Reference)	1.00 (Reference)
Yes	105 (8.6)	0.76 (0.37-1.53)	0.75 (0.37-1.55)	103 (1.9)	0.52 (0.12-2.18)	0.56 (0.13-2.38)	101 (8.9)	0.77 (0.38-1.58)	0.80 (0.39-1.63)
**Ghana/No hypertension**									
No	1575 (5.9)	1.00 (Reference)	1.00 (Reference)	1584 (2.1)	1.00 (Reference)	1.00 (Reference)	1572 (6.7)	1.00 (Reference)	1.00 (Reference)
Yes	82 (2.4)	0.36 (0.09-1.51)	0.35 (0.08-1.44)	82 (1.2)	0.39 (0059-2.95)	0.39 (0.05-2.97)	82 (3.7)	0.44 (0.13-1.42)	0.43 (0.13-1.40)
**Ghana/Hypertension**									
No	636 (19.5)	1.00 (Reference)	1.00 (Reference)	637 (8.2)	1.00 (Reference)	1.00 (Reference)	634 (19.6)	1.00 (Reference)	1.00 (Reference)
Yes	32 (12.5)	0.59 (0.20-1.72)	0.59 (0.20-1.74)	32 (6.3)	0.74 (0.17-3.25)	0.76 (0.17-3.33)	32 (18.8)	0.94 (0.0.37-2.37)	0.94 (0.38-2.37)

Model 1, adjusted for age and sex; Model 2, adjusted for age, sex, and educational level for Ghanaians (SSA) and length of stay for those in Europe; Abbreviations: CI, confidence interval; ACR, albumin creatinine ration; eGFR, estimated glomerular filtration rate; CKD, chronic kidney disease; OR, odds ratio, n= total number of Ghanaians living in Ghana and Europe among the various levels of PS constructs; %, proportion of individuals with CKD among the various levels of PS constructs in Ghana and Europe.
