# Supplementary material for: Cross-sectional study of association between psychosocial stressors with chronic kidney disease among migrant and non-migrant Ghanaians living in Europe and Ghana: the RODAM study
Source: BMJ Open. 2019 Aug 1;9(8):e027931. doi: 10.1136/bmjopen-2018-027931 (PMC6688695; doi:10.1136/bmjopen-2018-027931)
Supplement: Author's manuscript [file bmjopen-2018-027931.draft_revisions.pdf]

BMJ Open

BMJ Open is committed to open peer review. As part of this commitment we make the peer review history of every article we publish publicly available.

When an article is published we post the peer reviewers' comments and the authors' responses online. We also post the versions of the paper that were used during peer review. These are the versions that the peer review comments apply to.

The versions of the paper that follow are the versions that were submitted during the peer review process. They are not the versions of record or the final published versions. They should not be cited or distributed as the published version of this manuscript.

BMJ Open is an open access journal and the full, final, typeset and author-corrected version of record of the manuscript is available on our site with no access controls, subscription charges or pay-per-view fees (http://bmjopen.bmj.com).

If you have any questions on BMJ Open's open peer review process please email info.bmjopen@bmj.com

A CROSS SECTIONAL STUDY OF ASSOCIATION BETWEEN PSYCHOSOCIAL STRESSORS WITH CHRONIC KIDNEY DISEASE AMONG MIGRANT AND NON-MIGRANT GHANAIANS: THE RODAM STUDY

Journal:	*BMJ Open*
Manuscript ID	bmjopen-2018-027931
Article Type:	Research
Date Submitted by the Author:	17-Nov-2018
Complete List of Authors:	Adjei Nana, David ; University of Ghana, Department of Medical Laboratory Sciences; University of Amsterdam, Department of Public Health<br>Stronks, Karien; Academic Medical Center , Department of Public Health<br>Adu, Dwomoa; Korle-bu Teaching Hospital, Department of Medicine<br>Beune, Erik; AMC<br>Meeks, Karlijn; AMC, Public Health<br>Smeeth, Liam; London School of Hygiene and Tropical Medicine,<br>Addo, Juliet; London School of Hygiene and Tropical Medicine, Non Communicable Disease Epidemiology<br>Owusu-Dabo, Ellis; Kwame Nkrumah University of Science and Technology, Kumasi Centre for Collaborative Research in Tropical Medicine<br>Klipstein-Grobusch, Kerstin; 1 Julius Global Health, Julius Center for Health Sciences and Primary Care, University Medical Centre Utrecht, Utrecht, The Netherlands<br>Mockenhaupt, Frank; Charité – University Medicine, Berlin, Institute of Tropical Medicine and International Health<br>Schulze, Matthias; German Institute of Human Nutrition Potsdam-Rehbruecke<br>Danquah, Ina; German Institute of Human Nutrition, Molecular Epidemiology; Charite Universitatsmedizin Berlin, Institute for Social Medicine, Epidemiology and Health Economy<br>Spranger, Joachim; Department of Endocrinology and Metabolism, 1. Charité-University Medicine Berlin, Berlin, Germany.<br>Bahendeka, Silver; St. Francis Hospital Nsambya,<br>Agyemang, Charles; Academic Medical centre, University of Amsterdam, Department of Public Health
Keywords:	Chronic Kidney Disease, Psychosocial stressors, migrants, rodam study, Europe, Ghana

For peer review only - http://bmjopen.bmj.com/site/about/guidelines.xhtml

A CROSS SECTIONAL STUDY OF ASSOCIATION BETWEEN PSYCHOSOCIAL STRESSORS WITH CHRONIC KIDNEY DISEASE AMONG MIGRANT AND NON-MIGRANT GHANAIANS: THE RODAM STUDY

David N. Adjei, MSc, PhD[1,2]; Karien Stronks, MSc, PhD[1]; Dwomoa Adu, MD[3]; Erik Beune, MSc, PhD [1]; Karlijn Meeks, MSc, PhD [1]; Liam Smeeth, MD, PhD[4] ; Juliet, Addo, MD, PhD [4]; Ellis Owuso-Dabo, MSc, PhD [5], Kerstin Klipstein-Grobusch, MSc, PhD [6,7]; Frank P. Mockenhaupt, MD, PhD [8] ; Matthias B. Schulze, MSc, PhD [9]; Ina, Danquah, MSc, PhD [9,10] ; Joachim, Spranger, MD, PhD [11,12,13]; Silver Bahendeka, MD, PhD[14]; Charles Agyemang, MPH, PHD[1]

1. Department of Public Health, Academic Medical Center, University of Amsterdam, Amsterdam, Amsterdam Public Health Research Institute, Amsterdam, The Netherlands.
2. Department of Medical Laboratory Sciences, School of Biomedical and Allied Health Sciences, College of Health Sciences, University of Ghana, Accra, Ghana.
3. Department of Medicine, School of Medicine and Dentistry, University of Ghana and Korle-Bu Teaching Hospital, Accra, Ghana.
4. Department of Non-Communicable Disease Epidemiology, London School of Hygiene and Tropical Medicine, London, United Kingdom.
5. Kumasi Centre for collaborative Research, KNUST, Kumasi, Ghana.
6. Julius Global Health, Julius Center for Health Sciences and Primary Care, University Medical Centre, Utrecht University, The Netherlands
7. Division of Epidemiology & Biostatistics, School of Public Health, Faculty of Health Sciences, University of the Witwatersrand, Johannesburg, South Africa
8. Institute of Tropical Medicine and International Health, Charité -University Medicine Berlin, Augustenburger Platz 1, 13353 Berlin, Germany.

For peer review only - http://bmjopen.bmj.com/site/about/guidelines.xhtml

9. Department of Molecular Epidemiology, German Institute of Human Nutrition Potsdam-Rehbrücke, Nuthetal, Germany.
10. Charité - Universitaetsmedizin Berlin, Institute for Social Medicine, Epidemiology and Health Economics, Berlin, Germany.
11. Department of Endocrinology and Metabolism, Charité-University Medicine Berlin, Berlin, Germany.
12. German Centre for Cardiovascular Research (DZHK), Berlin, Germany.
13. Center for Cardiovascular Research (CCR), Charité-University, Medicine, Berlin, Germany.
14. MKPGMS - Uganda Martyrs University, Kampala, Uganda.

Address correspondence to David Nana Adjei, MSc, Department of Public Health, Academic Medical Centre, University of Amsterdam, Meibergdreef 9, 1105 AZ Amsterdam, the Netherlands, School Biomedical and Allied Health Sciences, Medical Laboratory Sciences, University of Ghana, E-mail: dna@chs.edu.gh, d.n.adjei@amc.uva.nl, Tel: +233236717850

For peer review only

For peer review only

Abstract

For peer review only - http://bmjopen.bmj.com/site/about/guidelines.xhtml

Objectives: The association between Psychosocial stressors (PS) and CKD within sub-Saharan African (SSA) populations is unknown. We examined the association between PS and CKD prevalence among rural and urban Ghanaians and their migrants living in three European cities. We also assessed if the influence of PS on CKD is partially mediated by primary risk factors (hypertension and diabetes) of CKD.

Methods: A multi-centred cross sectional baseline data from the Research on Obesity and Diabetes among African Migrants (RODAM) study. A random sample of 5,659 adults (Europe, 3167, rural, 1,043, Urban Ghana, 1,449, Ghana) aged 25 to 70 years. PS defined by negative life events, perceived discrimination, perceived stress at work/home and depressive symptoms. Three CKD outcomes were considered using the 2012 KDIGO (Kidney Disease: Improving Global Outcomes) severity of CKD classification. Comparisons between PS and CKD were made using logistic regression analyses across all sites.

Results: We observed higher proportion of Ghanaians living in SSA with negative life events (68.7%) and perceived permanent stress (15.9%). Depressive symptoms (7.5%) and perceived discrimination (29.7%) were more common among Ghanaians living in Europe. No association was observed between either one of the four constructs of PS and CKD across all the sites except for those with some level of stress at work/home in Berlin that had a higher risk of CKD (2.78, 95% C.I. 1.43-5.43).

Conclusion: Our study shows no convincing evidence for associations between stress as indicated by four PS constructs and prevalence of CKD. Further studies aimed at identifying potential factors driving the high prevalence of CKD among these populations are needed.

For peer review only - http://bmjopen.bmj.com/site/about/guidelines.xhtml

Index Words: Chronic kidney disease; psychosocial stressors; risk factor; migrants; RODAM study, Europe, Ghana

Strengths and limitations of the study

- This study used all three categories of CKD definitions (albuminuria, reduced eGFR and CKD risk) by KDIGO 2012 in assessing association of SS with CKD across all sites. This provided more detailed information on CKD outcomes.
- All sites in our study used well standardized study protocols and this eliminated intra protocol variability.

For peer review only - http://bmjopen.bmj.com/site/about/guidelines.xhtml

- The use of four constructs of psychosocial stressors (negative life events, perceived discrimination, perceived stress at work/home and depressive symptoms) provided a robust approach to assessing SS associations with CKD.

- The limitation of intra laboratory variability in earlier studies was eliminated using the same standard operating procedures in the same laboratory for running all samples for all sites.

- PS is captured and experienced in different magnitude across different populations. We were unable to ascertain if PS as defined in this study was adequately captured among Ghanaians living in rural and urban Ghana.

For peer review only

Introduction

Worldwide, Chronic Kidney Disease (CKD) is a leading cause of mortality and morbidity with several risk factors (diabetes mellitus, obesity, hypertension and cardiovascular disease) (1). The epidemiologic transition in low-and-middle income countries (LMICs) shows increased burden of these risk factors (2-4). CKD's high morbidity and mortality is mostly driven by uncontrolled comorbidities such as diabetes and hypertension (5, 6). CKD treatment and management cost is very high and not sustainable even in high-income countries and this underscores the need for prevention (7). Available literature has shown that both individual and community level economic factors influence CKD (8-10). However, after adjusting for both individual and community level socioeconomic position, differences in CKD risk among different populations remained (8, 10, 11). These findings seem to suggest other social environmental factors may be driving CKD prevalence and progression in high-risk populations. Recent studies have shown that whereas Ghanaians living in Europe have a higher CKD risk compared to their host nation populations, they have a lower CKD risk compared with their peers living in urban Ghana (12). The increased risk of CKD observed in urban Ghana was not fully explained by conventional risk factors (12) and socio-economic status (13). This underscores the need to identify other modifiable risk factors of CKD for prevention, optimum treatment and efficient management.

For peer review only - http://bmjopen.bmj.com/site/about/guidelines.xhtml

Evidence shows that where an individual works or stays influences his or her physiological wellbeing leading to an increased risk of chronic diseases (14, 15). Thus, migrants' physiological wellbeing are influenced by the environment (host nations) they move to stay. The association between PS and CKD as well as the biological pathways through which PS influences CKD progression is poorly understood and complex (5). Despite this, PS have been reported to be associated with alteration in the sympathetic/autonomic nervous system activity leading to higher rates of traditional risk factors of CKD including hypertension and diabetes (16, 17). A link between stress, hypertension, diabetes and CKD through innervation of all segments of the kidney by renal sympathetic nerves has been suggested (18). Also, environmental stressors have been reported to contribute to the development of insulin resistance, metabolic syndrome, obesity and diabetes which if uncontrolled leads to CKD incidence (19, 20). Thus, the above-mentioned risk factors of CKD may explain the link between PS and CKD.

However, studies linking psychosocial stressors (PS) to CKD prevalence and progression vary greatly among different geographical populations. (5, 21-26). Specifically, in the USA whereas no association was found between PS and CKD (26-28) another study reported lower prevalence of CKD was associated with greater life stressors at baseline (26). In contrast, in the Netherlands depressive and anxiety symptoms were observed to be common among CKD patients and such patients had increased risk of poor clinical outcomes (22). Similarly, a study conducted in Korea reported a positive relationship between depressive symptoms and CKD (21). These observations suggest differential impact of PS at different geographical locations. For

For peer review only - http://bmjopen.bmj.com/site/about/guidelines.xhtml

example, discrimination among migrants may differ greatly between host population and from their SSA compatriots. Specifically, some studies have reported differences in PS among rural and urban populations (29).

Current literature on the association between PS and CKD among sub-Saharan African populations and their migrants in Europe is scanty and uncertain. We therefore sought to determine the association between PS and CKD prevalence among Ghanaians in rural and urban Ghana and their migrants living in three European cities. Furthermore, we examined the influence of psychosocial stressors on risk factors (obesity, diabetes and hypertension) of CKD.

Methods

Study population and study design

For this study, data from the Research on Obesity & Diabetes among African Migrants (RODAM) study, a multi-centre cross sectional study, were used. The rationale, conceptual framework, design and methodology of the RODAM study have been described in detail elsewhere (12, 13, 30, 31). To summarize, the study was conducted from 2012 to 2015. Ghanaians aged 25-70 years living in rural and urban communities in Ghana as well as in three European cities (Amsterdam, Berlin and London) were included in this study. We standardized data collection across all sites. The ethics committees in Ghana, the Netherlands, Germany and the UK approved the study protocol prior to data collection. Informed consent was obtained from each participant prior to enrollment in the study. In Ghana, participants were randomly drawn

For peer review only - http://bmjopen.bmj.com/site/about/guidelines.xhtml

For peer review only

from a list of 30 enumeration areas in the Ashanti region based on the 2010 population census. These enumeration areas came from both rural areas and two purposively selected urban cities (Kumasi and Obuasi). For Ghanaians in Amsterdam, we randomly drew participants from the Municipal register. This register holds data on country of birth of citizens and their parents, thus allowing for sampling based on the Dutch standard indicator for ethnic origin. London lacked a population register for migrant groups. Thus, Ghanaian organizations served as sampling frame for the study. Lists of these organizations were obtained from the Ghanaian Embassy and the Association of Ghanaian Churches in the UK in the boroughs known to have the greatest concentration of Ghanaians. Members were selected from the lists of all members of these organizations. In Berlin, the registration office of the federal state of Berlin provided a list of Ghanaian individuals in Berlin but this resulted in low response rate. Because of this, a change was made to use lists of Ghanaian churches and organizations as the sampling frame. Across all sites in Europe, all selected participants were sent a written invitation combined with written information (information sheet) regarding the study and a response card. The participants were contacted by phone to schedule a date and location of the interview with a trained research assistant or opt for the self-administration of the paper questionnaire or digital online version depending on the preference of the participant. After the completion of the questionnaire, a date for physical examination was then scheduled after a positive response. The participants were instructed to fast from 10.00 p.m. the night before the physical examination. The response rate was 76% in rural Ghana and 74% in urban Ghana. In London, of those individuals who were registered in the various Ghanaian

For peer review only - http://bmjopen.bmj.com/site/about/guidelines.xhtml

organizations and were invited, 75% agreed and participated in the study, while in Berlin, this figure was 68%, and 53% in Amsterdam. For the current study, 5898 participants with data available on both questionnaire data and physical measurements were used. Individuals who were outside the age range of 25-70 years (n=239) were excluded because not all the study sites had individuals outside this age range resulting in 5659 individuals comprising of 2492 from rural and urban Ghana and 3167 from the three European cities. In the conduct of analysis, we further excluded individuals with no data on CKD and all other indicators (n=52), resulting in a data set of 5607 participants for analysis.

Measurements

Covariates

Demographic and lifestyle factors

For this study, we obtained information on demographics, educational level and lifestyle factors (smoking and physical activity) by questionnaire. Physical examinations were performed across all sites using validated devices per standardized operational procedures. Weight was measured in light clothing and without shoes with SECA 877 scales to the nearest 0.1 kg. Height was measured without shoes with a portable stadiometer (SECA 217) to the nearest 0.1 cm. Body mass index (BMI) was calculated as weight (kg) divided by height squared (m2). Overweight was defined as BMI of 25 to < 30 kg/m^2 and obesity as BMI ≥30 kg/m^2. Waist circumference was measured in centimetres at the midpoint between the lower rib and the upper margin of the iliac

For peer review only - http://bmjopen.bmj.com/site/about/guidelines.xhtml

crest. We used the same assessor for each participant in measuring all anthropometrics and each was measured twice; the average of the two measurements was used for analyses.

Predictor: SS

For this study, four constructs of psychosocial stress (discrimination, perceived stress at work or at home, negative life events and depressive symptoms) were used as explanatory variables.

Perceived discrimination

Everyday discrimination as perceived by participants was reported as routinely experiencing instances of unfair treatment. We used the the Everyday Discrimination Scale (EDS). The EDS comprises of a 9-items which rates the frequency at which participants experience daily mistreatment and it focuses on being treated with less courtesy or less respect, receiving poorer service than other people or being called names or insulted. Participants had the option of rating each of the 9-items from "never" = 1 to "very often" = 5. The obtained scores were summed and an average of the scores was computed to obtain a final score of 1 to 5. This scale was used because it is commonly used for self-reported discrimination (32), with consistent high reliability among a variety of ethnicities (33), comprising African migrants in the Netherland (34).

Perceived stress at work or at home

We defined perceived stress at work or at home as "sense of irritation, filled with anxiety, or as having difficulties in sleeping because of circumstances at work or at

For peer review only - http://bmjopen.bmj.com/site/about/guidelines.xhtml

home". We used the psychological stress scale created by the INTERHEART study (35). Participants in the study were asked about their opinion on frequency of stress at work and at home, and could answer "never", "some periods", "several periods", or "continually". Both answers were then combined into a composite score and graded into four categories: never experienced to experienced permanent stress at home or at work (35). Due to the very small numbers in the permanent periods of stress group, we combined experienced several periods of stress at home or at work and permanent periods of stress at home or at work.

Negative life events

The presence of major negative life events among participants was perceived as any event that could cause acute stress to an individual. We therefore applied the well-validated and widely used List of Threatening Experiences (LTE) (36, 37). The scale comprised of 12 unpleasant events participants perceived to have experienced in the past 12 months. We used a slightly-altered version of LTE consisting of 9 unpleasant items. We dichotomized participants into two groups namely "no negative life events" and "one or more events" and participants in the second category were expected to have higher levels of stress (37).

Depressive symptoms

Depressive symptoms were measured by the 9-item Patient Health Questionnaire (PHQ-9). The PHQ-9 consists of nine items, with a response scales 0 'not at all', 1 'on several days', 2 'on more than half of the days' and 3 'nearly every day'. A participant was considered to be in a significant depressed mood (SDM) when one or

For peer review only - http://bmjopen.bmj.com/site/about/guidelines.xhtml
For peer review only

both of the items 1 (little interest or pleasure in doing things) and 2 (feeling down, depressed, or hopeless) were answered with at least ‘on more than half of the days’, and at least 5 of the 9 items were answered with at least ‘on more than half of the days’(38).

Co-morbidity factors

Blood pressure (BP) was measured three times using a validated semi-automated device (The Microlife WatchBP home) with appropriate cuffs in a sitting position after at least 5min rest. The mean of the last two BP measurements was used in the analyses. Hypertension was defined as systolic BP 140mmHg and/or diastolic BP 90mmHg, and/or being on antihypertensive medication treatment, and/or self-reported hypertension. Trained research assistants in all sites collected fasting venous blood samples according to standard operation procedures, and then temporarily stored at the local research location. The stored blood samples from the local research centres were transported to Berlin, Germany, according to standardized procedures, for biochemical analyses. This was done to avoid intra-laboratory variability. Fasting plasma glucose concentration was measured using an enzymatic method (hexokinase). We defined Type 2 diabetes according to the World Health Organization diagnostic criteria (fasting glucose 7.0mmol/L, and/or current use of medication prescribed to treat diabetes, and/or self-reported diabetes) (39). We assessed concentration of total cholesterol using colorimetric test kits. All biochemical analyses were performed using an ABX Pentra 400 Chemistry Analyzer (ABX Pentra; Horiba 90 ABX, Germany). Hypercholesterolaemia was defined as total cholesterol level ≥6.22mmol/L. Serum creatinine concentration (in mol/L) was determined by a kinetic colorimetric

For peer review only

For peer review only - http://bmjopen.bmj.com/site/about/guidelines.xhtml

spectrophotometric isotope dilution mass spectrometry calibration method (Roche Diagnostics).

Outcome: CKD prevalence

We asked participants to bring an early morning urine sample for the analyses of albuminuria and creatinine levels. Urinary albumin concentration (in µmol/L) was measured by an immunochemical turbidimetric method (Roche Diagnostics). Urinary creatinine concentration (in I mol/L) was measured by a kinetic spectrophotometric method (Roche Diagnostics). Extensive quality checks were done inclusive of blinded serial measurements. Estimated glomerular filtration rate (eGFR) was calculated using the CKDEPI (CKD Epidemiology Collaboration) creatinine equation (40). Urinary albumin-creatinine ratio (ACR; expressed in mg/mmol) was calculated by taking the ratio between urinary albumin and urinary creatinine. eGFR and albuminuria were categorized according to the 2012 KDIGO classification (41). eGFR was categorized as follows: G1, 90mL/min/1.73m^2 (normal kidney function); G2, 60-89mL/min/1.73m^2 (mildly decreased); G3a, 45-59mL/min/1.73m^2 (mildly to moderatelydecreased); G3b, 30-44mL/min/1.73m^2 (moderately to severely decreased); G4, 15-29 mL/min/1.73 m^2 (severely decreased); and G5, <15mL/min/1.73m^2 (kidney failure). Albuminuria categories were derived from ACR and were as follows: A1, < 3mg/mmol (normal to mildly increased); A2, 3-30mg/mmol (moderately increased); and A3, > 30mg/mmol (severely increased). CKD status was categorized according to severity of kidney disease (green, low risk; yellow, moderately increased risk; orange, high risk; and red, very high risk) using the combination of eGFR (G1-G5) and albuminuria (A1-A3) levels

For peer review only - http://bmjopen.bmj.com/site/about/guidelines.xhtml

defined by the 2012 KDIGO guideline (42). Due to the small number of participants in the very high-risk category of CKD (n=27), the high and very high-risk groups were combined. Because of the small number of participants in the severely increased albuminuria category (A3, n=62), we defined albuminuria as ACR 3mg/ mmol by combining the moderately increased (A2) and severely increased (A3) categories.

Covariates assessed were age, sex, educational level and length of stay in Europe, hypertension and diabetes. Length of stay was assessed for Ghanaian migrants only. Length of stay was defined as the number of years lived in Europe at the time of data collection. Length of stay was controlled for due to evidence suggesting that it influences mental health (43). Other covariates were hypertension, obesity and diabetes.

Patient and Public Involvement

Community leaders were involved in the recruitment of patients. These comprised of religious communities (churches and mosques), endorsement from local key leaders and establishing relationships with healthcare organizations. We also provided information on the study by involving the local media (radio and television stations). We sent letters to all selected health and community authorities to notify participants of the study. Team members were sent to the various communities to stay among the community and organize mini clinics for a period of 1-2 weeks. Results of the study were disseminated through seminars, durbars and via radio and television stations.

Statistical methods

Characteristics of participants were expressed as absolute numbers and percentages for categorical variables and means and standard deviations for continuous variables. Odds ratios (ORs) and their corresponding 95% confidence intervals (CIs) and adjusted

For peer review only - http://bmjopen.bmj.com/site/about/guidelines.xhtml

CIs were estimated by means of binary logistic regression analyses to study odds of albuminuria (ACR>3 mg/mmol, A2-A3, moderately to severely increased albuminuria), reduced kidney function (eGFR< 60 mL/min/1.73m^2, G3-G5 moderately to severely decreased kidney function) and increased CKD risk (high and very high CKD risk), with adjustments for covariates (44). The Spearman's correlation test was used to test for associations between all four constructs of PS. Two models were used to examine the data. Model 1 was adjusted for age and sex, model 2 was adjusted for age and sex and educational level for Ghanaians living in SSA while age, sex, educational level and length of stay for Ghanaians living in Europe (45-47). Model 3 was adjusted for sex, age, educational level and conventional risk factors (hypertension, diabetes, hypercholesterolemia, BMI, physical activity and smoking status) of CKD. The analyses were performed for all 4 constructs of PS using individuals who have not experienced either of the PS per outcome as reference. All tests were stratified per sites, Ghanaians living in SSA and Europe; Ghanaians living in rural and urban Ghana and Ghanaians living in Amsterdam, Berlin and London due to an observed interaction between PS and site. Furthermore, the analysis were stratified for those with and without obesity, diaetes, hypertension across all sites. All analyses were performed using STATA, version 14.0 (StataCorp LP).

Results

Characteristics of the study population

Participants characteristics are shown in Table 1. Ghanaians living in Ghana were older than their peers living in Europe. There were more females compared with males

For peer review only - http://bmjopen.bmj.com/site/about/guidelines.xhtml

in both Ghana and Europe. Ghanaians living in Ghana were significantly less educated than those living in Europe. Higher proportion of Ghanaians living in Ghana had experienced negative life events in the last 12 months compared with their peers living in Europe. More than half of Ghanaians living in Ghana had experienced some stress at home or work whereas only a third of those living in Europe had experienced some stress at home or work. Permanent stress at home/work was fairly the same among Ghanaians living in SSA and Europe. Perceived discrimination was significantly higher among Ghanaians living in Europe compared with their peers living in Ghana. Depressive symptoms were more prevalent among Ghanaians living in Europe compared with their peers living in Ghana. Almost all Ghanaians living in Europe were first generation migrants. Ghanaians in Europe were more obese, more likely to smoke and less physically active compared with their peers living in Ghana. Prevalence of hypercholesterolemia was higher, but type 2 diabetes and hypertension were lower among Ghanaians living in Ghana compared with their peers living in Europe. Prevalence of albuminuria, reduced eGRF and CKD risk were higher in Ghanaians living in Ghana compared with those living in Europe.

Table 1: Baseline characteristics of respondents

	Ghanaians (SSA) n (%)	Ghanaians (Europe) n (%)	p-value
**N**	2,492 (44.1)	3,167 (55.9)	

For peer review only

			0.001*
**Female sex**	1672 (67.1)	1,851 (58.5)	0.001*
**Age (years)**	45.7±11.9	46.6±9.9	0.006*
**Educational status**			
Low	1169 (49.2)	635 (21.8)	0.001*
Middle	858 (36.1)	1111 (38.1)	0.122
High	347 (14.6)	1168 (40.1)	0.001*
**Negative life events in the past 12 months**			
No	739 (31.3)	1158 (41.0)	0.001*
Yes	1619 (68.7)	1667 (59.0)	0.001*
**Perceived stress at home/work**			
Never	692 (29.4)	1371 (48.8)	0.001*
Some periods	1290 (54.7)	1033 (36.8)	0.001*
Several/Permanent	375 (15.9)	407 (14.4)	0.117
**Perceived discrimination**			
**No**	2065 (95.2)	1960 (70.3)	0.001*
**Yes**	104 (4.8)	829 (29.7)	0.001*
**Depressive symptoms**			
**No**	2239 (94.9)	2582 (92.5)	0.001*
**Yes**	119 (5.1)	209 (7.5)	0.001*
**Migration generation**			
First	Not applicable	2868 (98.7)	Not applicable
Second	Not applicable	38 (1.3)	Not applicable

For peer review only

For peer review only - http://bmjopen.bmj.com/site/about/guidelines.xhtml

**BMI**			
Normal (<25kg/m2)	1373 (55.2)	643 (20.4)	0.001*
Overweight (25 ≤ 30kg/m2)	684 (27.5)	1,350 (42.8)	0.001*
Obese (>30kg/m2)	432 (17.3)	1163 (36.8)	0.001*
Currently smoking	36 (1.5)	121 (4.1)	0.001*
**Physical activity**	1255 (52.8)	1131 (44.0)	0.001*
**Hypocholesteraemia**	352 (14.2)	354 (11.3)	0.007*
**Type 2 diabetes**	206 (8.3)	444 (14.0)	0.001*
**Hypertension**	837 (33.6)	1801 (56.9)	0.001*
**ACR**			
A1 < 3mg/mmol	2215 (90.2)	2814 (91.8)	0.001*
A2-A ≥ 3mg/mmol	243 (9.8)	252 (8.2)	0.285
**eGFR**			
G1-G2 ≥ 60 mL/min/1.73 m2	2377 (96.3)	2936 (97.4)	0.018*
G3a-G5 < 60 mL/min/1.73 m2	85 (3.7)	78 (2.6)	0.018*
**CKD Risk**			
Low risk	2197 (89.6)	2705 (91.5)	0.015*
Moderate-very high risk	256 (10.4)	252 (8.5)	0.015*

Abbreviations: N, number of respondents; BMI, body mass index; eGFR, estimated glomerular filtration rate; G1-G2, normal to high kidney function to mildly decreased; G3a-G5, mildly to moderately decreased to Kidney failure; ACR, albumin creatinine ratio; A1, normal to mildly increased; A2-A3, moderately increased to severely increased; CKD, Chronic kidney disease; SSA, Sub-Saharan Africa; CI, confidence intervals.

For peer review only

For peer review only - http://bmjopen.bmj.com/site/about/guidelines.xhtml

Association between PS and CKD

Figure 1-4 shows CKD prevalence by negative life events in the past 12 months among Ghanaians living in Ghana and Europe. In Europe, CKD prevalence was fairly the same among those who had experienced any negative life events compared with those who had not in the last 12 months. Prevalence of CKD was higher among Ghanaians who had not experienced any negative life events in the past 12 months compared with those who had experienced some negative life events and living in Ghana. CKD prevalence was higher among Ghanaians who had not experienced any form of discrimination than those who had not in Ghana as well as in Europe (Figure 2). CKD prevalence was higher among Ghanaians who had experienced several/permanent stress at work/home in the past 12 months and living in Ghana or Europe (Figure 3). Ghanaians who did not report any form of depressive symptoms

For peer review only - http://bmjopen.bmj.com/site/about/guidelines.xhtml

had a higher CKD prevalence than those who did and living in Ghana and Europe (Figure 4).

For peer review only

For peer review only - http://bmjopen.bmj.com/site/about/guidelines.xhtml

Table 2 shows the correlation matrix between the 4 constructs of PS among Ghanaians living in Ghana and those living in Europe. All four constructs of PS were positively correlated with each other among Ghanaians living in Europe and Ghanaians living in Ghana, except stress at work/home and discrimination among Ghanaians living in Ghana.

Table 2: Relationship between PS constructs (negative life events, discrimination, stress at work or home and depression) by Ghanaians (SSA) and Ghanaians (Europe)

Correlation matrix	Negative events	Discrimination	Stress at work/home	Depression
**Europe**				
Negative events	1.000			
Discrimination	0.152**	1.000		
Stress at work/home	0.297**	0.161**	1.000	
Depressive symptoms	0.143**	0.1366**	0.285**	1.000
**Ghana**				
Negative events	1.000			
Discrimination	0.079**	1.000		

For peer review only

For peer review only - http://bmjopen.bmj.com/site/about/guidelines.xhtml

Stress at work/home	0.101**	-0.032	1.000	
Depressive symptoms	0.091**	0.042	0.185**	1.000

Significant at 1%, Spearman's correlation

For peer review only

Table 3 shows association between all 4 constructs of PS and CKD among Ghanaians living in Ghana and those living in Europe. There was no statistically significant association between PS and albuminuria, reduced eGFR and CKD risk among Ghanaians living in Ghana and those living in Europe except individuals living in Europe with some stress and lower risk of reduced eGFR. Table S1 shows further

For peer review only - http://bmjopen.bmj.com/site/about/guidelines.xhtml

adjustments for conventional risk factors of CKD. This did show any associations between PS and albuminuria, reduced eGFR and CKD risk among Ghanaians living in Ghana and Europe (S1). Table S2 shows further stratification based on obesity status. We did not find any association between PS and CKD for obese participants and those who were not obese for Ghanaians living in rural and urban Ghana and those living in Europe. However, we observed an inverse association between PS and CKD among migrants who were not obese but have experienced discrimination for the past 12 months (S2). In Table S3 we stratified our analysis based on diabetic status. We did not find any associations between PS and CKD for participants with and without diabetes for both Ghanaians living in rural and urban Ghana and their migrant peers in Europe (S3). Finally, Table S4 stratified analysis by hypertension status. No associations were observed between PS and CKD for individuals who had hypertension and those who did not have hypertension for both those living in rural and urban Ghana as well as their compatriots living in Europe. An inverse association was observed between PS and CKD among Ghanaians who have experienced discrimination in the last 12 months with no hypertension and living in Europe. Also, we observed that having experienced some stress at home/work was inversely associated with reduced eGFR among Ghanaians with hypertension and living in Europe (S4).

For peer review only - http://bmjopen.bmj.com/site/about/guidelines.xhtml
For peer review only

For peer review only

For peer review only - http://bmjopen.bmj.com/site/about/guidelines.xhtml

Table 3: Association of psychosocial stressors (PS) indicators (negative life events, discrimination, stress at work/home and depression) with albuminuria, reduced eGFR and CKD risk for Ghanaians living in Ghana and those living in Europe

For peer review only

	Albuminuria (ACR ≥ 3 mg/mmol)			eGFR < 60 mL/min/1.73 m2			High to very high CKD risk (KDIGO, 2012)		
		OR (95% CI)			OR (95% CI)			OR (95% CI)	
	n (%)	Model 1	Model 2	n (%)	Model 1	Model 2	n cases (%)	Model 1	Model 2
**Negative events**									
**Europe**									
No	1128 (8.2)	1.00 (Reference)	1.00 (Reference)	1106 (2.6)	1.00 (Reference)	1.0((Reference	1090 (8.5)	1.00 (Reference)	1.00 (Reference)
Yes	1615 (8.4)	1.03 (0.78-1.35)	1.07 (0.80-1.42)	1587 (2.5)	0.86 (0.53-1.42)	0.83 (0.49-1.39)	1557 (8.6)	0.97 (0.76-1.32)	0.99 (0.74-1.32)
**Ghana**									
No	732 (8.7)	1.00 (Reference)	1.00 (Reference)	736 (4.5)	1.00 (Reference)	1.00 (Reference)	732 (10.9)	1.00 (Reference)	1.00 (Reference)
Yes	1595 (3.8)	0.87 (0.65-1.16)	0.85 (0.63-1.14)	1601 (3.4)	0.69 (0.45-1.08)	0.67 (0.44-1.09)	1590 (9.9)	0.88 (0.66-1.17)	0.86 (0.64-1.15)
**Discrimination**									
**Europe**									
No	1899 (8.5)	1.00 (Reference)	1.00 (Reference)	1867 (2.6)	1.00 (Reference)	1.00 (Reference)	1832 (8.9)	1.00 (Reference)	1.00 (Reference)

For peer review only - http://bmjopen.bmj.com/site/about/guidelines.xhtml

Yes	810 (7.4)	0.87 (0.64-1.19)	0.92 (0.67-1.26)	791 (2.2)	0.83 (0.47-1.47)	0.84 (0.46-1.52)	782 (7.3)	0.82 (0.59-1.12)	0.84 (0.60-1.16)
**Ghana**									
No	2034 (10.0)	1.00 (Reference)	1.00 (Reference)	2047 (3.9)	1.00 (Reference)	1.00 (Reference)	2031 (10.6)	1.00 (Reference)	1.00 (Reference)
Yes	104 (7.7)	0.83 (0.39-1.73)	0.91 (0.67-1.24)	104 (1.9)	0.67 (0.15-2.85)	0.67 (0.16-2.84)	104 (6.7)	0.70 (0.32-1.55)	0.71 (0.32-1.55)
**Stress at home/work**									
**Europe**									
Never	1330 (8.2)	1.00 (Reference)	1.00 (Reference)	1305 (3.4)	1.00 (Reference)	1.00 (Reference)	1282 (8.5)	1.00 (Reference)	1.00 (Reference)
Some stress	1002 (7.9)	0.97 (0.72-1.31)	1.04 (0.76-1.42)	984 (1.4)	**0.47 (0.26-0.87)**	**0.46 (0.24-0.88)**	968 (8.6)	0.96 (0.71-1.30)	1.02 (0.74-1.39)
Several/Permanent stresses	397 (9.1)	1.11 (0.74-1.64)	1.153 (0.77-1.72)	390 (2.3)	0.73 (0.35-1.52)	0.76 (0.36-1.61)	383 (9.7)	1.13 (0.77-1.68)	1.19 (0.80-1.79)
**Ghana**									
Never	682 (10.3)	1.00 (Reference)	1.00 (Reference)	688 (3.3)	1.00 (Reference)	1.00 (Reference)	682 (9.9)	1.00 (Reference)	1.00 (Reference)
Some stress	1279 (9.5)	0.87 (0.64-1.19)	0.80 (0.59-1.11)	1279 (3.9)	1.06 (0.63-1.77)	1.11 (0.66-1.87)	1274 (10.3)	0.95 (0.69-1.30)	0.92 (0.67-1.26)
Several/Permanent stresses	365 (8.5)	0.75 (0.48-1.18)	0.68 (0.59-1.11)	369 (4.1)	1.13 (0.57-2.23)	1.22 (0.61-2.46)	365 (10.4)	0.96 (0.63-1.47)	0.92 (0.59-1.42)
**Depressive symptoms**									
**Europe**									
No	2505	1.00	1.00	2457	1.00	1.00	2416	1.00	1.00

For peer review only - http://bmjopen.bmj.com/site/about/guidelines.xhtml

	(8.5)	(Reference)	(Reference)	(2.7)	(Reference)	(Reference)	(8.7)	(Reference)	(Reference)
Yes	206 (6.3)	0.71 (0.39-1.27)	0.76 (0.43-1.36)	202 (1.5)	0.63 (0.19-2.03)	0.68 (0.21-2.23)	199 (7.1)	0.78 (0.44-1.37)	0.83 (0.47-1.46)
**Ghana**									
No	2212 (9.9)	1.00 (Reference)	1.00 (Reference)	2222 (3.8)	1.00 (Reference)	1.00 (Reference)	2207 (10.4)	1.00 (Reference)	1.00 (Reference)
Yes	114 (5.3)	0.45 (0.19-1.03)	0.45 (0.19-1.01)	114 (2.6)	0.52 (0.16-1.72)	0.53 (0.17-1.74)	114 (7.9)	0.62 (0.30-1.25)	0.61 (0.30-1.24)

Model 1, adjusted for age and sex; Model 2, adjusted for age, sex, and educational level for Ghanaians (SSA) and length of stay for those in Europe; Abbreviations: CI, confidence interval; ACR, albumin creatinine ration; eGFR, estimated glomerular filtration rate; CKD, chronic kidney disease; OR, odds ratio, n= total number of Ghanaians living in Ghana and Europe among the various levels of PS constructs; %, proportion of individuals with CKD among the various levels of PS constructs in Ghana and Europe

For peer review only

Table 4 shows associations between all 4 constructs of PS and CKD stratified by Ghanaians living in urban and rural Ghana. There was no association between PS and albuminuria, reduced eGFR and CKD risk among Ghanaians living rural and urban Ghana.

For peer review only

For peer review only - http://bmjopen.bmj.com/site/about/guidelines.xhtml

Table 4: **Association of psychosocial stressors (PS) indicators (negative life events, discrimination, stress at work/home and depressive symptoms) with albuminuria, reduced eGFR and CKD risk among rural and urban Ghana**

	Albuminuria (ACR ≥ 3 mg/mmol)			eGFR < 60 mL/min/1.73 m2			High to very high CKD risk (KDIGO, 2012)		
		OR (95% CI)			OR (95% CI)			OR (95% CI)	
	n (%)	Model 1	Model 2	n (%)	Model 1	Model 2	n cases (%)	Model 1	Model 2
**Negative events**									
**Urban Ghana**									
No	477 (11.9)	1.00 (Reference)	1.00 (Reference)	477 (4.4)	1.00 (Reference)	1.0((Reference	477 (12.2)	1.00 (Reference)	1.00 (Reference)
Yes	912 (10.5)	0.87 (0.61-1.23)	0.87 (0.61-1.24)	911 (3.4)	0.73 (0.41-1.31)	0.72 (0.40-1.29)	910 (10.8)	0.87 (0.61-1.24)	0.87 (0.61-1.25)
**Rural Ghana**									
No	255 (7.5)	1.00 (Reference)	1.00 (Reference)	259 (4.6)	1.00 (Reference)	1.00 (Reference)	255 (8.6)	1.00 (Reference)	1.00 (Reference)
Yes	683 (7.6)	0.97 (0.56-1.69)	0.94 (0.54-1.64)	690 (3.5)	0.63 (0.31-1.31)	0.66 (0.32-1.37)	680 (8.8)	0.93 (0.55-1.58)	0.92 (0.54-1.56)
**Discrimination**									
**Urban Ghana**									
No	1326 (11.1)	1.00 (Reference)	1.00 (Reference)	1326 (3.9)	1.00 (Reference)	1.00 (Reference)	1325 (11.4)	1.00 (Reference)	1.00 (Reference)

For peer review only

Yes	71 (8.5)	0.85 (0.36-2.00)	0.89 (0.37-2.11)	71 (2.8)	1.17 (0.27-2.09)	1.16 (0.27-2.06)	71 (7.1)	0.69 (0.27-1.77)	0.72 (0.28-1.83)
**Rural Ghana**									
No	708 (8.1)	1.00 (Reference)	1.00 (Reference)	721 (3.9)	1.00 (Reference)	1.00 (Reference)	706 (9.2)	1.00 (Reference)	1.00 (Reference)
Yes	33 (6.1)	0.79 (0.18-3.47)	0.84 (0.19-2.65)	33 (0.0)	***	***	33 (6.1)	0.75 (0.17-2.89)	0.83 (0.19-2.65)
**Stress at home/work**									
**Urban Ghana**									
Never	460 (10.9)	1.00 (Reference)	1.00 (Reference)	460 (3.3)	1.00 (Reference)	1.00 (Reference)	460 (10.2)	1.00 (Reference)	1.00 (Reference)
Some stress	732 (11.5)	1.04 (0.71-1.51)	0.91 (0.62-1.37)	730 (4.1)	1.27 (0.66-2.43)	1.30 (0.67-2.51)	730 (11.8)	1.13 (0.77-1.65)	1.04 (0.71-1.53)
Several/Permanent stresses	197 (9.6)	0.87 (0.50-1.52)	0.74 (0.42-1.02)	198 (3.5)	1.17 (0.46-2.84)	1.20 (0.47-3.09)	197 (11.7)	1.15 (0.68-1.71)	1.05 (0.61-1.81)
**Rural Ghana**									
Never	222 (9.0)	1.00 (Reference)	1.00 (Reference)	228 (3.5)	1.00 (Reference)	1.00 (Reference)	222 (9.5)	1.00 (Reference)	1.00 (Reference)
Some stress	547 (6.9)	0.69 (0.39-1.23)	0.68 (0.38-1.22)	549 (3.6)	0.88 (0.38-2.07)	0.92 (0.39-2.18)	544 (8.3)	0.74 (0.42-1.30)	0.75 (0.42-1.31)
Several/Permanent stresses	168 (7.1)	0.63 (0.30-1.37)	0.60 (0.28-1.29)	171 (4.7)	1.07(0.38-3.03)	1.21(0.43-3.46)	168 (8.9)	0.71 (0.34-1.50)	0.73 (0.35-1.50)
**Depressive symptoms**									
**Urban Ghana**									
No	1336	1.00	1.00	1335	1.00	1.00	1334	1.00	1.00

For peer review only - http://bmjopen.bmj.com/site/about/guidelines.xhtml

	(11.3)	(Reference)	(Reference)	(3.8)	(Reference)	(Reference)	(11.5)	(Reference)	(Reference)
Yes	52 (3.9)	0.30 (0.07-1.25)	0.30 (0.07-1.27)	52 (1.9)	0.46 (0.06-2.50)	0.45 (0.06-2.13)	52 (5.8)	0.44 (0.14-1.45)	0.45 (0.14-1.48)
**Rural Ghana**									
No	876 (7.7)	1.00 (Reference)	1.00 (Reference)	887 (3.8)	1.00 (Reference)	1.00 (Reference)	873 (8.7)	1.00 (Reference)	1.00 (Reference)
Yes	62 (6.5)	0.67 (0.23-1.94)	0.67 (0.23-1.94)	62 (3.2)	0.58 (0.13-2.56)	0.61 (0.14-2.68)	62 (9.7)	0.82 (0.33-2.01)	0.85 (0.34-2.09)

Model 1, adjusted for age and sex; Model 2, adjusted for age, sex, and educational level; Abbreviations: CI, confidence interval; ACR, albumin creatinine ration; eGFR, estimated glomerular filtration rate; CKD, chronic kidney disease; OR, odds ratio, n= total number of Ghanaians living in urban and rural Ghana among the various levels of PS constructs; %, proportion of individuals with CKD among the various levels of PS constructs in rural and urban Ghana. ***; no case of CKD and therefore odds ratios were not calculated

For peer review only

For peer review only

For peer review only - http://bmjopen.bmj.com/site/about/guidelines.xhtml

Table 5 shows associations between all 4 constructs of PS and CKD stratified by Ghanaians living in Amsterdam, Berlin and London. There was no association between PS and albuminuria, reduced eGFR and CKD risk among Ghanaians living Amsterdam, Berlin and London except for positive association between stress at work/home and albuminuria and CKD risk among Ghanaians living in Berlin.

For peer review only

For peer review only - http://bmjopen.bmj.com/site/about/guidelines.xhtml

Table 5: Association of psychosocial stressors (PS) indicators (negative life events, discrimination, stress at work/home and depressive symptoms) with albuminuria, reduced eGFR and CKD risk among Ghanaians in three European cities.

	Albuminuria (ACR ≥ 3 mg/mmol)			eGFR < 60 mL/min/1.73 m2			High to very high CKD risk (KDIGO, 2012)		
		OR (95% CI)			OR (95% CI)			OR (95% CI)	
	n (%)	Model 1	Model 2	n (%)	Model 1	Model 2	n (%)	Model 1	Model 2
**Negative events**									
**Amsterdam**									
No	548 (7.3)	1.00 (Reference)	1.00 (Reference)	534 (2.4)	1.00 (Reference)	1.00 (Reference))	521 (7.5)	1.00 (Reference)	1.00 (Reference)
Yes	784 (7.8)	1.08 (0.71-1.63)	1.18 (0.77-1.81)	764 (2.9)	1.11 (0.55-2.23)	1.15 (0.55-2.37)	742 (8.0)	1.06 (0.69-1.62)	1.08 (0.71-1.66)
**Berlin**									
No	213 (9.9)	1.00 (Reference)	1.00 (Reference)	213 (2.4)	1.00 (Reference)	1.00 (Reference)	213 (10.8)	1.00 (Reference)	1.00 (Reference)
Yes	329 (10.9)	1.12 (0.63-1.99)	1.19 (0.67-2.15)	330 (1.8)	0.64 (0.19-2.17)	0.61 (0.18-2.11)	329 (9.4)	0.86 (0.48-1.52)	0.91 (0.51-1.63)
**London**									
No	367 (8.7)	1.00 (Reference)	1.00 (Reference)	359 (3.1)	1.00 (Reference)	1.00 (Reference)	356 (8.7)	1.00 (Reference)	1.00 (Reference)
Yes	502 (7.8)	0.89 (0.55-	0.83 (0.49-	493	0.68 (0.28-	0.58 (0.22-	486 (9.1)	1.04 (0.64-	0.99 (0.58-1.68)

For peer review only - http://bmjopen.bmj.com/site/about/guidelines.xhtml

		1.46)	1.41)	(2.2)	1.65)	1.51)		1.68)	
**Discrimination**									
**Amsterdam**									
No	956 (8.3)	1.00 (Reference)	1.00 (Reference)	935 (2.9)	1.00 (Reference)	1.00 (Reference)	909 (8.5)	1.00 (Reference)	1.00 (Reference)
Yes	363 (5.0)	0.59 (0.34-1.00)	0.59 (0.35-1.02)	349 (2.1)	0.69 (0.30-1.62)	0.81 (0.34-1.91)	342 (5.9)	0.69 (0.41-1.14)	0.69 (0.41-1.16)
**Berlin**									
No	329(10.0)	1.00 (Reference)	1.00 (Reference)	329 (2.1)	1.00 (Reference)	1.00 (Reference)	329 (10.3)	1.00 (Reference)	1.00 (Reference)
Yes	209 (11.0)	1.11 (0.63-1.95)	1.16 (0.65-2.05)	210 (1.9)	0.83 (0.24-2.93)	0.82 (0.23-2.91)	209 (9.1)	0.86 (0.48-1.56)	0.89 (0.49-1.63)
**London**									
No	614 (7.9)	1.00 (Reference)	1.00 (Reference)	603 (2.5)	1.00 (Reference)	1.00 (Reference)	594 (8.9)	1.00 (Reference)	1.00 (Reference)
Yes	238 (7.9)	1.03 (0.59-1.81)	1.18 (0.65-2.15)	232 (2.6)	1.29 (0.46-3.59)	1.09 (0.35-3.43)	231 (7.8)	0.93 (0.53-1.63)	0.98 (0.52-1.82)
**Stress at home/work**									
**Amsterdam**									
Never	634 (8.4)	1.00 (Reference)	1.00 (Reference)	622 (3.2)	1.00 (Reference)	1.00 (Reference)	603 (8.0)	1.00 (Reference)	1.00 (Reference)
Some stress	478 (5.7)	0.68 (0.42-1.11)	0.69 (0.42-1.13)	462 (1.9)	0.64 (0.29-1.43)	0.68 (0.30-1.52)	452 (6.0)	0.74 (0.45-1.20)	0.74 (0.45-1.22)
Several/Permanent stresses	210 (9.1)	1.09 (0.63-1.91)	1.12 (0.64-1.95)	204 (2.5)	0.71 (0.26-1.95)	0.77 (0.28-2.14)	198 (10.1)	1.24 (0.71-2.14)	1.26 (0.73-2.20)

For peer review only - http://bmjopen.bmj.com/site/about/guidelines.xhtml

**Berlin**									
Never	250 (9.0)	1.00 (Reference)	1.00 (Reference)	250 (2.0)	1.00 (Reference)	1.00 (Reference)	250 (6.4)	1.00 (Reference)	1.00 (Reference)
Some stress	196 (15.3)	**2.50 (1.33-4.71)**	**2.81 (1.46-5.40)**	197 (1.5)	0.88 (0.20-3.79)	0.83 (0.19-3.62)	196 (14.8)	**2.57 (1.34-4.90)**	**2.78 (1.43-5.43)**
Several/Permanent stresses	96 (10.4)	1.64 (0.72-3.73)	1.69 (0.73-3.91)	197 (3.1)	2.10 (0.47-9.46)	2.04 (0.44-9.26)	76 (9.4)	1.52 (0.65-3.58)	1.58 (0.66-3.75)
**London**									
Never	446 (9.2)	1.00 (Reference)	1.00 (Reference)	433 (4.4)	1.00 (Reference)	1.00 (Reference)	429 (10.5)	1.00 (Reference)	1.00 (Reference)
Some stress	328 (7.0)	0.73 (0.43-1.25)	0.79 (0.44-1.40)	325 (0.6)	0.17 (0.04-0.73)	0.09 (0.01-0.67)	320 (6.9)	0.65 (0.38-1.10)	0.66 (0.37-1.19)
Several/permanent stresses	91 (7.7)	0.81 (0.35-1.87)	0.86 (0.35-2.14)	90 (1.1)	0.27 (0.03-2.12)	0.24 (0.03-2.05)	74 (8.9)	0.83 (0.38-1.83)	0.92 (0.39-2.16)
**Depressive symptoms**									
**Amsterdam**									
No	1199 (7.8)	1.00 (Reference)	1.00 (Reference)	1135 (2.8)	1.00 (Reference)	1.00 (Reference)	1135 (7.9)	1.00 (Reference)	1.00 (Reference)
Yes	121 (6.6)	0.81 (0.39-1.72)	0.83 (0.39-1.76)	118 (1.7)	0.65 (0.15-2.77)	0.71 (0.16-3.06)	116 (6.9)	0.83 (0.39-1.76)	0.82 (0.38-1.74)
**Berlin**									
No	503 (10.7)	1.00 (Reference)	1.00 (Reference)	504 (2.2)	1.00 (Reference)	1.00 (Reference)	503 (10.1)	1.00 (Reference)	1.00 (Reference)

For peer review only

Yes	34 (5.9)	0.53 (0.12-2.27)	0.49 (90.11-2.13)	34 (0.0)	***	***	34 (5.9)	0.55 (0.13-2.37)	0.52 (0.12-2.24)
**London**									
No	803 (8.3)	1.00 (Reference)	1.00 (Reference)	785 (2.6)	1.00 (Reference)	1.00 (Reference)	778 (8.9)	1.00 (Reference)	1.00 (Reference)
Yes	51 (5.9)	0.67 (0.20-2.21)	0.91 (0.27-3.07)	50 (2.0)	0.91 (0.11-7.43)	1.15 (0.14-9.54)	49 (8.2)	0.94 (0.33-2.69)	1.30 (0.44-3.81)

Model 1, adjusted for age and sex; Model 2, adjusted for age, sex, educational level and length of stay; Abbreviations: CI, confidence interval; ACR, albumin creatinine ration; eGFR, estimated glomerular filtration rate; CKD, chronic kidney disease; OR, odds ratio, n= total number of Ghanaians living in Amsterdam, Berlin and London among the various levels of PS constructs; %, proportion of individuals with CKD among the various levels of PS constructs in Europe ***; no case of CKD and therefore odds ratios were not calculated.

For peer review only

For peer review only - http://bmjopen.bmj.com/site/about/guidelines.xhtml

Discussion

Key findings

Whereas there was an association between those who have experienced some stress at home/work and reduced eGFR among Ghanaians living in Europe, we did not find any association between PS and CKD among Ghanaians living in rural and urban Ghana and their peers in Europe. Also, PS was not associated with CKD for those living in rural and urban Ghana and neither for those living in the three European cities. However, there was an association between stress at work/home and albuminuria and CKD risk among Ghanaians living in Berlin. Further adjustment for conventional risk factors of CKD yielded similar results.

Discussion of key findings

Association between PS and CKD in Ghana

Our study did not find any association between any of the four constructs of PS and prevalence of CKD (albuminuria, reduced eGFR and CKD risk) among Ghanaians living in rural and urban Ghana. Our findings are however in contrast with earlier studies which reported positive associations between PS and prevalent of CKD (22, 26, 48). Other studies have hypothesised that the influence of PS on CKD may be important in only those with hypertension and diabetes and that PS may mediate or moderate the association between renal functioning and lifestyle behaviours such as smoking and physical activity (28). For example, they argue that stress enhances

For peer review only - http://bmjopen.bmj.com/site/about/guidelines.xhtml

Sympathetic Nervous System (SNS) activity to increase glucocorticoid secretion and inflammatory cytokines, which heavily contribute to hypertension, diabetes and vascular disease which are major risk factors of CKD incidence and prevalence (49). We did not find any literature on the association between PS and CKD prevalence in rural and urban populations. Worth noting, however, is the presence of rich family support systems in the Ghanaian context especially in rural Ghana which may help individuals with CKD to cope with PS thereby minimizing its effect. For example, patients with limited social networks and low social support have been shown to have augmented risk of morbidity and mortality (50-52). Specifically, there is evidence that positive social support is a protective factor for persons dealing with long-term disease conditions (53). Other studies have reported a protective relationship between social networks, emotionally supportive relationship and threats to physiological and psychosocial health (54).

Association between PS and CKD Amsterdam, Berlin and London

Literature on the association between PS and CKD prevalence among migrants is scant and absent in most European populations. The lack of positive association between PS and CKD in our study is consistent with recent studies conducted among African Americans (26, 28) and other populations (27, 55). Specifically, a recent study using data from the Jackson Heart Study which comprised of extensive constructs of psychosocial variables reported that greater life stressors were associated with lower prevalence of CKD at baseline (26). Several studies in other parts of the world have

For peer review only - http://bmjopen.bmj.com/site/about/guidelines.xhtml

reported a positive relationship between higher prevalence of stressors and CKD risk (21, 22), although study findings have been inconsistent. Whereas some did not find any associations among African Americans (26) others found associations in other populations. Even among those who found some associations the directions differed (22). Reasons for the lack of association observed in our study among migrants are not fully understood but this lack of association may be a reflection of the real world. First, migrants from Ghana practice both nuclear and extended family support system as their peers living in rural and urban, this practice may mitigate the impact of stressors such as unemployment, death of a love one, discrimination, etc. They also belong to several religious organisations such as churches, which provide similar support systems against stressors. Moreover, there are several associations of the various ethnic groups (Akan, Ga and Ewe) providing such support when the need arises. These systems provide both instrumental and/or emotional social support (56). These assertions are supported by several studies. Specifically, these studies have shown that social support positively affect outcomes through mechanisms such as increased patient compliance with therapies, decreased levels of depressive affect, direct physiologic effects on the immune system and improved perception of quality of life (53, 54).

Strength and limitation

Our study is the first to use all four robust constructs of PS to determine association between PS and CKD. This gave our study a more robust definition of PS compared to other similar studies. The use of all three definitions of CKD per KDIGO guidelines

For peer review only - http://bmjopen.bmj.com/site/about/guidelines.xhtml

also provided a broader definition of CKD and allowed comparison between different geographical regions. The use of a homogenous population of Ghanaians and standardized protocols and diagnostic criteria in this study also provided a novel opportunity to compare Ghanaians living in rural and urban Ghana and their compatriots living in Europe. There are limitations to our study. First, the use of cross-sectional design prevented us from determining the longitudinal effect of repeated exposure to PS among the two populations. PS is captured and experienced in different magnitude across different populations. We were unable to ascertain if PS as defined in this study was adequately captured among Ghanaians living in rural and urban Ghana.

Conclusion

Generally, our study shows no associations between stress as indicated by four PS indicators and prevalence of CKD. Consequently, there is the need to explore other factors that may be responsible for the observed differences in the prevalence of CKD among Ghanaians living in rural and urban Ghana and their peers living in Europe.

Acknowledgement

The authors are very grateful to the research assistants, interviewers and other staff of the five research locations who took part in gathering the data and the Ghanaian volunteers in all the participating RODAM sites. We gratefully acknowledge the advisory board members for their valuable support in shaping the RODAM study methods and the Academic Medical Centre Biobank for their support in biobank management and high-quality storage of collected samples.

For peer review only - http://bmjopen.bmj.com/site/about/guidelines.xhtml

Contributors

My co-authors have all contributed substantially to this manuscript and approve of this submission. Research idea and study design: DNA, CA, KS, DA, EB, KM, JA; data acquisition and curation: DNA, CA, EB, KM, data analysis/interpretation: DNA, CA, KS, DA, EB, KM, LS, JA, EOD, KKG, FPM, ID, JS, SB, ADA; statistical analysis: DNA, CA, KS. DNA, CA, KS, DA, EB, KM, LS, JA, EOD, KKG, FPM, ID, JS, SB, ADA contributed important intellectual content during manuscript drafting or revision and accepts accountability for the overall work by ensuring that questions pertaining to the accuracy or integrity of any portion of the work are appropriately investigated and resolved. DNA and CA takes responsibility that this study has been reported honestly, accurately, and transparently; that no important aspects of the study have been omitted; and that any discrepancies from the study as planned have been explained.

For peer review only

Funding

This work was supported by the European Commission under the Framework Programme (Grant Number: 278901). The funders had no role in study design, data collection and analysis, decision to publish, or preparation of the manuscript. The

For peer review only - http://bmjopen.bmj.com/site/about/guidelines.xhtml

Wellcome Trust supported Professor Smeeth's contribution, grant number WT082178. Professor Joachim Spranger was supported by the DZHK (German Center for cardiovascular research) and the Berlin Institute of Health (BIH).

Competing interest: I have communicated with all my co-authors and obtained their full disclosures. My co-authors and I declare no conflicts of interest.

Patient Consent: None declared

Ethics approval: IRBs at each participating site.

Data sharing statement: Data are available from the RODAM research cohort, a third party. Dr. Eric Beune affiliated with the RODAM research cohort and a co-author of this paper in accordance with the RODAM requirements for collaboration. Dr. Beune is the Data Collection Coordinator of RODAM and may be contacted with further questions (e.j.beune@amc.uva.nl). Additionally, researchers interested in further collaboration with RODAM may see the following URL: http://www.rod-am.eu/

For peer review only

For peer review only

References

1. Weisbord SD. Symptoms and their correlates in chronic kidney disease. Adv Chronic Kidney Dis. 2007;14(4):319-27.
2. Correa-Rotter R, Naicker S, Katz IJ, Agarwal SK, Valdes RH, Kaseje D, et al. Demographic and epidemiologic transition in the developing world: role of albuminuria in the early diagnosis and prevention of renal and cardiovascular disease. Kidney Int. 2004;66:S32-S7.
3. Lozano R, Naghavi M, Foreman K, Lim S, Shibuya K, Aboyans V, et al. Global and regional mortality from 235 causes of death for 20 age groups in 1990 and 2010: a systematic analysis for the Global Burden of Disease Study 2010. The lancet. 2012;380(9859):2095-128.

For peer review only - http://bmjopen.bmj.com/site/about/guidelines.xhtml

4. Katz IJ, Gerntholtz T, Naicker S. Africa and nephrology: the forgotten continent. Nephron Clinical Practice. 2011;117(4):320-7.
5. Bruce MA, Beech BM, Sims M, Brown TN, Wyatt SB, Taylor HA, et al. Social environmental stressors, psychological factors, and kidney disease. J Investig Med. 2009;57(4):583-9.
6. Osafo C, Mate-Kole M, Affram K, Adu D. Prevalence of chronic kidney disease in hypertensive patients in Ghana. Ren Fail. 2011;33(4):388-92.
7. Ojo A. Addressing the global burden of chronic kidney disease through clinical and translational research. Trans Am Clin Climatol Assoc. 2014;125:229.
8. Norris K, Nissenson AR. Race, gender, and socioeconomic disparities in CKD in the United States. J Am Soc Nephrol. 2008;19(7):1261-70.
9. Volkova N, McClellan W, Klein M, Flanders D, Kleinbaum D, Soucie JM, et al. Neighborhood poverty and racial differences in ESRD incidence. J Am Soc Nephrol. 2008;19(2):356-64.
10. Shoham DA, Vupputuri S, Roux AVD, Kaufman JS, Coresh J, Kshirsagar AV, et al. Kidney disease in life-course socioeconomic context: the Atherosclerosis Risk in Communities (ARIC) Study. Am J Kidney Dis. 2007;49(2):217-26.
11. Tarver-Carr ME, Powe NR, Eberhardt MS, LaVeist TA, Kington RS, Coresh J, et al. Excess risk of chronic kidney disease among African-American versus white subjects in the United States: a population-based study of potential explanatory factors. J Am Soc Nephrol. 2002;13(9):2363-70.
12. Adjei DN, Stronks K, Adu D, Beune E, Meeks K, Smeeth L, et al. Chronic kidney disease burden among African migrants in three European countries and in urban and rural Ghana: the RODAM cross-sectional study. Nephrology Dialysis Transplantation. 2018.
13. Adjei DN, Stronks K, Adu D, Snijder MB, Modesti PA, Peters RJ, et al. Relationship between educational and occupational levels, and Chronic Kidney Disease in a multi-ethnic sample-The HELIUS study. PLoS One. 2017;12(11):e0186460.
14. Fremont A, Bird CE. Social and psychological factors, physiological processes, and physical health. 2000.
15. Krantz DS, McCeney MK. Effects of psychological and social factors on organic disease: a critical assessment of research on coronary heart disease. Annu Rev Psychol. 2002;53(1):341-69.
16. Amerena J, Julius S. The role of the autonomic nervous system in hypertension. Hypertens Res. 1995;18(2):99-110.
17. Calhoun DA, Mutinga ML. Race, family history of hypertension, and sympathetic response to cold pressor testing. Blood Press. 1997;6(4):209-13.
18. Dibona GF. Neural control of the kidney: past, present, and future. Hypertension. 2003;41(3):621-4.
19. Auchincloss AH, Diez Roux AV, Brown DG, O'meara ES, Raghunathan TE. Association of insulin resistance with distance to wealthy areas: the multi-ethnic study of atherosclerosis. Am J Epidemiol. 2006;165(4):389-97.
20. Bruce MA, Sims M, Miller S, Elliott V, Ladipo M. One size fits all? Race, gender and body mass index among US adults. J Natl Med Assoc. 2007;99(10):1152.

For peer review only

For peer review only - http://bmjopen.bmj.com/site/about/guidelines.xhtml

21. Kim JW, Moon SJ, Kim HJ, Lee DG. Relationship between Chronic Kidney Disease and Depression in Elderly Koreans Using the 2013 Korea National Health and Nutrition Examination Survey Data. Korean journal of family medicine. 2017;38(3):156-62.
22. Loosman WL, Rottier MA, Honig A, Siegert CE. Association of depressive and anxiety symptoms with adverse events in Dutch chronic kidney disease patients: a prospective cohort study. BMC Nephrol. 2015;16(1):155.
23. Novak M, Mucsi I, Mendelssohn D. Screening for depression: only one piece of the puzzle. Nephrology Dialysis Transplantation. 2013;28(6):1336-40.
24. McKercher CM, Venn AJ, Blizzard L, Nelson MR, Palmer AJ, Ashby MA, et al. Psychosocial factors in adults with chronic kidney disease: characteristics of pilot participants in the Tasmanian Chronic Kidney Disease study. BMC Nephrol. 2013;14(1):83.
25. Cukor D, Fruchter Y, Ver Halen N, Naidoo S, Patel A, Saggi SJ. A preliminary investigation of depression and kidney functioning in patients with chronic kidney disease. Nephron Clinical practice. 2012;122(3-4):139-45.
26. Lunyera J, Davenport CA, Bhavsar NA, Sims M, Scialla J, Pendergast J, et al. Nondepressive Psychosocial Factors and CKD Outcomes in Black Americans. Clin J Am Soc Nephrol. 2018:CJN. 06430617.
27. Annor FB, Masyn KE, Okosun IS, Roblin DW, Goodman M. Psychosocial stress and changes in estimated glomerular filtration rate among adults with diabetes mellitus. Kidney research and clinical practice. 2015;34(3):146-53.
28. Gholson GK, Mwendwa DT, Wright RS, Callender CO, Campbell AL. The Combined Influence of Psychological Factors on Biomarkers of Renal Functioning in African Americans. Ethn Dis. 2015;25(2):117.
29. Kaur M, Kaur A, Saggu A, Kaur A, Kaur A, Arundeep DK, et al. Comparative study on psychosocial stresses among urban and rural geriatric population in selected areas of district Ludhiana (Punjab), 2006. Nursing and Midwifery Research. 2007;3(2).
30. Agyemang C, Beune E, Meeks K, Owusu-Dabo E, Agyei-Baffour P, Aikins Ad-G, et al. Rationale and cross-sectional study design of the Research on Obesity and type 2 Diabetes among African Migrants: the RODAM study. BMJ open. 2015;4(3):e004877.
31. Agyemang C, Snijder MB, Adjei DN, van den Born B-JH, Modesti PA, Peters RJ, et al. Ethnic Disparities in CKD in the Netherlands: The Healthy Life in an Urban Setting (HELIUS) Study. Am J Kidney Dis. 2016;67(3):391-9.
32. Peek ME, Nunez-Smith M, Drum M, Lewis TT. Adapting the everyday discrimination scale to medical settings: reliability and validity testing in a sample of African American patients. Ethn Dis. 2011;21(4):502.
33. Pérez DJ, Fortuna L, Alegria M. Prevalence and correlates of everyday discrimination among US Latinos. J Community Psychol. 2008;36(4):421-33.
34. Ikram UZ, Snijder MB, Fassaert TJ, Schene AH, Kunst AE, Stronks K. The contribution of perceived ethnic discrimination to the prevalence of depression. The European Journal of Public Health. 2014;25(2):243-8.
35. Rosengren A, Hawken S, Ôunpuu S, Sliwa K, Zubaid M, Almahmeed WA, et al. Association of psychosocial risk factors with risk of acute myocardial infarction in 11 119 cases and 13 648 controls from 52 countries (the INTERHEART study): case-control study. The Lancet. 2004;364(9438):953-62.

For peer review only - http://bmjopen.bmj.com/site/about/guidelines.xhtml

36. Rosmalen J, Bos E, De Jonge P. Validation of the Long-term Difficulties Inventory (LDI) and the List of Threatening Experiences (LTE) as measures of stress in epidemiological population-based cohort studies. Psychol Med. 2012;42(12):2599-608.
37. Brugha T, Bebbington P, Tennant C, Hurry J. The List of Threatening Experiences: a subset of 12 life event categories with considerable long-term contextual threat. Psychol Med. 1985;15(1):189-94.
38. Kroenke K, Spitzer RL, Williams JB. The phq-9. J Gen Intern Med. 2001;16(9):606-13.
39. Association AD. Standards of Medical Care in Diabetes—2014. Diabetes Care 2014; 37 (Suppl. 1): S14–S80Diagnosis and Classification of Diabetes Mellitus. Diabetes Care 2014; 37 (Suppl. 1): S81–S90. Diabetes Care. 2014;37(3):887-.
40. Stevens LA, Schmid CH, Greene T, Zhang YL, Beck GJ, Froissart M, et al. Comparative performance of the CKD Epidemiology Collaboration (CKD-EPI) and the Modification of Diet in Renal Disease (MDRD) Study equations for estimating GFR levels above 60 mL/min/1.73 m2. Am J Kidney Dis. 2010;56(3):486-95.
41. Kellum JA, Lameire N, Aspelin P, Barsoum RS, Burdmann EA, Goldstein SL, et al. Kidney disease: improving global outcomes (KDIGO) acute kidney injury work group. KDIGO clinical practice guideline for acute kidney injury. Kidney international supplements. 2012;2(1):1-138.
42. KDIGO G. Work Group. KDIGO clinical practice guideline for glomerulonephritis. Kidney inter, Suppl. 2012;2:139-274.
43. Rivera B, Casal B, Currais L. Length of stay and mental health of the immigrant population in Spain: Evidence of the healthy immigrant effect. Applied Economics. 2015;47(19):1972-82.
44. Collins AJ, Foley RN, Herzog C, Chavers B, Gilbertson D, Ishani A, et al. US Renal Data System 2010 Annual Data Report. American journal of kidney diseases: the official journal of the National Kidney Foundation. 2011;57(1 Suppl 1):A8, e1.
45. Fox CS, Larson MG, Leip EP, Culleton B, Wilson PW, Levy D. Predictors of new-onset kidney disease in a community-based population. JAMA. 2004;291(7):844-50.
46. Banks J, Marmot M, Oldfield Z, Smith JP. Disease and disadvantage in the United States and in England. JAMA. 2006;295(17):2037-45.
47. Levin A, Stevens L, McCullough PA. Cardiovascular disease and the kidney: Tracking a killer in chronic kidney disease. Postgrad Med. 2002;111(4):53-60.
48. Tsai Y-C, Chiu Y-W, Hung C-C, Hwang S-J, Tsai J-C, Wang S-L, et al. Association of symptoms of depression with progression of CKD. Am J Kidney Dis. 2012;60(1):54-61.
49. Bruce MA, Griffith DM, Thorpe RJ. Stress and the kidney. Adv Chronic Kidney Dis. 2015;22(1):46-53.
50. Brummett BH, Barefoot JC, Siegler IC, Clapp-Channing NE, Lytle BL, Bosworth HB, et al. Characteristics of socially isolated patients with coronary artery disease who are at elevated risk for mortality. Psychosom Med. 2001;63(2):267-72.
51. Eng PM, Rimm EB, Fitzmaurice G, Kawachi I. Social ties and change in social ties in relation to subsequent total and cause-specific mortality and coronary heart disease incidence in men. Am J Epidemiol. 2002;155(8):700-9.
52. Patel SS, Peterson RA, Kimmel PL, editors. Psychosocial factors in patients with chronic kidney disease: The impact of social support on end-stage renal disease. Seminars in dialysis; 2005: Wiley Online Library.

For peer review only

For peer review only - http://bmjopen.bmj.com/site/about/guidelines.xhtml

53. Cohen SD, Sharma T, Acquaviva K, Peterson RA, Patel SS, Kimmel PL. Social support and chronic kidney disease: an update. Adv Chronic Kidney Dis. 2007;14(4):335-44.
54. Uchino BN, Cacioppo JT, Kiecolt-Glaser JK. The relationship between social support and physiological processes: a review with emphasis on underlying mechanisms and implications for health. Psychol Bull. 1996;119(3):488.
55. Tsurugano S, Nakao M, Takeuchi T, Nomura K, Yano E. Job stress strengthens the link between metabolic risk factors and renal dysfunction in adult men. The Tohoku journal of experimental medicine. 2012;226(2):101-8.
56. Tróccol BT. Desenvolvimento de escala para avaliação do suporte social em HIV/aids. Psicologia: Teoria e Pesquisa. 2006;22(3):317-26.

For peer review only

Legend for figures

For peer review only - http://bmjopen.bmj.com/site/about/guidelines.xhtml

Figure 1: Prevalence of CKD risk among Ghanaians who have experienced negative life events and those who have not experienced any negative life events stratified by site. Definition per 2012 KDIGO (Kidney Disease: Improving Global Outcomes) guidelines.

Figure 2: Prevalence of CKD risk among Ghanaians who have experienced discrimination and those who have not experienced any discrimination stratified by site. Definitions per 2012 KDIGO (Kidney Disease: Improving Global Outcomes) guidelines.

Figure 3: Prevalence of CKD risk among Ghanaians who have experienced stress at home/work and those who have not experienced any stress at home/work in the past 12 months stratified by site. Definitions per 2012 KDIGO (Kidney Disease: Improving Global Outcomes) guidelines.

Figure 4: Prevalence of CKD risk among Ghanaians who have depressive symptoms and those who do not have depressive symptoms stratified by site. Definitions per 2012 KDIGO (Kidney Disease: Improving Global Outcomes) guidelines.

For peer review only

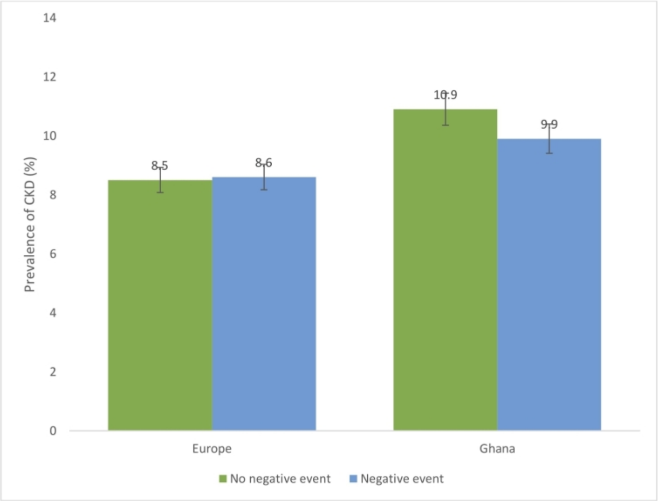

Figure 1: Prevalence of CKD risk among Ghanaians who have experienced negative life events and those who have not experienced any negative life events stratified by site. Definition per 2012 KDIGO (Kidney Disease: Improving Global Outcomes) guidelines.

Figure 1: Prevalence of CKD risk among Ghanaians who have experienced negative life events and those who have not experienced any negative life events stratified by site. Definition per 2012 KDIGO (Kidney Disease: Improving Global Outcomes) guidelines.

215x279mm (300 x 300 DPI)

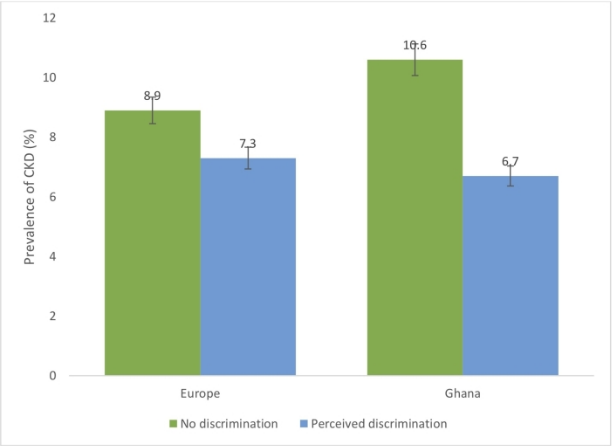

Figure 2: Prevalence of CKD risk among Ghanaians who have experienced discrimination and those who have not experienced any discrimination stratified by site. Definitions per 2012 KDIGO (Kidney Disease: Improving Global Outcomes) guidelines.

Figure 2: Prevalence of CKD risk among Ghanaians who have experienced discrimination and those who have not experienced any discrimination stratified by site. Definitions per 2012 KDIGO (Kidney Disease: Improving Global Outcomes) guidelines.

215x279mm (300 x 300 DPI)

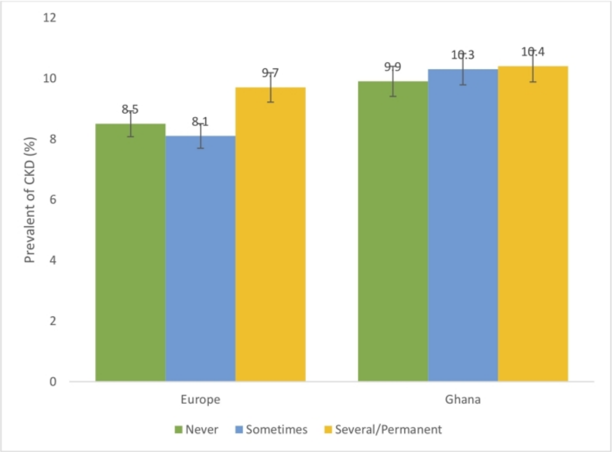

Figure 3: Prevalence of CKD risk among Ghanaians who have experienced stress at home/work and those who have not experienced any stress at home/work in the past 12 months stratified by site. Definitions per 2012 KDIGO (Kidney Disease: Improving Global Outcomes) guidelines.

Figure 3: Prevalence of CKD risk among Ghanaians who have experienced stress at home/work and those who have not experienced any stress at home/work in the past 12 months stratified by site. Definitions per 2012 KDIGO (Kidney Disease: Improving Global Outcomes) guidelines.

215x279mm (300 x 300 DPI)

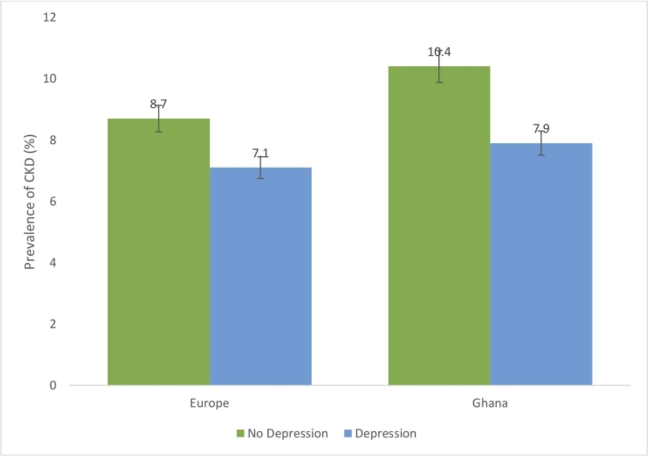

Figure 4: Prevalence of CKD risk among Ghanaians who have depressive symptoms and those who do not have depressive symptoms stratified by site. Definitions per 2012 KDIGO (Kidney Disease: Improving Global Outcomes) guidelines.

Figure 4: Prevalence of CKD risk among Ghanaians who have depressive symptoms and those who do not have depressive symptoms stratified by site. Definitions per 2012 KDIGO (Kidney Disease: Improving Global Outcomes) guidelines

215x279mm (300 x 300 DPI)

Supplementary Tables

S1: Association of psychosocial stressors (PS) indicators (negative life events, discrimination, stress at work/home and depression) with albuminuria, reduced eGFR and CKD risk for Ghanaians living in Ghana and those living in Europe

	Albuminuria (ACR ≥ 3 mg/mmol)		eGFR < 60 mL/min/1.73 m2		High to very high CKD risk (KDIGO, 2012)	
		OR (95% CI)		OR (95% CI)		OR (95% CI)
	n (%)	Model 3	n (%)	Model 3	n cases (%)	Model 3
**Negative events**						
**Europe**						
No	1128 (8.2)	1.00 (Reference)	1106 (2.6)	1.00 (Reference)	1090 (8.5)	1.00 (Reference)
Yes	1615 (8.4)	0.95 (0.70-1.30)	1587 (2.5)	0.77 (0.44-1.37)	1557 (8.6)	0.92 (0.67-1.26)
**Ghana**						
No	732 (10.4)	1.00 (Reference)	736 (4.5)	1.00 (Reference)	732 (10.9)	1.00 (Reference)
Yes	1595 (9.2)	0.79 (0.58-1.07)	1601 (3.4)	0.69 (0.43-1.09)	1590 (9.9)	0.79 (0.58-1.06)
**Discrimination**						
**Europe**						
No	1899 (8.5)	1.00 (Reference)	1867 (2.6)	1.00 (Reference)	1832 (8.9)	1.00 (Reference)
Yes	810 (7.4)	0.87 (0.61-1.21)	791 (2.2)	0.87 (0.45-1.65)	782 (7.3)	0.79 (0.55-1.12)
**Ghana**						
No	2034 (10.0)	1.00 (Reference)	2047 (3.9)	1.00 (Reference)	2031 (10.6)	1.00 (Reference)
Yes	104 (7.7)	0.92 (0.43-1.98)	104 (1.9)	0.65 (0.15-2.79)	104 (6.7)	0.74 (0.33-1.66)
**Stress at home/work**						
**Europe**						
Never	1330 (8.2)	1.00 (Reference)	1305 (3.4)	1.00 (Reference)	1282 (8.5)	1.00 (Reference)
Some stress	1002 (7.9)	1.01 (0.72-1.41)	984 (1.4)	0.51 (0.25-1.04)	968 (8.1)	1.01 (0.72-1.42)
Several/Permanent stresses	397 (9.1)	1.00 (0.64-1.59)	390 (2.3)	0.91 (0.40-2.03)	383 (9.7)	1.15 (0.74-1.79)

For peer review only - http://bmjopen.bmj.com/site/about/guidelines.xhtml

**Ghana**						
Never	682 (10.3)	1.00 (Reference)	688 (3.3)	1.00 (Reference)	682 (9.9)	1.00 (Reference)
Some stress	1279 (9.5)	0.78 (0.56-1.09)	1279 (3.9)	1.14 (0.67-1.93)	1274 (10.3)	0.93 (0.67-1.29)
Several/Permanent stresses	365 (8.5)	0.70 (0.43-1.13)	369 (4.1)	1.32 (0.65-1.68)	365 (10.4)	0.97 (0.62-1.52)
**Depressive symptoms**						
**Europe**						
No	2505 (8.5)	1.00 (Reference)	2457 (2.7)	1.00 (Reference)	2416 (8.7)	1.00 (Reference)
Yes	206 (6.3)	0.65 (0.33-1.28)	202 (1.5)	0.94 (0.28-3.14)	199 (7.1)	0.78 (0.41-1.47)
**Ghana**						
No	2212 (9.9)	1.00 (Reference)	2222 (3.8)	1.00 (Reference)	2207 (10.4)	1.00 (Reference)
Yes	114 (5.3)	0.41 (0.16-1.04)	114 (2.6)	0.58 (0.17-1.95)	114 (7.9)	0.62 (0.28-1.31)

Model 3, adjusted for age, sex educational level, hypertension, diabetes, hypercholesterolemia, BMI, physical activity and smoking; Abbreviations: CI, confidence interval; ACR, albumin creatinine ration; eGFR, estimated glomerular filtration rate; CKD, chronic kidney disease; OR, odds ratio, n= total number of Ghanaians living in Ghana and Europe among the various levels of PS constructs; %, proportion of individuals with CKD among the various levels of PS constructs in Ghana and Europe.

For peer review only - http://bmjopen.bmj.com/site/about/guidelines.xhtml

S2: Association of psychosocial stressors (PS) indicators (negative life events, discrimination, stress at work/home and depression) with albuminuria, reduced eGFR and CKD risk for Ghanaians living in Ghana and those living in Europe stratified by obesity status

	Albuminuria (ACR ≥ 3 mg/mmol)			eGFR < 60 mL/min/1.73 m2			High to very high CKD risk (KDIGO, 2012)		
		OR (95% CI)			OR (95% CI)			OR (95% CI)	
	n (%)	Model 1	Model 2	n (%)	Model 1	Model 2	n cases (%)	Model 1	Model 2
**Negative events**									
**Europe/Not obese**									
No	736 (7.4)	1.00 (Reference)	1.00 (Reference)	729 (2.5)	1.00 (Reference)	1.00 (Reference)	719 (8.5)	1.00 (Reference)	1.00 (Reference)
Yes	1003 (7.7)	0.98 (0.67-1.40)	0.99 (0.69-1.43)	992 (2.0)	0.77 (0.40-1.48)	0.71 (0.36-1.42)	971 (7.9)	0.91 (0.64-1.29)	0.89 (0.62-1.29)
**Europe/Obese**									
No	390 (9.2)	1.00 (Reference)	1.00 (Reference)	375 (2.9)	1.00 (Reference)	1.00 (Reference)	369 (8.7)	1.00 (Reference)	1.00 (Reference)
Yes	607 (9.6)	1.05 (0.67-1.63)	1.14 (0.72-1.82)	590 (3.2)	0.97 (0.44-2.11)	1.01 (0.44-2.26)	581 (9.6)	1.15 (0.73-1.82)	1.15 (0.71-1.85)
**Ghana/Not obese**									
No	588 (9.5)	1.00 (Reference)	1.00 (Reference)	592 (4.7)	1.00 (Reference)	1.00 (Reference)	588 (10.0)	1.00 (Reference)	1.00 (Reference)
Yes	1324 (8.5)	0.86 (0.61-1.21)	0.83 (0.59-1.17)	1331 (3.4)	0.64 (0.39-1.05)	0.65 (0.39-1.83)	1321 (9.0)	0.86 (0.61-1.20)	0.84 (0.60-1.18)
**Ghana/Obese**									
No	144 (13.9)	1.00 (Reference)	1.00 (Reference)	144 (3.5)	1.00 (Reference)	1.00 (Reference)	144 (14.6)	1.00 (Reference)	1.00 (Reference)
Yes	268 (13.1)	0.91 (0.50-1.64)	0.91 (0.49-1.65)	267 (3.8)	1.04 (0.34-3.16)	1.09 (0.35-3.37)	266 (14.3)	0.94 (0.52-1.68)	0.94 (0.52-1.67)
**Discrimination**									
**Europe/Not obese**									
No	1186 (8.4)	1.00 (Reference)	1.00 (Reference)	1172 (2.4)	1.00 (Reference)	1.00 (Reference)	1150 (9.4)	1.00 (Reference)	1.00 (Reference)
Yes	538 (5.9)	0.69 (0.46-1.05)	0.71 (0.46-1.11)	532 (1.5)	0.62 (0.28-1.37)	0.57 (0.24-1.34)	525 (5.9)	**0.63 (0.42-0.96)**	**0.63 (0.41-0.97)**
**Europe/Obese**									
No	709 (8.7)	1.00 (Reference)	1.00 (Reference)	691 (3.0)	1.00 (Reference)	1.00 (Reference)	678 (8.9)	1.00 (Reference)	1.00 (Reference)
Yes	269 (10.0)	1.14 (0.71-1.85)	1.29 (0.78-2.12)	256 (3.5)	1.15 (0.50-2.63)	1.27 (0.54-2.99)	254 (9.8)	1.13 (0.69-1.86)	1.22 (0.73-2.13)
**Ghana/Not obese**									
No	1648 (9.1)	1.00 (Reference)	1.00 (Reference)	1661 (3.9)	1.00 (Reference)	1.00 (Reference)	1646 (9.6)	1.00 (Reference)	1.00 (Reference)
Yes	81 (8.6)	1.06 (0.48-2.38)	1.04 (0.47-2.32)	81 (2.5)	1.04 (0.48-2.32)	0.86 (0.20-3.69)	81 (7.4)	0.90 (0.38-2.12)	0.89 (0.38-2.11)

For peer review only - http://bmjopen.bmj.com/site/about/guidelines.xhtml

**Ghana/Obese**									
No	383 (13.8)	1.00 (Reference)	1.00 (Reference)	383 (3.7)	1.00 (Reference)	1.00 (Reference)	382 (14.9)	1.00 (Reference)	1.00 (Reference)
Yes	23 (4.4)	0.31 (0.04-2.36)	0.40 (0.05-3.11)	23 (0.0)	**** (***-***)	**** (***-***)	23 (4.4)	0.29 (0.04-2.22)	0.34 (0.04-2.66)
**Stress at home/work**									
**Europe/Not obese**									
Never	855 (7.4)	1.00 (Reference)	1.00 (Reference)	847 (3.4)	1.00 (Reference)	1.00 (Reference)	831 (8.2)	1.00 (Reference)	1.00 (Reference)
Some stress	629 (8.3)	1.16 (0.79-1.70)	1.20 (0.81-1.79)	627 (1.4)	0.52 (0.24-1.12)	0.47 (0.21-1.07)	615 (7.9)	0.98 (0.66-1.43)	0.98 (0.65-1.46)
Several/Permanent stresses	247 (6.9)	0.93 (0.53-1.62)	1.15 (0.77-1.72)	239 (2.3)	0.45 (0.13-1.52)	0.49 (0.15-1.67)	236 (9.1)	0.97 (0.57-1.65)	1.04 (0.61-1.78)
**Europe/Obese**									
Never	472 (9.8)	1.00 (Reference)	1.00 (Reference)	455 (3.9)	1.00 (Reference)	1.00 (Reference)	448 (10.3)	1.00 (Reference)	1.00 (Reference)
Some stress	372 (7.5)	0.72 (0.44-1.18)	0.79 (0.47-1.32)	356 (1.4)	0.41 (0.15-1.16)	0.43 (0.15-1.21)	352 (9.5)	0.94 (0.57-1.53)	1.05 (0.62-1.76)
Several/Permanent stresses	147 (12.2)	1.32 (0.74-2.38)	1.31 (0.72-2.42)	148 (4.1)	1.05 (0.40-2.79)	1.09 (0.40-2.91)	144 (8.5)	1.36 (0.74-2.49)	1.41 (0.75-2.64)
**Ghana/Not obese**									
Never	537 (9.7)	1.00 (Reference)	1.00 (Reference)	542 (3.1)	1.00 (Reference)	1.00 (Reference)	537 (8.9)	1.00 (Reference)	1.00 (Reference)
Some stress	1055 (8.3)	0.79 (0.56-1.15)	0.74 (0.52-1.07)	1057 (3.9)	1.16 (0.65-2.09)	1.21 (0.67-2.18)	1052 (9.0)	0.92 (0.64-1.35)	0.90 (0.62-1.32)
Several/permanent stresses	319 (8.5)	0.81 (0.49-1.32)	0.73 (0.44-1.20)	323 (4.3)	1.34 (0.64-2.83)	1.43 (0.67-3.04)	319 (10.7)	1.12 (0.69-1.79)	1.09 (0.68-1.76)
**Ghana/Obese**									
Never	145 (12.4)	1.00 (Reference)	1.00 (Reference)	146 (4.1)	1.00 (Reference)	1.00 (Reference)	145 (13.8)	1.00 (Reference)	1.00 (Reference)
Some stress	221 (14.9)	1.21 (0.64-2.26)	1.05 (0.55-1.99)	219 (3.7)	0.74 (0.25-2.22)	0.83 (0.26-2.06)	219 (15.9)	1.13 (0.62-2.07)	1.02 (0.55-1.89)
Several/Permanent stresses	46 (8.7)	0.64 (0.20-2.01)	0.52 (0.16-1.65)	46 (2.2)	0.41 (0.05-3.61)	0.45 (0.05-4.01)	46 (8.8)	0.55 (0.17-1.71)	0.47 (0.14-1.50)
**Depressive symptoms**									
**Europe/Not obese**									
No	1601 (7.9)	1.00 (Reference)	1.00 (Reference)	1586 (2.2)	1.00 (Reference)	1.00 (Reference)	1558 (8.3)	1.00 (Reference)	1.00 (Reference)
Yes	121 (4.9)	0.59 (0.25-1.36)	0.65 (0.27-1.48)	117 (1.7)	0.79 (0.18-2.35)	0.86 (0.21-3.80)	115 (5.22)	0.58 (0.25-1.35)	0.64 (0.27-1.49)
**Europe/Obese**									
No	899 (9.7)	1.00 (Reference)	1.00 (Reference)	866 (3.4)	1.00 (Reference)	1.00 (Reference)	853 (9.4)	1.00 (Reference)	1.00 (Reference)

For peer review only - http://bmjopen.bmj.com/site/about/guidelines.xhtml

Yes	83 (7.2)	0.71 (0.29-1.67)	0.72 (0.30-1.74)	83 (1.2)	0.46 (0.06-3.52)	0.46 (0.06-3.57)	82 (8.5)	0.89 (0.40-2.01)	0.90 (0.40-2.04)
**Ghana/Not obese**									
No	1811 (8.9)	1.00 (Reference)	1.00 (Reference)	1822 (3.9)	1.00 (Reference)	1.00 (Reference)	1808 (9.4)	1.00 (Reference)	1.00 (Reference)
Yes	100 (6.0)	0.57 (0.24-1.33)	0.56 (0.24-1.32)	100 (2.0)	0.38 (0.09-1.63)	0.38 (0.09-1.63)	100 (8.0)	0.69 (0.32-1.46)	0.69 (0.32-1.47)
**Ghana/Obese**									
No	398 (13.8)	1.00 (Reference)	1.00 (Reference)	397 (3.5)	1.00 (Reference)	1.00 (Reference)	396 (14.7)	1.00 (Reference)	1.00 (Reference)
Yes	14 (0.0)	*** (****-****)	*** (****-****)	14 (7.1)	1.76 (0.21-14.89)	2.14 (0.25-8.78)	14 (7.1)	0.42 (0.05-3.32)	0.38 (0.05-3.07)

Model 1, adjusted for age and sex; Model 2, adjusted for age, sex, and educational level for Ghanaians (SSA) and length of stay for those in Europe; Abbreviations: CI, confidence interval; ACR, albumin creatinine ration; eGFR, estimated glomerular filtration rate; CKD, chronic kidney disease; OR, odds ratio, n= total number of Ghanaians living in Ghana and Europe among the various levels of PS constructs; %, proportion of individuals with CKD among the various levels of PS constructs in Ghana and Europe.

For peer review only

S3: **Association of psychosocial stressors (PS) indicators (negative life events, discrimination, stress at work/home and depression) with albuminuria, reduced eGFR and CKD risk for Ghanaians living in Ghana and those living in Europe stratified by diabetes status**

	Albuminuria (ACR ≥ 3 mg/mmol)			eGFR < 60 mL/min/1.73 m2			High to very high CKD risk (KDIGO, 2012)		
		OR (95% CI)			OR (95% CI)			OR (95% CI)	
	n (%)	Model 1	Model 2	n (%)	Model 1	Model 2	n cases (%)	Model 1	Model 2
**Negative events**									
**Europe/No diabetes**									
No	986 (8.0)	1.00 (Reference)	1.00 (Reference)	971 (2.1)	1.00 (Reference)	1.00 (Reference)	957 (7.9)	1.00 (Reference)	1.00 (Reference)
Yes	1359 (7.7)	0.95 (0.70-1.29)	0.97 (0.71-1.34)	1340 (2.0)	0.90 (0.50-1.63)	0.90 (0.48-1.68)	1315 (8.1)	1.01 (0.75-1.37)	0.99 (0.72-1.36)
**Europe/Diabetes**									
No	142 (9.9)	1.00 (Reference)	1.00 (Reference)	135 (6.7)	1.00 (Reference)	1.00 (Reference)	133 (12.8)	1.00 (Reference)	1.00 (Reference)
Yes	256 (12.5)	1.28 (0.66-2.50)	1.41 (0.71-2.82)	247 (4.9)	0.75 (0.29-1.89)	10.68 (0.27-1.77)	242 (11.2)	0.87 (0.46-1.69)	0.96 (0.49-1.88)
**Ghana/No diabetes**									
No	683 (9.1)	1.00 (Reference)	1.00 (Reference)	687 (4.1)	1.00 (Reference)	1.00 (Reference)	683 (9.8)	1.00 (Reference)	1.00 (Reference)
Yes	1447 (7.7)	0.84 (0.60-1.16)	0.82 (0.59-1.14)	1451 (3.1)	0.69 (0.42-1.13)	0.69 (0.42-1.13)	1442 (8.3)	0.82 (0.59-1.12)	0.80 (0.58-1.10)
**Ghana/Diabetes**									
No	49 (28.3)	1.00 (Reference)	1.00 (Reference)	49 (10.2)	1.00 (Reference)	1.00 (Reference)	49 (26.4)	1.00 (Reference)	1.00 (Reference)
Yes	148 (24.3)	0.78 (0.37-1.61)	0.72 (0.34-1.52)	150 (7.3)	0.55 (0.16-1.81)	0.57 (0.17-1.88)	148 (26.4)	0.93 (0.45-1.97)	0.90 (0.43-1.92)
**Discrimination**									
**Europe/No diabetes**									
No	1629 (7.9)	1.00 (Reference)	1.00 (Reference)	1609 (2.2)	1.00 (Reference)	1.00 (Reference)	1580 (8.3)	1.00 (Reference)	1.00 (Reference)
Yes	684 (6.7)	0.85 (0.60-1.21)	0.89 (0.62-1.28)	669 (1.5)	0.68 (0.33-1.40)	0.69 (0.32-1.47)	661 (7.0)	0.84 (0.59-1.20)	0.86 (0.59-1.25)
**Europe/Diabetes**									

For peer review only - http://bmjopen.bmj.com/site/about/guidelines.xhtml

For peer review only

No	270 (11.9)	1.00 (Reference)	1.00 (Reference)	258 (5.4)	1.00 (Reference)	1.00 (Reference)	252 (13.1)	1.00 (Reference)	1.00 (Reference)
Yes	126 (11.1)	0.87 (0.44-1.71)	0.89 (0.45-1.76)	122 (5.7)	1.29 (0.48-3.45)	1.25 (0.46-3.37)	121 (9.1)	0.68 (0.32-1.40)	0.69 (0.33-1.42)
**Ghana/No diabetes**									
No	1854 (8.4)	1.00 (Reference)	1.00 (Reference)	1865 (3.5)	1.00 (Reference)	1.00 (Reference)	1851 (9.0)	1.00 (Reference)	1.00 (Reference)
Yes	99 (8.1)	1.03 (0.49-2.17)	1.04 (0.49-2.19)	99 (2.0)	0.79 (0.19-3.33)	0.78 (0.18-3.29)	99 (7.1)	0.87 (0.39-1.93)	0.88 (0.39-1.95)
**Ghana/Diabetes**									
No	180 (26.7)	1.00 (Reference)	1.00 (Reference)	182 (7.7)	1.00 (Reference)	1.00 (Reference)	180 (27.2)	1.00 (Reference)	1.00 (Reference)
Yes	5 (0.0)	**** (***-***)	0.40 (0.05-3.11)	5 (0.0)	**** (***-***)	**** (***-***)	5 (0.0)	**** (***-***)	**** (***-***)
**Stress at home/work**									
**Europe/No diabetes**									
Never	1137 (7.9)	1.00 (Reference)	1.00 (Reference)	1127 (2.3)	1.00 (Reference)	1.00 (Reference)	1102 (8.0)	1.00 (Reference)	1.00 (Reference)
Some stress	860 (7.2)	0.90 (0.65-1.27)	0.96 (0.68-1.36)	850 (1.2)	0.49 (0.24-1.02)	0.49 (0.22-1.07)	835 (7.4)	0.94 (0.67-1.31)	0.99 (0.69-1.41)
Several/Permanent stresses	335 (8.4)	1.04 (0.67-1.62)	1.07 (0.68-1.70)	327 (2.1)	0.84 (0.36-1.95)	0.92 (0.39-2.17)	322 (9.6)	1.20 (0.77-1.85)	1.28 (0.82-1.99)
**Europe/Diabetes**									
Never	193 (9.8)	1.00 (Reference)	1.00 (Reference)	184 (8.2)	1.00 (Reference)	1.00 (Reference)	180 (11.7)	1.00 (Reference)	1.00 (Reference)
Some stress	142 (12.7)	1.22 (0.61-2.45)	1.38 (0.67-2.81)	134 (3.0)	0.43 (0.13-1.39)	0.40 (0.12-1.29)	133 (12.0)	1.04 (0.52-2.09)	1.13 (0.56-2.32)
Several/Permanent stresses	62 (12.9)	1.31 (0.54-3.16)	1.38 (0.56-3.41)	63 (3.2)	0.49 (0.10-2.29)	0.46 (0.10-2.23)	61 (9.8)	0.82 (0.31-2.13)	0.89 (0.34-2.35)
**Ghana/No diabetes**									
Never	628 (7.8)	1.00 (Reference)	1.00 (Reference)	634 (2.7)	1.00 (Reference)	1.00 (Reference)	628 (7.6)	1.00 (Reference)	1.00 (Reference)
Some stress	1163 (8.6)	1.06 (0.74-1.51)	0.97 (0.67-1.39)	1163 (3.7)	1.27 (0.71-2.27)	1.32 (0.74-2.34)	1158 (9.3)	1.15 (0.80-1.65)	1.10 (0.77-1.58)
Several/permanent stresses	339 (7.4)	0.88 (0.53-1.46)	0.76 (0.46-1.27)	341 (3.5)	1.24 (0.58-2.67)	1.35 (0.62-2.95)	339 (8.9)	1.07 (0.66-1.73)	1.00 (0.61-1.64)
**Ghana/Diabetes**									
Never	54 (38.9)	1.00 (Reference)	1.00 (Reference)	54 (11.1)	1.00 (Reference)	1.00 (Reference)	54 (37.0)	1.00 (Reference)	1.00 (Reference)
Some stress	116 (18.9)	**0.37 (0.37-0.77)**	**0.35 (0.17-0.75)**	116 (6.0)	0.47 (0.14-1.60)	0.45 (0.12-1.55)	116 (19.8)	**0.43 (0.21-0.89)**	**0.40 (0.19-0.86)**
Several/Permanent stresses	26 (25.0)	0.44 (0.44-1.29)	0.39 (0.13-1.19)	28 (10.7)	0.65 (0.13-3.21)	0.62 (0.12-3.23)	26 (30.8)	0.69 (0.25-1.93)	0.64 (0.22-1.87)

For peer review only - http://bmjopen.bmj.com/site/about/guidelines.xhtml

**Depressive symptoms**									
**Europe/No diabetes**									
No	2154 (8.0)	1.00 (Reference)	1.00 (Reference)	2122 (2.1)	1.00 (Reference)	1.00 (Reference)	2087 (8.1)	1.00 (Reference)	1.00 (Reference)
Yes	167 (5.4)	0.63 (0.32-1.25)	0.69 (0.34-1.38)	163 (2.0)	0.65 (0.15-2.72)	0.72 (0.17-3.04)	161 (6.8)	0.58 (0.25-1.35)	0.86 (0.46-1.64)
**Europe/Diabetes**									
No	351 (12.0)	1.00 (Reference)	1.00 (Reference)	335 (35.9)	1.00 (Reference)	1.00 (Reference)	329 (12.5)	1.00 (Reference)	1.00 (Reference)
Yes	39 (10.3)	0.79 (0.26-1.35)	0.73 (0.24-2.19)	39 (2.6)	0.49 (0.06-3.97)	0.49 (0.06-3.99)	38 (7.9)	0.89 (0.40-2.01)	0.60 (0.17-2.08)
**Ghana/No diabetes**									
No	2027 (8.3)	1.00 (Reference)	1.00 (Reference)	2035 (3.4)	1.00 (Reference)	1.00 (Reference)	2022 (8.8)	1.00 (Reference)	1.00 (Reference)
Yes	102 (4.9)	0.50 (0.20-1.25)	0.48 (0.19-1.19)	102 (2.9)	0.64 (0.19-2.13)	0.67 (0.20-2.21)	102 (7.8)	0.69 (0.32-1.46)	0.71 (0.34-1.51)
**Ghana/Diabetes**									
No	185 (26.5)	1.00 (Reference)	1.00 (Reference)	187 (8.7)	1.00 (Reference)	1.00 (Reference)	185 27.6)	1.00 (Reference)	1.00 (Reference)
Yes	12 (8.3)	0.27 (0.03-2.13)	0.25 (0.03-2.01)	12 (0.0)	*** (****-****)	*** (***-***)	12 (8.3)	0.42 (0.05-3.32)	0.27 (0.03-2.19)

Model 1, adjusted for age and sex; Model 2, adjusted for age, sex, and educational level for Ghanaians (SSA) and length of stay for those in Europe; Abbreviations: CI, confidence interval; ACR, albumin creatinine ration; eGFR, estimated glomerular filtration rate; CKD, chronic kidney disease; OR, odds ratio, n= total number of Ghanaians living in Ghana and Europe among the various levels of PS constructs; %, proportion of individuals with CKD among the various levels of PS constructs in Ghana and Europe.

For peer review only

S4: Association of psychosocial stressors (PS) indicators (negative life events, discrimination, stress at work/home and depression) with albuminuria, reduced eGFR and CKD risk for Ghanaians living in Ghana and those living in Europe stratified by hypertensive status

	Albuminuria (ACR ≥ 3 mg/mmol)			eGFR < 60 mL/min/1.73 m2			High to very high CKD risk (KDIGO, 2012)		
		OR (95% CI)			OR (95% CI)			OR (95% CI)	
	n (%)	Model 1	Model 2	n (%)	Model 1	Model 2	n cases (%)	Model 1	Model 2
**Negative events**									
**Europe/No hypertension**									
No	568 (6.9)	1.00 (Reference)	1.00 (Reference)	562 (1.3)	1.00 (Reference)	1.00 (Reference)	551 (7.1)	1.00 (Reference)	1.00 (Reference)
Yes	743 (5.0)	0.72 (0.45-1.15)	0.78 (0.48-1.27)	740 (0.8)	0.59 (0.19-1.77)	0.60 (0.20-1.83)	724 (5.1)	0.71 (0.44-1.13)	0.75 (0.46-1.22)
**Europe/Hypertension**									
No	560 (9.6)	1.00 (Reference)	1.00 (Reference)	544 (4.0)	1.00 (Reference)	1.00 (Reference)	539 (10.0)	1.00 (Reference)	1.00 (Reference)
Yes	872 (11.4)	1.22 (0.86-1.73)	1.20 (0.83-1.73)	847 (3.9)	0.95 (0.54-1.67)	0.89 (0.49-1.62)	833 (11.6)	1.19 (0.83-1.69)	1.13 (0.78-1.62)
**Ghana/No Hypertension**									
No	540 (6.3)	1.00 (Reference)	1.00 (Reference)	543 (2.2)	1.00 (Reference)	1.00 (Reference)	540 (6.9)	1.00 (Reference)	1.00 (Reference)
Yes	1118 (5.6)	0.88 (0.57-1.36)	0.84 (0.54-1.31)	1124 (1.9)	0.82 (0.39-1.69)	0.86 (0.41-1.79)	1115 (6.4)	0.93 (0.61-1.40)	0.90 (0.59-1.36)
**Ghana/Hypertension**									
No	192 (21.9)	1.00 (Reference)	1.00 (Reference)	193 (10.9)	1.00 (Reference)	1.00 (Reference)	192 (22.4)	1.00 (Reference)	1.00 (Reference)
Yes	476 (18.1)	0.78 (0.52-1.18)	0.79 (0.52-1.20)	476 (6.9)	0.58 (0.32-1.04)	0.58 (0.33-1.05)	474 (18.4)	0.77 (0.50-1.16)	0.76 (0.51-1.16)

For peer review only - http://bmjopen.bmj.com/site/about/guidelines.xhtml

For peer review only

**Discrimination**									
**Europe/No hypertension**									
No	916 (6.3)	1.00 (Reference)	1.00 (Reference)	907 (0.8)	1.00 (Reference)	1.00 (Reference)	885 (6.8)	1.00 (Reference)	1.00 (Reference)
Yes	379 (3.7)	0.60 (0.33-1.09)	0.65 (0.36-1.21)	378 (1.1)	1.35 (0.39-4.74)	1.37 (0.38-4.89)	374 (3.2)	**0.47 (0.25-0.89)**	**0.51 (0.27-0.97)**
**Europe/Hypertension**									
No	983 (10.5)	1.00 (Reference)	1.00 (Reference)	960 (4.4)	1.00 (Reference)	1.00 (Reference)	947 (10.9)	1.00 (Reference)	1.00 (Reference)
Yes	431 (10.7)	1.03 (0.71-1.49)	1.05 (0.72-1.54)	413 (3.2)	0.73 (0.38-1.39)	0.75 (0.38-1.48)	408 (11.0)	1.03 (0.71-1.49)	1.04 (0.71-1.53)
**Ghana/No hypertension**									
No	1427 (5.7)	1.00 (Reference)	1.00 (Reference)	1437 (2.2)	1.00 (Reference)	1.00 (Reference)	1424 (6.5)	1.00 (Reference)	1.00 (Reference)
Yes	81 (7.4)	1.30 (0.55-3.09)	1.30 (0.55-3.10)	81 (0.0)	**** (***-***)	**** (***-***)	81 (7.4)	1.21 (0.51-2.88)	1.22 (0.51-2.89)
**Ghana/Hypertension**									
No	606 (20.3)	1.00 (Reference)	1.00 (Reference)	609 (7.9)	1.00 (Reference)	1.00 (Reference)	606 (20.3)	1.00 (Reference)	1.00 (Reference)
Yes	23 (8.7)	0.38 (0.09-1.68)	0.40 (0.09-1.74)	23 (8.7)	1.27 (0.28-5.70)	1.20 (0.26-5.44)	23 (4.4)	0.19 (0.03-1.44)	0.19 (0.03-1.45)
**Stress at home/work**									
**Europe/No hypertension**									
Never	644 (6.1)	1.00 (Reference)	1.00 (Reference)	640 (1.3)	1.00 (Reference)	1.00 (Reference)	625 (5.8)	1.00 (Reference)	1.00 (Reference)
Some stress	468 (4.5)	0.74 (0.43-1.27)	0.81 (0.46-1.43)	464 (0.4)	0.37 (0.07-1.74)	0.41 (0.09-2.01)	457 (4.8)	0.84 (0.49-1.45)	0.91 (0.52-1.60)
Several/Permanent stresses	192 (6.8)	1.07 (0.56-2.05)	1.19 (0.61-2.29)	191 (1.6)	1.49 (0.38-5.78)	1.52 (0.39-5.98)	186 (8.1)	1.38 (0.74-2.60)	1.49 (0.79-2.84)
**Europe/Hypertension**									
Never	686 (10.2)	1.00 (Reference)	1.00 (Reference)	665 (5.4)	1.00 (Reference)	1.00 (Reference)	657 (11.1)	1.00 (Reference)	1.00 (Reference)
Some stress	534 (11.1)	1.03 (0.71-1.49)	1.05 (0.72-1.54)	520 (2.3)	**0.49 (0.25-0.96)**	**0.47 (0.23-0.95)**	511 (10.9)	0.97 (0.67-1.41)	0.99 (0.67-0.47)
Several/Permanent stresses	205 (11.2)	1.11 (0.67-1.82)	1.12 (0.67-1.87)	199 (3.0)	0.56 (0.23-1.37)	0.58 (0.24-1.43)	197 (11.2)	0.99 (0.59-1.64)	1.03 (0.61-1.74)
**Ghana/No hypertension**									
Never	495 (5.5)	1.00 (Reference)	1.00 (Reference)	498 (1.4)	1.00 (Reference)	1.00 (Reference)	495 (5.9)	1.00 (Reference)	1.00 (Reference)
Some stress	899 (5.9)	1.07 (0.66-1.73)	0.99 (0.61-1.60)	901 (2.2)	1.30 (0.54-3.15)	1.39 (0.57-3.37)	896 (6.5)	1.04 (0.65-1.65)	1.00 (0.63-1.59)

For peer review only - http://bmjopen.bmj.com/site/about/guidelines.xhtml

Several/permanent stresses	263 (5.7)	1.01 (0.53-1.95)	0.89 (0.46-1.72)	267 (2.6)	1.69 (0.58-5.01)	1.89 (0.63-5.65)	263 (7.6)	1.21 (0.67-2.20)	1.15 (0.63-2.11)
**Ghana/Hypertension**									
Never	187 (22.9)	1.00 (Reference)	1.00 (Reference)	190 (8.4)	1.00 (Reference)	1.00 (Reference)	187 (20.9)	1.00 (Reference)	1.00 (Reference)
Some stress	379 (18.2)	0.74 (0.48-1.14)	0.68 (0.43-1.05)	377 (7.9)	0.95 (0.50-1.80)	0.99 (0.51-1.91)	377 (19.4)	0.90 (0.58-1.40)	0.87 (0.55-1.36)
Several/Permanent stresses	102 (15.7)	0.62 (0.33-1.17)	0.54 (0.28-1.04)	102 (7.8)	0.92 (0.37-2.26)	0.97 (0.39-2.43)	102 (17.7)	0.79 (0.42-1.49)	0.76 (0.40-1.43)
**Depressive symptoms**									
**Europe/No hypertension**									
No	1195 (5.9)	1.00 (Reference)	1.00 (Reference)	1187 (1.1)	1.00 (Reference)	1.00 (Reference)	1162 (6.0)	1.00 (Reference)	1.00 (Reference)
Yes	101 (3.9)	0.59 (0.21-1.65)	0.64 (0.23-1.82)	99 (1.0)	1.18 (0.15-2.72)	1.27 (0.16-10.17)	98 (5.1)	0.77 (0.30-1.97)	0.84 (0.33-2.17)
**Europe/Hypertension**									
No	1310(10.9)	1.00 (Reference)	1.00 (Reference)	1270 (4.1)	1.00 (Reference)	1.00 (Reference)	1254 (11.2)	1.00 (Reference)	1.00 (Reference)
Yes	105 (8.6)	0.76 (0.37-1.53)	0.75 (0.37-1.55)	103 (1.9)	0.52 (0.12-2.18)	0.56 (0.13-2.38)	101 (8.9)	0.77 (0.38-1.58)	0.80 (0.39-1.63)
**Ghana/No hypertension**									
No	1575 (5.9)	1.00 (Reference)	1.00 (Reference)	1584 (2.1)	1.00 (Reference)	1.00 (Reference)	1572 (6.7)	1.00 (Reference)	1.00 (Reference)
Yes	82 (2.4)	0.36 (0.09-1.51)	0.35 (0.08-1.44)	82 (1.2)	0.39 (0059-2.95)	0.39 (0.05-2.97)	82 (3.7)	0.44 (0.13-1.42)	0.43 (0.13-1.40)
**Ghana/Hypertension**									
No	636 (19.5)	1.00 (Reference)	1.00 (Reference)	637 (8.2)	1.00 (Reference)	1.00 (Reference)	634 (19.6)	1.00 (Reference)	1.00 (Reference)
Yes	32 (12.5)	0.59 (0.20-1.72)	0.59 (0.20-1.74)	32 (6.3)	0.74 (0.17-3.25)	0.76 (0.17-3.33)	32 (18.8)	0.94 (0.0.37-2.37)	0.94 (0.38-2.37)

Model 1, adjusted for age and sex; Model 2, adjusted for age, sex, and educational level for Ghanaians (SSA) and length of stay for those in Europe; Abbreviations: CI, confidence interval; ACR, albumin creatinine ration; eGFR, estimated glomerular filtration rate; CKD, chronic kidney disease; OR, odds ratio, n= total number of Ghanaians living in Ghana and Europe among the various levels of PS constructs; %, proportion of individuals with CKD among the various levels of PS constructs in Ghana and Europe.

For peer review only - http://bmjopen.bmj.com/site/about/guidelines.xhtml

For peer review only

For peer review only - http://bmjopen.bmj.com/site/about/guidelines.xhtml

STROBE Statement—checklist of items that should be included in reports of observational studies

	Item No.	Recommendation	Page No.	Relevant text from manuscript
**Title and abstract**	1	(*a*) Indicate the study's design with a commonly used term in the title or the abstract	1,2	We have included a commonly used term in the title and abstract
		(*b*) Provide in the abstract an informative and balanced summary of what was done and what was found	2	Our study shows no convincing evidence for associations between stress as indicated by four PS constructs and prevalence of CKD. Further studies aimed at identifying potential factors driving the high prevalence of CKD among these populations are needed.
**Introduction**				
Background/rationale	2	Explain the scientific background and rationale for the investigation being reported	4	The theoretical and scientific background as well as the rationale for conducting the study have been provided in the introduction section.
Objectives	3	State specific objectives, including any prespecified hypotheses	4	We examined the

For peer review only - http://bmjopen.bmj.com/site/about/guidelines.xhtml
For peer review only

				association between PS and CKD prevalence among rural and urban Ghanaians and their migrants living in three European cities. We also assessed if the influence of PS on CKD is partially mediated by primary risk factors (hypertension and diabetes) of CKD.
**Methods**				
Study design	4	Present key elements of study design early in the paper	5-6	Details given in the methods
Setting	5	Describe the setting, locations, and relevant dates, including periods of recruitment, exposure, follow-up, and data collection	5-6	Rural or urban Ghana and three (Amsterdam, Berlin, London) European cities (migrants)
Participants	6	(*a*) *Cohort study*—Give the eligibility criteria, and the sources and methods of selection of participants. Describe methods of follow-up<br>*Case-control study*—Give the eligibility criteria, and the sources and methods of case ascertainment and control selection. Give the rationale for the choice of cases and controls<br>*Cross-sectional study*—Give the eligibility criteria, and the sources and methods of selection of participants	6-8	A multi-centred cross sectional baseline data from the Research on Obesity and Diabetes among African Migrants (RODAM) study. A random sample of

For peer review only

				5,659 adults (Europe, 3167, rural, 1,043, Urban Ghana, 1,449, Ghana) aged 25 to 70 years.
		(*b*) *Cohort study*—For matched studies, give matching criteria and number of exposed and unexposed<br>*Case-control study*—For matched studies, give matching criteria and the number of controls per case		
Variables	7	Clearly define all outcomes, exposures, predictors, potential confounders, and effect modifiers. Give diagnostic criteria, if applicable	6-8	The main outcomes have been clearly defined.
Data sources/ measurement	8*	For each variable of interest, give sources of data and details of methods of assessment (measurement). Describe comparability of assessment methods if there is more than one group	*6-8*	We defined each variable of interest in the methods accordingly
Bias	9	Describe any efforts to address potential sources of bias	18	Potential sources of bias have discussed in the discussion section
Study size	10	Explain how the study size was arrived at	5	Given in the methods section and we have also referred to the RODAM study methods paper

Continued on next page

For peer review only - http://bmjopen.bmj.com/site/about/guidelines.xhtml

Quantitative variables	11	Explain how quantitative variables were handled in the analyses. If applicable, describe which groupings were chosen and why	8-9	Please see methods
Statistical methods	12	(*a*) Describe all statistical methods, including those used to control for confounding	8-9	Please see methods
		(*b*) Describe any methods used to examine subgroups and interactions	8-9	Please see methods
		(*c*) Explain how missing data were addressed	8-9	
		(*d*) *Cohort study*—If applicable, explain how loss to follow-up was addressed<br>*Case-control study*—If applicable, explain how matching of cases and controls was addressed<br>*Cross-sectional study*—If applicable, describe analytical methods taking account of sampling strategy	NA	We have reported non-response across sites
		(*e*) Describe any sensitivity analyses	NA	
**Results**				
Participants	13*	(a) Report numbers of individuals at each stage of study—eg numbers potentially eligible, examined for eligibility, confirmed eligible, included in the study, completing follow-up, and analysed	5	Non-response analysis was done to shed light on the differential response rates across sites
		(b) Give reasons for non-participation at each stage	5	
		(c) Consider use of a flow diagram	5	We have also referred to RODAM methods paper
Descriptive data	14*	(a) Give characteristics of study participants (eg demographic, clinical, social) and information on exposures and potential confounders	5	We have also referred to RODAM methods paper
		(b) Indicate number of participants with missing data for each variable of interest	5	We have also referred to RODAM methods paper
		(c) *Cohort study*—Summarise follow-up time (eg, average and total amount)	NA	
Outcome data	15*	*Cohort study*—Report numbers of outcome events or summary measures over time	*NA*	
		Case-control study—Report numbers in each exposure category, or summary measures of exposure	*NA*	
		Cross-sectional study—Report numbers of outcome events or summary measures	*9-10*	Summary measures are given in the results section and in tables and figures
Main results	16	(*a*) Give unadjusted estimates and, if applicable, confounder-adjusted estimates and their precision (eg, 95% confidence interval). Make clear which confounders were adjusted for and why they were included	*12-16*	Unadjusted and adjusted estimates are given in the results section and in figures
		(*b*) Report category boundaries when continuous variables were categorized	12-16	We have provided mean and corresponding standard deviations for the continuous variables.

For peer review only - http://bmjopen.bmj.com/site/about/guidelines.xhtml

		(*c*) If relevant, consider translating estimates of relative risk into absolute risk for a meaningful time	NA	

Continued on next page

Other analyses	17	Report other analyses done—eg analyses of subgroups and interactions, and sensitivity analyses	NA	
**Discussion**				
Key results	18	Summarise key results with reference to study objectives	8	
Limitations	19	Discuss limitations of the study, taking into account sources of potential bias or imprecision. Discuss both direction and magnitude of any potential bias	18	Key limitations regarding study methods including differential response rates and sampling methods in the various study sites have been provided
Interpretation	20	Give a cautious overall interpretation of results considering objectives, limitations, multiplicity of analyses, results from similar studies, and other relevant evidence	17-18	Cautious overall interpretation of the key findings have been provided.
Generalisability	21	Discuss the generalisability (external validity) of the study results	17-18	
**Other information**				
Funding	22	Give the source of funding and the role of the funders for the present study and, if applicable, for the original study on which the present article is based	19	The funders had no role in study design, data collection and analysis, decision to publish, or preparation of the manuscript

*Give information separately for cases and controls in case-control studies and, if applicable, for exposed and unexposed groups in cohort and cross-sectional studies.

Note: An Explanation and Elaboration article discusses each checklist item and gives methodological background and published examples of transparent reporting. The STROBE checklist is best used in conjunction with this article (freely available on the Web sites of PLoS Medicine at http://www.plosmedicine.org/, Annals of Internal Medicine at http://www.annals.org/, and Epidemiology at http://www.epidem.com/). Information on the STROBE Initiative is available at www.strobe-statement.org.

For peer review only - http://bmjopen.bmj.com/site/about/guidelines.xhtml

BMJ Open

A CROSS SECTIONAL STUDY OF ASSOCIATION BETWEEN PSYCHOSOCIAL STRESSORS WITH CHRONIC KIDNEY DISEASE AMONG MIGRANT AND NON-MIGRANT GHANAIANS LIVING IN EUROPE AND GHANA: THE RODAM STUDY

Journal:	*BMJ Open*
Manuscript ID	bmjopen-2018-027931.R1
Article Type:	Research
Date Submitted by the Author:	12-Mar-2019

Complete List of Authors:	Adjei Nana, David ; University of Ghana, Department of Medical Laboratory Sciences; University of Amsterdam, Department of Public Health<br>Stronks, Karien; Academic Medical Center , Department of Public Health<br>Adu, Dwomoa; Korle-bu Teaching Hospital, Department of Medicine<br>Beune, Erik; AMC<br>Meeks, Karlijn; AMC, Public Health<br>Smeeth, Liam; London School of Hygiene and Tropical Medicine,<br>Addo, Juliet; London School of Hygiene and Tropical Medicine, Non Communicable Disease Epidemiology<br>Owusu-Dabo, Ellis; Kwame Nkrumah University of Science and Technology, Kumasi Centre for Collaborative Research in Tropical Medicine<br>Klipstein-Grobusch, Kerstin; 1 Julius Global Health, Julius Center for Health Sciences and Primary Care, University Medical Centre Utrecht, Utrecht, The Netherlands<br>Mockenhaupt, Frank; Charité – University Medicine, Berlin, Institute of Tropical Medicine and International Health<br>Schulze, Matthias; German Institute of Human Nutrition Potsdam-Rehbruecke<br>Danquah, Ina; German Institute of Human Nutrition, Molecular Epidemiology; Charite Universitatsmedizin Berlin, Institute for Social Medicine, Epidemiology and Health Economy<br>Spranger, Joachim; Department of Endocrinology and Metabolism, 1. Charité-University Medicine Berlin, Berlin, Germany.<br>Bahendeka, Silver; St. Francis Hospital Nsambya,<br>Agyemang, Charles; Academic Medical centre, University of Amsterdam, Department of Public Health

**Primary Subject Heading**:	Public health
Secondary Subject Heading:	Epidemiology, Public health, Renal medicine
Keywords:	Chronic Kidney Disease, Psychosocial stressors, migrants, rodam study, Europe, Ghana

SCHOLARONE™
Manuscripts

A CROSS SECTIONAL STUDY OF ASSOCIATION BETWEEN PSYCHOSOCIAL STRESSORS WITH CHRONIC KIDNEY DISEASE AMONG MIGRANT AND NON-MIGRANT GHANAIANS LIVING IN EUROPE AND GHANA: THE RODAM STUDY

David N. Adjei, MSc, PhD[1,2]; Karien Stronks, MSc, PhD[1]; Dwomoa Adu, MD[3]; Erik Beune, MSc, PhD [1]; Karlijn Meeks, MSc, PhD [1]; Liam Smeeth, MD, PhD[4] ; Juliet, Addo, MD, PhD [4]; Ellis Owusu-Dabo, MSc, PhD [5], Kerstin Klipstein-Grobusch, MSc, PhD [6,7]; Frank P. Mockenhaupt, MD, PhD [8] ; Matthias B. Schulze, MSc, PhD [9]; Ina, Danquah, MSc, PhD [9,10] ; Joachim, Spranger, MD, PhD [11,12,13]; Silver Bahendeka, MD, PhD[14]; Charles Agyemang, MPH, PHD[1]

1. Department of Public Health, Academic Medical Center, University of Amsterdam, Amsterdam, Amsterdam Public Health Research Institute, Amsterdam, The Netherlands.
2. Department of Medical Laboratory Sciences, School of Biomedical and Allied Health Sciences, College of Health Sciences, University of Ghana, Accra, Ghana.
3. Department of Medicine, School of Medicine and Dentistry, University of Ghana and Korle-Bu Teaching Hospital, Accra, Ghana.
4. Department of Non-Communicable Disease Epidemiology, London School of Hygiene and Tropical Medicine, London, United Kingdom.
5. Kumasi Centre for collaborative Research, KNUST, Kumasi, Ghana.
6. Julius Global Health, Julius Center for Health Sciences and Primary Care, University Medical Centre, Utrecht University, The Netherlands
7. Division of Epidemiology & Biostatistics, School of Public Health, Faculty of Health Sciences, University of the Witwatersrand, Johannesburg, South Africa
8. Institute of Tropical Medicine and International Health, Charité –University Medicine Berlin, Augustenburger Platz 1, 13353 Berlin, Germany.
9. Department of Molecular Epidemiology, German Institute of Human Nutrition Potsdam-Rehbrücke, Nuthetal, Germany.
10. Charité - Universitaetsmedizin Berlin, Institute for Social Medicine, Epidemiology and Health Economics, Berlin, Germany.
11. Department of Endocrinology and Metabolism, Charité-University Medicine Berlin, Berlin, Germany.
12. German Centre for Cardiovascular Research (DZHK), Berlin, Germany.
13. Center for Cardiovascular Research (CCR), Charité-University, Medicine, Berlin, Germany.

14. MKPGMS - Uganda Martyrs University, Kampala, Uganda.

For peer review only

For peer review only - http://bmjopen.bmj.com/site/about/guidelines.xhtml

Address correspondence to David Nana Adjei, MSc, Department of Public Health, Academic Medical Centre, University of Amsterdam, Meibergdreef 9, 1105 AZ Amsterdam, the Netherlands, School Biomedical and Allied Health Sciences, Medical Laboratory Sciences, University of Ghana, E-mail: dna@chs.edu.gh, d.n.adjei@amc.uva.nl, Tel: +233236717850

For peer review only

For peer review only - http://bmjopen.bmj.com/site/about/guidelines.xhtml

Abstract

Objectives: The association between Psychosocial stressors (PS) and CKD among sub-Saharan African (SSA) populations is unknown. We examined the association between PS and CKD prevalence among rural and urban Ghanaians and Ghanaian migrants living in three European cities. We also assessed if the influence of PS on CKD is partially mediated by primary risk factors (hypertension and diabetes) of CKD.

Design: A multi-centred cross sectional data from the Research on Obesity and Diabetes among African Migrants (RODAM) study.

Setting: Rural and urban Ghana and three European cities (Amsterdam, Berlin and London).

Participants: A random sample of 5,659 adults (Europe 3167, rural Ghana 1,043, and Urban Ghana 1,449) aged 25 to 70 years.

Explanatory and outcome measures: PS was defined by negative life events, perceived discrimination, perceived stress at work/home and depressive symptoms. Three CKD outcomes were considered using the 2012 KDIGO (Kidney Disease: Improving Global Outcomes) severity of CKD classification. Comparisons between PS and CKD outcomes were made using logistic regression analyses across all sites.

Results: We observed higher proportion of negative life events (68.7%) and perceived permanent stress (15.9%) among Ghanaians living in Ghana than Ghanaians living in Europe. Depressive symptoms (7.5%) and perceived discrimination (29.7%) were more common among Ghanaians living in Europe than Ghanaians living in Ghana. No significant association was observed between any of the PS constructs and CKD outcomes across sites except for positive association between stress at work/home and albuminuria (2.81, 95% C.I. 1.46-5.40) and CKD risk (2.78, 95% C.I. 1.43-5.43) among Ghanaians living in Berlin.

Conclusion: Our study shows no convincing evidence of associations between PS constructs and the prevalence of CKD outcomes. Further studies are needed to identify potential factors driving the high prevalence of CKD among these populations.

Index Words: Chronic kidney disease; psychosocial stressors; risk factor; migrants; RODAM study, Europe, Ghana

For peer review only - http://bmjopen.bmj.com/site/about/guidelines.xhtml

Strengths and limitations of the study

- This study used all three categories of CKD definitions (albuminuria, reduced eGFR and CKD risk) by KDIGO 2012 in assessing association of SS with CKD across all sites. This provided more detailed information on CKD outcomes.
- All sites in our study used well standardized study protocols and this eliminated intra protocol variability.
- The use of four constructs of psychosocial stressors (negative life events, perceived discrimination, perceived stress at work/home and depressive symptoms) provided a robust approach to assessing SS associations with CKD.
- The limitation of intra laboratory variability in earlier studies was eliminated using the same standard operating procedures in the same laboratory for running all samples for all sites.
- PS is captured and experienced in different magnitude across different populations. We were unable to ascertain if PS as defined in this study was adequately captured among Ghanaians living in rural and urban Ghana.

For peer review only

Introduction

Worldwide, Chronic Kidney Disease (CKD) is a leading cause of mortality and morbidity with several risk factors (diabetes mellitus, obesity, hypertension and cardiovascular disease) [1]. The epidemiologic transition in low-and-middle income countries (LMICs) shows increased burden of these risk factors [2-4]. CKD's high morbidity and mortality is mostly driven by uncontrolled comorbidities such as diabetes and hypertension [5 6]. CKD treatment and management cost is very high and not sustainable even in high-income countries and this underscores the need for prevention [7]. Available literature has shown that both individual and community level economic factors influence CKD [8-10]. However, after adjusting for both individual and community level socioeconomic position, differences in CKD risk among different populations remained [8 10 11]. These findings seem to suggest that other social environmental factors may be driving CKD prevalence and progression in high-risk populations. Recent studies have shown that whereas Ghanaians living in Europe have a higher CKD risk compared to their host nation populations, they have a lower CKD risk compared with their peers living in urban Ghana [12]. The increased risk of CKD observed in urban Ghana was not fully explained by conventional risk factors [12] and socio-economic status [13]. This underscores the need to identify other modifiable risk factors of CKD for prevention, optimum treatment and efficient management.

Evidence shows that where an individual works or stays influences his or her physiological wellbeing leading to an increased risk of chronic diseases [14 15]. Thus, migrants' physiological wellbeing is influenced by the environment (host nations) they move to stay. The association between PS and CKD as well as the biological pathways through which PS influences CKD progression is poorly understood and complex [5] although several pathways have been suggested [16 17]. Specifically, PS have been reported to be associated with alteration in the sympathetic/autonomic nervous system activity leading to higher rates of traditional risk factors of CKD including hypertension and diabetes [18-20]. Environmental stressors have been reported to contribute to the development of insulin resistance, metabolic syndrome, obesity and diabetes which if uncontrolled leads to CKD incidence [21 22]. Other studies [23 24] have suggested that stress attributable to social and/or economic disadvantage is associated with CKD development and progression through an interaction between other psychosocial factors and comorbid behaviors such as alcohol, tobacco and drug use [25]. In addition, undernutrition due to stress adversely impact on fetal environment by impeding fetal growth leading to low birth weight, which has been shown to be associated with CKD in adult life [25 26].

However, studies linking psychosocial stressors (PS) to CKD prevalence and progression vary greatly among different geographical populations. [5 16 27-31]. Specifically, in the USA whereas no association was found between PS and CKD [31-33] another study reported lower prevalence of CKD was associated with

For peer review only - http://bmjopen.bmj.com/site/about/guidelines.xhtml

greater life stressors at baseline [31]. In contrast, in the Netherlands depressive and anxiety symptoms were observed to be common among CKD patients and such patients had increased risk of poor clinical outcomes [28]. Similarly, a study conducted in Korea reported a positive relationship between depressive symptoms and CKD [27]. These observations suggest differential impact of PS at different geographical locations. For example, discrimination among migrants may differ greatly between host population and from their SSA compatriots. Specifically, some studies have reported differences in PS among rural and urban populations [34].

Current literature on the association between PS and CKD among sub-Saharan African populations and their migrants in Europe is scanty and uncertain. We therefore sought to determine the association between PS and CKD prevalence among Ghanaians in rural and urban Ghana and their migrants living in three European cities. Furthermore, we examined the influence of psychosocial stressors on risk factors (obesity, diabetes and hypertension) of CKD.

Methods

Study population and study design

For this study, data from the Research on Obesity & Diabetes among African Migrants (RODAM) study, a multi-centre cross sectional study, were used. The rationale, conceptual framework, design and methodology of the RODAM study have been described in detail elsewhere [12 13 35 36]. To summarize, the study was conducted from 2012 to 2015. Ghanaians aged 25–70 years living in rural and urban communities in Ghana as well as in three European cities (Amsterdam, Berlin and London) were included in this study. We standardized data collection across all sites. The ethics committees in Ghana, the Netherlands, Germany and the UK approved the study protocol prior to data collection. Informed consent was obtained from each participant prior to enrollment in the study. In Ghana, participants were randomly drawn from a list of 30 enumeration areas in the Ashanti region based on the 2010 population census. These enumeration areas came from both rural areas and two purposively selected urban cities (Kumasi and Obuasi). For Ghanaians in Amsterdam, we randomly drew participants from the Municipal register. This register holds data on country of birth of citizens and their parents, thus allowing for sampling based on the Dutch standard indicator for ethnic origin. London lacked a population register for migrant groups. Thus, Ghanaian organizations served as sampling frame for the study. Lists of these organizations were obtained from the Ghanaian Embassy and the Association of Ghanaian Churches in the UK in the boroughs known to have the greatest concentration of Ghanaians. Members were selected from the lists of all members of these organizations. In Berlin, the registration office of the federal state of Berlin provided a list of Ghanaian individuals in Berlin but this resulted in low response rate. Because of this, a change was made to use lists

For peer review only

of Ghanaian churches and organizations as the sampling frame. Across all sites in Europe, all selected participants were sent a written invitation combined with written information (information sheet) regarding the study and a response card. The participants were contacted by phone to schedule a date and location of the interview with a trained research assistant or opt for the self-administration of the paper questionnaire or digital online version depending on the preference of the participant. After the completion of the questionnaire, a date for physical examination was then scheduled after a positive response. The participants were instructed to fast from 10.00 p.m. the night before the physical examination. The response rate was 76% in rural Ghana and 74% in urban Ghana. In London, of those individuals who were registered in the various Ghanaian organizations and were invited, 75% agreed and participated in the study, while in Berlin, this figure was 68%, and 53% in Amsterdam. For the current study, 5898 participants with data available on both questionnaire data and physical measurements were used. Individuals who were outside the age range of 25–70 years (n=239) were excluded because not all the study sites had individuals outside this age range resulting in 5659 individuals comprising of 2492 from rural and urban Ghana and 3167 from the three European cities. In the conduct of analysis, we further excluded individuals with no data on CKD and all other indicators (n=52), resulting in a data set of 5607 participants for analysis.

Measurements

Covariates

Demographic and lifestyle factors

For this study, we obtained information on demographics, educational level and lifestyle factors (smoking and physical activity) by questionnaire. Physical examinations were performed across all sites using validated devices per standardized operational procedures. Weight was measured in light clothing and without shoes with SECA 877 scales to the nearest 0.1 kg. Height was measured without shoes with a portable stadiometer (SECA 217) to the nearest 0.1 cm. Body mass index (BMI) was calculated as weight (kg) divided by height squared (m2). Overweight was defined as BMI of 25 to < 30 kg/m^2 and obesity as BMI ≥ 30 kg/m^2. Waist circumference was measured in centimetres at the midpoint between the lower rib and the upper margin of the iliac crest. We used the same assessor for each participant in measuring all anthropometrics and each was measured twice; the average of the two measurements was used for analyses.

Predictor: SS

For this study, four constructs of psychosocial stress (discrimination, perceived stress at work or at home, negative life events and depressive symptoms) were used as explanatory variables.

Perceived discrimination

For peer review only - http://bmjopen.bmj.com/site/about/guidelines.xhtml

Everyday discrimination as perceived by participants was reported as routinely experiencing instances of unfair treatment. We used the the Everyday Discrimination Scale (EDS). The EDS comprises of a 9-items which rates the frequency at which participants experience daily mistreatment and it focuses on being treated with less courtesy or less respect, receiving poorer service than other people or being called names or insulted. Participants had the option of rating each of the 9-items from "never" = 1 to "very often" = 5. The obtained scores were summed and an average of the scores was computed to obtain a final score of 1 to 5. This scale was used because it is commonly used for self-reported discrimination [37], with consistent high reliability among a variety of ethnicities [38], comprising African migrants in the Netherland [39].

Perceived stress at work or at home

We defined perceived stress at work or at home as "sense of irritation, filled with anxiety, or as having difficulties in sleeping because of circumstances at work or at home". We used the psychological stress scale created by the INTERHEART study [40]. Participants in the study were asked about their opinion on frequency of stress at work and at home, and could answer "never", "some periods", "several periods", or "continually". Both answers were then combined into a composite score and graded into four categories: never experienced to experienced permanent stress at home or at work [40]. Due to the very small numbers in the permanent periods of stress group, we combined experienced several periods of stress at home or at work and permanent periods of stress at home or at work.

Negative life events

The presence of major negative life events among participants was perceived as any event that could cause acute stress to an individual. We therefore applied the well-validated and widely used List of Threatening Experiences (LTE) [41 42]. The scale comprised of 12 unpleasant events participants perceived to have experienced in the past 12 months. We used a slightly-altered version of LTE consisting of 9 unpleasant items. We dichotomized participants into two groups namely "no negative life events" and "one or more events" and participants in the second category were expected to have higher levels of stress [42].

Depressive symptoms

Depressive symptoms were measured by the 9-item Patient Health Questionnaire (PHQ-9). The PHQ-9 consists of nine items, with a response scales 0 'not at all', 1 'on several days', 2 'on more than half of the days' and 3 'nearly every day'. A participant was considered to be in a significant depressed mood (SDM) when one or both of the items 1 (little interest or pleasure in doing things) and 2 (feeling down, depressed, or hopeless) were answered with at least 'on more than half of the days', and at least 5 of the 9 items were answered with at least 'on more than half of the days'[43].

For peer review only - http://bmjopen.bmj.com/site/about/guidelines.xhtml

Co-morbidity factors

Blood pressure (BP) was measured three times using a validated semi-automated device (The Microlife WatchBP home) with appropriate cuffs in a sitting position after at least 5min rest. The mean of the last two BP measurements was used in the analyses. Hypertension was defined as systolic BP 140mmHg and/or diastolic BP 90mmHg, and/or being on antihypertensive medication treatment, and/or self-reported hypertension. Trained research assistants in all sites collected fasting venous blood samples according to standard operation procedures, and then temporarily stored at the local research location. The stored blood samples from the local research centres were transported to Berlin, Germany, according to standardized procedures, for biochemical analyses. This was done to avoid intra-laboratory variability. Fasting plasma glucose concentration was measured using an enzymatic method (hexokinase). We defined Type 2 diabetes according to the World Health Organization diagnostic criteria (fasting glucose 7.0mmol/L, and/or current use of medication prescribed to treat diabetes, and/or self-reported diabetes) [44]. We assessed concentration of total cholesterol using colorimetric test kits. All biochemical analyses were performed using an ABX Pentra 400 Chemistry Analyzer (ABX Pentra; Horiba 90 ABX, Germany). Hypercholesterolaemia was defined as total cholesterol level ≥6.22mmol/L. Serum creatinine concentration (in mol/L) was determined by a kinetic colorimetric spectrophotometric isotope dilution mass spectrometry calibration method (Roche Diagnostics).

Outcome: CKD prevalence

We asked participants to bring an early morning urine sample for the analyses of albuminuria and creatinine levels. Urinary albumin concentration (in µmol/L) was measured by an immunochemical turbidimetric method (Roche Diagnostics). Urinary creatinine concentration (in 1 mol/L) was measured by a kinetic spectrophotometric method (Roche Diagnostics). Extensive quality checks were done inclusive of blinded serial measurements. Estimated glomerular filtration rate (eGFR) was calculated using the CKDEPI (CKD Epidemiology Collaboration) creatinine equation [45]. Urinary albumin–creatinine ratio (ACR; expressed in mg/mmol) was calculated by taking the ratio between urinary albumin and urinary creatinine. eGFR and albuminuria were categorized according to the 2012 KDIGO classification [46]. eGFR was categorized as follows: G1, 90mL/min/1.73m^2 (normal kidney function); G2, 60–89mL/min/1.73m^2 (mildly decreased); G3a, 45–59mL/min/1.73m^2 (mildly to moderatelydecreased); G3b, 30–44mL/min/1.73m^2 (moderately to severely decreased); G4, 15–29 mL/min/1.73 m^2 (severely decreased); and G5, <15mL/min/1.73m^2 (kidney failure). Albuminuria categories were derived from ACR and were as follows: A1, < 3mg/mmol (normal to mildly increased); A2, 3–30mg/mmol (moderately increased); and A3, > 30mg/mmol (severely increased). CKD status was categorized according to severity of kidney disease (green, low risk; yellow, moderately increased risk; orange, high risk; and red, very high risk) using the combination of eGFR (G1–

For peer review only

For peer review only - http://bmjopen.bmj.com/site/about/guidelines.xhtml

G5) and albuminuria (A1–A3) levels defined by the 2012 KDIGO guideline [47]. Due to the small number of participants in the very high-risk category of CKD (n=27), the high and very high-risk groups were combined. Because of the small number of participants in the severely increased albuminuria category (A3, n=62), we defined albuminuria as ACR 3mg/ mmol by combining the moderately increased (A2) and severely increased (A3) categories.

Covariates assessed were age, sex, educational level and length of stay in Europe, hypertension and diabetes. Length of stay was assessed for Ghanaian migrants only. Length of stay was defined as the number of years lived in Europe at the time of data collection. Length of stay was controlled for due to evidence suggesting that it influences mental health [48]. Other covariates were hypertension, obesity and diabetes.

Patient and Public Involvement

Community leaders were involved in the recruitment of patients. These comprised of religious communities (churches and mosques), endorsement from local key leaders and establishing relationships with healthcare organizations. We also provided information on the study by involving the local media (radio and television stations). We sent letters to all selected health and community authorities to notify participants of the study. Team members were sent to the various communities to stay among the community and organize mini clinics for a period of 1-2 weeks. Results of the study were disseminated through seminars, durbars and via radio and television stations.

Statistical methods

Characteristics of participants were expressed as absolute numbers and percentages for categorical variables and means and standard deviations for continuous variables. The z-test was used to compare proportions of demographic and clinical variables among the various sites and the independent t-test was also used to test for mean differences between the two sites. Odds Ratios (ORs) and their corresponding 95% confidence intervals (CIs) were estimated by means of binary logistic regression analyses to study the associations of albuminuria (ACR>3 mg/mmol, A2-A3, moderately to severely increased albuminuria), reduced kidney function (eGFR< 60 mL/min/1.73m^2, G3-G5 moderately to severely decreased kidney function) and increased CKD risk (high and very high CKD risk), with adjustments for covariates [49]. The Spearman's correlation test was used to test for associations between all four constructs of PS. Three models were used to examine the data. Model 1 was adjusted for age and sex, model 2 was adjusted for age and sex and educational level for Ghanaians living in SSA while age, sex, educational level and length of stay for Ghanaians living in Europe [50-52]. Model 3 was adjusted for sex, age, educational level and conventional risk factors (hypertension, diabetes, hypercholesterolemia, BMI, physical activity and smoking status) of CKD.

For peer review only

The analyses were performed for all 4 constructs of PS using individuals who have not experienced either of the PS per outcome as reference. All tests were stratified per sites due to interactions, Ghanaians living in SSA and Europe; Ghanaians living in rural and urban Ghana and Ghanaians living in Amsterdam, Berlin and London due to an observed interaction between PS and site. Furthermore, the analyses were stratified for those with and without obesity, diabetes, hypertension across all sites. P-values less than 0.05 were interpreted as statistically significant. All analyses were performed using STATA, version 14.0 (StataCorp LP).

Results

Characteristics of the study population

Participants characteristics are shown in Table 1. Ghanaians living in Ghana were significantly older than their peers living in Europe (47.7±11.9 versus 46.6±9.9, p=0.006). There were more female participants in the Ghana sample compared with European sample (67.1% versus 58.5%, p=0.001). Ghanaians living in Ghana were significantly less educated than those living in Europe. Higher proportion of Ghanaians living in Ghana had experienced negative life events in the last 12 months compared with their peers living in Europe (68.7% versus 59.0%, p=0.001). More than half of Ghanaians living in Ghana had experienced some stress at home or work whereas only a third of those living in Europe had experienced some stress at home or work (p=0.001). Permanent stress at home/work was fairly the same among Ghanaians living in SSA and Europe. Perceived discrimination was significantly higher among Ghanaians living in Europe compared with their peers living in Ghana (29.7% versus 4.8%, p=0.001). Depressive symptoms were more prevalent among Ghanaians living in Europe 7.5% compared with their peers living in Ghana 5.1%. Almost all Ghanaians living in Europe were first generation migrants. Ghanaians in Europe were significantly more obese, more likely to smoke and less physically active compared with their peers living in Ghana. Prevalence of hypercholesterolemia was significantly higher, but type 2 diabetes and hypertension were significantly lower among Ghanaians living in Ghana compared with their peers living in Europe (p=0.001). Prevalence of albuminuria, reduced eGRF and CKD risk were higher in Ghanaians living in Ghana compared with those living in Europe.

For peer review only - http://bmjopen.bmj.com/site/about/guidelines.xhtml

Table 1: Baseline characteristics of respondents

	Ghanaians (SSA) n (%)	Ghanaians (Europe) n (%)	p-value
**N**	2,492 (44.1)	3,167 (55.9)	0.001
**Female sex**	1672 (67.1)	1,851 (58.5)	0.001
**Age (years)**	47.7±11.9	46.6±9.9	0.006
**Educational status**			
Low	1169 (49.2)	635 (21.8)	0.001
Middle	858 (36.1)	1111 (38.1)	
High	347 (14.6)	1168 (40.1)	
**Negative life events in the past 12 months**			
No	739 (31.3)	1158 (41.0)	0.001
Yes	1619 (68.7)	1667 (59.0)	
**Perceived stress at home/work**			
Never	692 (29.4)	1371 (48.8)	0.001
Some periods	1290 (54.7)	1033 (36.8)	
Several/Permanent	375 (15.9)	407 (14.4)	
**Perceived discrimination**			
**No**	2065 (95.2)	1960 (70.3)	0.001
**Yes**	104 (4.8)	829 (29.7)	
**Depressive symptoms**			
**No**	2239 (94.9)	2582 (92.5)	0.001
**Yes**	119 (5.1)	209 (7.5)	
**Migration generation**			
First	Not applicable	2868 (98.7)	Not applicable
Second	Not applicable	38 (1.3)	Not applicable
**BMI**			
Normal (<25kg/m2)	1373 (55.2)	643 (20.4)	0.001
Overweight (25 ≤ 30kg/m2)	684 (27.5)	1,350 (42.8)	

For peer review only - http://bmjopen.bmj.com/site/about/guidelines.xhtml

Obese (>30kg/m2)	432 (17.3)	1163 (36.8)	
Currently smoking	36 (1.5)	121 (4.1)	0.001
**Physical activity**	1255 (52.8)	1131 (44.0)	0.001
**Hypocholesteraemia**	352 (14.2)	354 (11.3)	0.007
**Type 2 diabetes**	206 (8.3)	444 (14.0)	0.001
**Hypertension**	837 (33.6)	1801 (56.9)	0.001
**ACR**			
A1 < 3mg/mmol	2215 (90.2)	2814 (91.8)	0.001
A2-A ≥ 3mg/mmol	243 (9.8)	252 (8.2)	
**eGFR**			
G1-G2 ≥ 60 mL/min/1.73 m2	2377 (96.3)	2936 (97.4)	0.018
G3a-G5 < 60 mL/min/1.73 m2	85 (3.7)	78 (2.6)	
**CKD Risk**			
Low risk	2197 (89.6)	2705 (91.5)	0.015
Moderate-very high risk	256 (10.4)	252 (8.5)	

Abbreviations: N, number of respondents; BMI, body mass index; eGFR, estimated glomerular filtration rate; G1-G2, normal to high kidney function to mildly decreased; G3a-G5, mildly to moderately decreased to Kidney failure; ACR, albumin creatinine ratio; A1, normal to mildly increased; A2-A3, moderately increased to severely increased; CKD, Chronic kidney disease; SSA, Sub-Saharan Africa; CI, confidence intervals.

Association between PS and CKD

Figure 1-4 shows CKD prevalence by negative life events in the past 12 months among Ghanaians living in Ghana and Europe. In Europe, CKD prevalence was fairly the same among those who had experienced any negative life events compared with those who had not in the last 12 months. Prevalence of CKD was higher among Ghanaians who had not experienced any negative life events in the past 12 months (10.9%) compared with those who had experienced some negative life events (9.9%) and living in Ghana. CKD prevalence was higher among Ghanaians who had not experienced any form of discrimination (10.6%) than those who had (6.7%) in Ghana as well as in Europe (Figure 2). CKD prevalence was slightly higher among Ghanaians who had experienced several/permanent stress at work/home in the past 12 months and living in Ghana (10.4%) or Europe (9.7%) (Figure 3). Ghanaians who did not report any form of depressive symptoms had a significantly higher CKD prevalence than those who did and living in Ghana (10.4%) and Europe (8.7**%)** (Figure 4).

For peer review only

For peer review only - http://bmjopen.bmj.com/site/about/guidelines.xhtml

Table 2 shows the correlation matrix between the 4 constructs of PS among Ghanaians living in Ghana and those living in Europe. All four constructs of PS were positively correlated with each other among Ghanaians living in Europe and Ghanaians living in Ghana ($p<0.001$), except stress at work/home and discrimination among Ghanaians living in Ghana.

Table 2: Relationship between PS constructs (negative life events, discrimination, stress at work or home and depression) by Ghanaians (SSA) and Ghanaians (Europe)

Correlation matrix	Negative events	Discrimination	Stress at work/home	Depression
**Europe**				
Negative events	1.000			
Discrimination	0.152**	1.000		
Stress at work/home	0.297**	0.161**	1.000	
Depressive symptoms	0.143**	0.136**	0.285**	1.000
**Ghana**				
Negative events	1.000			
Discrimination	0.079**	1.000		
Stress at work/home	0.101**	-0.032	1.000	
Depressive symptoms	0.091**	0.042	0.185**	1.000

****Significant at 1%, Spearman's correlation**

Table 3 shows association between all 4 constructs of PS and CKD among Ghanaians living in Ghana and those living in Europe. There was no statistically significant association between PS and albuminuria, reduced eGFR and CKD risk among Ghanaians living in Ghana and those living in Europe except individuals living in Europe with some stress and lower risk of reduced eGFR (0.46, 95% C.I. 0.24-0.88). Table S1 shows further adjustments for conventional risk factors of CKD. This did not show any statistically significant associations between PS and albuminuria, reduced eGFR and CKD risk among Ghanaians living in Ghana and Europe (Table S1). Table S2 shows further stratification based on obesity status. We did not find any association between PS and CKD for obese participants and those who were not obese for Ghanaians living in rural and urban Ghana and those living in Europe. However, we observed an inverse association between PS and CKD among migrants who were not obese but have experienced discrimination for the past 12 months (0.63 95% C.I. 0.41-0.97) (Table S2). In Table S3 we stratified our analysis based on diabetic status. We did not find any associations between PS and

For peer review only - http://bmjopen.bmj.com/site/about/guidelines.xhtml

CKD for participants with and without diabetes for both Ghanaians living in rural and urban Ghana and their migrant peers in Europe (Table S3). Finally, Table S4 stratified analysis by hypertension status. No associations were observed between PS and CKD for individuals who had hypertension and those who did not have hypertension for both those living in rural and urban Ghana as well as their compatriots living in Europe. An inverse association was observed between PS and CKD among Ghanaians who have experienced discrimination in the last 12 months with no hypertension and living in Europe (0.51, 95% C.I. 0.27-0.97). Also, we observed that having experienced some stress at home/work was inversely associated with reduced eGFR among Ghanaians with hypertension and living in Europe (0.47, 95% C.I. 0. 0.23-0.95) (Table S4).

For peer review only

For peer review only - http://bmjopen.bmj.com/site/about/guidelines.xhtml

Table 3: **Association of psychosocial stressors (PS) indicators (negative life events, discrimination, stress at work/home and depression) with albuminuria, reduced eGFR and CKD risk for Ghanaians living in Ghana and those living in Europe**

	Albuminuria (ACR ≥ 3 mg/mmol)			eGFR < 60 mL/min/1.73 m2			High to very high CKD risk (KDIGO, 2012)		
		OR (95% CI)			OR (95% CI)			OR (95% CI)	
	n (%)	Model 1	Model 2	n (%)	Model 1	Model 2	n cases (%)	Model 1	Model 2
**Negative events**									
**Europe**									
No	1128 (8.2)	1.00 (Reference)	1.00 (Reference)	1106 (2.6)	1.00 (Reference)	1.00 (Reference	1090 (8.5)	1.00 (Reference)	1.00 (Reference)
Yes	1615 (8.4)	1.03 (0.78-1.35)	1.07 (0.80-1.42)	1587 (2.5)	0.86 (0.53-1.42)	0.83 (0.49-1.39)	1557 (8.6)	0.97 (0.76-1.32)	0.99 (0.74-1.32)
**Ghana**									
No	732 (8.7)	1.00 (Reference)	1.00 (Reference)	736 (4.5)	1.00 (Reference)	1.00 (Reference)	732 (10.9)	1.00 (Reference)	1.00 (Reference)
Yes	1595 (3.8)	0.87 (0.65-1.16)	0.85 (0.63-1.14)	1601 (3.4)	0.69 (0.45-1.08)	0.67 (0.44-1.09)	1590 (9.9)	0.88 (0.66-1.17)	0.86 (0.64-1.15)
**Discrimination**									
**Europe**									
No	1899 (8.5)	1.00 (Reference)	1.00 (Reference)	1867 (2.6)	1.00 (Reference)	1.00 (Reference)	1832 (8.9)	1.00 (Reference)	1.00 (Reference)
Yes	810 (7.4)	0.87 (0.64-1.19)	0.92 (0.67-1.26)	791 (2.2)	0.83 (0.47-1.47)	0.84 (0.46-1.52)	782 (7.3)	0.82 (0.59-1.12)	0.84 (0.60-1.16)
**Ghana**									
No	2034 (10.0)	1.00 (Reference)	1.00 (Reference)	2047 (3.9)	1.00 (Reference)	1.00 (Reference)	2031 (10.6)	1.00 (Reference)	1.00 (Reference)
Yes	104 (7.7)	0.83 (0.39-1.73)	0.91 (0.67-1.24)	104 (1.9)	0.67 (0.15-2.85)	0.67 (0.16-2.84)	104 (6.7)	0.70 (0.32-1.55)	0.71 (0.32-1.55)
**Stress at home/work**									
**Europe**									
Never	1330 (8.2)	1.00 (Reference)	1.00 (Reference)	1305 (3.4)	1.00 (Reference)	1.00 (Reference)	1282 (8.5)	1.00 (Reference)	1.00 (Reference)
Some stress	1002 (7.9)	0.97 (0.72-1.31)	1.04 (0.76-1.42)	984 (1.4)	**0.47 (0.26-0.87)**	**0.46 (0.24-0.88)**	968 (8.6)	0.96 (0.71-1.30)	1.02 (0.74-1.39)
Several/Permanent stresses	397 (9.1)	1.11 (0.74-1.64)	1.153 (0.77-1.72)	390 (2.3)	0.73 (0.35-1.52)	0.76 (0.36-1.61)	383 (9.7)	1.13 (0.77-1.68)	1.19 (0.80-1.79)
**Ghana**									
Never	682 (10.3)	1.00 (Reference)	1.00 (Reference)	688 (3.3)	1.00 (Reference)	1.00 (Reference)	682 (9.9)	1.00 (Reference)	1.00 (Reference)

For peer review only - http://bmjopen.bmj.com/site/about/guidelines.xhtml

Some stress	1279 (9.5)	0.87 (0.64-1.19)	0.80 (0.59-1.11)	1279 (3.9)	1.06 (0.63-1.77)	1.11 (0.66-1.87)	1274 (10.3)	0.95 (0.69-1.30)	0.92 (0.67-1.26)
Several/Permanent stresses	365 (8.5)	0.75 (0.48-1.18)	0.68 (0.59-1.11)	369 (4.1)	1.13 (0.57-2.23)	1.22 (0.61-2.46)	365 (10.4)	0.96 (0.63-1.47)	0.92 (0.59-1.42)
**Depressive symptoms**									
**Europe**									
No	2505 (8.5)	1.00 (Reference)	1.00 (Reference)	2457 (2.7)	1.00 (Reference)	1.00 (Reference)	2416 (8.7)	1.00 (Reference)	1.00 (Reference)
Yes	206 (6.3)	0.71 (0.39-1.27)	0.76 (0.43-1.36)	202 (1.5)	0.63 (0.19-2.03)	0.68 (0.21-2.23)	199 (7.1)	0.78 (0.44-1.37)	0.83 (0.47-1.46)
**Ghana**									
No	2212 (9.9)	1.00 (Reference)	1.00 (Reference)	2222 (3.8)	1.00 (Reference)	1.00 (Reference)	2207 (10.4)	1.00 (Reference)	1.00 (Reference)
Yes	114 (5.3)	0.45 (0.19-1.03)	0.45 (0.19-1.01)	114 (2.6)	0.52 (0.16-1.72)	0.53 (0.17-1.74)	114 (7.9)	0.62 (0.30-1.25)	0.61 (0.30-1.24)

Model 1, adjusted for age and sex; Model 2, adjusted for age, sex, and educational level for Ghanaians (SSA) and length of stay for those in Europe; Abbreviations: CI, confidence interval; ACR, albumin creatinine ration; eGFR, estimated glomerular filtration rate; CKD, chronic kidney disease; OR, odds ratio, n= total number of Ghanaians living in Ghana and Europe among the various levels of PS constructs; %, proportion of individuals with CKD among the various levels of PS constructs in Ghana and Europe

For peer review only

481 Table 4 shows associations between all 4 constructs of PS and CKD stratified by Ghanaians living in urban and rural Ghana. There was no association between PS and albuminuria, reduced eGFR and CKD risk among Ghanaians living rural and urban Ghana.

For peer review only

For peer review only - http://bmjopen.bmj.com/site/about/guidelines.xhtml

Table 4: Association of psychosocial stressors (PS) indicators (negative life events, discrimination, stress at work/home and depressive symptoms) with albuminuria, reduced eGFR and CKD risk among rural and urban Ghana

	Albuminuria (ACR ≥ 3 mg/mmol)			eGFR < 60 mL/min/1.73 m2			High to very high CKD risk (KDIGO, 2012)		
		OR (95% CI)			OR (95% CI)			OR (95% CI)	
	n (%)	Model 1	Model 2	n (%)	Model 1	Model 2	n cases (%)	Model 1	Model 2
**Negative events**									
**Urban Ghana**									
No	477 (11.9)	1.00 (Reference)	1.00 (Reference)	477 (4.4)	1.00 (Reference)	1.00 (Reference	477 (12.2)	1.00 (Reference)	1.00 (Reference)
Yes	912 (10.5)	0.87 (0.61-1.23)	0.87 (0.61-1.24)	911 (3.4)	0.73 (0.41-1.31)	0.72 (0.40-1.29)	910 (10.8)	0.87 (0.61-1.24)	0.87 (0.61-1.25)
**Rural Ghana**									
No	255 (7.5)	1.00 (Reference)	1.00 (Reference)	259 (4.6)	1.00 (Reference)	1.00 (Reference)	255 (8.6)	1.00 (Reference)	1.00 (Reference)
Yes	683 (7.6)	0.97 (0.56-1.69)	0.94 (0.54-1.64)	690 (3.5)	0.63 (0.31-1.31)	0.66 (0.32-1.37)	680 (8.8)	0.93 (0.55-1.58)	0.92 (0.54-1.56)
**Discrimination**									
**Urban Ghana**									
No	1326 (11.1)	1.00 (Reference)	1.00 (Reference)	1326 (3.9)	1.00 (Reference)	1.00 (Reference)	1325 (11.4)	1.00 (Reference)	1.00 (Reference)
Yes	71 (8.5)	0.85 (0.36-2.00)	0.89 (0.37-2.11)	71 (2.8)	1.17 (0.27-2.09)	1.16 (0.27-2.06)	71 (7.1)	0.69 (0.27-1.77)	0.72 (0.28-1.83)
**Rural Ghana**									
No	708 (8.1)	1.00 (Reference)	1.00 (Reference)	721 (3.9)	1.00 (Reference)	1.00 (Reference)	706 (9.2)	1.00 (Reference)	1.00 (Reference)
Yes	33 (6.1)	0.79 (0.18-3.47)	0.84 (0.19-2.65)	33 (0.0)	***	***	33 (6.1)	0.75 (0.17-2.89)	0.83 (0.19-2.65)
**Stress at home/work**									
**Urban Ghana**									
Never	460 (10.9)	1.00 (Reference)	1.00 (Reference)	460 (3.3)	1.00 (Reference)	1.00 (Reference)	460 (10.2)	1.00 (Reference)	1.00 (Reference)
Some stress	732 (11.5)	1.04 (0.71-1.51)	0.91 (0.62-1.37)	730 (4.1)	1.27 (0.66-2.43)	1.30 (0.67-2.51)	730 (11.8)	1.13 (0.77-1.65)	1.04 (0.71-1.53)
Several/Permanent stresses	197 (9.6)	0.87 (0.50-1.52)	0.74 (0.42-1.02)	198 (3.5)	1.17 (0.46-2.84)	1.20 (0.47-3.09)	197 (11.7)	1.15 (0.68-1.71)	1.05 (0.61-1.81)

For peer review only - http://bmjopen.bmj.com/site/about/guidelines.xhtml

**Rural Ghana**									
Never	222 (9.0)	1.00 (Reference)	1.00 (Reference)	228 (3.5)	1.00 (Reference)	1.00 (Reference)	222 (9.5)	1.00 (Reference)	1.00 (Reference)
Some stress	547 (6.9)	0.69 (0.39-1.23)	0.68 (0.38-1.22)	549 (3.6)	0.88 (0.38-2.07)	0.92 (0.39-2.18)	544 (8.3)	0.74 (0.42-1.30)	0.75 (0.42-1.31)
Several/Permanent stresses	168 (7.1)	0.63 (0.30-1.37)	0.60 (0.28-1.29)	171 (4.7)	1.07(0.38-3.03)	1.21(0.43-3.46)	168 (8.9)	0.71 (0.34-1.50)	0.73 (0.35-1.50)
**Depressive symptoms**									
**Urban Ghana**									
No	1336 (11.3)	1.00 (Reference)	1.00 (Reference)	1335 (3.8)	1.00 (Reference)	1.00 (Reference)	1334 (11.5)	1.00 (Reference)	1.00 (Reference)
Yes	52 (3.9)	0.30 (0.07-1.25)	0.30 (0.07-1.27)	52 (1.9)	0.46 (0.06-2.50)	0.45 (0.06-2.13)	52 (5.8)	0.44 (0.14-1.45)	0.45 (0.14-1.48)
**Rural Ghana**									
No	876 (7.7)	1.00 (Reference)	1.00 (Reference)	887 (3.8)	1.00 (Reference)	1.00 (Reference)	873 (8.7)	1.00 (Reference)	1.00 (Reference)
Yes	62 (6.5)	0.67 (0.23-1.94)	0.67 (0.23-1.94)	62 (3.2)	0.58 (0.13-2.56)	0.61 (0.14-2.68)	62 (9.7)	0.82 (0.33-2.01)	0.85 (0.34-2.09)

Model 1, adjusted for age and sex; Model 2, adjusted for age, sex, and educational level; Abbreviations: CI, confidence interval; ACR, albumin creatinine ration; eGFR, estimated glomerular filtration rate; CKD, chronic kidney disease; OR, odds ratio, n= total number of Ghanaians living in urban and rural Ghana among the various levels of PS constructs; %, proportion of individuals with CKD among the various levels of PS constructs in rural and urban Ghana. ***; no case of CKD and therefore odds ratios were not calculated

For peer review only

Table 5 shows associations between all 4 constructs of PS and CKD stratified by Ghanaians living in Amsterdam, Berlin and London. There were no associations between PS and albuminuria, reduced eGFR and CKD risk among Ghanaians living in Europe except for positive association between stress at work/home and albuminuria (2.81, 95% C.I. 1.46-5.40) and CKD risk (2.78, 95% C.I. 1.43-5.43) among Ghanaians living in Berlin.

For peer review only

For peer review only - http://bmjopen.bmj.com/site/about/guidelines.xhtml

Table 5: **Association of psychosocial stressors (PS) indicators (negative life events, discrimination, stress at work/home and depressive symptoms) with albuminuria, reduced eGFR and CKD risk among Ghanaians in three European cities.**

	Albuminuria (ACR ≥ 3 mg/mmol)			eGFR < 60 mL/min/1.73 m2			High to very high CKD risk (KDIGO, 2012)		
		OR (95% CI)			OR (95% CI)			OR (95% CI)	
	n (%)	Model 1	Model 2	n (%)	Model 1	Model 2	n (%)	Model 1	Model 2
**Negative events**									
**Amsterdam**									
No	548 (7.3)	1.00 (Reference)	1.00 (Reference)	534 (2.4)	1.00 (Reference)	1.00 (Reference))	521 (7.5)	1.00 (Reference)	1.00 (Reference)
Yes	784 (7.8)	1.08 (0.71-1.63)	1.18 (0.77-1.81)	764 (2.9)	1.11 (0.55-2.23)	1.15 (0.55-2.37)	742 (8.0)	1.06 (0.69-1.62)	1.08 (0.71-1.66)
**Berlin**									
No	213 (9.9)	1.00 (Reference)	1.00 (Reference)	213 (2.4)	1.00 (Reference)	1.00 (Reference)	213 (10.8)	1.00 (Reference)	1.00 (Reference)
Yes	329 (10.9)	1.12 (0.63-1.99)	1.19 (0.67-2.15)	330 (1.8)	0.64 (0.19-2.17)	0.61 (0.18-2.11)	329 (9.4)	0.86 (0.48-1.52)	0.91 (0.51-1.63)
**London**									
No	367 (8.7)	1.00 (Reference)	1.00 (Reference)	359 (3.1)	1.00 (Reference)	1.00 (Reference)	356 (8.7)	1.00 (Reference)	1.00 (Reference)
Yes	502 (7.8)	0.89 (0.55-1.46)	0.83 (0.49-1.41)	493 (2.2)	0.68 (0.28-1.65)	0.58 (0.22-1.51)	486 (9.1)	1.04 (0.64-1.68)	0.99 (0.58-1.68)
**Discrimination**									
**Amsterdam**									
No	956 (8.3)	1.00 (Reference)	1.00 (Reference)	935 (2.9)	1.00 (Reference)	1.00 (Reference)	909 (8.5)	1.00 (Reference)	1.00 (Reference)
Yes	363 (5.0)	0.59 (0.34-1.00)	0.59 (0.35-1.02)	349 (2.1)	0.69 (0.30-1.62)	0.81 (0.34-1.91)	342 (5.9)	0.69 (0.41-1.14)	0.69 (0.41-1.16)
**Berlin**									
No	329(10.0)	1.00 (Reference)	1.00 (Reference)	329 (2.1)	1.00 (Reference)	1.00 (Reference)	329 (10.3)	1.00 (Reference)	1.00 (Reference)
Yes	209 (11.0)	1.11 (0.63-1.95)	1.16 (0.65-2.05)	210 (1.9)	0.83 (0.24-2.93)	0.82 (0.23-2.91)	209 (9.1)	0.86 (0.48-1.56)	0.89 (0.49-1.63)
**London**									
No	614 (7.9)	1.00 (Reference)	1.00 (Reference)	603 (2.5)	1.00 (Reference)	1.00 (Reference)	594 (8.9)	1.00 (Reference)	1.00 (Reference)
Yes	238 (7.9)	1.03 (0.59-1.81)	1.18 (0.65-2.15)	232 (2.6)	1.29 (0.46-3.59)	1.09 (0.35-3.43)	231 (7.8)	0.93 (0.53-1.63)	0.98 (0.52-1.82)
**Stress at home/work**									

For peer review only - http://bmjopen.bmj.com/site/about/guidelines.xhtml

**Amsterdam**									
Never	634 (8.4)	1.00 (Reference)	1.00 (Reference)	622 (3.2)	1.00 (Reference)	1.00 (Reference)	603 (8.0)	1.00 (Reference)	1.00 (Reference)
Some stress	478 (5.7)	0.68 (0.42-1.11)	0.69 (0.42-1.13)	462 (1.9)	0.64 (0.29-1.43)	0.68 (0.30-1.52)	452 (6.0)	0.74 (0.45-1.20)	0.74 (0.45-1.22)
Several/Permanent stresses	210 (9.1)	1.09 (0.63-1.91)	1.12 (0.64-1.95)	204 (2.5)	0.71 (0.26-1.95)	0.77 (0.28-2.14)	198 (10.1)	1.24 (0.71-2.14)	1.26 (0.73-2.20)
**Berlin**									
Never	250 (9.0)	1.00 (Reference)	1.00 (Reference)	250 (2.0)	1.00 (Reference)	1.00 (Reference)	250 (6.4)	1.00 (Reference)	1.00 (Reference)
Some stress	196 (15.3)	**2.50 (1.33-4.71)**	**2.81 (1.46-5.40)**	197 (1.5)	0.88 (0.20-3.79)	0.83 (0.19-3.62)	196 (14.8)	**2.57 (1.34-4.90)**	**2.78 (1.43-5.43)**
Several/Permanent stresses	96 (10.4)	1.64 (0.72-3.73)	1.69 (0.73-3.91)	197 (3.1)	2.10 (0.47-9.46)	2.04 (0.44-9.26)	76 (9.4)	1.52 (0.65-3.58)	1.58 (0.66-3.75)
**London**									
Never	446 (9.2)	1.00 (Reference)	1.00 (Reference)	433 (4.4)	1.00 (Reference)	1.00 (Reference)	429 (10.5)	1.00 (Reference)	1.00 (Reference)
Some stress	328 (7.0)	0.73 (0.43-1.25)	0.79 (0.44-1.40)	325 (0.6)	0.17 (0.04-0.73)	0.09 (0.01-0.67)	320 (6.9)	0.65 (0.38-1.10)	0.66 (0.37-1.19)
Several/permanent stresses	91 (7.7)	0.81 (0.35-1.87)	0.86 (0.35-2.14)	90 (1.1)	0.27 (0.03-2.12)	0.24 (0.03-2.05)	74 (8.9)	0.83 (0.38-1.83)	0.92 (0.39-2.16)
**Depressive symptoms**									
**Amsterdam**									
No	1199 (7.8)	1.00 (Reference)	1.00 (Reference)	1135 (2.8)	1.00 (Reference)	1.00 (Reference)	1135 (7.9)	1.00 (Reference)	1.00 (Reference)
Yes	121 (6.6)	0.81 (0.39-1.72)	0.83 (0.39-1.76)	118 (1.7)	0.65 (0.15-2.77)	0.71 (0.16-3.06)	116 (6.9)	0.83 (0.39-1.76)	0.82 (0.38-1.74)
**Berlin**									
No	503 (10.7)	1.00 (Reference)	1.00 (Reference)	504 (2.2)	1.00 (Reference)	1.00 (Reference)	503 (10.1)	1.00 (Reference)	1.00 (Reference)
Yes	34 (5.9)	0.53 (0.12-2.27)	0.49 (90.11-2.13)	34 (0.0)	***	***	34 (5.9)	0.55 (0.13-2.37)	0.52 (0.12-2.24)
**London**									
No	803 (8.3)	1.00 (Reference)	1.00 (Reference)	785 (2.6)	1.00 (Reference)	1.00 (Reference)	778 (8.9)	1.00 (Reference)	1.00 (Reference)
Yes	51 (5.9)	0.67 (0.20-2.21)	0.91 (0.27-3.07)	50 (2.0)	0.91 (0.11-7.43)	1.15 (0.14-9.54)	49 (8.2)	0.94 (0.33-2.69)	1.30 (0.44-3.81)

Model 1, adjusted for age and sex; Model 2, adjusted for age, sex, educational level and length of stay; Abbreviations: CI, confidence interval; ACR, albumin creatinine ration; eGFR, estimated glomerular filtration rate; CKD, chronic kidney disease; OR, odds ratio, n= total number of Ghanaians living in Amsterdam, Berlin and London among the various levels of PS constructs; %, proportion of individuals with CKD among the various levels of PS constructs in Europe ***; no case of CKD and therefore odds ratios were not calculated.

For peer review only

Discussion

Key findings

Whereas there was an association between those who have experienced some stress at home/work and reduced eGFR among Ghanaians living in Europe, we did not find any association between PS and CKD among Ghanaians living in rural and urban Ghana and their peers in Europe. Also, PS was not associated with CKD for those living in rural and urban Ghana and neither for those living in the three European cities. However, there was an association between stress at work/home and albuminuria and CKD risk among Ghanaians living in Berlin. Further adjustment for conventional risk factors of CKD yielded similar results.

Discussion of key findings

Association between PS and CKD in Ghana

Our study did not find any association between any of the four constructs of PS and prevalence of CKD (albuminuria, reduced eGFR and CKD risk) among Ghanaians living in rural and urban Ghana. Our findings are however in contrast with earlier studies which reported positive associations between PS and prevalent of CKD [28 31 53]. Other studies have hypothesised that the influence of PS on CKD may be important in only those with hypertension and diabetes and that PS may mediate or moderate the association between renal functioning and lifestyle behaviours such as smoking and physical activity [33]. For example, they argue that stress enhances Sympathetic Nervous System (SNS) activity to increase glucocorticoid secretion and inflammatory cytokines, which heavily contribute to hypertension, diabetes and vascular disease, which are major risk factors of CKD incidence and prevalence [54]. The lack of association between PS and CKD in this present study is unclear due to lack of literature on the association between PS and CKD prevalence particularly in rural and urban populations. Worth noting, however, is the presence of rich family support systems in the Ghanaian context especially in rural Ghana, which may help individuals with CKD to cope with PS thereby minimizing its effect. For example, patients with limited social networks and low social support have been shown to have augmented risk of morbidity and mortality [55-57]. Specifically, there is evidence that positive social support is a protective factor for persons dealing with long-term disease conditions [58]. Other studies have reported a protective relationship between social networks, emotionally supportive relationship and threats to physiological and psychosocial health [59].

Association between PS and CKD Amsterdam, Berlin and London

Literature on the association between PS and CKD prevalence among migrants is scant and absent in most European populations. The lack of positive association between PS and CKD in our study is consistent with

For peer review only - http://bmjopen.bmj.com/site/about/guidelines.xhtml

recent studies conducted among African Americans [31 33] and other populations [32 60]. Specifically, a recent study using data from the Jackson Heart Study, which comprised of extensive constructs of psychosocial variables reported that greater life stressors were associated with lower prevalence of CKD at baseline [31]. Several studies in other parts of the world have reported a positive relationship between higher prevalence of stressors and CKD risk [27 28], although the study findings have been inconsistent. Whereas some did not find any associations among African Americans [31] others found associations in other populations. Even among those who found some associations the directions differed [28]. Reasons for the lack of association observed in our study among migrants are not fully understood but may reflect the real world situation. First, migrants from Ghana practice both nuclear and extended family support system as their peers living in rural and urban, this practice may mitigate the impact of stressors such as unemployment, death of a love one, discrimination, etc. They also belong to several religious organisations such as churches, which provide similar support systems against stressors. Moreover, there are several associations of the various ethnic groups (Akan, Ga and Ewe) providing such support when the need arises. These systems provide both instrumental and/or emotional social support [61]. These assertions are supported by several studies. Specifically, these studies have shown that social support positively affect health outcomes through mechanisms such as increased patient compliance with therapies, decreased levels of depressive affect, direct physiologic effects on the immune system and improved perception of quality of life [58 59]. The lack of association observed in this study may also be attributed to other mechanisms, which influence the associations between PS and CKD. Another reason for the lack of association between PS and CKD in this study could be the cross-sectional analysis of our study. The association between PS and CKD has been shown to be cumulative and builds over substantial period of time[62]. To effectively evaluate this, longitudinal study design is required. This suggests the needs for more longitudinal studies in future research in assessing the associations between PS and CKD outcomes [62 63].

Strength and limitation

Our study is the first to use all four robust constructs of PS to determine association between PS and CKD. This gave our study a more robust definition of PS compared to other similar studies. The use of all three definitions of CKD per KDIGO guidelines also provided a broader definition of CKD and allowed comparison between different geographical regions. The use of a homogenous population of Ghanaians and standardized protocols and diagnostic criteria in this study also provided a novel opportunity to compare Ghanaians living in rural and urban Ghana and their compatriots living in Europe. There are limitations to our study. The effect of PS on CKD has been reported to be cumulative and takes a long period of time, therefore the use of cross-sectional design prevented us from determining the longitudinal and cumulative

For peer review only - http://bmjopen.bmj.com/site/about/guidelines.xhtml

effect of repeated exposure to PS among the two populations. PS is captured and experienced in different magnitude across different populations. We were unable to ascertain if PS as defined in this study was adequately captured among Ghanaians living in rural and urban Ghana.

Conclusion

Generally, our study shows no associations between stress as indicated by four PS indicators and prevalence of CKD. Consequently, there is the need to explore other factors that may be responsible for the observed differences in the prevalence of CKD among Ghanaians living in rural and urban Ghana and their peers living in Europe.

Acknowledgement

The authors are very grateful to the research assistants, interviewers and other staff of the five research locations who took part in gathering the data and the Ghanaian volunteers in all the participating RODAM sites. We gratefully acknowledge the advisory board members for their valuable support in shaping the RODAM study methods and the Academic Medical Centre Biobank for their support in biobank management and high-quality storage of collected samples.

Contributors

My co-authors have all contributed substantially to this manuscript and approve of this submission. Research idea and study design: DNA, CA, KS, DA, EB, KM, JA; data acquisition and curation: DNA, CA, EB, KM, data analysis/interpretation: DNA, CA, KS, DA, EB, KM, LS, JA, EOD, KKG, FPM, MBS, ID, JS, SB; statistical analysis: DNA, CA, KS. DNA, CA, KS, DA, EB, KM, LS, JA, EOD, KKG, FPM, MBS, ID, JS, SB; contributed important intellectual content during manuscript drafting or revision and accepts accountability for the overall work by ensuring that questions pertaining to the accuracy or integrity of any portion of the work are appropriately investigated and resolved. DNA and CA takes responsibility that this study has been reported honestly, accurately, and transparently; that no important aspects of the study have been omitted; and that any discrepancies from the study as planned have been explained.

Funding

This work was supported by the European Commission under the Framework Programme (Grant Number: 278901). The funders had no role in study design, data collection and analysis, decision to publish, or

For peer review only

preparation of the manuscript. The Wellcome Trust supported Professor Smeeth's contribution, grant number WT082178. Professor Joachim Spranger was supported by the DZHK (German Center for cardiovascular research) and the Berlin Institute of Health (BIH).

Competing interest: I have communicated with all my co-authors and obtained their full disclosures. My co-authors and I declare no conflicts of interest.

Patient Consent: None declared

Ethics approval: IRBs at each participating site.

Data sharing statement: Data are available from the RODAM research cohort, a third party. Dr. Eric Beune affiliated with the RODAM research cohort and a co-author of this paper in accordance with the RODAM requirements for collaboration. Dr. Beune is the Data Collection Coordinator of RODAM and may be contacted with further questions (e.j.beune@amc.uva.nl). Additionally, researchers interested in further collaboration with RODAM may see the following URL: http://www.rod-am.eu/

For peer review only

For peer review only - http://bmjopen.bmj.com/site/about/guidelines.xhtml

References

1. Weisbord SD. Symptoms and their correlates in chronic kidney disease. *Adv Chronic Kidney Dis* 2007;14(4):319-27.
2. Correa-Rotter R, Naicker S, Katz IJ, et al. Demographic and epidemiologic transition in the developing world: role of albuminuria in the early diagnosis and prevention of renal and cardiovascular disease. *Kidney Int* 2004;66:S32-S37.
3. Lozano R, Naghavi M, Foreman K, et al. Global and regional mortality from 235 causes of death for 20 age groups in 1990 and 2010: a systematic analysis for the Global Burden of Disease Study 2010. *The lancet* 2012;380(9859):2095-128.
4. Katz IJ, Gerntholtz T, Naicker S. Africa and nephrology: the forgotten continent. *Nephron Clinical Practice* 2011;117(4):320-27.
5. Bruce MA, Beech BM, Sims M, et al. Social environmental stressors, psychological factors, and kidney disease. *J Investig Med* 2009;57(4):583-89.
6. Osafo C, Mate-Kole M, Affram K, et al. Prevalence of chronic kidney disease in hypertensive patients in Ghana. *Ren Fail* 2011;33(4):388-92.
7. Ojo A. Addressing the global burden of chronic kidney disease through clinical and translational research. *Trans Am Clin Climatol Assoc* 2014;125:229.
8. Norris K, Nissenson AR. Race, gender, and socioeconomic disparities in CKD in the United States. *J Am Soc Nephrol* 2008;19(7):1261-70.
9. Volkova N, McClellan W, Klein M, et al. Neighborhood poverty and racial differences in ESRD incidence. *J Am Soc Nephrol* 2008;19(2):356-64.
10. Shoham DA, Vupputuri S, Roux AVD, et al. Kidney disease in life-course socioeconomic context: the Atherosclerosis Risk in Communities (ARIC) Study. *Am J Kidney Dis* 2007;49(2):217-26.
11. Tarver-Carr ME, Powe NR, Eberhardt MS, et al. Excess risk of chronic kidney disease among African-American versus white subjects in the United States: a population-based study of potential explanatory factors. *J Am Soc Nephrol* 2002;13(9):2363-70.
12. Adjei DN, Stronks K, Adu D, et al. Chronic kidney disease burden among African migrants in three European countries and in urban and rural Ghana: the RODAM cross-sectional study. *Nephrology Dialysis Transplantation* 2018
13. Adjei DN, Stronks K, Adu D, et al. Relationship between educational and occupational levels, and Chronic Kidney Disease in a multi-ethnic sample-The HELIUS study. *PLoS One* 2017;12(11):e0186460.
14. Fremont A, Bird CE. Social and psychological factors, physiological processes, and physical health. 2000
15. Krantz DS, McCeney MK. Effects of psychological and social factors on organic disease: a critical assessment of research on coronary heart disease. *Annu Rev Psychol* 2002;53(1):341-69.
16. Cukor D, Fruchter Y, Ver Halen N, et al. A preliminary investigation of depression and kidney functioning in patients with chronic kidney disease. *Nephron Clinical practice* 2012;122(3-4):139-45.
17. Davidson K, Jonas BS, Dixon KE, et al. Do depression symptoms predict early hypertension incidence in young adults in the CARDIA study? *Arch Intern Med* 2000;160(10):1495-500.

For peer review only - http://bmjopen.bmj.com/site/about/guidelines.xhtml

18. Amerena J, Julius S. The role of the autonomic nervous system in hypertension. *Hypertens Res* 1995;18(2):99-110.
19. Calhoun DA, Mutinga ML. Race, family history of hypertension, and sympathetic response to cold pressor testing. *Blood Press* 1997;6(4):209-13.
20. Dibona GF. Neural control of the kidney: past, present, and future. *Hypertension* 2003;41(3):621-24.
21. Auchincloss AH, Diez Roux AV, Brown DG, et al. Association of insulin resistance with distance to wealthy areas: the multi-ethnic study of atherosclerosis. *Am J Epidemiol* 2006;165(4):389-97.
22. Bruce MA, Sims M, Miller S, et al. One size fits all? Race, gender and body mass index among US adults. *J Natl Med Assoc* 2007;99(10):1152.
23. McEwen BS. Protective and damaging effects of stress mediators. *N Engl J Med* 1998;338(3):171-79.
24. Cohen S, Herbert TB. Health psychology: Psychological factors and physical disease from the perspective of human psychoneuroimmunology. *Annu Rev Psychol* 1996;47(1):113-42.
25. Barker D. Fetal origins of coronary heart disease. *Br Heart J* 1993;69(3):195.
26. Phillips DI, Jones A, Goulden PA. Birth weight, stress, and the metabolic syndrome in adult life. *Ann N Y Acad Sci* 2006;1083(1):28-36.
27. Kim JW, Moon SJ, Kim HJ, et al. Relationship between Chronic Kidney Disease and Depression in Elderly Koreans Using the 2013 Korea National Health and Nutrition Examination Survey Data. *Korean journal of family medicine* 2017;38(3):156-62.
28. Loosman WL, Rottier MA, Honig A, et al. Association of depressive and anxiety symptoms with adverse events in Dutch chronic kidney disease patients: a prospective cohort study. *BMC Nephrol* 2015;16(1):155.
29. Novak M, Mucsi I, Mendelssohn D. Screening for depression: only one piece of the puzzle. *Nephrology Dialysis Transplantation* 2013;28(6):1336-40.
30. McKercher CM, Venn AJ, Blizzard L, et al. Psychosocial factors in adults with chronic kidney disease: characteristics of pilot participants in the Tasmanian Chronic Kidney Disease study. *BMC Nephrol* 2013;14(1):83.
31. Lunyera J, Davenport CA, Bhavsar NA, et al. Nondepressive Psychosocial Factors and CKD Outcomes in Black Americans. *Clin J Am Soc Nephrol* 2018:CJN. 06430617.
32. Annor FB, Masyn KE, Okosun IS, et al. Psychosocial stress and changes in estimated glomerular filtration rate among adults with diabetes mellitus. *Kidney research and clinical practice* 2015;34(3):146-53.
33. Gholson GK, Mwendwa DT, Wright RS, et al. The Combined Influence of Psychological Factors on Biomarkers of Renal Functioning in African Americans. *Ethn Dis* 2015;25(2):117.
34. Kaur M, Kaur A, Saggu A, et al. Comparative study on psychosocial stresses among urban and rural geriatric population in selected areas of district Ludhiana (Punjab), 2006. *Nursing and Midwifery Research* 2007;3(2)
35. Agyemang C, Beune E, Meeks K, et al. Rationale and cross-sectional study design of the Research on Obesity and type 2 Diabetes among African Migrants: the RODAM study. *BMJ open* 2015;4(3):e004877.
36. Agyemang C, Snijder MB, Adjei DN, et al. Ethnic Disparities in CKD in the Netherlands: The Healthy Life in an Urban Setting (HELIUS) Study. *Am J Kidney Dis* 2016;67(3):391-99.

For peer review only

For peer review only - http://bmjopen.bmj.com/site/about/guidelines.xhtml

37. Peek ME, Nunez-Smith M, Drum M, et al. Adapting the everyday discrimination scale to medical settings: reliability and validity testing in a sample of African American patients. *Ethn Dis* 2011;21(4):502.
38. Pérez DJ, Fortuna L, Alegria M. Prevalence and correlates of everyday discrimination among US Latinos. *J Community Psychol* 2008;36(4):421-33.
39. Ikram UZ, Snijder MB, Fassaert TJ, et al. The contribution of perceived ethnic discrimination to the prevalence of depression. *The European Journal of Public Health* 2014;25(2):243-48.
40. Rosengren A, Hawken S, Ôunpuu S, et al. Association of psychosocial risk factors with risk of acute myocardial infarction in 11 119 cases and 13 648 controls from 52 countries (the INTERHEART study): case-control study. *The Lancet* 2004;364(9438):953-62.
41. Rosmalen J, Bos E, De Jonge P. Validation of the Long-term Difficulties Inventory (LDI) and the List of Threatening Experiences (LTE) as measures of stress in epidemiological population-based cohort studies. *Psychol Med* 2012;42(12):2599-608.
42. Brugha T, Bebbington P, Tennant C, et al. The List of Threatening Experiences: a subset of 12 life event categories with considerable long-term contextual threat. *Psychol Med* 1985;15(1):189-94.
43. Kroenke K, Spitzer RL, Williams JB. The phq-9. *J Gen Intern Med* 2001;16(9):606-13.
44. Association AD. Standards of Medical Care in Diabetes—2014. Diabetes Care 2014; 37 (Suppl. 1): S14–S80Diagnosis and Classification of Diabetes Mellitus. Diabetes Care 2014; 37 (Suppl. 1): S81–S90. *Diabetes Care* 2014;37(3):887-87.
45. Stevens LA, Schmid CH, Greene T, et al. Comparative performance of the CKD Epidemiology Collaboration (CKD-EPI) and the Modification of Diet in Renal Disease (MDRD) Study equations for estimating GFR levels above 60 mL/min/1.73 m2. *Am J Kidney Dis* 2010;56(3):486-95.
46. Kellum JA, Lameire N, Aspelin P, et al. Kidney disease: improving global outcomes (KDIGO) acute kidney injury work group. KDIGO clinical practice guideline for acute kidney injury. *Kidney international supplements* 2012;2(1):1-138.
47. KDIGO G. Work Group. KDIGO clinical practice guideline for glomerulonephritis. *Kidney inter, Suppl* 2012;2:139-274.
48. Rivera B, Casal B, Currais L. Length of stay and mental health of the immigrant population in Spain: Evidence of the healthy immigrant effect. *Applied Economics* 2015;47(19):1972-82.
49. Collins AJ, Foley RN, Herzog C, et al. US Renal Data System 2010 Annual Data Report. *American journal of kidney diseases: the official journal of the National Kidney Foundation* 2011;57(1 Suppl 1):A8, e1.
50. Fox CS, Larson MG, Leip EP, et al. Predictors of new-onset kidney disease in a community-based population. *JAMA* 2004;291(7):844-50.
51. Banks J, Marmot M, Oldfield Z, et al. Disease and disadvantage in the United States and in England. *JAMA* 2006;295(17):2037-45.
52. Levin A, Stevens L, McCullough PA. Cardiovascular disease and the kidney: Tracking a killer in chronic kidney disease. *Postgrad Med* 2002;111(4):53-60.
53. Tsai Y-C, Chiu Y-W, Hung C-C, et al. Association of symptoms of depression with progression of CKD. *Am J Kidney Dis* 2012;60(1):54-61.

For peer review only - http://bmjopen.bmj.com/site/about/guidelines.xhtml

54. Bruce MA, Griffith DM, Thorpe RJ. Stress and the kidney. *Adv Chronic Kidney Dis* 2015;22(1):46-53.
55. Brummett BH, Barefoot JC, Siegler IC, et al. Characteristics of socially isolated patients with coronary artery disease who are at elevated risk for mortality. *Psychosom Med* 2001;63(2):267-72.
56. Eng PM, Rimm EB, Fitzmaurice G, et al. Social ties and change in social ties in relation to subsequent total and cause-specific mortality and coronary heart disease incidence in men. *Am J Epidemiol* 2002;155(8):700-09.
57. Psychosocial factors in patients with chronic kidney disease: The impact of social support on end-stage renal disease. Seminars in dialysis; 2005. Wiley Online Library.
58. Cohen SD, Sharma T, Acquaviva K, et al. Social support and chronic kidney disease: an update. *Adv Chronic Kidney Dis* 2007;14(4):335-44.
59. Uchino BN, Cacioppo JT, Kiecolt-Glaser JK. The relationship between social support and physiological processes: a review with emphasis on underlying mechanisms and implications for health. *Psychol Bull* 1996;119(3):488.
60. Tsurugano S, Nakao M, Takeuchi T, et al. Job stress strengthens the link between metabolic risk factors and renal dysfunction in adult men. *The Tohoku journal of experimental medicine* 2012;226(2):101-08.
61. Tróccol BT. Desenvolvimento de escala para avaliação do suporte social em HIV/aids. *Psicologia: Teoria e Pesquisa* 2006;22(3):317-26.
62. Schneiderman N, Ironson G, Siegel SD. Stress and health: psychological, behavioral, and biological determinants. *Annu Rev Clin Psychol* 2005;1:607-28.
63. McKercher C, Sanderson K, Jose MD. Psychosocial factors in people with chronic kidney disease prior to renal replacement therapy. *Nephrology* 2013;18(9):585-91.

For peer review only

For peer review only - http://bmjopen.bmj.com/site/about/guidelines.xhtml

Legend for figures

Figure 1: Prevalence of CKD risk among Ghanaians who have experienced negative life events and those who have not experienced any negative life events stratified by site. Definition per 2012 KDIGO (Kidney Disease: Improving Global Outcomes) guidelines.

Figure 2: Prevalence of CKD risk among Ghanaians who have experienced discrimination and those who have not experienced any discrimination stratified by site. Definitions per 2012 KDIGO (Kidney Disease: Improving Global Outcomes) guidelines.

Figure 3: Prevalence of CKD risk among Ghanaians who have experienced stress at home/work and those who have not experienced any stress at home/work in the past 12 months stratified by site. Definitions per 2012 KDIGO (Kidney Disease: Improving Global Outcomes) guidelines.

Figure 4: Prevalence of CKD risk among Ghanaians who have depressive symptoms and those who do not have depressive symptoms stratified by site. Definitions per 2012 KDIGO (Kidney Disease: Improving Global Outcomes) guidelines.

For peer review only

For peer review only - http://bmjopen.bmj.com/site/about/guidelines.xhtml

Legend for Supplementary Tables

S1: Association of psychosocial stressors (PS) indicators (negative life events, discrimination, stress at work/home and depression) with albuminuria, reduced eGFR and CKD risk for Ghanaians living in Ghana and those living in Europe.

S2: Association of psychosocial stressors (PS) indicators (negative life events, discrimination, stress at work/home and depression) with albuminuria, reduced eGFR and CKD risk for Ghanaians living in Ghana and those living in Europe stratified by obesity status.

S3: Association of psychosocial stressors (PS) indicators (negative life events, discrimination, stress at work/home and depression) with albuminuria, reduced eGFR and CKD risk for Ghanaians living in Ghana and those living in Europe stratified by diabetes status.

S4: Association of psychosocial stressors (PS) indicators (negative life events, discrimination, stress at work/home and depression) with albuminuria, reduced eGFR and CKD risk for Ghanaians living in Ghana and those living in Europe stratified by hypertensive status.

For peer review only

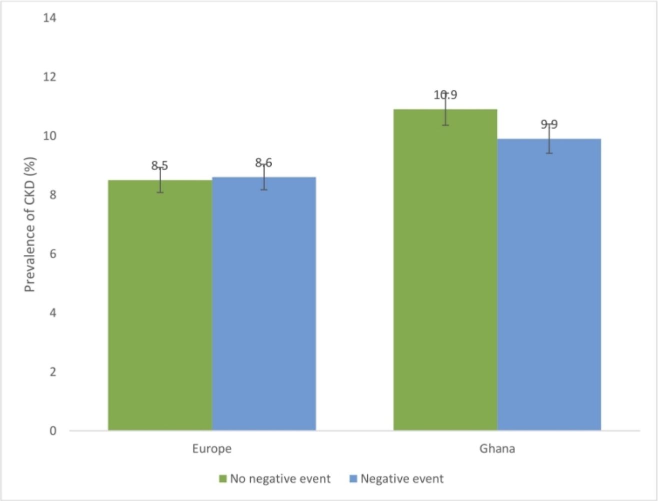

Figure 1: Prevalence of CKD risk among Ghanaians who have experienced negative life events and those who have not experienced any negative life events stratified by site. Definition per 2012 KDIGO (Kidney Disease: Improving Global Outcomes) guidelines.

Figure 1: Prevalence of CKD risk among Ghanaians who have experienced negative life events and those who have not experienced any negative life events stratified by site. Definition per 2012 KDIGO (Kidney Disease: Improving Global Outcomes) guidelines.

215x279mm (300 x 300 DPI)

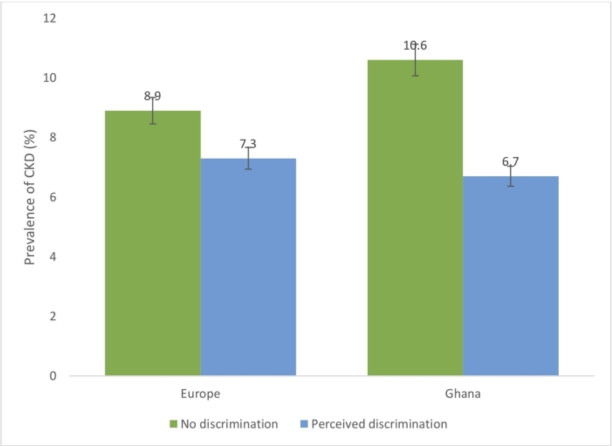

Figure 2: Prevalence of CKD risk among Ghanaians who have experienced discrimination and those who have not experienced any discrimination stratified by site. Definitions per 2012 KDIGO (Kidney Disease: Improving Global Outcomes) guidelines.

Figure 2: Prevalence of CKD risk among Ghanaians who have experienced discrimination and those who have not experienced any discrimination stratified by site. Definitions per 2012 KDIGO (Kidney Disease: Improving Global Outcomes) guidelines.

215x279mm (300 x 300 DPI)

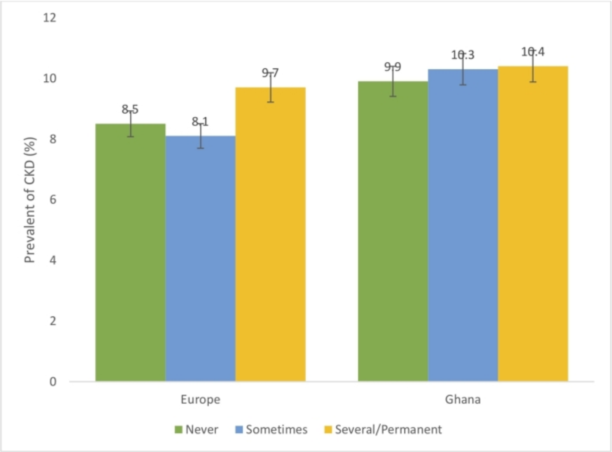

Figure 3: Prevalence of CKD risk among Ghanaians who have experienced stress at home/work and those who have not experienced any stress at home/work in the past 12 months stratified by site. Definitions per 2012 KDIGO (Kidney Disease: Improving Global Outcomes) guidelines.

Figure 3: Prevalence of CKD risk among Ghanaians who have experienced stress at home/work and those who have not experienced any stress at home/work in the past 12 months stratified by site. Definitions per 2012 KDIGO (Kidney Disease: Improving Global Outcomes) guidelines.

215x279mm (300 x 300 DPI)

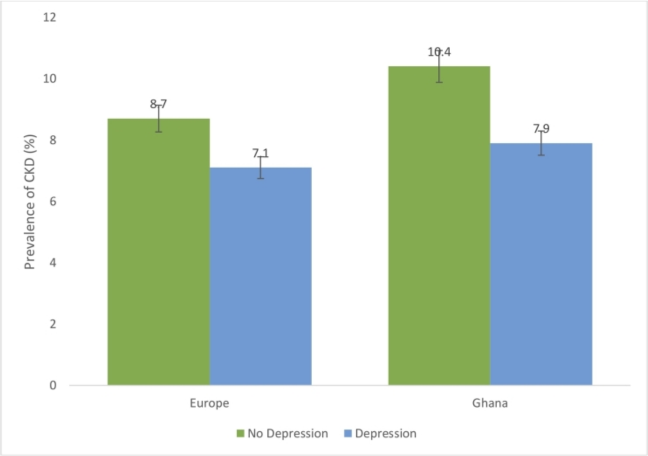

Figure 4: Prevalence of CKD risk among Ghanaians who have depressive symptoms and those who do not have depressive symptoms stratified by site. Definitions per 2012 KDIGO (Kidney Disease: Improving Global Outcomes) guidelines.

Figure 4: Prevalence of CKD risk among Ghanaians who have depressive symptoms and those who do not have depressive symptoms stratified by site. Definitions per 2012 KDIGO (Kidney Disease: Improving Global Outcomes) guidelines

215x279mm (300 x 300 DPI)

Supplementary Tables

S1: Association of psychosocial stressors (PS) indicators (negative life events, discrimination, stress at work/home and depression) with albuminuria, reduced eGFR and CKD risk for Ghanaians living in Ghana and those living in Europe

	Albuminuria (ACR ≥ 3 mg/mmol)		eGFR < 60 mL/min/1.73 m2		High to very high CKD risk (KDIGO, 2012)	
		OR (95% CI)		OR (95% CI)		OR (95% CI)
	n (%)	Model 3	n (%)	Model 3	n cases (%)	Model 3
**Negative events**						
**Europe**						
No	1128 (8.2)	1.00 (Reference)	1106 (2.6)	1.00 (Reference)	1090 (8.5)	1.00 (Reference)
Yes	1615 (8.4)	0.95 (0.70-1.30)	1587 (2.5)	0.77 (0.44-1.37)	1557 (8.6)	0.92 (0.67-1.26)
**Ghana**						
No	732 (10.4)	1.00 (Reference)	736 (4.5)	1.00 (Reference)	732 (10.9)	1.00 (Reference)
Yes	1595 (9.2)	0.79 (0.58-1.07)	1601 (3.4)	0.69 (0.43-1.09)	1590 (9.9)	0.79 (0.58-1.06)
**Discrimination**						
**Europe**						
No	1899 (8.5)	1.00 (Reference)	1867 (2.6)	1.00 (Reference)	1832 (8.9)	1.00 (Reference)
Yes	810 (7.4)	0.87 (0.61-1.21)	791 (2.2)	0.87 (0.45-1.65)	782 (7.3)	0.79 (0.55-1.12)
**Ghana**						
No	2034 (10.0)	1.00 (Reference)	2047 (3.9)	1.00 (Reference)	2031 (10.6)	1.00 (Reference)
Yes	104 (7.7)	0.92 (0.43-1.98)	104 (1.9)	0.65 (0.15-2.79)	104 (6.7)	0.74 (0.33-1.66)
**Stress at home/work**						
**Europe**						
Never	1330 (8.2)	1.00 (Reference)	1305 (3.4)	1.00 (Reference)	1282 (8.5)	1.00 (Reference)
Some stress	1002 (7.9)	1.01 (0.72-1.41)	984 (1.4)	0.51 (0.25-1.04)	968 (8.1)	1.01 (0.72-1.42)
Several/Permanent stresses	397 (9.1)	1.00 (0.64-1.59)	390 (2.3)	0.91 (0.40-2.03)	383 (9.7)	1.15 (0.74-1.79)
**Ghana**						

For peer review only - http://bmjopen.bmj.com/site/about/guidelines.xhtml

Never	682 (10.3)	1.00 (Reference)	688 (3.3)	1.00 (Reference)	682 (9.9)	1.00 (Reference)
Some stress	1279 (9.5)	0.78 (0.56-1.09)	1279 (3.9)	1.14 (0.67-1.93)	1274 (10.3)	0.93 (0.67-1.29)
Several/Permanent stresses	365 (8.5)	0.70 (0.43-1.13)	369 (4.1)	1.32 (0.65-1.68)	365 (10.4)	0.97 (0.62-1.52)
**Depressive symptoms**						
**Europe**						
No	2505 (8.5)	1.00 (Reference)	2457 (2.7)	1.00 (Reference)	2416 (8.7)	1.00 (Reference)
Yes	206 (6.3)	0.65 (0.33-1.28)	202 (1.5)	0.94 (0.28-3.14)	199 (7.1)	0.78 (0.41-1.47)
**Ghana**						
No	2212 (9.9)	1.00 (Reference)	2222 (3.8)	1.00 (Reference)	2207 (10.4)	1.00 (Reference)
Yes	114 (5.3)	0.41 (0.16-1.04)	114 (2.6)	0.58 (0.17-1.95)	114 (7.9)	0.62 (0.28-1.31)

Model 3, adjusted for age, sex educational level, hypertension, diabetes, hypercholesterolemia, BMI, physical activity and smoking; Abbreviations: CI, confidence interval; ACR, albumin creatinine ration; eGFR, estimated glomerular filtration rate; CKD, chronic kidney disease; OR, odds ratio, n= total number of Ghanaians living in Ghana and Europe among the various levels of PS constructs; %, proportion of individuals with CKD among the various levels of PS constructs in Ghana and Europe.

For peer review only

S2: Association of psychosocial stressors (PS) indicators (negative life events, discrimination, stress at work/home and depression) with albuminuria, reduced eGFR and CKD risk for Ghanaians living in Ghana and those living in Europe stratified by obesity status

	Albuminuria (ACR ≥ 3 mg/mmol)			eGFR < 60 mL/min/1.73 m2			High to very high CKD risk (KDIGO, 2012)		
		OR (95% CI)			OR (95% CI)			OR (95% CI)	
	n (%)	Model 1	Model 2	n (%)	Model 1	Model 2	n cases (%)	Model 1	Model 2
**Negative events**									
**Europe/Not obese**									
No	736 (7.4)	1.00 (Reference)	1.00 (Reference)	729 (2.5)	1.00 (Reference)	1.00 (Reference)	719 (8.5)	1.00 (Reference)	1.00 (Reference)
Yes	1003 (7.7)	0.98 (0.67-1.40)	0.99 (0.69-1.43)	992 (2.0)	0.77 (0.40-1.48)	0.71 (0.36-1.42)	971 (7.9)	0.91 (0.64-1.29)	0.89 (0.62-1.29)
**Europe/Obese**									
No	390 (9.2)	1.00 (Reference)	1.00 (Reference)	375 (2.9)	1.00 (Reference)	1.00 (Reference)	369 (8.7)	1.00 (Reference)	1.00 (Reference)
Yes	607 (9.6)	1.05 (0.67-1.63)	1.14 (0.72-1.82)	590 (3.2)	0.97 (0.44-2.11)	1.01 (0.44-2.26)	581 (9.6)	1.15 (0.73-1.82)	1.15 (0.71-1.85)
**Ghana/Not obese**									
No	588 (9.5)	1.00 (Reference)	1.00 (Reference)	592 (4.7)	1.00 (Reference)	1.00 (Reference)	588 (10.0)	1.00 (Reference)	1.00 (Reference)
Yes	1324 (8.5)	0.86 (0.61-1.21)	0.83 (0.59-1.17)	1331 (3.4)	0.64 (0.39-1.05)	0.65 (0.39-1.83)	1321 (9.0)	0.86 (0.61-1.20)	0.84 (0.60-1.18)
**Ghana/Obese**									
No	144 (13.9)	1.00 (Reference)	1.00 (Reference)	144 (3.5)	1.00 (Reference)	1.00 (Reference)	144 (14.6)	1.00 (Reference)	1.00 (Reference)
Yes	268 (13.1)	0.91 (0.50-1.64)	0.91 (0.49-1.65)	267 (3.8)	1.04 (0.34-3.16)	1.09 (0.35-3.37)	266 (14.3)	0.94 (0.52-1.68)	0.94 (0.52-1.67)
**Discrimination**									
**Europe/Not obese**									
No	1186 (8.4)	1.00 (Reference)	1.00 (Reference)	1172 (2.4)	1.00 (Reference)	1.00 (Reference)	1150 (9.4)	1.00 (Reference)	1.00 (Reference)
Yes	538 (5.9)	0.69 (0.46-1.05)	0.71 (0.46-1.11)	532 (1.5)	0.62 (0.28-1.37)	0.57 (0.24-1.34)	525 (5.9)	**0.63 (0.42-0.96)**	**0.63 (0.41-0.97)**
**Europe/Obese**									
No	709 (8.7)	1.00 (Reference)	1.00 (Reference)	691 (3.0)	1.00 (Reference)	1.00 (Reference)	678 (8.9)	1.00 (Reference)	1.00 (Reference)
Yes	269 (10.0)	1.14 (0.71-1.85)	1.29 (0.78-2.12)	256 (3.5)	1.15 (0.50-2.63)	1.27 (0.54-2.99)	254 (9.8)	1.13 (0.69-1.86)	1.22 (0.73-2.13)
**Ghana/Not obese**									
No	1648 (9.1)	1.00 (Reference)	1.00 (Reference)	1661 (3.9)	1.00 (Reference)	1.00 (Reference)	1646 (9.6)	1.00 (Reference)	1.00 (Reference)
Yes	81 (8.6)	1.06 (0.48-2.38)	1.04 (0.47-2.32)	81 (2.5)	1.04 (0.48-2.32)	0.86 (0.20-3.69)	81 (7.4)	0.90 (0.38-2.12)	0.89 (0.38-2.11)
**Ghana/Obese**									

For peer review only

No	383 (13.8)	1.00 (Reference)	1.00 (Reference)	383 (3.7)	1.00 (Reference)	1.00 (Reference)	382 (14.9)	1.00 (Reference)	1.00 (Reference)
Yes	23 (4.4)	0.31 (0.04-2.36)	0.40 (0.05-3.11)	23 (0.0)	**** (***-***)	**** (***-***)	23 (4.4)	0.29 (0.04-2.22)	0.34 (0.04-2.66)
**Stress at home/work**									
**Europe/Not obese**									
Never	855 (7.4)	1.00 (Reference)	1.00 (Reference)	847 (3.4)	1.00 (Reference)	1.00 (Reference)	831 (8.2)	1.00 (Reference)	1.00 (Reference)
Some stress	629 (8.3)	1.16 (0.79-1.70)	1.20 (0.81-1.79)	627 (1.4)	0.52 (0.24-1.12)	0.47 (0.21-1.07)	615 (7.9)	0.98 (0.66-1.43)	0.98 (0.65-1.46)
Several/Permanent stresses	247 (6.9)	0.93 (0.53-1.62)	1.15 (0.77-1.72)	239 (2.3)	0.45 (0.13-1.52)	0.49 (0.15-1.67)	236 (9.1)	0.97 (0.57-1.65)	1.04 (0.61-1.78)
**Europe/Obese**									
Never	472 (9.8)	1.00 (Reference)	1.00 (Reference)	455 (3.9)	1.00 (Reference)	1.00 (Reference)	448 (10.3)	1.00 (Reference)	1.00 (Reference)
Some stress	372 (7.5)	0.72 (0.44-1.18)	0.79 (0.47-1.32)	356 (1.4)	0.41 (0.15-1.16)	0.43 (0.15-1.21)	352 (9.5)	0.94 (0.57-1.53)	1.05 (0.62-1.76)
Several/Permanent stresses	147 (12.2)	1.32 (0.74-2.38)	1.31 (0.72-2.42)	148 (4.1)	1.05 (0.40-2.79)	1.09 (0.40-2.91)	144 (8.5)	1.36 (0.74-2.49)	1.41 (0.75-2.64)
**Ghana/Not obese**									
Never	537 (9.7)	1.00 (Reference)	1.00 (Reference)	542 (3.1)	1.00 (Reference)	1.00 (Reference)	537 (8.9)	1.00 (Reference)	1.00 (Reference)
Some stress	1055 (8.3)	0.79 (0.56-1.15)	0.74 (0.52-1.07)	1057 (3.9)	1.16 (0.65-2.09)	1.21 (0.67-2.18)	1052 (9.0)	0.92 (0.64-1.35)	0.90 (0.62-1.32)
Several/permanent stresses	319 (8.5)	0.81 (0.49-1.32)	0.73 (0.44-1.20)	323 (4.3)	1.34 (0.64-2.83)	1.43 (0.67-3.04)	319 (10.7)	1.12 (0.69-1.79)	1.09 (0.68-1.76)
**Ghana/Obese**									
Never	145 (12.4)	1.00 (Reference)	1.00 (Reference)	146 (4.1)	1.00 (Reference)	1.00 (Reference)	145 (13.8)	1.00 (Reference)	1.00 (Reference)
Some stress	221 (14.9)	1.21 (0.64-2.26)	1.05 (0.55-1.99)	219 (3.7)	0.74 (0.25-2.22)	0.83 (0.26-2.06)	219 (15.9)	1.13 (0.62-2.07)	1.02 (0.55-1.89)
Several/Permanent stresses	46 (8.7)	0.64 (0.20-2.01)	0.52 (0.16-1.65)	46 (2.2)	0.41 (0.05-3.61)	0.45 (0.05-4.01)	46 (8.8)	0.55 (0.17-1.71)	0.47 (0.14-1.50)
**Depressive symptoms**									
**Europe/Not obese**									
No	1601 (7.9)	1.00 (Reference)	1.00 (Reference)	1586 (2.2)	1.00 (Reference)	1.00 (Reference)	1558 (8.3)	1.00 (Reference)	1.00 (Reference)
Yes	121 (4.9)	0.59 (0.25-1.36)	0.65 (0.27-1.48)	117 (1.7)	0.79 (0.18-2.35)	0.86 (0.21-3.80)	115 (5.22)	0.58 (0.25-1.35)	0.64 (0.27-1.49)
**Europe/Obese**									
No	899 (9.7)	1.00 (Reference)	1.00 (Reference)	866 (3.4)	1.00 (Reference)	1.00 (Reference)	853 (9.4)	1.00 (Reference)	1.00 (Reference)
Yes	83 (7.2)	0.71 (0.29-1.67)	0.72 (0.30-1.74)	83 (1.2)	0.46 (0.06-3.52)	0.46 (0.06-3.57)	82 (8.5)	0.89 (0.40-2.01)	0.90 (0.40-2.04)

For peer review only - http://bmjopen.bmj.com/site/about/guidelines.xhtml

**Ghana/Not obese**									
No	1811 (8.9)	1.00 (Reference)	1.00 (Reference)	1822 (3.9)	1.00 (Reference)	1.00 (Reference)	1808 (9.4)	1.00 (Reference)	1.00 (Reference)
Yes	100 (6.0)	0.57 (0.24-1.33)	0.56 (0.24-1.32)	100 (2.0)	0.38 (0.09-1.63)	0.38 (0.09-1.63)	100 (8.0)	0.69 (0.32-1.46)	0.69 (0.32-1.47)
**Ghana/Obese**									
No	398 (13.8)	1.00 (Reference)	1.00 (Reference)	397 (3.5)	1.00 (Reference)	1.00 (Reference)	396 (14.7)	1.00 (Reference)	1.00 (Reference)
Yes	14 (0.0)	*** (****-****)	*** (****-****)	14 (7.1)	1.76 (0.21-14.89)	2.14 (0.25-8.78)	14 (7.1)	0.42 (0.05-3.32)	0.38 (0.05-3.07)

Model 1, adjusted for age and sex; Model 2, adjusted for age, sex, and educational level for Ghanaians (SSA) and length of stay for those in Europe; Abbreviations: CI, confidence interval; ACR, albumin creatinine ration; eGFR, estimated glomerular filtration rate; CKD, chronic kidney disease; OR, odds ratio, n= total number of Ghanaians living in Ghana and Europe among the various levels of PS constructs; %, proportion of individuals with CKD among the various levels of PS constructs in Ghana and Europe.

For peer review only

For peer review only - http://bmjopen.bmj.com/site/about/guidelines.xhtml

S3: Association of psychosocial stressors (PS) indicators (negative life events, discrimination, stress at work/home and depression) with albuminuria, reduced eGFR and CKD risk for Ghanaians living in Ghana and those living in Europe stratified by diabetes status

	Albuminuria (ACR ≥ 3 mg/mmol)			eGFR < 60 mL/min/1.73 m2			High to very high CKD risk (KDIGO, 2012)		
		OR (95% CI)			OR (95% CI)			OR (95% CI)	
	n (%)	Model 1	Model 2	n (%)	Model 1	Model 2	n cases (%)	Model 1	Model 2
**Negative events**									
**Europe/No diabetes**									
No	986 (8.0)	1.00 (Reference)	1.00 (Reference)	971 (2.1)	1.00 (Reference)	1.00 (Reference)	957 (7.9)	1.00 (Reference)	1.00 (Reference)
Yes	1359 (7.7)	0.95 (0.70-1.29)	0.97 (0.71-1.34)	1340 (2.0)	0.90 (0.50-1.63)	0.90 (0.48-1.68)	1315 (8.1)	1.01 (0.75-1.37)	0.99 (0.72-1.36)
**Europe/Diabetes**									
No	142 (9.9)	1.00 (Reference)	1.00 (Reference)	135 (6.7)	1.00 (Reference)	1.00 (Reference)	133 (12.8)	1.00 (Reference)	1.00 (Reference)
Yes	256 (12.5)	1.28 (0.66-2.50)	1.41 (0.71-2.82)	247 (4.9)	0.75 (0.29-1.89)	10.68 (0.27-1.77)	242 (11.2)	0.87 (0.46-1.69)	0.96 (0.49-1.88)
**Ghana/No diabetes**									
No	683 (9.1)	1.00 (Reference)	1.00 (Reference)	687 (4.1)	1.00 (Reference)	1.00 (Reference)	683 (9.8)	1.00 (Reference)	1.00 (Reference)
Yes	1447 (7.7)	0.84 (0.60-1.16)	0.82 (0.59-1.14)	1451 (3.1)	0.69 (0.42-1.13)	0.69 (0.42-1.13)	1442 (8.3)	0.82 (0.59-1.12)	0.80 (0.58-1.10)
**Ghana/Diabetes**									
No	49 (28.3)	1.00 (Reference)	1.00 (Reference)	49 (10.2)	1.00 (Reference)	1.00 (Reference)	49 (26.4)	1.00 (Reference)	1.00 (Reference)
Yes	148 (24.3)	0.78 (0.37-1.61)	0.72 (0.34-1.52)	150 (7.3)	0.55 (0.16-1.81)	0.57 (0.17-1.88)	148 (26.4)	0.93 (0.45-1.97)	0.90 (0.43-1.92)
**Discrimination**									
**Europe/No diabetes**									
No	1629 (7.9)	1.00 (Reference)	1.00 (Reference)	1609 (2.2)	1.00 (Reference)	1.00 (Reference)	1580 (8.3)	1.00 (Reference)	1.00 (Reference)
Yes	684 (6.7)	0.85 (0.60-1.21)	0.89 (0.62-1.28)	669 (1.5)	0.68 (0.33-1.40)	0.69 (0.32-1.47)	661 (7.0)	0.84 (0.59-1.20)	0.86 (0.59-1.25)
**Europe/Diabetes**									
No	270 (11.9)	1.00 (Reference)	1.00 (Reference)	258 (5.4)	1.00 (Reference)	1.00 (Reference)	252 (13.1)	1.00 (Reference)	1.00 (Reference)
Yes	126 (11.1)	0.87 (0.44-1.71)	0.89 (0.45-1.76)	122 (5.7)	1.29 (0.48-3.45)	1.25 (0.46-3.37)	121 (9.1)	0.68 (0.32-1.40)	0.69 (0.33-1.42)
**Ghana/No diabetes**									
No	1854 (8.4)	1.00 (Reference)	1.00 (Reference)	1865 (3.5)	1.00 (Reference)	1.00 (Reference)	1851 (9.0)	1.00 (Reference)	1.00 (Reference)
Yes	99 (8.1)	1.03 (0.49-2.17)	1.04 (0.49-2.19)	99 (2.0)	0.79 (0.19-3.33)	0.78 (0.18-3.29)	99 (7.1)	0.87 (0.39-1.93)	0.88 (0.39-1.95)

For peer review only - http://bmjopen.bmj.com/site/about/guidelines.xhtml

**Ghana/Diabetes**									
No	180 (26.7)	1.00 (Reference)	1.00 (Reference)	182 (7.7)	1.00 (Reference)	1.00 (Reference)	180 (27.2)	1.00 (Reference)	1.00 (Reference)
Yes	5 (0.0)	**** (***-***)	0.40 (0.05-3.11)	5 (0.0)	**** (***-***)	**** (***-***)	5 (0.0)	**** (***-***)	**** (***-***)
**Stress at home/work**									
**Europe/No diabetes**									
Never	1137 (7.9)	1.00 (Reference)	1.00 (Reference)	1127 (2.3)	1.00 (Reference)	1.00 (Reference)	1102 (8.0)	1.00 (Reference)	1.00 (Reference)
Some stress	860 (7.2)	0.90 (0.65-1.27)	0.96 (0.68-1.36)	850 (1.2)	0.49 (0.24-1.02)	0.49 (0.22-1.07)	835 (7.4)	0.94 (0.67-1.31)	0.99 (0.69-1.41)
Several/Permanent stresses	335 (8.4)	1.04 (0.67-1.62)	1.07 (0.68-1.70)	327 (2.1)	0.84 (0.36-1.95)	0.92 (0.39-2.17)	322 (9.6)	1.20 (0.77-1.85)	1.28 (0.82-1.99)
**Europe/Diabetes**									
Never	193 (9.8)	1.00 (Reference)	1.00 (Reference)	184 (8.2)	1.00 (Reference)	1.00 (Reference)	180 (11.7)	1.00 (Reference)	1.00 (Reference)
Some stress	142 (12.7)	1.22 (0.61-2.45)	1.38 (0.67-2.81)	134 (3.0)	0.43 (0.13-1.39)	0.40 (0.12-1.29)	133 (12.0)	1.04 (0.52-2.09)	1.13 (0.56-2.32)
Several/Permanent stresses	62 (12.9)	1.31 (0.54-3.16)	1.38 (0.56-3.41)	63 (3.2)	0.49 (0.10-2.29)	0.46 (0.10-2.23)	61 (9.8)	0.82 (0.31-2.13)	0.89 (0.34-2.35)
**Ghana/No diabetes**									
Never	628 (7.8)	1.00 (Reference)	1.00 (Reference)	634 (2.7)	1.00 (Reference)	1.00 (Reference)	628 (7.6)	1.00 (Reference)	1.00 (Reference)
Some stress	1163 (8.6)	1.06 (0.74-1.51)	0.97 (0.67-1.39)	1163 (3.7)	1.27 (0.71-2.27)	1.32 (0.74-2.34)	1158 (9.3)	1.15 (0.80-1.65)	1.10 (0.77-1.58)
Several/permanent stresses	339 (7.4)	0.88 (0.53-1.46)	0.76 (0.46-1.27)	341 (3.5)	1.24 (0.58-2.67)	1.35 (0.62-2.95)	339 (8.9)	1.07 (0.66-1.73)	1.00 (0.61-1.64)
**Ghana/Diabetes**									
Never	54 (38.9)	1.00 (Reference)	1.00 (Reference)	54 (11.1)	1.00 (Reference)	1.00 (Reference)	54 (37.0)	1.00 (Reference)	1.00 (Reference)
Some stress	116 (18.9)	**0.37 (0.37-0.77)**	**0.35 (0.17-0.75)**	116 (6.0)	0.47 (0.14-1.60)	0.45 (0.12-1.55)	116 (19.8)	**0.43 (0.21-0.89)**	**0.40 (0.19-0.86)**
Several/Permanent stresses	26 (25.0)	0.44 (0.44-1.29)	0.39 (0.13-1.19)	28 (10.7)	0.65 (0.13-3.21)	0.62 (0.12-3.23)	26 (30.8)	0.69 (0.25-1.93)	0.64 (0.22-1.87)
**Depressive symptoms**									
**Europe/No diabetes**									
No	2154 (8.0)	1.00 (Reference)	1.00 (Reference)	2122 (2.1)	1.00 (Reference)	1.00 (Reference)	2087 (8.1)	1.00 (Reference)	1.00 (Reference)
Yes	167 (5.4)	0.63 (0.32-1.25)	0.69 (0.34-1.38)	163 (2.0)	0.65 (0.15-2.72)	0.72 (0.17-3.04)	161 (6.8)	0.58 (0.25-1.35)	0.86 (0.46-1.64)
**Europe/Diabetes**									

For peer review only - http://bmjopen.bmj.com/site/about/guidelines.xhtml

No	351 (12.0)	1.00 (Reference)	1.00 (Reference)	335 (35.9)	1.00 (Reference)	1.00 (Reference)	329 (12.5)	1.00 (Reference)	1.00 (Reference)
Yes	39 (10.3)	0.79 (0.26-1.35)	0.73 (0.24-2.19)	39 (2.6)	0.49 (0.06-3.97)	0.49 (0.06-3.99)	38 (7.9)	0.89 (0.40-2.01)	0.60 (0.17-2.08)
**Ghana/No diabetes**									
No	2027 (8.3)	1.00 (Reference)	1.00 (Reference)	2035 (3.4)	1.00 (Reference)	1.00 (Reference)	2022 (8.8)	1.00 (Reference)	1.00 (Reference)
Yes	102 (4.9)	0.50 (0.20-1.25)	0.48 (0.19-1.19)	102 (2.9)	0.64 (0.19-2.13)	0.67 (0.20-2.21)	102 (7.8)	0.69 (0.32-1.46)	0.71 (0.34-1.51)
**Ghana/Diabetes**									
No	185 (26.5)	1.00 (Reference)	1.00 (Reference)	187 (8.7)	1.00 (Reference)	1.00 (Reference)	185 27.6)	1.00 (Reference)	1.00 (Reference)
Yes	12 (8.3)	0.27 (0.03-2.13)	0.25 (0.03-2.01)	12 (0.0)	*** (****-****)	*** (***-***)	12 (8.3)	0.42 (0.05-3.32)	0.27 (0.03-2.19)

Model 1, adjusted for age and sex; Model 2, adjusted for age, sex, and educational level for Ghanaians (SSA) and length of stay for those in Europe; Abbreviations: CI, confidence interval; ACR, albumin creatinine ration; eGFR, estimated glomerular filtration rate; CKD, chronic kidney disease; OR, odds ratio, n= total number of Ghanaians living in Ghana and Europe among the various levels of PS constructs; %, proportion of individuals with CKD among the various levels of PS constructs in Ghana and Europe.

For peer review only

For peer review only - http://bmjopen.bmj.com/site/about/guidelines.xhtml

S4: Association of psychosocial stressors (PS) indicators (negative life events, discrimination, stress at work/home and depression) with albuminuria, reduced eGFR and CKD risk for Ghanaians living in Ghana and those living in Europe stratified by hypertensive status

	Albuminuria (ACR ≥ 3 mg/mmol)			eGFR < 60 mL/min/1.73 m2			High to very high CKD risk (KDIGO, 2012)		
		OR (95% CI)			OR (95% CI)			OR (95% CI)	
	n (%)	Model 1	Model 2	n (%)	Model 1	Model 2	n cases (%)	Model 1	Model 2
**Negative events**									
**Europe/No hypertension**									
No	568 (6.9)	1.00 (Reference)	1.00 (Reference)	562 (1.3)	1.00 (Reference)	1.00 (Reference)	551 (7.1)	1.00 (Reference)	1.00 (Reference)
Yes	743 (5.0)	0.72 (0.45-1.15)	0.78 (0.48-1.27)	740 (0.8)	0.59 (0.19-1.77)	0.60 (0.20-1.83)	724 (5.1)	0.71 (0.44-1.13)	0.75 (0.46-1.22)
**Europe/Hypertension**									
No	560 (9.6)	1.00 (Reference)	1.00 (Reference)	544 (4.0)	1.00 (Reference)	1.00 (Reference)	539 (10.0)	1.00 (Reference)	1.00 (Reference)
Yes	872 (11.4)	1.22 (0.86-1.73)	1.20 (0.83-1.73)	847 (3.9)	0.95 (0.54-1.67)	0.89 (0.49-1.62)	833 (11.6)	1.19 (0.83-1.69)	1.13 (0.78-1.62)
**Ghana/No Hypertension**									
No	540 (6.3)	1.00 (Reference)	1.00 (Reference)	543 (2.2)	1.00 (Reference)	1.00 (Reference)	540 (6.9)	1.00 (Reference)	1.00 (Reference)
Yes	1118 (5.6)	0.88 (0.57-1.36)	0.84 (0.54-1.31)	1124 (1.9)	0.82 (0.39-1.69)	0.86 (0.41-1.79)	1115 (6.4)	0.93 (0.61-1.40)	0.90 (0.59-1.36)
**Ghana/Hyperten sion**									
No	192 (21.9)	1.00 (Reference)	1.00 (Reference)	193 (10.9)	1.00 (Reference)	1.00 (Reference)	192 (22.4)	1.00 (Reference)	1.00 (Reference)
Yes	476 (18.1)	0.78 (0.52-1.18)	0.79 (0.52-1.20)	476 (6.9)	0.58 (0.32-1.04)	0.58 (0.33-1.05)	474 (18.4)	0.77 (0.50-1.16)	0.76 (0.51-1.16)
**Discrimination**									
**Europe/No hypertension**									
No	916 (6.3)	1.00 (Reference)	1.00 (Reference)	907 (0.8)	1.00 (Reference)	1.00 (Reference)	885 (6.8)	1.00 (Reference)	1.00 (Reference)
Yes	379 (3.7)	0.60 (0.33-1.09)	0.65 (0.36-1.21)	378 (1.1)	1.35 (0.39-4.74)	1.37 (0.38-4.89)	374 (3.2)	**0.47 (0.25-0.89)**	**0.51 (0.27-0.97)**
**Europe/Hypertension**									
No	983 (10.5)	1.00 (Reference)	1.00 (Reference)	960 (4.4)	1.00 (Reference)	1.00 (Reference)	947 (10.9)	1.00 (Reference)	1.00 (Reference)
Yes	431 (10.7)	1.03 (0.71-1.49)	1.05 (0.72-1.54)	413 (3.2)	0.73 (0.38-1.39)	0.75 (0.38-1.48)	408 (11.0)	1.03 (0.71-1.49)	1.04 (0.71-1.53)
**Ghana/No hypertension**									
No	1427 (5.7)	1.00 (Reference)	1.00 (Reference)	1437 (2.2)	1.00 (Reference)	1.00 (Reference)	1424 (6.5)	1.00 (Reference)	1.00 (Reference)

For peer review only - http://bmjopen.bmj.com/site/about/guidelines.xhtml
For peer review only

Yes	81 (7.4)	1.30 (0.55-3.09)	1.30 (0.55-3.10)	81 (0.0)	**** (***-***)	**** (***-***)	81 (7.4)	1.21 (0.51-2.88)	1.22 (0.51-2.89)
**Ghana/Hypertension**									
No	606 (20.3)	1.00 (Reference)	1.00 (Reference)	609 (7.9)	1.00 (Reference)	1.00 (Reference)	606 (20.3)	1.00 (Reference)	1.00 (Reference)
Yes	23 (8.7)	0.38 (0.09-1.68)	0.40 (0.09-1.74)	23 (8.7)	1.27 (0.28-5.70)	1.20 (0.26-5.44)	23 (4.4)	0.19 (0.03-1.44)	0.19 (0.03-1.45)
**Stress at home/work**									
**Europe/No hypertension**									
Never	644 (6.1)	1.00 (Reference)	1.00 (Reference)	640 (1.3)	1.00 (Reference)	1.00 (Reference)	625 (5.8)	1.00 (Reference)	1.00 (Reference)
Some stress	468 (4.5)	0.74 (0.43-1.27)	0.81 (0.46-1.43)	464 (0.4)	0.37 (0.07-1.74)	0.41 (0.09-2.01)	457 (4.8)	0.84 (0.49-1.45)	0.91 (0.52-1.60)
Several/Permanent stresses	192 (6.8)	1.07 (0.56-2.05)	1.19 (0.61-2.29)	191 (1.6)	1.49 (0.38-5.78)	1.52 (0.39-5.98)	186 (8.1)	1.38 (0.74-2.60)	1.49 (0.79-2.84)
**Europe/Hypertension**									
Never	686 (10.2)	1.00 (Reference)	1.00 (Reference)	665 (5.4)	1.00 (Reference)	1.00 (Reference)	657 (11.1)	1.00 (Reference)	1.00 (Reference)
Some stress	534 (11.1)	1.03 (0.71-1.49)	1.05 (0.72-1.54)	520 (2.3)	**0.49 (0.25-0.96)**	**0.47 (0.23-0.95)**	511 (10.9)	0.97 (0.67-1.41)	0.99 (0.67-0.47)
Several/Permanent stresses	205 (11.2)	1.11 (0.67-1.82)	1.12 (0.67-1.87)	199 (3.0)	0.56 (0.23-1.37)	0.58 (0.24-1.43)	197 (11.2)	0.99 (0.59-1.64)	1.03 (0.61-1.74)
**Ghana/No hypertension**									
Never	495 (5.5)	1.00 (Reference)	1.00 (Reference)	498 (1.4)	1.00 (Reference)	1.00 (Reference)	495 (5.9)	1.00 (Reference)	1.00 (Reference)
Some stress	899 (5.9)	1.07 (0.66-1.73)	0.99 (0.61-1.60)	901 (2.2)	1.30 (0.54-3.15)	1.39 (0.57-3.37)	896 (6.5)	1.04 (0.65-1.65)	1.00 (0.63-1.59)
Several/permanent stresses	263 (5.7)	1.01 (0.53-1.95)	0.89 (0.46-1.72)	267 (2.6)	1.69 (0.58-5.01)	1.89 (0.63-5.65)	263 (7.6)	1.21 (0.67-2.20)	1.15 (0.63-2.11)
**Ghana/Hypertension**									
Never	187 (22.9)	1.00 (Reference)	1.00 (Reference)	190 (8.4)	1.00 (Reference)	1.00 (Reference)	187 (20.9)	1.00 (Reference)	1.00 (Reference)
Some stress	379 (18.2)	0.74 (0.48-1.14)	0.68 (0.43-1.05)	377 (7.9)	0.95 (0.50-1.80)	0.99 (0.51-1.91)	377 (19.4)	0.90 (0.58-1.40)	0.87 (0.55-1.36)
Several/Permanent stresses	102 (15.7)	0.62 (0.33-1.17)	0.54 (0.28-1.04)	102 (7.8)	0.92 (0.37-2.26)	0.97 (0.39-2.43)	102 (17.7)	0.79 (0.42-1.49)	0.76 (0.40-1.43)
**Depressive symptoms**									
**Europe/No hypertension**									

For peer review only

For peer review only - http://bmjopen.bmj.com/site/about/guidelines.xhtml

No	1195 (5.9)	1.00 (Reference)	1.00 (Reference)	1187 (1.1)	1.00 (Reference)	1.00 (Reference)	1162 (6.0)	1.00 (Reference)	1.00 (Reference)
Yes	101 (3.9)	0.59 (0.21-1.65)	0.64 (0.23-1.82)	99 (1.0)	1.18 (0.15-2.72)	1.27 (0.16-10.17)	98 (5.1)	0.77 (0.30-1.97)	0.84 (0.33-2.17)
**Europe/Hypertension**									
No	1310(10.9)	1.00 (Reference)	1.00 (Reference)	1270 (4.1)	1.00 (Reference)	1.00 (Reference)	1254 (11.2)	1.00 (Reference)	1.00 (Reference)
Yes	105 (8.6)	0.76 (0.37-1.53)	0.75 (0.37-1.55)	103 (1.9)	0.52 (0.12-2.18)	0.56 (0.13-2.38)	101 (8.9)	0.77 (0.38-1.58)	0.80 (0.39-1.63)
**Ghana/No hypertension**									
No	1575 (5.9)	1.00 (Reference)	1.00 (Reference)	1584 (2.1)	1.00 (Reference)	1.00 (Reference)	1572 (6.7)	1.00 (Reference)	1.00 (Reference)
Yes	82 (2.4)	0.36 (0.09-1.51)	0.35 (0.08-1.44)	82 (1.2)	0.39 (0059-2.95)	0.39 (0.05-2.97)	82 (3.7)	0.44 (0.13-1.42)	0.43 (0.13-1.40)
**Ghana/Hypertension**									
No	636 (19.5)	1.00 (Reference)	1.00 (Reference)	637 (8.2)	1.00 (Reference)	1.00 (Reference)	634 (19.6)	1.00 (Reference)	1.00 (Reference)
Yes	32 (12.5)	0.59 (0.20-1.72)	0.59 (0.20-1.74)	32 (6.3)	0.74 (0.17-3.25)	0.76 (0.17-3.33)	32 (18.8)	0.94 (0.0.37-2.37)	0.94 (0.38-2.37)

Model 1, adjusted for age and sex; Model 2, adjusted for age, sex, and educational level for Ghanaians (SSA) and length of stay for those in Europe; Abbreviations: CI, confidence interval; ACR, albumin creatinine ration; eGFR, estimated glomerular filtration rate; CKD, chronic kidney disease; OR, odds ratio, n= total number of Ghanaians living in Ghana and Europe among the various levels of PS constructs; %, proportion of individuals with CKD among the various levels of PS constructs in Ghana and Europe.

For peer review only

STROBE Statement—checklist of items that should be included in reports of observational studies

	Item No.	Recommendation	Page No.	Relevant text from manuscript
**Title and abstract**	1	(*a*) Indicate the study's design with a commonly used term in the title or the abstract	1 lines 1-3	We have included a commonly used term in the title and abstract
		(*b*) Provide in the abstract an informative and balanced summary of what was done and what was found	3 lines 87-116	Our study shows no convincing evidence for associations between stress as indicated by four PS constructs and prevalence of CKD. Further studies aimed at identifying potential factors driving the high prevalence of CKD among these populations are needed.
**Introduction**				
Background/rationale	2	Explain the scientific background and rationale for the investigation being reported	5 lines 180-226	The theoretical and scientific background as well as the rationale for conducting the study have been provided in the introduction section.
Objectives	3	State specific objectives, including any pre specified hypotheses	5 lines 222-226	We examined the association between PS and CKD prevalence among rural and urban Ghanaians and their migrants living in three European cities. We also

For peer review only - http://bmjopen.bmj.com/site/about/guidelines.xhtml

				assessed if the influence of PS on CKD is partially mediated by primary risk factors (hypertension and diabetes) of CKD.
**Methods**				
Study design	4	Present key elements of study design early in the paper	-6-7 lines 232-264	Details given in the methods
Setting	5	Describe the setting, locations, and relevant dates, including periods of recruitment, exposure, follow-up, and data collection	6-7 lines 232-264	Rural or urban Ghana and three (Amsterdam, Berlin, London) European cities (migrants)
Participants	6	(*a*) *Cohort study*—Give the eligibility criteria, and the sources and methods of selection of participants. Describe methods of follow-up<br>*Case-control study*—Give the eligibility criteria, and the sources and methods of case ascertainment and control selection. Give the rationale for the choice of cases and controls<br>*Cross-sectional study*—Give the eligibility criteria, and the sources and methods of selection of participants	Pg. 7 lines 254-264	A multi-centred cross sectional baseline data from the Research on Obesity and Diabetes among African Migrants (RODAM) study. A random sample of 5,659 adults (Europe, 3167, rural, 1,043, Urban Ghana, 1,449, Ghana) aged 25 to 70 years.
		(*b*) *Cohort study*—For matched studies, give matching criteria and number of exposed and unexposed<br>*Case-control study*—For matched studies, give matching criteria and the number of controls per case		
Variables	7	Clearly define all outcomes, exposures, predictors, potential confounders, and effect modifiers. Give diagnostic criteria, if applicable	Pg. 7-10 lines 266-357	The main outcomes have been clearly defined.
Data sources/ measurement	8*	For each variable of interest, give sources of data and details of methods of assessment (measurement). Describe comparability of assessment methods if there is more than one group	Pg. 7-10 lines 266-357	We defined each variable of interest in the methods

For peer review only - http://bmjopen.bmj.com/site/about/guidelines.xhtml

				accordingly
Bias	9	Describe any efforts to address potential sources of bias	Pg. 9 lines 315-331	Potential sources of bias have been reported in the methods sections.
Study size	10	Explain how the study size was arrived at	Pg. 7 lines 259-264	Given in the methods section and we have also referred to the RODAM study methods paper

Continued on next page

For peer review only

Quantitative variables	11	Explain how quantitative variables were handled in the analyses. If applicable, describe which groupings were chosen and why	Pg. 10-11 lines 368-388	Please see methods
Statistical methods	12	(*a*) Describe all statistical methods, including those used to control for confounding	Pg. 10-11 lines 367-387	Please see methods
		(*b*) Describe any methods used to examine subgroups and interactions	Pg. 10-11 lines 367-387	Please see methods
		(*c*) Explain how missing data were addressed	Pg. 7 lines 259-264	
		(*d*) *Cohort study*—If applicable, explain how loss to follow-up was addressed<br>*Case-control study*—If applicable, explain how matching of cases and controls was addressed<br>*Cross-sectional study*—If applicable, describe analytical methods taking account of sampling strategy	NA	We have reported non-response across sites
		(*e*) Describe any sensitivity analyses	NA	
**Results**				
Participants	13*	(a) Report numbers of individuals at each stage of study—eg numbers potentially eligible, examined for eligibility, confirmed eligible, included in the study, completing follow-up, and analysed	Pg. 7 lines 254-264	Non-response analysis was done to shed light on the differential response rates across sites
		(b) Give reasons for non-participation at each stage	Pg. 7 lines 259-264	
		(c) Consider use of a flow diagram	Pg. 6 lines 232-234	We have also referred to RODAM methods paper
Descriptive data	14*	(a) Give characteristics of study participants (eg demographic, clinical, social) and information on exposures and potential confounders	Pg. 7 lines 269-357	We have also referred to RODAM methods paper
		(b) Indicate number of participants with missing data for each variable of interest	Pg. 7 lines 260-264	We have also referred to RODAM methods paper
		(c) *Cohort study*—Summarise follow-up time (eg, average and total amount)	NA	
Outcome data	15*	*Cohort study*—Report numbers of outcome events or summary measures over time	*NA*	
		Case-control study—Report numbers in each exposure category, or summary measures of exposure	*NA*	

For peer review only - http://bmjopen.bmj.com/site/about/guidelines.xhtml

		Cross-sectional study—Report numbers of outcome events or summary measures	Pg. 11-13	Summary measures are given in the results section and in tables and figures
Main results	16	(*a*) Give unadjusted estimates and, if applicable, confounder-adjusted estimates and their precision (eg, 95% confidence interval). Make clear which confounders were adjusted for and why they were included	*Pg. 11-23 lines 406-521*	Unadjusted and adjusted estimates are given in the results section and in figures
		(*b*) Report category boundaries when continuous variables were categorized	*Pg. 11-23 lines 389-521*	We have provided mean and corresponding standard deviations for the continuous variables.
		(*c*) If relevant, consider translating estimates of relative risk into absolute risk for a meaningful time	NA	

Continued on next page

Other analyses	17	Report other analyses done—eg analyses of subgroups and interactions, and sensitivity analyses	NA	
**Discussion**				
Key results	18	Summarise key results with reference to study objectives	Pg. 24 lines 524--531	
Limitations	19	Discuss limitations of the study, taking into account sources of potential bias or imprecision. Discuss both direction and magnitude of any potential bias	Pg. 25 lines 584-596	Key limitations regarding study methods including differential response rates and sampling methods in the various study sites have been provided
Interpretation	20	Give a cautious overall interpretation of results considering objectives, limitations, multiplicity of analyses, results from similar studies, and other relevant evidence	Pg. 24-26 lines 522-603	Cautious overall interpretation of the key findings have been provided.
Generalisability	21	Discuss the generalisability (external validity) of the study results	Pg. 25 lines 584-596	
**Other information**				
Funding	22	Give the source of funding and the role of the funders for the present study and, if applicable, for the original study on which the present article is based	Pg. 26 lines 624-630	The funders had no role in study design, data collection and analysis, decision to publish, or preparation of the manuscript

*Give information separately for cases and controls in case-control studies and, if applicable, for exposed and unexposed groups in cohort and cross-sectional studies.

For peer review only - http://bmjopen.bmj.com/site/about/guidelines.xhtml

For peer review only

Note: An Explanation and Elaboration article discusses each checklist item and gives methodological background and published examples of transparent reporting. The STROBE checklist is best used in conjunction with this article (freely available on the Web sites of PLoS Medicine at http://www.plosmedicine.org/, Annals of Internal Medicine at http://www.annals.org/, and Epidemiology at http://www.epidem.com/). Information on the STROBE Initiative is available at www.strobe-statement.org.

For peer review only

BMJ Open

A CROSS-SECTIONAL STUDY OF ASSOCIATION BETWEEN PSYCHOSOCIAL STRESSORS WITH CHRONIC KIDNEY DISEASE AMONG MIGRANT AND NON-MIGRANT GHANAIANS LIVING IN EUROPE AND GHANA: THE RODAM STUDY

Journal:	*BMJ Open*
Manuscript ID	bmjopen-2018-027931.R2
Article Type:	Research
Date Submitted by the Author:	28-May-2019
Complete List of Authors:	Adjei Nana, David ; University of Ghana, Department of Medical Laboratory Sciences; University of Amsterdam, Department of Public Health<br>Stronks, Karien; Academic Medical Center , Department of Public Health<br>Adu, Dwomoa; Korle-bu Teaching Hospital, Department of Medicine<br>Beune, Erik; AMC<br>Meeks, Karlijn; AMC, Public Health<br>Smeeth, Liam; London School of Hygiene and Tropical Medicine,<br>Addo, Juliet; London School of Hygiene and Tropical Medicine, Non Communicable Disease Epidemiology<br>Owusu-Dabo, Ellis; Kwame Nkrumah University of Science and Technology, Kumasi Centre for Collaborative Research in Tropical Medicine<br>Klipstein-Grobusch, Kerstin; 1 Julius Global Health, Julius Center for Health Sciences and Primary Care, University Medical Centre Utrecht, Utrecht, The Netherlands<br>Mockenhaupt, Frank; Charité – University Medicine, Berlin, Institute of Tropical Medicine and International Health<br>Schulze, Matthias; German Institute of Human Nutrition Potsdam-Rehbruecke<br>Danquah, Ina; German Institute of Human Nutrition, Molecular Epidemiology; Charite Universitatsmedizin Berlin, Institute for Social Medicine, Epidemiology and Health Economy<br>Spranger, Joachim; Department of Endocrinology and Metabolism, 1. Charité-University Medicine Berlin, Berlin, Germany.<br>Bahendeka, Silver; St. Francis Hospital Nsambya,<br>Agyemang, Charles; Academic Medical centre, University of Amsterdam, Department of Public Health
**Primary Subject Heading**:	Public health
Secondary Subject Heading:	Epidemiology, Public health, Renal medicine
Keywords:	Chronic Kidney Disease, Psychosocial stressors, migrants, rodam study, Europe, Ghana

SCHOLARONE™
Manuscripts

A CROSS SECTIONAL STUDY OF ASSOCIATION BETWEEN PSYCHOSOCIAL STRESSORS WITH CHRONIC KIDNEY DISEASE AMONG MIGRANT AND NON-MIGRANT GHANAIANS LIVING IN EUROPE AND GHANA: THE RODAM STUDY

David N. Adjei, MSc, PhD[1,2]; Karien Stronks, MSc, PhD[1]; Dwomoa Adu, MD[3]; Erik Beune, MSc, PhD [1]; Karlijn Meeks, MSc, PhD [1]; Liam Smeeth, MD, PhD[4] ; Juliet, Addo, MD, PhD [4]; Ellis Owusu-Dabo, MSc, PhD [5], Kerstin Klipstein-Grobusch, MSc, PhD [6,7]; Frank P. Mockenhaupt, MD, PhD [8] ; Matthias B. Schulze, MSc, PhD [9]; Ina, Danquah, MSc, PhD [9,10] ; Joachim, Spranger, MD, PhD [11,12,13]; Silver Bahendeka, MD, PhD[14]; Charles Agyemang, MPH, PHD[1]

1. Department of Public Health, Academic Medical Center, University of Amsterdam, Amsterdam, Amsterdam Public Health Research Institute, Amsterdam, The Netherlands.
2. Department of Medical Laboratory Sciences, School of Biomedical and Allied Health Sciences, College of Health Sciences, University of Ghana, Accra, Ghana.
3. Department of Medicine, School of Medicine and Dentistry, University of Ghana and Korle-Bu Teaching Hospital, Accra, Ghana.
4. Department of Non-Communicable Disease Epidemiology, London School of Hygiene and Tropical Medicine, London, United Kingdom.
5. Kumasi Centre for collaborative Research, KNUST, Kumasi, Ghana.
6. Julius Global Health, Julius Center for Health Sciences and Primary Care, University Medical Centre, Utrecht University, The Netherlands
7. Division of Epidemiology & Biostatistics, School of Public Health, Faculty of Health Sciences, University of the Witwatersrand, Johannesburg, South Africa
8. Institute of Tropical Medicine and International Health, Charité –University Medicine Berlin, Augustenburger Platz 1, 13353 Berlin, Germany.
9. Department of Molecular Epidemiology, German Institute of Human Nutrition Potsdam-Rehbrücke, Nuthetal, Germany.
10. Charité - Universitaetsmedizin Berlin, Institute for Social Medicine, Epidemiology and Health Economics, Berlin, Germany.
11. Department of Endocrinology and Metabolism, Charité-University Medicine Berlin, Berlin, Germany.
12. German Centre for Cardiovascular Research (DZHK), Berlin, Germany.
13. Center for Cardiovascular Research (CCR), Charité-University, Medicine, Berlin, Germany.

14. MKPGMS - Uganda Martyrs University, Kampala, Uganda.

For peer review only

Address correspondence to David Nana Adjei, MSc, Department of Public Health, Academic Medical Centre, University of Amsterdam, Meibergdreef 9, 1105 AZ Amsterdam, the Netherlands, School Biomedical and Allied Health Sciences, Medical Laboratory Sciences, University of Ghana, E-mail: dna@chs.edu.gh, d.n.adjei@amc.uva.nl, Tel: +233236717850

For peer review only

For peer review only - http://bmjopen.bmj.com/site/about/guidelines.xhtml

Abstract

Objectives: The association between Psychosocial stressors (PS) and CKD among sub-Saharan African (SSA) populations is unknown. We examined the association between PS and CKD prevalence among rural and urban Ghanaians and Ghanaian migrants living in three European cities. We also assessed if the influence of PS on CKD is partially mediated by primary risk factors (hypertension and diabetes) of CKD

Design: A multi-centred cross sectional data from the Research on Obesity and Diabetes among African Migrants (RODAM) study

Setting: Rural and urban Ghana and three European cities (Amsterdam, Berlin and London).

Participants: A random sample of 5,659 adults (Europe 3167, rural Ghana 1,043, and Urban Ghana 1,449) aged 25 to 70 years

Explanatory measures: PS defined by negative life events, perceived discrimination, perceived stress at work/home and depressive symptoms. Three CKD outcomes were considered using the 2012 KDIGO (Kidney Disease: Improving Global Outcomes) severity of CKD classification. Comparisons between PS and CKD outcomes were made using logistic regression analyses across all sites

Results: We observed higher proportion of negative life events (68.7%) and perceived permanent stress (15.9%) among Ghanaians living in Ghana than Ghanaians living in Europe. Depressive symptoms (7.5%) and perceived discrimination (29.7%) were more common among Ghanaians living in Europe than Ghanaians living in Ghana. No significant association was observed between any of the PS constructs and CKD outcomes across sites except for positive association between stress at work/home and albuminuria (2.81, 95% C.I. 1.46-5.40) and CKD risk (2.78, 95% C.I. 1.43-5.43) among Ghanaians living in Berlin

Conclusion: Our study found a positive association between stress at work/home and albuminuria and CKD risk. There was no convincing evidence of associations between the other PS constructs and the prevalence of CKD risk. Further studies, are needed to identify potential factors driving the high prevalence of CKD among these populations.

Index Words: Chronic kidney disease; psychosocial stressors; risk factor; migrants; RODAM study, Europe, Ghana

For peer review only - http://bmjopen.bmj.com/site/about/guidelines.xhtml

Strengths and limitations of the study

- This study used all three categories of CKD definitions (albuminuria, reduced eGFR and CKD risk) by KDIGO 2012 in assessing association of SS with CKD across all sites. This provided more detailed information on CKD outcomes.
- All sites in our study used well standardized study protocols and this eliminated intra protocol variability.
- The use of four constructs of psychosocial stressors (negative life events, perceived discrimination, perceived stress at work/home and depressive symptoms) provided a robust approach to assessing SS associations with CKD.
- The limitation of intra laboratory variability in earlier studies was eliminated using the same standard operating procedures in the same laboratory for running all samples for all sites.
- PS is captured and experienced in different magnitude across different populations. We were unable to ascertain if PS as defined in this study was adequately captured among Ghanaians living in rural and urban Ghana.

For peer review only

Introduction

Worldwide, Chronic Kidney Disease (CKD) is a leading cause of mortality and morbidity with several risk factors (diabetes mellitus, obesity, hypertension and cardiovascular disease) [1]. The epidemiologic transition in low-and-middle income countries (LMICs) shows increased burden of these risk factors [2-4]. CKD's high morbidity and mortality is mostly driven by uncontrolled comorbidities such as diabetes and hypertension [5 6]. CKD treatment and management cost is very high and not sustainable even in high-income countries and this underscores the need for prevention [7]. Available literature has shown that both individual and community level economic factors influence CKD [8-10]. However, after adjusting for both individual and community level socioeconomic position, differences in CKD risk among different populations remained [8 10 11]. These findings seem to suggest that other social environmental factors may be driving CKD prevalence and progression in high-risk populations. Recent studies have shown that whereas Ghanaians living in Europe have a higher CKD risk compared to their host nation populations, they have a lower CKD risk compared with their peers living in urban Ghana [12]. The increased risk of CKD observed in urban Ghana was not fully explained by conventional risk factors [12] and socio-economic status [13]. This underscores the need to identify other modifiable risk factors of CKD for prevention, optimum treatment and efficient management.

Evidence shows that where an individual works or stays influences his or her physiological wellbeing leading to an increased risk of chronic diseases [14 15]. Thus, migrants' physiological wellbeing is influenced by the environment (host nations) they move to stay. The association between PS and CKD as well as the biological pathways through which PS influences CKD progression is poorly understood and complex [5] although several pathways have been suggested [16 17]. Specifically, PS have been reported to be associated with alteration in the sympathetic/autonomic nervous system activity leading to higher rates of traditional risk factors of CKD including hypertension and diabetes [18-20]. Environmental stressors have been reported to contribute to the development of insulin resistance, metabolic syndrome, obesity and diabetes which if uncontrolled leads to CKD incidence [21 22]. Other studies [23 24] have suggested that stress attributable to social and/or economic disadvantage is associated with CKD development and progression through an interaction between other psychosocial factors and comorbid behaviors such as alcohol, tobacco and drug use [25]. In addition, undernutrition due to stress adversely impact on fetal environment by impeding fetal growth leading to low birth weight, which has been shown to be associated with CKD in adult life [25 26].

However, studies linking psychosocial stressors (PS) to CKD prevalence and progression vary greatly among different geographical populations. [5 16 27-31]. Specifically, in the USA whereas no association was

For peer review only - http://bmjopen.bmj.com/site/about/guidelines.xhtml

found between PS and CKD [31-33] another study reported lower prevalence of CKD was associated with greater life stressors at baseline [31]. In contrast, in the Netherlands depressive and anxiety symptoms were observed to be common among CKD patients and such patients had increased risk of poor clinical outcomes [28]. Similarly, a study conducted in Korea reported a positive relationship between depressive symptoms and CKD [27]. These observations suggest differential impact of PS at different geographical locations. For example, discrimination among migrants may differ greatly between host population and from their SSA compatriots. Specifically, some studies have reported differences in PS among rural and urban populations [34].

Current literature on the association between PS and CKD among sub-Saharan African populations and their migrants in Europe is scanty and uncertain. We therefore sought to determine the association between PS and CKD prevalence among Ghanaians in rural and urban Ghana and their migrants living in three European cities. Furthermore, we examined the influence of psychosocial stressors on risk factors (obesity, diabetes and hypertension) of CKD.

Methods

Study population and study design

For this study, data from the Research on Obesity & Diabetes among African Migrants (RODAM) study, a multi-centre cross sectional study, were used. The rationale, conceptual framework, design and methodology of the RODAM study have been described in detail elsewhere [12 13 35 36]. To summarize, the study was conducted from 2012 to 2015. Ghanaians aged 25–70 years living in rural and urban communities in Ghana as well as in three European cities (Amsterdam, Berlin and London) were included in this study. We standardized data collection across all sites. The ethics committees in Ghana, the Netherlands, Germany and the UK approved the study protocol prior to data collection. Informed consent was obtained from each participant prior to enrollment in the study. In Ghana, participants were randomly drawn from a list of 30 enumeration areas in the Ashanti region based on the 2010 population census. These enumeration areas came from both rural areas and two purposively selected urban cities (Kumasi and Obuasi). For Ghanaians in Amsterdam, we randomly drew participants from the Municipal register. This register holds data on country of birth of citizens and their parents, thus allowing for sampling based on the Dutch standard indicator for ethnic origin. London lacked a population register for migrant groups. Thus, Ghanaian organizations served as sampling frame for the study. Lists of these organizations were obtained from the Ghanaian Embassy and the Association of Ghanaian Churches in the UK in the boroughs known to have the greatest concentration of Ghanaians. Members were selected from the lists of all members of these organizations. In Berlin, the registration office of the federal state of Berlin provided a list of Ghanaian

For peer review only - http://bmjopen.bmj.com/site/about/guidelines.xhtml

individuals in Berlin but this resulted in low response rate. Because of this, a change was made to use lists of Ghanaian churches and organizations as the sampling frame. Across all sites in Europe, all selected participants were sent a written invitation combined with written information (information sheet) regarding the study and a response card. The participants were contacted by phone to schedule a date and location of the interview with a trained research assistant or opt for the self-administration of the paper questionnaire or digital online version depending on the preference of the participant. After the completion of the questionnaire, a date for physical examination was then scheduled after a positive response. The participants were instructed to fast from 10.00 p.m. the night before the physical examination. The response rate was 76% in rural Ghana and 74% in urban Ghana. In London, of those individuals who were registered in the various Ghanaian organizations and were invited, 75% agreed and participated in the study, while in Berlin, this figure was 68%, and 53% in Amsterdam. For the current study, 5898 participants with data available on both questionnaire data and physical measurements were used. Individuals who were outside the age range of 25–70 years (n=239) were excluded because not all the study sites had individuals outside this age range resulting in 5659 individuals comprising of 2492 from rural and urban Ghana and 3167 from the three European cities. In the conduct of analysis, we further excluded individuals with no data on CKD and all other indicators (n=52), resulting in a data set of 5607 participants for analysis.

Measurements

Covariates

Demographic and lifestyle factors

For this study, we obtained information on demographics, educational level and lifestyle factors (smoking and physical activity) by questionnaire. Physical examinations were performed across all sites using validated devices per standardized operational procedures. Educational level was based on the highest qualification gained either in the Netherlands or in the country of origin and was classified into 4 groups: those who have never been to school or had elementary schooling only, those with lower vocational schooling or lower secondary schooling, those with intermediate vocational schooling or intermediate/higher secondary education schooling, and those with higher vocational schooling or university. The four categories were further categorized into three categories by combining the second and third categories. Smoking status was determined from the response to the question “Do you smoke at all?” and was classified into nonsmokers and current smokers. Physical activity was assessed using the WHO Global Physical Activity Questionnaire V.2. Weight was measured in light clothing and without shoes with SECA 877 scales to the nearest 0.1 kg. Height was measured without shoes with a portable stadiometer (SECA 217) to the nearest 0.1 cm. Body mass index (BMI) was calculated as weight (kg) divided by height

For peer review only - http://bmjopen.bmj.com/site/about/guidelines.xhtml

squared (m2). Overweight was defined as BMI of 25 to < 30 kg/m^2 and obesity as BMI ≥30 kg/m^2. Waist circumference was measured in centimetres at the midpoint between the lower rib and the upper margin of the iliac crest. We used the same assessor for each participant in measuring all anthropometrics and each was measured twice; the average of the two measurements was used for analyses.

Predictor: SS

For this study, four constructs of psychosocial stress (discrimination, perceived stress at work or at home, negative life events and depressive symptoms) were used as explanatory variables.

Perceived discrimination

Everyday discrimination as perceived by participants was reported as routinely experiencing instances of unfair treatment. We used the Everyday Discrimination Scale (EDS). The EDS comprises of a 9-items which rates the frequency at which participants experience daily mistreatment and it focuses on being treated with less courtesy or less respect, receiving poorer service than other people or being called names or insulted. Participants had the option of rating each of the 9-items from "never" = 1 to "very often" = 5. The obtained scores were summed and an average of the scores was computed to obtain a final score of 1 to 5. This scale was used because it is commonly used for self-reported discrimination [37], with consistent high reliability among a variety of ethnicities [38], comprising African migrants in the Netherland [39].

Perceived stress at work or at home

We defined perceived stress at work or at home as "sense of irritation, filled with anxiety, or as having difficulties in sleeping because of circumstances at work or at home". We used the psychological stress scale created by the INTERHEART study [40]. Participants in the study were asked about their opinion on frequency of stress at work and at home, and could answer "never", "some periods", "several periods", or "continually". Both answers were then combined into a composite score and graded into four categories: never experienced to experienced permanent stress at home or at work [40]. Due to the very small numbers in the permanent periods of stress group, we combined experienced several periods of stress at home or at work and permanent periods of stress at home or at work.

Negative life events

The presence of major negative life events among participants was perceived as any event that could cause acute stress to an individual. We therefore applied the well-validated and widely used List of Threatening Experiences (LTE) [41 42]. The scale comprised of 12 unpleasant events participants perceived to have experienced in the past 12 months. We used a slightly-altered version of LTE consisting of 9 unpleasant items. We dichotomized participants into two groups namely "no negative life events" and "one or more events" and participants in the second category were expected to have higher levels of stress [42].

For peer review only - http://bmjopen.bmj.com/site/about/guidelines.xhtml

Depressive symptoms

Depressive symptoms were measured by the 9-item Patient Health Questionnaire (PHQ-9). The PHQ-9 consists of nine items, with a response scales 0 'not at all', 1 'on several days', 2 'on more than half of the days' and 3 'nearly every day'. A participant was considered to be in a significant depressed mood (SDM) when one or both of the items 1 (little interest or pleasure in doing things) and 2 (feeling down, depressed, or hopeless) were answered with at least 'on more than half of the days', and at least 5 of the 9 items were answered with at least 'on more than half of the days'[43].

Co-morbidity factors

Blood pressure (BP) was measured three times using a validated semi-automated device (The Microlife WatchBP home) with appropriate cuffs in a sitting position after at least 5min rest. The mean of the last two BP measurements was used in the analyses. Hypertension was defined as systolic BP 140mmHg and/or diastolic BP 90mmHg, and/or being on antihypertensive medication treatment, and/or self-reported hypertension. Trained research assistants in all sites collected fasting venous blood samples according to standard operation procedures, and then temporarily stored at the local research location. The stored blood samples from the local research centres were transported to Berlin, Germany, according to standardized procedures, for biochemical analyses. This was done to avoid intra-laboratory variability. Fasting plasma glucose concentration was measured using an enzymatic method (hexokinase). We defined Type 2 diabetes according to the World Health Organization diagnostic criteria (fasting glucose 7.0mmol/L, and/or current use of medication prescribed to treat diabetes, and/or self-reported diabetes) [44]. We assessed concentration of total cholesterol using colorimetric test kits. All biochemical analyses were performed using an ABX Pentra 400 Chemistry Analyzer (ABX Pentra; Horiba 90 ABX, Germany). Hypercholesterolaemia was defined as total cholesterol level ≥6.22mmol/L. Serum creatinine concentration (in mol/L) was determined by a kinetic colorimetric spectrophotometric isotope dilution mass spectrometry calibration method (Roche Diagnostics).

Outcome: CKD prevalence

We asked participants to bring an early morning urine sample for the analyses of albuminuria and creatinine levels. Urinary albumin concentration (in μmol/L) was measured by an immunochemical turbidimetric method (Roche Diagnostics). Urinary creatinine concentration (in l mol/L) was measured by a kinetic spectrophotometric method (Roche Diagnostics). Extensive quality checks were done inclusive of blinded serial measurements. Estimated glomerular filtration rate (eGFR) was calculated using the CKDEPI (CKD Epidemiology Collaboration) creatinine equation [45]. Urinary albumin–creatinine ratio (ACR; expressed in mg/mmol) was calculated by taking the ratio between urinary albumin and urinary creatinine. eGFR and

For peer review only - http://bmjopen.bmj.com/site/about/guidelines.xhtml

For peer review only

albuminuria were categorized according to the 2012 KDIGO classification [46]. eGFR was categorized as follows: G1, 90mL/min/1.73m^2 (normal kidney function); G2, 60–89mL/min/1.73m^2 (mildly decreased); G3a, 45–59mL/min/1.73m^2 (mildly to moderately decreased); G3b, 30–44mL/min/1.73m^2 (moderately to severely decreased); G4, 15–29 mL/min/1.73 m^2 (severely decreased); and G5, <15mL/min/1.73m^2 (kidney failure). Albuminuria categories were derived from ACR and were as follows: A1, < 3mg/mmol (normal to mildly increased); A2, 3–30mg/mmol (moderately increased); and A3, > 30mg/mmol (severely increased). CKD risk was categorized according to severity of kidney disease (green, low risk; yellow, moderately increased risk; orange, high risk; and red, very high risk) using the combination of eGFR (G1–G5) and albuminuria (A1–A3) levels defined by the 2012 KDIGO guideline [47]. Due to the small number of participants in the very high-risk category of CKD (n=27), the high and very high-risk groups were combined. Because of the small number of participants in the severely increased albuminuria category (A3, n=62), we defined albuminuria as ACR 3mg/ mmol by combining the moderately increased (A2) and severely increased (A3) categories.

Covariates assessed were age, sex, educational level and length of stay in Europe, hypertension and diabetes. Length of stay was assessed for Ghanaian migrants only. Length of stay was defined as the number of years lived in Europe at the time of data collection. Length of stay was controlled for due to evidence suggesting that it influences mental health [48]. Other covariates were hypertension, obesity and diabetes.

Patient and Public Involvement

Community leaders were involved in the recruitment of patients. These comprised of religious communities (churches and mosques), endorsement from local key leaders and establishing relationships with healthcare organizations. We also provided information on the study by involving the local media (radio and television stations). We sent letters to all selected health and community authorities to notify participants of the study. Team members were sent to the various communities to stay among the community and organize mini clinics for a period of 1-2 weeks. Results of the study were disseminated through seminars, durbars and via radio and television stations.

Statistical methods

Characteristics of participants were expressed as absolute numbers and percentages for categorical variables and means and standard deviations for continuous variables. The z-test for proportions was used to compare proportions of demographic and clinical variables among the various sites and the independent t-test was also used to test for mean differences between the two sites. Odds Ratios (ORs) and their corresponding 95% confidence intervals (CIs) were estimated by means of binary logistic regression analyses to study the

For peer review only

associations of albuminuria (ACR>3 mg/mmol, A2-A3, moderately to severely increased albuminuria), reduced kidney function (eGFR< 60 mL/min/1.73m^2, G3-G5 moderately to severely decreased kidney function) and increased CKD risk (high and very high CKD risk), with adjustments for covariates [49]. The Spearman's correlation test was used to test for associations between all four constructs of PS. Three models were used to examine the data. Model 1 was adjusted for age and sex, model 2 was adjusted for age and sex and educational level for Ghanaians living in SSA while age, sex, educational level and length of stay for Ghanaians living in Europe [50-52]. Model 3 was adjusted for sex, age, educational level and conventional risk factors (hypertension, diabetes, hypercholesterolemia, BMI, physical activity and smoking status) of CKD. The analyses were performed for all 4 constructs of PS using individuals who have not experienced either of the PS per outcome as reference. All tests were stratified per sites due to interactions, Ghanaians living in SSA and Europe; Ghanaians living in rural and urban Ghana and Ghanaians living in Amsterdam, Berlin and London due to an observed interaction between PS and site. Furthermore, the analyses were stratified for those with and without obesity, diabetes, hypertension across all sites due to interactions between these disease risks. P-values less than 0.05 were interpreted as statistically significant. All analyses were performed using STATA, version 14.0 (StataCorp LP).

Results

Characteristics of the study population

Participants characteristics are shown in Table 1. Ghanaians living in Ghana were significantly older than their peers living in Europe (47.7±11.9 versus 46.6±9.9, p=0.006). There were more female participants in the Ghana sample compared with European sample (67.1% versus 58.5%, p=0.001). Ghanaians living in Ghana were significantly less educated than those living in Europe. Higher proportion of Ghanaians living in Ghana had experienced negative life events in the last 12 months compared with their peers living in Europe (68.7% versus 59.0%, p=0.001). More than half of Ghanaians living in Ghana had experienced some stress at home or work whereas only a third of those living in Europe had experienced some stress at home or work (p=0.001). Permanent stress at home/work was fairly the same among Ghanaians living in SSA and Europe. Perceived discrimination was significantly higher among Ghanaians living in Europe compared with their peers living in Ghana (29.7% versus 4.8%, p=0.001). Depressive symptoms were more prevalent among Ghanaians living in Europe 7.5% compared with their peers living in Ghana 5.1%. Almost all Ghanaians living in Europe were first generation migrants. Ghanaians in Europe were significantly more obese, more likely to smoke and less physically active compared with their peers living in Ghana. Prevalence of hypercholesterolemia was significantly higher, but type 2 diabetes and hypertension were significantly lower among Ghanaians living in Ghana compared with their peers living in Europe (p=0.001).

For peer review only - http://bmjopen.bmj.com/site/about/guidelines.xhtml

416 Prevalence of albuminuria, reduced eGRF and CKD risk were higher in Ghanaians living in Ghana
417 compared with those living in Europe.

418

419

420

421

422 **Table 1: Baseline characteristics of respondents**

	Ghanaians (SSA) n (%)	Ghanaians (Europe) n (%)	p-value
**N**	2,492 (44.1)	3,167 (55.9)	0.001
**Female sex**	1672 (67.1)	1,851 (58.5)	0.001
**Age (years)**	47.7±11.9	46.6±9.9	0.006
**Educational status**			
Low	1169 (49.2)	635 (21.8)	0.001
Middle	858 (36.1)	1111 (38.1)	
High	347 (14.6)	1168 (40.1)	
**Negative life events in the past 12 months**			
No	739 (31.3)	1158 (41.0)	0.001
Yes	1619 (68.7)	1667 (59.0)	
**Perceived stress at home/work**			
Never	692 (29.4)	1371 (48.8)	0.001
Some periods	1290 (54.7)	1033 (36.8)	
Several/Permanent	375 (15.9)	407 (14.4)	
**Perceived discrimination**			
**No**	2065 (95.2)	1960 (70.3)	0.001
**Yes**	104 (4.8)	829 (29.7)	
**Depressive symptoms**			
**No**	2239 (94.9)	2582 (92.5)	0.001

For peer review only - http://bmjopen.bmj.com/site/about/guidelines.xhtml

**Yes**	119 (5.1)	209 (7.5)	
**Migration generation**			
First	Not applicable	2868 (98.7)	Not applicable
Second	Not applicable	38 (1.3)	Not applicable
**BMI**			
Normal (<25kg/m2)	1373 (55.2)	643 (20.4)	0.001
Overweight (25 ≤ 30kg/m2)	684 (27.5)	1,350 (42.8)	
Obese (>30kg/m2)	432 (17.3)	1163 (36.8)	
Currently smoking	36 (1.5)	121 (4.1)	0.001
**Physical activity**	1255 (52.8)	1131 (44.0)	0.001
**Hypocholesteraemia**	352 (14.2)	354 (11.3)	0.007
**Type 2 diabetes**	206 (8.3)	444 (14.0)	0.001
**Hypertension**	837 (33.6)	1801 (56.9)	0.001
**ACR**			
A1 < 3mg/mmol	2215 (90.2)	2814 (91.8)	0.001
A2-A ≥ 3mg/mmol	243 (9.8)	252 (8.2)	
**eGFR**			
G1-G2 ≥ 60 mL/min/1.73 m2	2377 (96.3)	2936 (97.4)	0.018
G3a-G5 < 60 mL/min/1.73 m2	85 (3.7)	78 (2.6)	
**CKD Risk**			
Low risk	2197 (89.6)	2705 (91.5)	0.015
Moderate-very high risk	256 (10.4)	252 (8.5)	

Abbreviations: N, number of respondents; BMI, body mass index; eGFR, estimated glomerular filtration rate; G1-G2, normal to high kidney function to mildly decreased; G3a-G5, mildly to moderately decreased to Kidney failure; ACR, albumin creatinine ratio; A1, normal to mildly increased; A2-A3, moderately increased to severely increased; CKD, Chronic kidney disease; SSA, Sub-Saharan Africa; CI, confidence intervals.

Association between PS and CKD

Figure 1-4 shows CKD prevalence by negative life events in the past 12 months among Ghanaians living in Ghana and Europe. In Europe, CKD prevalence was fairly the same among those who had experienced any negative life events compared with those who had not in the last 12 months. Prevalence of CKD was higher among Ghanaians who had not experienced any negative life events in the past 12 months (10.9%)

For peer review only

For peer review only - http://bmjopen.bmj.com/site/about/guidelines.xhtml

compared with those who had experienced some negative life events (9.9%) and living in Ghana. CKD prevalence was higher among Ghanaians who had not experienced any form of discrimination (10.6%) than those who had (6.7%) in Ghana as well as in Europe (Figure 2). CKD prevalence was slightly higher among Ghanaians who had experienced several/permanent stress at work/home in the past 12 months and living in Ghana (10.4%) or Europe (9.7%) (Figure 3). Ghanaians who did not report any form of depressive symptoms had a significantly higher CKD prevalence than those who did and living in Ghana (10.4%) and Europe (8.7**%)** (Figure 4).

Table 2 shows the correlation matrix between the 4 constructs of PS among Ghanaians living in Ghana and those living in Europe. All four constructs of PS were positively correlated with each other among Ghanaians living in Europe and Ghanaians living in Ghana (p<0.001), except stress at work/home and discrimination among Ghanaians living in Ghana.

Table 2: Relationship between PS constructs (negative life events, discrimination, stress at work or home and depression) by Ghanaians (SSA) and Ghanaians (Europe)

Correlation matrix	Negative events	Discrimination	Stress at work/home	Depression
**Europe**				
Negative events	1.000			
Discrimination	**0.152****	1.000		
Stress at work/home	**0.297****	**0.161****	1.000	
Depressive symptoms	**0.143****	**0.136****	**0.285****	1.000
**Ghana**				
Negative events	1.000			
Discrimination	**0.079****	1.000		
Stress at work/home	**0.101****	-0.032	1.000	
Depressive symptoms	**0.091****	0.042	**0.185****	1.000

****Significant at 1%, Spearman's correlation**

Table 3 shows association between all 4 constructs of PS and CKD among Ghanaians living in Ghana and those living in Europe. There was no statistically significant association between PS and albuminuria, reduced eGFR and CKD risk among Ghanaians living in Ghana and those living in Europe except individuals living in Europe with some stress and lower risk of reduced eGFR (0.46, 95% C.I. 0.24-0.88).

For peer review only - http://bmjopen.bmj.com/site/about/guidelines.xhtml

Table S1 shows further adjustments for conventional risk factors of CKD. This did not show any statistically significant associations between PS and albuminuria, reduced eGFR and CKD risk among Ghanaians living in Ghana and Europe (Table S1). Table S2 shows further stratification based on obesity status. We did not find any association between PS and CKD for obese participants and those who were not obese for Ghanaians living in rural and urban Ghana and those living in Europe. However, we observed an inverse association between PS and CKD among migrants who were not obese but have experienced discrimination for the past 12 months (0.63 95% C.I. 0.41-0.97) (Table S2). In Table S3 we stratified our analysis based on diabetic status. We did not find any associations between PS and CKD for participants with and without diabetes for both Ghanaians living in rural and urban Ghana and their migrant peers in Europe (Table S3). Finally, Table S4 stratified analysis by hypertension status. No associations were observed between PS and CKD for individuals who had hypertension and those who did not have hypertension for both those living in rural and urban Ghana as well as their compatriots living in Europe. An inverse association was observed between PS and CKD among Ghanaians who have experienced discrimination in the last 12 months with no hypertension and living in Europe (0.51, 95% C.I. 0.27-0.97). Also, we observed that having experienced some stress at home/work was inversely associated with reduced eGFR among Ghanaians with hypertension and living in Europe (0.47, 95% C.I. 0. 0.23-0.95) (Table S4).

For peer review only - http://bmjopen.bmj.com/site/about/guidelines.xhtml

Table 3: **Association of psychosocial stressors (PS) indicators (negative life events, discrimination, stress at work/home and depression) with albuminuria, reduced eGFR and CKD risk for Ghanaians living in Ghana and those living in Europe**

	Albuminuria (ACR ≥ 3 mg/mmol)			eGFR < 60 mL/min/1.73 m2			High to very high CKD risk (KDIGO, 2012)		
		OR (95% CI)			OR (95% CI)			OR (95% CI)	
	n (%)	Model 1	Model 2	n (%)	Model 1	Model 2	n cases (%)	Model 1	Model 2
**Negative events**									
**Europe**									
No	1128 (8.2)	1.00 (Reference)	1.00 (Reference)	1106 (2.6)	1.00 (Reference)	1.00 (Reference	1090 (8.5)	1.00 (Reference)	1.00 (Reference)
Yes	1615 (8.4)	1.03 (0.78-1.35)	1.07 (0.80-1.42)	1587 (2.5)	0.86 (0.53-1.42)	0.83 (0.49-1.39)	1557 (8.6)	0.97 (0.76-1.32)	0.99 (0.74-1.32)
**Ghana**									
No	732 (8.7)	1.00 (Reference)	1.00 (Reference)	736 (4.5)	1.00 (Reference)	1.00 (Reference)	732 (10.9)	1.00 (Reference)	1.00 (Reference)
Yes	1595 (3.8)	0.87 (0.65-1.16)	0.85 (0.63-1.14)	1601 (3.4)	0.69 (0.45-1.08)	0.67 (0.44-1.09)	1590 (9.9)	0.88 (0.66-1.17)	0.86 (0.64-1.15)
**Discrimination**									
**Europe**									
No	1899 (8.5)	1.00 (Reference)	1.00 (Reference)	1867 (2.6)	1.00 (Reference)	1.00 (Reference)	1832 (8.9)	1.00 (Reference)	1.00 (Reference)
Yes	810 (7.4)	0.87 (0.64-1.19)	0.92 (0.67-1.26)	791 (2.2)	0.83 (0.47-1.47)	0.84 (0.46-1.52)	782 (7.3)	0.82 (0.59-1.12)	0.84 (0.60-1.16)
**Ghana**									
No	2034 (10.0)	1.00 (Reference)	1.00 (Reference)	2047 (3.9)	1.00 (Reference)	1.00 (Reference)	2031 (10.6)	1.00 (Reference)	1.00 (Reference)
Yes	104 (7.7)	0.83 (0.39-1.73)	0.91 (0.67-1.24)	104 (1.9)	0.67 (0.15-2.85)	0.67 (0.16-2.84)	104 (6.7)	0.70 (0.32-1.55)	0.71 (0.32-1.55)
**Stress at home/work**									
**Europe**									
Never	1330 (8.2)	1.00 (Reference)	1.00 (Reference)	1305 (3.4)	1.00 (Reference)	1.00 (Reference)	1282 (8.5)	1.00 (Reference)	1.00 (Reference)
Some stress	1002 (7.9)	0.97 (0.72-1.31)	1.04 (0.76-1.42)	984 (1.4)	**0.47 (0.26-0.87)**	**0.46 (0.24-0.88)**	968 (8.6)	0.96 (0.71-1.30)	1.02 (0.74-1.39)
Several/Permanent stresses	397 (9.1)	1.11 (0.74-1.64)	1.153 (0.77-1.72)	390 (2.3)	0.73 (0.35-1.52)	0.76 (0.36-1.61)	383 (9.7)	1.13 (0.77-1.68)	1.19 (0.80-1.79)
**Ghana**									
Never	682 (10.3)	1.00 (Reference)	1.00 (Reference)	688 (3.3)	1.00 (Reference)	1.00 (Reference)	682 (9.9)	1.00 (Reference)	1.00 (Reference)

For peer review only - http://bmjopen.bmj.com/site/about/guidelines.xhtml

Some stress	1279 (9.5)	0.87 (0.64-1.19)	0.80 (0.59-1.11)	1279 (3.9)	1.06 (0.63-1.77)	1.11 (0.66-1.87)	1274 (10.3)	0.95 (0.69-1.30)	0.92 (0.67-1.26)
Several/Permanent stresses	365 (8.5)	0.75 (0.48-1.18)	0.68 (0.59-1.11)	369 (4.1)	1.13 (0.57-2.23)	1.22 (0.61-2.46)	365 (10.4)	0.96 (0.63-1.47)	0.92 (0.59-1.42)
**Depressive symptoms**									
**Europe**									
No	2505 (8.5)	1.00 (Reference)	1.00 (Reference)	2457 (2.7)	1.00 (Reference)	1.00 (Reference)	2416 (8.7)	1.00 (Reference)	1.00 (Reference)
Yes	206 (6.3)	0.71 (0.39-1.27)	0.76 (0.43-1.36)	202 (1.5)	0.63 (0.19-2.03)	0.68 (0.21-2.23)	199 (7.1)	0.78 (0.44-1.37)	0.83 (0.47-1.46)
**Ghana**									
No	2212 (9.9)	1.00 (Reference)	1.00 (Reference)	2222 (3.8)	1.00 (Reference)	1.00 (Reference)	2207 (10.4)	1.00 (Reference)	1.00 (Reference)
Yes	114 (5.3)	0.45 (0.19-1.03)	0.45 (0.19-1.01)	114 (2.6)	0.52 (0.16-1.72)	0.53 (0.17-1.74)	114 (7.9)	0.62 (0.30-1.25)	0.61 (0.30-1.24)

Model 1, adjusted for age and sex; Model 2, adjusted for age, sex, and educational level for Ghanaians (SSA) and length of stay for those in Europe; Abbreviations: CI, confidence interval; ACR, albumin creatinine ration; eGFR, estimated glomerular filtration rate; CKD, chronic kidney disease; OR, odds ratio, n= total number of Ghanaians living in Ghana and Europe among the various levels of PS constructs; %, proportion of individuals with CKD among the various levels of PS constructs in Ghana and Europe

For peer review only

490 Table 4 shows associations between all 4 constructs of PS and CKD stratified by Ghanaians living in urban and rural Ghana. There was no association between PS and albuminuria, reduced eGFR and CKD risk among Ghanaians living rural and urban Ghana.

For peer review only

Table 4: Association of psychosocial stressors (PS) indicators (negative life events, discrimination, stress at work/home and depressive symptoms) with albuminuria, reduced eGFR and CKD risk among rural and urban Ghana

	Albuminuria (ACR ≥ 3 mg/mmol)			eGFR < 60 mL/min/1.73 m2			High to very high CKD risk (KDIGO, 2012)		
		OR (95% CI)			OR (95% CI)			OR (95% CI)	
	n (%)	Model 1	Model 2	n (%)	Model 1	Model 2	n cases (%)	Model 1	Model 2
**Negative events**									
**Urban Ghana**									
No	477 (11.9)	1.00 (Reference)	1.00 (Reference)	477 (4.4)	1.00 (Reference)	1.00 (Reference	477 (12.2)	1.00 (Reference)	1.00 (Reference)
Yes	912 (10.5)	0.87 (0.61-1.23)	0.87 (0.61-1.24)	911 (3.4)	0.73 (0.41-1.31)	0.72 (0.40-1.29)	910 (10.8)	0.87 (0.61-1.24)	0.87 (0.61-1.25)
**Rural Ghana**									
No	255 (7.5)	1.00 (Reference)	1.00 (Reference)	259 (4.6)	1.00 (Reference)	1.00 (Reference)	255 (8.6)	1.00 (Reference)	1.00 (Reference)
Yes	683 (7.6)	0.97 (0.56-1.69)	0.94 (0.54-1.64)	690 (3.5)	0.63 (0.31-1.31)	0.66 (0.32-1.37)	680 (8.8)	0.93 (0.55-1.58)	0.92 (0.54-1.56)
**Discrimination**									
**Urban Ghana**									
No	1326 (11.1)	1.00 (Reference)	1.00 (Reference)	1326 (3.9)	1.00 (Reference)	1.00 (Reference)	1325 (11.4)	1.00 (Reference)	1.00 (Reference)
Yes	71 (8.5)	0.85 (0.36-2.00)	0.89 (0.37-2.11)	71 (2.8)	1.17 (0.27-2.09)	1.16 (0.27-2.06)	71 (7.1)	0.69 (0.27-1.77)	0.72 (0.28-1.83)
**Rural Ghana**									
No	708 (8.1)	1.00 (Reference)	1.00 (Reference)	721 (3.9)	1.00 (Reference)	1.00 (Reference)	706 (9.2)	1.00 (Reference)	1.00 (Reference)
Yes	33 (6.1)	0.79 (0.18-3.47)	0.84 (0.19-2.65)	33 (0.0)	***	***	33 (6.1)	0.75 (0.17-2.89)	0.83 (0.19-2.65)
**Stress at home/work**									
**Urban Ghana**									
Never	460 (10.9)	1.00 (Reference)	1.00 (Reference)	460 (3.3)	1.00 (Reference)	1.00 (Reference)	460 (10.2)	1.00 (Reference)	1.00 (Reference)
Some stress	732 (11.5)	1.04 (0.71-1.51)	0.91 (0.62-1.37)	730 (4.1)	1.27 (0.66-2.43)	1.30 (0.67-2.51)	730 (11.8)	1.13 (0.77-1.65)	1.04 (0.71-1.53)
Several/Permanent stresses	197 (9.6)	0.87 (0.50-1.52)	0.74 (0.42-1.02)	198 (3.5)	1.17 (0.46-2.84)	1.20 (0.47-3.09)	197 (11.7)	1.15 (0.68-1.71)	1.05 (0.61-1.81)

For peer review only - http://bmjopen.bmj.com/site/about/guidelines.xhtml

**Rural Ghana**									
Never	222 (9.0)	1.00 (Reference)	1.00 (Reference)	228 (3.5)	1.00 (Reference)	1.00 (Reference)	222 (9.5)	1.00 (Reference)	1.00 (Reference)
Some stress	547 (6.9)	0.69 (0.39-1.23)	0.68 (0.38-1.22)	549 (3.6)	0.88 (0.38-2.07)	0.92 (0.39-2.18)	544 (8.3)	0.74 (0.42-1.30)	0.75 (0.42-1.31)
Several/Permanent stresses	168 (7.1)	0.63 (0.30-1.37)	0.60 (0.28-1.29)	171 (4.7)	1.07(0.38-3.03)	1.21(0.43-3.46)	168 (8.9)	0.71 (0.34-1.50)	0.73 (0.35-1.50)
**Depressive symptoms**									
**Urban Ghana**									
No	1336 (11.3)	1.00 (Reference)	1.00 (Reference)	1335 (3.8)	1.00 (Reference)	1.00 (Reference)	1334 (11.5)	1.00 (Reference)	1.00 (Reference)
Yes	52 (3.9)	0.30 (0.07-1.25)	0.30 (0.07-1.27)	52 (1.9)	0.46 (0.06-2.50)	0.45 (0.06-2.13)	52 (5.8)	0.44 (0.14-1.45)	0.45 (0.14-1.48)
**Rural Ghana**									
No	876 (7.7)	1.00 (Reference)	1.00 (Reference)	887 (3.8)	1.00 (Reference)	1.00 (Reference)	873 (8.7)	1.00 (Reference)	1.00 (Reference)
Yes	62 (6.5)	0.67 (0.23-1.94)	0.67 (0.23-1.94)	62 (3.2)	0.58 (0.13-2.56)	0.61 (0.14-2.68)	62 (9.7)	0.82 (0.33-2.01)	0.85 (0.34-2.09)

Model 1, adjusted for age and sex; Model 2, adjusted for age, sex, and educational level; Abbreviations: CI, confidence interval; ACR, albumin creatinine ration; eGFR, estimated glomerular filtration rate; CKD, chronic kidney disease; OR, odds ratio, n= total number of Ghanaians living in urban and rural Ghana among the various levels of PS constructs; %, proportion of individuals with CKD among the various levels of PS constructs in rural and urban Ghana. ***; no case of CKD and therefore odds ratios were not calculated

For peer review only

For peer review only - http://bmjopen.bmj.com/site/about/guidelines.xhtml

Table 5 shows associations between all 4 constructs of PS and CKD stratified by Ghanaians living in Amsterdam, Berlin and London. There were no associations between PS and albuminuria, reduced eGFR and CKD risk among Ghanaians living in Europe except for positive association between stress at work/home and albuminuria (2.81, 95% C.I. 1.46-5.40) and CKD risk (2.78, 95% C.I. 1.43-5.43) among Ghanaians living in Berlin.

For peer review only

Table 5: **Association of psychosocial stressors (PS) indicators (negative life events, discrimination, stress at work/home and depressive symptoms) with albuminuria, reduced eGFR and CKD risk among Ghanaians in three European cities.**

	Albuminuria (ACR ≥ 3 mg/mmol)			eGFR < 60 mL/min/1.73 m2			High to very high CKD risk (KDIGO, 2012)		
		OR (95% CI)			OR (95% CI)			OR (95% CI)	
	n (%)	Model 1	Model 2	n (%)	Model 1	Model 2	n (%)	Model 1	Model 2
**Negative events**									
**Amsterdam**									
No	548 (7.3)	1.00 (Reference)	1.00 (Reference)	534 (2.4)	1.00 (Reference)	1.00 (Reference))	521 (7.5)	1.00 (Reference)	1.00 (Reference)
Yes	784 (7.8)	1.08 (0.71-1.63)	1.18 (0.77-1.81)	764 (2.9)	1.11 (0.55-2.23)	1.15 (0.55-2.37)	742 (8.0)	1.06 (0.69-1.62)	1.08 (0.71-1.66)
**Berlin**									
No	213 (9.9)	1.00 (Reference)	1.00 (Reference)	213 (2.4)	1.00 (Reference)	1.00 (Reference)	213 (10.8)	1.00 (Reference)	1.00 (Reference)
Yes	329 (10.9)	1.12 (0.63-1.99)	1.19 (0.67-2.15)	330 (1.8)	0.64 (0.19-2.17)	0.61 (0.18-2.11)	329 (9.4)	0.86 (0.48-1.52)	0.91 (0.51-1.63)
**London**									
No	367 (8.7)	1.00 (Reference)	1.00 (Reference)	359 (3.1)	1.00 (Reference)	1.00 (Reference)	356 (8.7)	1.00 (Reference)	1.00 (Reference)
Yes	502 (7.8)	0.89 (0.55-1.46)	0.83 (0.49-1.41)	493 (2.2)	0.68 (0.28-1.65)	0.58 (0.22-1.51)	486 (9.1)	1.04 (0.64-1.68)	0.99 (0.58-1.68)
**Discrimination**									
**Amsterdam**									
No	956 (8.3)	1.00 (Reference)	1.00 (Reference)	935 (2.9)	1.00 (Reference)	1.00 (Reference)	909 (8.5)	1.00 (Reference)	1.00 (Reference)
Yes	363 (5.0)	0.59 (0.34-1.00)	0.59 (0.35-1.02)	349 (2.1)	0.69 (0.30-1.62)	0.81 (0.34-1.91)	342 (5.9)	0.69 (0.41-1.14)	0.69 (0.41-1.16)
**Berlin**									
No	329(10.0)	1.00 (Reference)	1.00 (Reference)	329 (2.1)	1.00 (Reference)	1.00 (Reference)	329 (10.3)	1.00 (Reference)	1.00 (Reference)
Yes	209 (11.0)	1.11 (0.63-1.95)	1.16 (0.65-2.05)	210 (1.9)	0.83 (0.24-2.93)	0.82 (0.23-2.91)	209 (9.1)	0.86 (0.48-1.56)	0.89 (0.49-1.63)
**London**									
No	614 (7.9)	1.00 (Reference)	1.00 (Reference)	603 (2.5)	1.00 (Reference)	1.00 (Reference)	594 (8.9)	1.00 (Reference)	1.00 (Reference)
Yes	238 (7.9)	1.03 (0.59-1.81)	1.18 (0.65-2.15)	232 (2.6)	1.29 (0.46-3.59)	1.09 (0.35-3.43)	231 (7.8)	0.93 (0.53-1.63)	0.98 (0.52-1.82)
**Stress at home/work**									

For peer review only

**Amsterdam**									
Never	634 (8.4)	1.00 (Reference)	1.00 (Reference)	622 (3.2)	1.00 (Reference)	1.00 (Reference)	603 (8.0)	1.00 (Reference)	1.00 (Reference)
Some stress	478 (5.7)	0.68 (0.42-1.11)	0.69 (0.42-1.13)	462 (1.9)	0.64 (0.29-1.43)	0.68 (0.30-1.52)	452 (6.0)	0.74 (0.45-1.20)	0.74 (0.45-1.22)
Several/Permanent stresses	210 (9.1)	1.09 (0.63-1.91)	1.12 (0.64-1.95)	204 (2.5)	0.71 (0.26-1.95)	0.77 (0.28-2.14)	198 (10.1)	1.24 (0.71-2.14)	1.26 (0.73-2.20)
**Berlin**									
Never	250 (9.0)	1.00 (Reference)	1.00 (Reference)	250 (2.0)	1.00 (Reference)	1.00 (Reference)	250 (6.4)	1.00 (Reference)	1.00 (Reference)
Some stress	196 (15.3)	**2.50 (1.33-4.71)**	**2.81 (1.46-5.40)**	197 (1.5)	0.88 (0.20-3.79)	0.83 (0.19-3.62)	196 (14.8)	**2.57 (1.34-4.90)**	**2.78 (1.43-5.43)**
Several/Permanent stresses	96 (10.4)	1.64 (0.72-3.73)	1.69 (0.73-3.91)	197 (3.1)	2.10 (0.47-9.46)	2.04 (0.44-9.26)	76 (9.4)	1.52 (0.65-3.58)	1.58 (0.66-3.75)
**London**									
Never	446 (9.2)	1.00 (Reference)	1.00 (Reference)	433 (4.4)	1.00 (Reference)	1.00 (Reference)	429 (10.5)	1.00 (Reference)	1.00 (Reference)
Some stress	328 (7.0)	0.73 (0.43-1.25)	0.79 (0.44-1.40)	325 (0.6)	0.17 (0.04-0.73)	0.09 (0.01-0.67)	320 (6.9)	0.65 (0.38-1.10)	0.66 (0.37-1.19)
Several/permanent stresses	91 (7.7)	0.81 (0.35-1.87)	0.86 (0.35-2.14)	90 (1.1)	0.27 (0.03-2.12)	0.24 (0.03-2.05)	74 (8.9)	0.83 (0.38-1.83)	0.92 (0.39-2.16)
**Depressive symptoms**									
**Amsterdam**									
No	1199 (7.8)	1.00 (Reference)	1.00 (Reference)	1135 (2.8)	1.00 (Reference)	1.00 (Reference)	1135 (7.9)	1.00 (Reference)	1.00 (Reference)
Yes	121 (6.6)	0.81 (0.39-1.72)	0.83 (0.39-1.76)	118 (1.7)	0.65 (0.15-2.77)	0.71 (0.16-3.06)	116 (6.9)	0.83 (0.39-1.76)	0.82 (0.38-1.74)
**Berlin**									
No	503 (10.7)	1.00 (Reference)	1.00 (Reference)	504 (2.2)	1.00 (Reference)	1.00 (Reference)	503 (10.1)	1.00 (Reference)	1.00 (Reference)
Yes	34 (5.9)	0.53 (0.12-2.27)	0.49 (90.11-2.13)	34 (0.0)	***	***	34 (5.9)	0.55 (0.13-2.37)	0.52 (0.12-2.24)
**London**									
No	803 (8.3)	1.00 (Reference)	1.00 (Reference)	785 (2.6)	1.00 (Reference)	1.00 (Reference)	778 (8.9)	1.00 (Reference)	1.00 (Reference)
Yes	51 (5.9)	0.67 (0.20-2.21)	0.91 (0.27-3.07)	50 (2.0)	0.91 (0.11-7.43)	1.15 (0.14-9.54)	49 (8.2)	0.94 (0.33-2.69)	1.30 (0.44-3.81)

Model 1, adjusted for age and sex; Model 2, adjusted for age, sex, educational level and length of stay; Abbreviations: CI, confidence interval; ACR, albumin creatinine ration; eGFR, estimated glomerular filtration rate; CKD, chronic kidney disease; OR, odds ratio, n= total number of Ghanaians living in Amsterdam, Berlin and London among the various levels of PS constructs; %, proportion of individuals with CKD among the various levels of PS constructs in Europe ***; no case of CKD and therefore odds ratios were not calculated.

For peer review only - http://bmjopen.bmj.com/site/about/guidelines.xhtml

Discussion

Key findings

Whereas there was an association between those who have experienced some stress at home/work and reduced eGFR among Ghanaians living in Europe, we did not find any association between PS and CKD among Ghanaians living in rural and urban Ghana and their peers in Europe. Also, PS was not associated with CKD for those living in rural and urban Ghana and neither for those living in the three European cities. However, there was an association between stress at work/home and albuminuria and CKD risk among Ghanaians living in Berlin. Further adjustment for conventional risk factors of CKD yielded similar results.

Discussion of key findings

Association between PS and CKD in Ghana

Our study did not find any association between any of the four constructs of PS and prevalence of CKD (albuminuria, reduced eGFR and CKD risk) among Ghanaians living in rural and urban Ghana. Our findings are however in contrast with earlier studies which reported positive associations between PS and prevalent of CKD [28 31 53]. Other studies have hypothesised that the influence of PS on CKD may be important in only those with hypertension and diabetes and that PS may mediate or moderate the association between renal functioning and lifestyle behaviours such as smoking and physical activity [33]. For example, they argue that stress enhances Sympathetic Nervous System (SNS) activity to increase glucocorticoid secretion and inflammatory cytokines, which heavily contribute to hypertension, diabetes and vascular disease, which are major risk factors of CKD incidence and prevalence [54]. The lack of association between PS and CKD in this present study is unclear due to lack of literature on the association between PS and CKD prevalence particularly in rural and urban populations. Worth noting, however, is the presence of rich family support systems in the Ghanaian context especially in rural Ghana, which may help individuals with CKD to cope with PS thereby minimizing its effect. For example, patients with limited social networks and low social support have been shown to have augmented risk of morbidity and mortality [55-57]. Specifically, there is evidence that positive social support is a protective factor for persons dealing with long-term disease conditions [58]. Other studies have reported a protective relationship between social networks, emotionally supportive relationship and threats to physiological and psychosocial health [59].

Association between PS and CKD Amsterdam, Berlin and London

Literature on the association between PS and CKD prevalence among migrants is scant and absent in most European populations. The lack of positive association between PS and CKD in our study is consistent with

For peer review only - http://bmjopen.bmj.com/site/about/guidelines.xhtml

recent studies conducted among African Americans [31 33] and other populations [32 60]. Specifically, a recent study using data from the Jackson Heart Study, which comprised of extensive constructs of psychosocial variables reported that greater life stressors were associated with lower prevalence of CKD at baseline [31]. Several studies in other parts of the world have reported a positive relationship between higher prevalence of stressors and CKD risk [27 28], although the study findings have been inconsistent. Whereas some did not find any associations among African Americans [31] others found associations in other populations. Even among those who found some associations the directions differed [28]. Reasons for the lack of association observed in our study among migrants are not fully understood but may reflect the real world situation. First, migrants from Ghana practice both nuclear and extended family support system as their peers living in rural and urban, this practice may mitigate the impact of stressors such as unemployment, death of a love one, discrimination, etc. They also belong to several religious organisations such as churches, which provide similar support systems against stressors. Moreover, there are several associations of the various ethnic groups (Akan, Ga and Ewe) providing such support when the need arises. These systems provide both instrumental and/or emotional social support [61]. These assertions are supported by several studies. Specifically, these studies have shown that social support positively affect health outcomes through mechanisms such as increased patient compliance with therapies, decreased levels of depressive affect, direct physiologic effects on the immune system and improved perception of quality of life [58 59]. The lack of association observed in this study may also be attributed to other mechanisms, which influence the associations between PS and CKD. Another reason for the lack of association between PS and CKD in this study could be the cross-sectional analysis of our study. The association between PS and CKD has been shown to be cumulative and builds over substantial period of time[62]. To effectively evaluate this, longitudinal study design is required. This suggests the needs for more longitudinal studies in future research in assessing the associations between PS and CKD outcomes [62 63].

Strength and limitation

Our study is the first to use all four robust constructs of PS to determine association between PS and CKD. This gave our study a more robust definition of PS compared to other similar studies. The use of all three definitions of CKD per KDIGO guidelines also provided a broader definition of CKD and allowed comparison between different geographical regions. The use of a homogenous population of Ghanaians and standardized protocols and diagnostic criteria in this study also provided a novel opportunity to compare Ghanaians living in rural and urban Ghana and their compatriots living in Europe. There are limitations to our study. The effect of PS on CKD has been reported to be cumulative and takes a long period of time, therefore the use of cross-sectional design prevented us from determining the longitudinal and cumulative

For peer review only - http://bmjopen.bmj.com/site/about/guidelines.xhtml

effect of repeated exposure to PS among the two populations. PS is captured and experienced in different magnitude across different populations. We were unable to ascertain if PS as defined in this study was adequately captured among Ghanaians living in rural and urban Ghana. Lastly, the four PS measures were assessed separately because of multicollinearity among the measures. There are several methods of addressing multicollinearity among measures such as partial least squares regression, principal component analysis, data reduction technique, which when used could have influenced the interpretation of the study results.

Conclusion

We identified positive association between stress at work/home and albuminuria and CKD risk among Ghanaians living in Berlin. Conversely, our study shows no associations between stress as indicated by four PS indicators and prevalence of CKD. Consequently, there is the need to explore other factors that may be responsible for the observed differences in the prevalence of CKD among Ghanaians living in rural and urban Ghana and their peers living in Europe.

Acknowledgement

The authors are very grateful to the research assistants, interviewers and other staff of the five research locations who took part in gathering the data and the Ghanaian volunteers in all the participating RODAM sites. We gratefully acknowledge the advisory board members for their valuable support in shaping the RODAM study methods and the Academic Medical Centre Biobank for their support in biobank management and high-quality storage of collected samples.

Contributors

My co-authors have all contributed substantially to this manuscript and approve of this submission. Research idea and study design: DNA, CA, KS, DA, EB, KM, JA; data acquisition and curation: DNA, CA, EB, KM, data analysis/interpretation: DNA, CA, KS, DA, EB, KM, LS, JA, EOD, KKG, FPM, MBS, ID, JS, SB; statistical analysis: DNA, CA, KS. DNA, CA, KS, DA, EB, KM, LS, JA, EOD, KKG, FPM, MBS, ID, JS, SB; contributed important intellectual content during manuscript drafting or revision and accepts accountability for the overall work by ensuring that questions pertaining to the accuracy or integrity of any portion of the work are appropriately investigated and resolved. DNA and CA takes responsibility that this study has been reported honestly, accurately, and transparently; that no important aspects of the study have been omitted; and that any discrepancies from the study as planned have been explained.

Funding

For peer review only

For peer review only - http://bmjopen.bmj.com/site/about/guidelines.xhtml

This work was supported by the European Commission under the Framework Programme (Grant Number: 278901). The funders had no role in study design, data collection and analysis, decision to publish, or preparation of the manuscript. The Wellcome Trust supported Professor Smeeth's contribution, grant number WT082178. Professor Joachim Spranger was supported by the DZHK (German Center for cardiovascular research) and the Berlin Institute of Health (BIH).

Competing interest: None Declared

Patient Consent: None declared

Ethics approval: IRBs at each participating site.

Data sharing statement: Data are available from the RODAM research cohort, a third party. Dr. Eric Beune affiliated with the RODAM research cohort and a co-author of this paper in accordance with the RODAM requirements for collaboration. Dr. Beune is the Data Collection Coordinator of RODAM and may be contacted with further questions (e.j.beune@amc.uva.nl). Additionally, researchers interested in further collaboration with RODAM may see the following URL: http://www.rod-am.eu/

For peer review only - http://bmjopen.bmj.com/site/about/guidelines.xhtml

References

1. Weisbord SD. Symptoms and their correlates in chronic kidney disease. *Adv Chronic Kidney Dis* 2007;14(4):319-27.
2. Correa-Rotter R, Naicker S, Katz IJ, et al. Demographic and epidemiologic transition in the developing world: role of albuminuria in the early diagnosis and prevention of renal and cardiovascular disease. *Kidney Int* 2004;66:S32-S37.
3. Lozano R, Naghavi M, Foreman K, et al. Global and regional mortality from 235 causes of death for 20 age groups in 1990 and 2010: a systematic analysis for the Global Burden of Disease Study 2010. *The lancet* 2012;380(9859):2095-128.
4. Katz IJ, Gerntholtz T, Naicker S. Africa and nephrology: the forgotten continent. *Nephron Clinical Practice* 2011;117(4):320-27.
5. Bruce MA, Beech BM, Sims M, et al. Social environmental stressors, psychological factors, and kidney disease. *J Investig Med* 2009;57(4):583-89.
6. Osafo C, Mate-Kole M, Affram K, et al. Prevalence of chronic kidney disease in hypertensive patients in Ghana. *Ren Fail* 2011;33(4):388-92.
7. Ojo A. Addressing the global burden of chronic kidney disease through clinical and translational research. *Trans Am Clin Climatol Assoc* 2014;125:229.
8. Norris K, Nissenson AR. Race, gender, and socioeconomic disparities in CKD in the United States. *J Am Soc Nephrol* 2008;19(7):1261-70.
9. Volkova N, McClellan W, Klein M, et al. Neighborhood poverty and racial differences in ESRD incidence. *J Am Soc Nephrol* 2008;19(2):356-64.
10. Shoham DA, Vupputuri S, Roux AVD, et al. Kidney disease in life-course socioeconomic context: the Atherosclerosis Risk in Communities (ARIC) Study. *Am J Kidney Dis* 2007;49(2):217-26.
11. Tarver-Carr ME, Powe NR, Eberhardt MS, et al. Excess risk of chronic kidney disease among African-American versus white subjects in the United States: a population-based study of potential explanatory factors. *J Am Soc Nephrol* 2002;13(9):2363-70.
12. Adjei DN, Stronks K, Adu D, et al. Chronic kidney disease burden among African migrants in three European countries and in urban and rural Ghana: the RODAM cross-sectional study. *Nephrology Dialysis Transplantation* 2018
13. Adjei DN, Stronks K, Adu D, et al. Relationship between educational and occupational levels, and Chronic Kidney Disease in a multi-ethnic sample-The HELIUS study. *PLoS One* 2017;12(11):e0186460.
14. Fremont A, Bird CE. Social and psychological factors, physiological processes, and physical health. 2000
15. Krantz DS, McCeney MK. Effects of psychological and social factors on organic disease: a critical assessment of research on coronary heart disease. *Annu Rev Psychol* 2002;53(1):341-69.

For peer review only - http://bmjopen.bmj.com/site/about/guidelines.xhtml

16. Cukor D, Fruchter Y, Ver Halen N, et al. A preliminary investigation of depression and kidney functioning in patients with chronic kidney disease. *Nephron Clinical practice* 2012;122(3-4):139-45.
17. Davidson K, Jonas BS, Dixon KE, et al. Do depression symptoms predict early hypertension incidence in young adults in the CARDIA study? *Arch Intern Med* 2000;160(10):1495-500.
18. Amerena J, Julius S. The role of the autonomic nervous system in hypertension. *Hypertens Res* 1995;18(2):99-110.
19. Calhoun DA, Mutinga ML. Race, family history of hypertension, and sympathetic response to cold pressor testing. *Blood Press* 1997;6(4):209-13.
20. Dibona GF. Neural control of the kidney: past, present, and future. *Hypertension* 2003;41(3):621-24.
21. Auchincloss AH, Diez Roux AV, Brown DG, et al. Association of insulin resistance with distance to wealthy areas: the multi-ethnic study of atherosclerosis. *Am J Epidemiol* 2006;165(4):389-97.
22. Bruce MA, Sims M, Miller S, et al. One size fits all? Race, gender and body mass index among US adults. *J Natl Med Assoc* 2007;99(10):1152.
23. McEwen BS. Protective and damaging effects of stress mediators. *N Engl J Med* 1998;338(3):171-79.
24. Cohen S, Herbert TB. Health psychology: Psychological factors and physical disease from the perspective of human psychoneuroimmunology. *Annu Rev Psychol* 1996;47(1):113-42.
25. Barker D. Fetal origins of coronary heart disease. *Br Heart J* 1993;69(3):195.
26. Phillips DI, Jones A, Goulden PA. Birth weight, stress, and the metabolic syndrome in adult life. *Ann N Y Acad Sci* 2006;1083(1):28-36.
27. Kim JW, Moon SJ, Kim HJ, et al. Relationship between Chronic Kidney Disease and Depression in Elderly Koreans Using the 2013 Korea National Health and Nutrition Examination Survey Data. *Korean journal of family medicine* 2017;38(3):156-62.
28. Loosman WL, Rottier MA, Honig A, et al. Association of depressive and anxiety symptoms with adverse events in Dutch chronic kidney disease patients: a prospective cohort study. *BMC Nephrol* 2015;16(1):155.
29. Novak M, Mucsi I, Mendelssohn D. Screening for depression: only one piece of the puzzle. *Nephrology Dialysis Transplantation* 2013;28(6):1336-40.
30. McKercher CM, Venn AJ, Blizzard L, et al. Psychosocial factors in adults with chronic kidney disease: characteristics of pilot participants in the Tasmanian Chronic Kidney Disease study. *BMC Nephrol* 2013;14(1):83.
31. Lunyera J, Davenport CA, Bhavsar NA, et al. Nondepressive Psychosocial Factors and CKD Outcomes in Black Americans. *Clin J Am Soc Nephrol* 2018:CJN. 06430617.
32. Annor FB, Masyn KE, Okosun IS, et al. Psychosocial stress and changes in estimated glomerular filtration rate among adults with diabetes mellitus. *Kidney research and clinical practice* 2015;34(3):146-53.
33. Gholson GK, Mwendwa DT, Wright RS, et al. The Combined Influence of Psychological Factors on Biomarkers of Renal Functioning in African Americans. *Ethn Dis* 2015;25(2):117.
34. Kaur M, Kaur A, Saggu A, et al. Comparative study on psychosocial stresses among urban and rural geriatric population in selected areas of district Ludhiana (Punjab), 2006. *Nursing and Midwifery Research* 2007;3(2)

For peer review only - http://bmjopen.bmj.com/site/about/guidelines.xhtml

35. Agyemang C, Beune E, Meeks K, et al. Rationale and cross-sectional study design of the Research on Obesity and type 2 Diabetes among African Migrants: the RODAM study. *BMJ open* 2015;4(3):e004877.
36. Agyemang C, Snijder MB, Adjei DN, et al. Ethnic Disparities in CKD in the Netherlands: The Healthy Life in an Urban Setting (HELIUS) Study. *Am J Kidney Dis* 2016;67(3):391-99.
37. Peek ME, Nunez-Smith M, Drum M, et al. Adapting the everyday discrimination scale to medical settings: reliability and validity testing in a sample of African American patients. *Ethn Dis* 2011;21(4):502.
38. Pérez DJ, Fortuna L, Alegria M. Prevalence and correlates of everyday discrimination among US Latinos. *J Community Psychol* 2008;36(4):421-33.
39. Ikram UZ, Snijder MB, Fassaert TJ, et al. The contribution of perceived ethnic discrimination to the prevalence of depression. *The European Journal of Public Health* 2014;25(2):243-48.
40. Rosengren A, Hawken S, Ôunpuu S, et al. Association of psychosocial risk factors with risk of acute myocardial infarction in 11 119 cases and 13 648 controls from 52 countries (the INTERHEART study): case-control study. *The Lancet* 2004;364(9438):953-62.
41. Rosmalen J, Bos E, De Jonge P. Validation of the Long-term Difficulties Inventory (LDI) and the List of Threatening Experiences (LTE) as measures of stress in epidemiological population-based cohort studies. *Psychol Med* 2012;42(12):2599-608.
42. Brugha T, Bebbington P, Tennant C, et al. The List of Threatening Experiences: a subset of 12 life event categories with considerable long-term contextual threat. *Psychol Med* 1985;15(1):189-94.
43. Kroenke K, Spitzer RL, Williams JB. The phq-9. *J Gen Intern Med* 2001;16(9):606-13.
44. Association AD. Standards of Medical Care in Diabetes—2014. Diabetes Care 2014; 37 (Suppl. 1): S14–S80Diagnosis and Classification of Diabetes Mellitus. Diabetes Care 2014; 37 (Suppl. 1): S81–S90. *Diabetes Care* 2014;37(3):887-87.
45. Stevens LA, Schmid CH, Greene T, et al. Comparative performance of the CKD Epidemiology Collaboration (CKD-EPI) and the Modification of Diet in Renal Disease (MDRD) Study equations for estimating GFR levels above 60 mL/min/1.73 m2. *Am J Kidney Dis* 2010;56(3):486-95.
46. Kellum JA, Lameire N, Aspelin P, et al. Kidney disease: improving global outcomes (KDIGO) acute kidney injury work group. KDIGO clinical practice guideline for acute kidney injury. *Kidney international supplements* 2012;2(1):1-138.
47. KDIGO G. Work Group. KDIGO clinical practice guideline for glomerulonephritis. *Kidney inter, Suppl* 2012;2:139-274.
48. Rivera B, Casal B, Currais L. Length of stay and mental health of the immigrant population in Spain: Evidence of the healthy immigrant effect. *Applied Economics* 2015;47(19):1972-82.
49. Collins AJ, Foley RN, Herzog C, et al. US Renal Data System 2010 Annual Data Report. *American journal of kidney diseases: the official journal of the National Kidney Foundation* 2011;57(1 Suppl 1):A8, e1.
50. Fox CS, Larson MG, Leip EP, et al. Predictors of new-onset kidney disease in a community-based population. *JAMA* 2004;291(7):844-50.
51. Banks J, Marmot M, Oldfield Z, et al. Disease and disadvantage in the United States and in England. *JAMA* 2006;295(17):2037-45.

For peer review only - http://bmjopen.bmj.com/site/about/guidelines.xhtml

52. Levin A, Stevens L, McCullough PA. Cardiovascular disease and the kidney: Tracking a killer in chronic kidney disease. *Postgrad Med* 2002;111(4):53-60.
53. Tsai Y-C, Chiu Y-W, Hung C-C, et al. Association of symptoms of depression with progression of CKD. *Am J Kidney Dis* 2012;60(1):54-61.
54. Bruce MA, Griffith DM, Thorpe RJ. Stress and the kidney. *Adv Chronic Kidney Dis* 2015;22(1):46-53.
55. Brummett BH, Barefoot JC, Siegler IC, et al. Characteristics of socially isolated patients with coronary artery disease who are at elevated risk for mortality. *Psychosom Med* 2001;63(2):267-72.
56. Eng PM, Rimm EB, Fitzmaurice G, et al. Social ties and change in social ties in relation to subsequent total and cause-specific mortality and coronary heart disease incidence in men. *Am J Epidemiol* 2002;155(8):700-09.
57. Psychosocial factors in patients with chronic kidney disease: The impact of social support on end-stage renal disease. Seminars in dialysis; 2005. Wiley Online Library.
58. Cohen SD, Sharma T, Acquaviva K, et al. Social support and chronic kidney disease: an update. *Adv Chronic Kidney Dis* 2007;14(4):335-44.
59. Uchino BN, Cacioppo JT, Kiecolt-Glaser JK. The relationship between social support and physiological processes: a review with emphasis on underlying mechanisms and implications for health. *Psychol Bull* 1996;119(3):488.
60. Tsurugano S, Nakao M, Takeuchi T, et al. Job stress strengthens the link between metabolic risk factors and renal dysfunction in adult men. *The Tohoku journal of experimental medicine* 2012;226(2):101-08.
61. Tróccol BT. Desenvolvimento de escala para avaliação do suporte social em HIV/aids. *Psicologia: Teoria e Pesquisa* 2006;22(3):317-26.
62. Schneiderman N, Ironson G, Siegel SD. Stress and health: psychological, behavioral, and biological determinants. *Annu Rev Clin Psychol* 2005;1:607-28.
63. McKercher C, Sanderson K, Jose MD. Psychosocial factors in people with chronic kidney disease prior to renal replacement therapy. *Nephrology* 2013;18(9):585-91.

For peer review only

For peer review only - http://bmjopen.bmj.com/site/about/guidelines.xhtml

Legend for figures

Figure 1: Prevalence of CKD risk among Ghanaians who have experienced negative life events and those who have not experienced any negative life events stratified by site. Definition per 2012 KDIGO (Kidney Disease: Improving Global Outcomes) guidelines.

Figure 2: Prevalence of CKD risk among Ghanaians who have experienced discrimination and those who have not experienced any discrimination stratified by site. Definitions per 2012 KDIGO (Kidney Disease: Improving Global Outcomes) guidelines.

Figure 3: Prevalence of CKD risk among Ghanaians who have experienced stress at home/work and those who have not experienced any stress at home/work in the past 12 months stratified by site. Definitions per 2012 KDIGO (Kidney Disease: Improving Global Outcomes) guidelines.

Figure 4: Prevalence of CKD risk among Ghanaians who have depressive symptoms and those who do not have depressive symptoms stratified by site. Definitions per 2012 KDIGO (Kidney Disease: Improving Global Outcomes) guidelines.

For peer review only

Legend for Supplementary Tables

S1: Association of psychosocial stressors (PS) indicators (negative life events, discrimination, stress at work/home and depression) with albuminuria, reduced eGFR and CKD risk for Ghanaians living in Ghana and those living in Europe.

S2: Association of psychosocial stressors (PS) indicators (negative life events, discrimination, stress at work/home and depression) with albuminuria, reduced eGFR and CKD risk for Ghanaians living in Ghana and those living in Europe stratified by obesity status.

S3: Association of psychosocial stressors (PS) indicators (negative life events, discrimination, stress at work/home and depression) with albuminuria, reduced eGFR and CKD risk for Ghanaians living in Ghana and those living in Europe stratified by diabetes status.

S4: Association of psychosocial stressors (PS) indicators (negative life events, discrimination, stress at work/home and depression) with albuminuria, reduced eGFR and CKD risk for Ghanaians living in Ghana and those living in Europe stratified by hypertensive status.

For peer review only

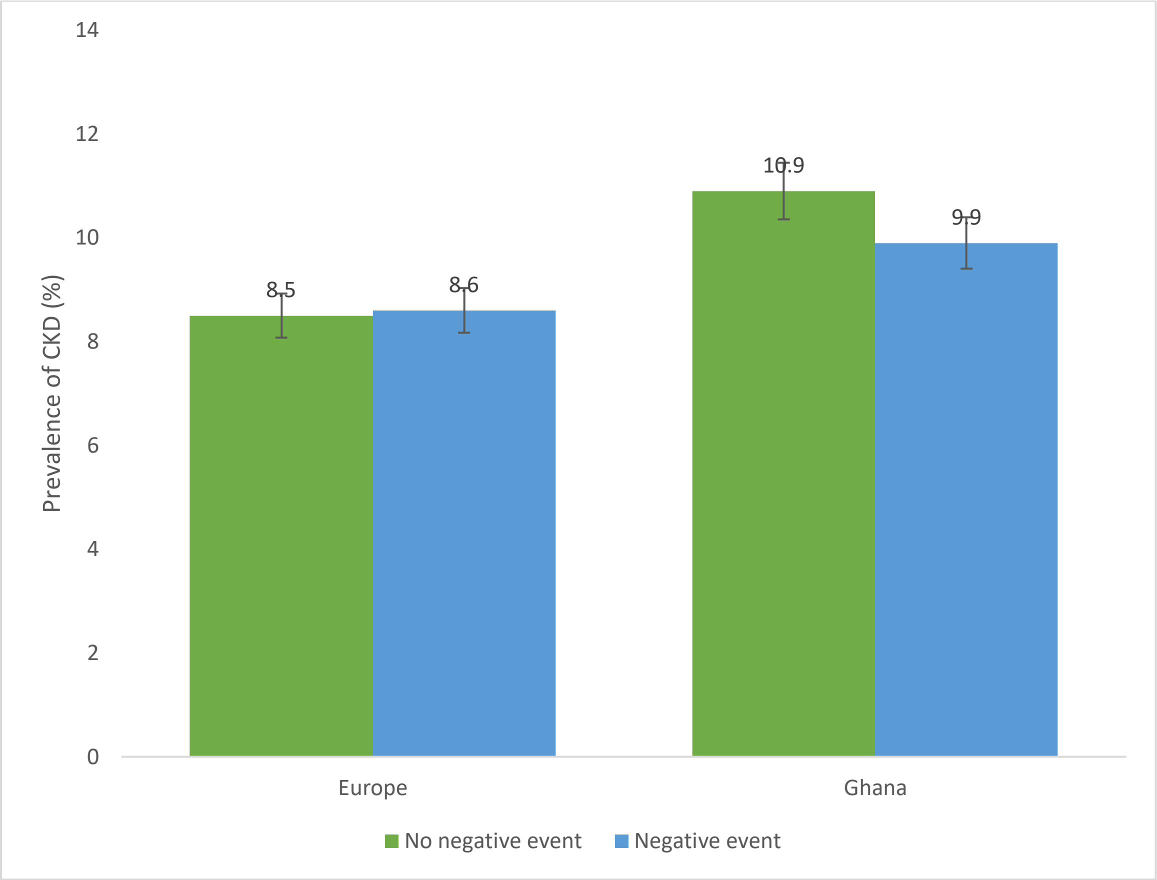

Figure 1: Prevalence of CKD risk among Ghanaians who have experienced negative life events and those who have not experienced any negative life events stratified by site. Definition per 2012 KDIGO (Kidney Disease: Improving Global Outcomes) guidelines.

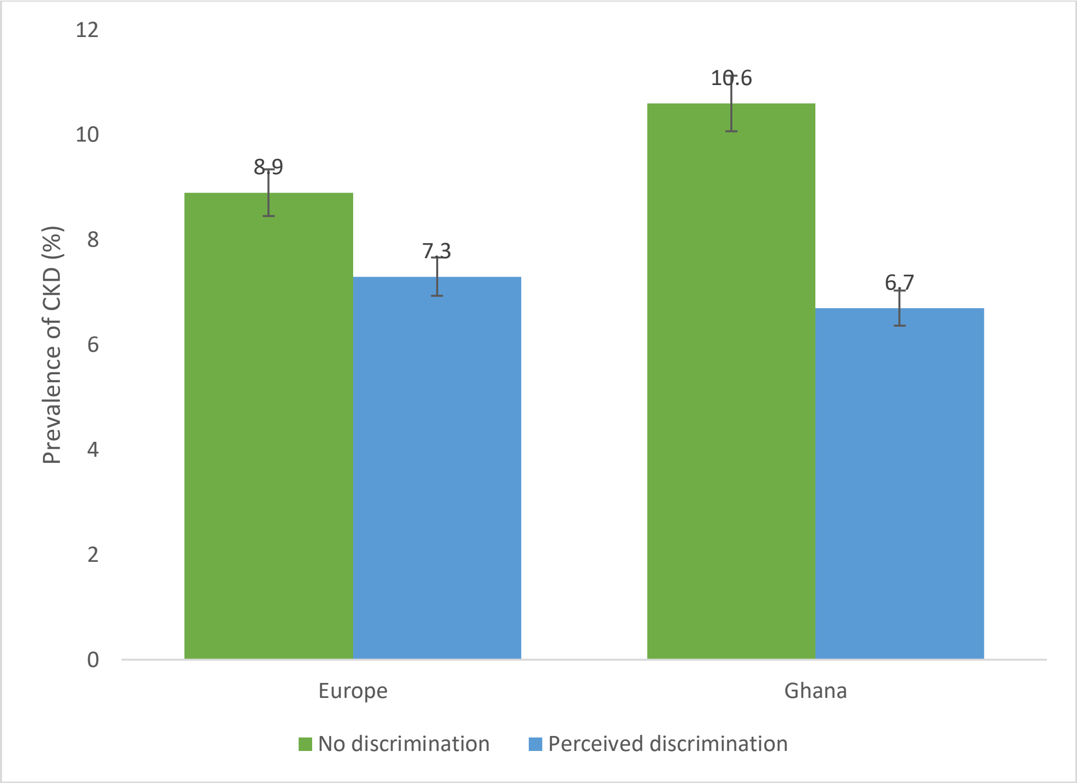

Figure 2: Prevalence of CKD risk among Ghanaians who have experienced discrimination and those who have not experienced any discrimination stratified by site. Definitions per 2012 KDIGO (Kidney Disease: Improving Global Outcomes) guidelines.

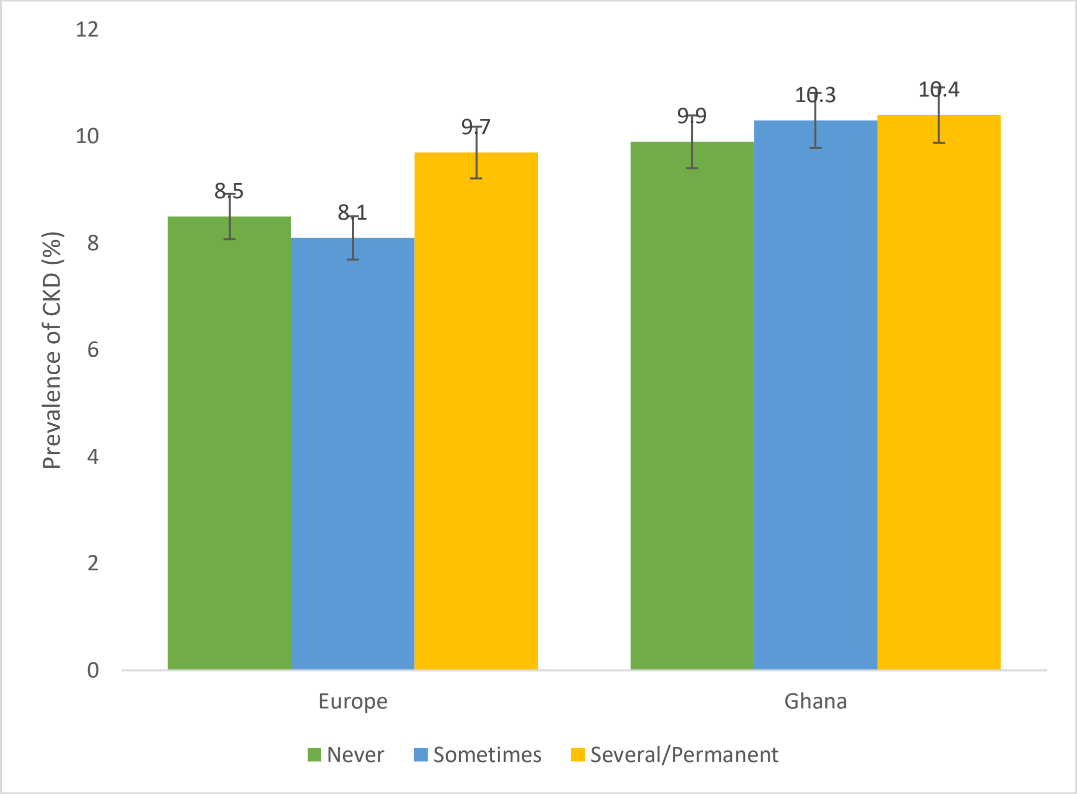

Figure 3: Prevalence of CKD risk among Ghanaians who have experienced stress at home/work and those who have not experienced any stress at home/work in the past 12 months stratified by site. Definitions per 2012 KDIGO (Kidney Disease: Improving Global Outcomes) guidelines.

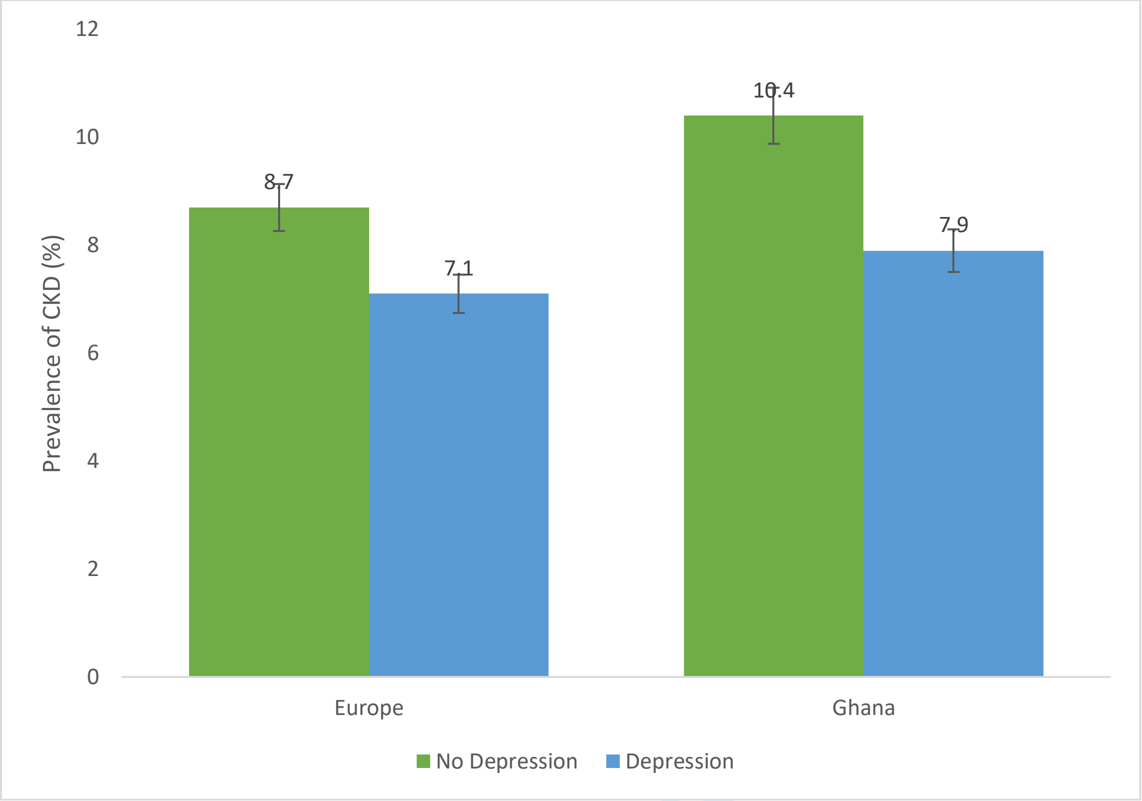

Figure 4: Prevalence of CKD risk among Ghanaians who have depressive symptoms and those who do not have depressive symptoms stratified by site. Definitions per 2012 KDIGO (Kidney Disease: Improving Global Outcomes) guidelines.

Supplementary Tables

S1: Association of psychosocial stressors (PS) indicators (negative life events, discrimination, stress at work/home and depression) with albuminuria, reduced eGFR and CKD risk for Ghanaians living in Ghana and those living in Europe

	Albuminuria (ACR ≥ 3 mg/mmol)		eGFR < 60 mL/min/1.73 m2		High to very high CKD risk (KDIGO, 2012)	
		OR (95% CI)		OR (95% CI)		OR (95% CI)
	n (%)	Model 3	n (%)	Model 3	n cases (%)	Model 3
**Negative events**						
**Europe**						
No	1128 (8.2)	1.00 (Reference)	1106 (2.6)	1.00 (Reference)	1090 (8.5)	1.00 (Reference)
Yes	1615 (8.4)	0.95 (0.70-1.30)	1587 (2.5)	0.77 (0.44-1.37)	1557 (8.6)	0.92 (0.67-1.26)
**Ghana**						
No	732 (10.4)	1.00 (Reference)	736 (4.5)	1.00 (Reference)	732 (10.9)	1.00 (Reference)
Yes	1595 (9.2)	0.79 (0.58-1.07)	1601 (3.4)	0.69 (0.43-1.09)	1590 (9.9)	0.79 (0.58-1.06)
**Discrimination**						
**Europe**						
No	1899 (8.5)	1.00 (Reference)	1867 (2.6)	1.00 (Reference)	1832 (8.9)	1.00 (Reference)
Yes	810 (7.4)	0.87 (0.61-1.21)	791 (2.2)	0.87 (0.45-1.65)	782 (7.3)	0.79 (0.55-1.12)
**Ghana**						
No	2034 (10.0)	1.00 (Reference)	2047 (3.9)	1.00 (Reference)	2031 (10.6)	1.00 (Reference)
Yes	104 (7.7)	0.92 (0.43-1.98)	104 (1.9)	0.65 (0.15-2.79)	104 (6.7)	0.74 (0.33-1.66)
**Stress at home/work**						
**Europe**						
Never	1330 (8.2)	1.00 (Reference)	1305 (3.4)	1.00 (Reference)	1282 (8.5)	1.00 (Reference)
Some stress	1002 (7.9)	1.01 (0.72-1.41)	984 (1.4)	0.51 (0.25-1.04)	968 (8.1)	1.01 (0.72-1.42)
Several/Permanent stresses	397 (9.1)	1.00 (0.64-1.59)	390 (2.3)	0.91 (0.40-2.03)	383 (9.7)	1.15 (0.74-1.79)
**Ghana**						

For peer review only - http://bmjopen.bmj.com/site/about/guidelines.xhtml

Never	682 (10.3)	1.00 (Reference)	688 (3.3)	1.00 (Reference)	682 (9.9)	1.00 (Reference)
Some stress	1279 (9.5)	0.78 (0.56-1.09)	1279 (3.9)	1.14 (0.67-1.93)	1274 (10.3)	0.93 (0.67-1.29)
Several/Permanent stresses	365 (8.5)	0.70 (0.43-1.13)	369 (4.1)	1.32 (0.65-1.68)	365 (10.4)	0.97 (0.62-1.52)
**Depressive symptoms**						
**Europe**						
No	2505 (8.5)	1.00 (Reference)	2457 (2.7)	1.00 (Reference)	2416 (8.7)	1.00 (Reference)
Yes	206 (6.3)	0.65 (0.33-1.28)	202 (1.5)	0.94 (0.28-3.14)	199 (7.1)	0.78 (0.41-1.47)
**Ghana**						
No	2212 (9.9)	1.00 (Reference)	2222 (3.8)	1.00 (Reference)	2207 (10.4)	1.00 (Reference)
Yes	114 (5.3)	0.41 (0.16-1.04)	114 (2.6)	0.58 (0.17-1.95)	114 (7.9)	0.62 (0.28-1.31)

Model 3, adjusted for age, sex educational level, hypertension, diabetes, hypercholesterolemia, BMI, physical activity and smoking; Abbreviations: CI, confidence interval; ACR, albumin creatinine ration; eGFR, estimated glomerular filtration rate; CKD, chronic kidney disease; OR, odds ratio, n= total number of Ghanaians living in Ghana and Europe among the various levels of PS constructs; %, proportion of individuals with CKD among the various levels of PS constructs in Ghana and Europe.

For peer review only

S2: Association of psychosocial stressors (PS) indicators (negative life events, discrimination, stress at work/home and depression) with albuminuria, reduced eGFR and CKD risk for Ghanaians living in Ghana and those living in Europe stratified by obesity status

	Albuminuria (ACR ≥ 3 mg/mmol)			eGFR < 60 mL/min/1.73 m2			High to very high CKD risk (KDIGO, 2012)		
		OR (95% CI)			OR (95% CI)			OR (95% CI)	
	n (%)	Model 1	Model 2	n (%)	Model 1	Model 2	n cases (%)	Model 1	Model 2
**Negative events**									
**Europe/Not obese**									
No	736 (7.4)	1.00 (Reference)	1.00 (Reference)	729 (2.5)	1.00 (Reference)	1.00 (Reference)	719 (8.5)	1.00 (Reference)	1.00 (Reference)
Yes	1003 (7.7)	0.98 (0.67-1.40)	0.99 (0.69-1.43)	992 (2.0)	0.77 (0.40-1.48)	0.71 (0.36-1.42)	971 (7.9)	0.91 (0.64-1.29)	0.89 (0.62-1.29)
**Europe/Obese**									
No	390 (9.2)	1.00 (Reference)	1.00 (Reference)	375 (2.9)	1.00 (Reference)	1.00 (Reference)	369 (8.7)	1.00 (Reference)	1.00 (Reference)
Yes	607 (9.6)	1.05 (0.67-1.63)	1.14 (0.72-1.82)	590 (3.2)	0.97 (0.44-2.11)	1.01 (0.44-2.26)	581 (9.6)	1.15 (0.73-1.82)	1.15 (0.71-1.85)
**Ghana/Not obese**									
No	588 (9.5)	1.00 (Reference)	1.00 (Reference)	592 (4.7)	1.00 (Reference)	1.00 (Reference)	588 (10.0)	1.00 (Reference)	1.00 (Reference)
Yes	1324 (8.5)	0.86 (0.61-1.21)	0.83 (0.59-1.17)	1331 (3.4)	0.64 (0.39-1.05)	0.65 (0.39-1.83)	1321 (9.0)	0.86 (0.61-1.20)	0.84 (0.60-1.18)
**Ghana/Obese**									
No	144 (13.9)	1.00 (Reference)	1.00 (Reference)	144 (3.5)	1.00 (Reference)	1.00 (Reference)	144 (14.6)	1.00 (Reference)	1.00 (Reference)
Yes	268 (13.1)	0.91 (0.50-1.64)	0.91 (0.49-1.65)	267 (3.8)	1.04 (0.34-3.16)	1.09 (0.35-3.37)	266 (14.3)	0.94 (0.52-1.68)	0.94 (0.52-1.67)
**Discrimination**									
**Europe/Not obese**									
No	1186 (8.4)	1.00 (Reference)	1.00 (Reference)	1172 (2.4)	1.00 (Reference)	1.00 (Reference)	1150 (9.4)	1.00 (Reference)	1.00 (Reference)
Yes	538 (5.9)	0.69 (0.46-1.05)	0.71 (0.46-1.11)	532 (1.5)	0.62 (0.28-1.37)	0.57 (0.24-1.34)	525 (5.9)	**0.63 (0.42-0.96)**	**0.63 (0.41-0.97)**
**Europe/Obese**									
No	709 (8.7)	1.00 (Reference)	1.00 (Reference)	691 (3.0)	1.00 (Reference)	1.00 (Reference)	678 (8.9)	1.00 (Reference)	1.00 (Reference)
Yes	269 (10.0)	1.14 (0.71-1.85)	1.29 (0.78-2.12)	256 (3.5)	1.15 (0.50-2.63)	1.27 (0.54-2.99)	254 (9.8)	1.13 (0.69-1.86)	1.22 (0.73-2.13)
**Ghana/Not obese**									
No	1648 (9.1)	1.00 (Reference)	1.00 (Reference)	1661 (3.9)	1.00 (Reference)	1.00 (Reference)	1646 (9.6)	1.00 (Reference)	1.00 (Reference)
Yes	81 (8.6)	1.06 (0.48-2.38)	1.04 (0.47-2.32)	81 (2.5)	1.04 (0.48-2.32)	0.86 (0.20-3.69)	81 (7.4)	0.90 (0.38-2.12)	0.89 (0.38-2.11)
**Ghana/Obese**									

For peer review only - http://bmjopen.bmj.com/site/about/guidelines.xhtml

No	383 (13.8)	1.00 (Reference)	1.00 (Reference)	383 (3.7)	1.00 (Reference)	1.00 (Reference)	382 (14.9)	1.00 (Reference)	1.00 (Reference)
Yes	23 (4.4)	0.31 (0.04-2.36)	0.40 (0.05-3.11)	23 (0.0)	**** (***-***)	**** (***-***)	23 (4.4)	0.29 (0.04-2.22)	0.34 (0.04-2.66)
**Stress at home/work**									
**Europe/Not obese**									
Never	855 (7.4)	1.00 (Reference)	1.00 (Reference)	847 (3.4)	1.00 (Reference)	1.00 (Reference)	831 (8.2)	1.00 (Reference)	1.00 (Reference)
Some stress	629 (8.3)	1.16 (0.79-1.70)	1.20 (0.81-1.79)	627 (1.4)	0.52 (0.24-1.12)	0.47 (0.21-1.07)	615 (7.9)	0.98 (0.66-1.43)	0.98 (0.65-1.46)
Several/Permanent stresses	247 (6.9)	0.93 (0.53-1.62)	1.15 (0.77-1.72)	239 (2.3)	0.45 (0.13-1.52)	0.49 (0.15-1.67)	236 (9.1)	0.97 (0.57-1.65)	1.04 (0.61-1.78)
**Europe/Obese**									
Never	472 (9.8)	1.00 (Reference)	1.00 (Reference)	455 (3.9)	1.00 (Reference)	1.00 (Reference)	448 (10.3)	1.00 (Reference)	1.00 (Reference)
Some stress	372 (7.5)	0.72 (0.44-1.18)	0.79 (0.47-1.32)	356 (1.4)	0.41 (0.15-1.16)	0.43 (0.15-1.21)	352 (9.5)	0.94 (0.57-1.53)	1.05 (0.62-1.76)
Several/Permanent stresses	147 (12.2)	1.32 (0.74-2.38)	1.31 (0.72-2.42)	148 (4.1)	1.05 (0.40-2.79)	1.09 (0.40-2.91)	144 (8.5)	1.36 (0.74-2.49)	1.41 (0.75-2.64)
**Ghana/Not obese**									
Never	537 (9.7)	1.00 (Reference)	1.00 (Reference)	542 (3.1)	1.00 (Reference)	1.00 (Reference)	537 (8.9)	1.00 (Reference)	1.00 (Reference)
Some stress	1055 (8.3)	0.79 (0.56-1.15)	0.74 (0.52-1.07)	1057 (3.9)	1.16 (0.65-2.09)	1.21 (0.67-2.18)	1052 (9.0)	0.92 (0.64-1.35)	0.90 (0.62-1.32)
Several/permanent stresses	319 (8.5)	0.81 (0.49-1.32)	0.73 (0.44-1.20)	323 (4.3)	1.34 (0.64-2.83)	1.43 (0.67-3.04)	319 (10.7)	1.12 (0.69-1.79)	1.09 (0.68-1.76)
**Ghana/Obese**									
Never	145 (12.4)	1.00 (Reference)	1.00 (Reference)	146 (4.1)	1.00 (Reference)	1.00 (Reference)	145 (13.8)	1.00 (Reference)	1.00 (Reference)
Some stress	221 (14.9)	1.21 (0.64-2.26)	1.05 (0.55-1.99)	219 (3.7)	0.74 (0.25-2.22)	0.83 (0.26-2.06)	219 (15.9)	1.13 (0.62-2.07)	1.02 (0.55-1.89)
Several/Permanent stresses	46 (8.7)	0.64 (0.20-2.01)	0.52 (0.16-1.65)	46 (2.2)	0.41 (0.05-3.61)	0.45 (0.05-4.01)	46 (8.8)	0.55 (0.17-1.71)	0.47 (0.14-1.50)
**Depressive symptoms**									
**Europe/Not obese**									
No	1601 (7.9)	1.00 (Reference)	1.00 (Reference)	1586 (2.2)	1.00 (Reference)	1.00 (Reference)	1558 (8.3)	1.00 (Reference)	1.00 (Reference)
Yes	121 (4.9)	0.59 (0.25-1.36)	0.65 (0.27-1.48)	117 (1.7)	0.79 (0.18-2.35)	0.86 (0.21-3.80)	115 (5.22)	0.58 (0.25-1.35)	0.64 (0.27-1.49)
**Europe/Obese**									
No	899 (9.7)	1.00 (Reference)	1.00 (Reference)	866 (3.4)	1.00 (Reference)	1.00 (Reference)	853 (9.4)	1.00 (Reference)	1.00 (Reference)
Yes	83 (7.2)	0.71 (0.29-1.67)	0.72 (0.30-1.74)	83 (1.2)	0.46 (0.06-3.52)	0.46 (0.06-3.57)	82 (8.5)	0.89 (0.40-2.01)	0.90 (0.40-2.04)

For peer review only - http://bmjopen.bmj.com/site/about/guidelines.xhtml

**Ghana/Not obese**									
No	1811 (8.9)	1.00 (Reference)	1.00 (Reference)	1822 (3.9)	1.00 (Reference)	1.00 (Reference)	1808 (9.4)	1.00 (Reference)	1.00 (Reference)
Yes	100 (6.0)	0.57 (0.24-1.33)	0.56 (0.24-1.32)	100 (2.0)	0.38 (0.09-1.63)	0.38 (0.09-1.63)	100 (8.0)	0.69 (0.32-1.46)	0.69 (0.32-1.47)
**Ghana/Obese**									
No	398 (13.8)	1.00 (Reference)	1.00 (Reference)	397 (3.5)	1.00 (Reference)	1.00 (Reference)	396 (14.7)	1.00 (Reference)	1.00 (Reference)
Yes	14 (0.0)	*** (****-****)	*** (****-****)	14 (7.1)	1.76 (0.21-14.89)	2.14 (0.25-8.78)	14 (7.1)	0.42 (0.05-3.32)	0.38 (0.05-3.07)

Model 1, adjusted for age and sex; Model 2, adjusted for age, sex, and educational level for Ghanaians (SSA) and length of stay for those in Europe; Abbreviations: CI, confidence interval; ACR, albumin creatinine ration; eGFR, estimated glomerular filtration rate; CKD, chronic kidney disease; OR, odds ratio, n= total number of Ghanaians living in Ghana and Europe among the various levels of PS constructs; %, proportion of individuals with CKD among the various levels of PS constructs in Ghana and Europe.

For peer review only

For peer review only - http://bmjopen.bmj.com/site/about/guidelines.xhtml

S3: Association of psychosocial stressors (PS) indicators (negative life events, discrimination, stress at work/home and depression) with albuminuria, reduced eGFR and CKD risk for Ghanaians living in Ghana and those living in Europe stratified by diabetes status

	Albuminuria (ACR ≥ 3 mg/mmol)			eGFR < 60 mL/min/1.73 m2			High to very high CKD risk (KDIGO, 2012)		
		OR (95% CI)			OR (95% CI)			OR (95% CI)	
	n (%)	Model 1	Model 2	n (%)	Model 1	Model 2	n cases (%)	Model 1	Model 2
**Negative events**									
**Europe/No diabetes**									
No	986 (8.0)	1.00 (Reference)	1.00 (Reference)	971 (2.1)	1.00 (Reference)	1.00 (Reference)	957 (7.9)	1.00 (Reference)	1.00 (Reference)
Yes	1359 (7.7)	0.95 (0.70-1.29)	0.97 (0.71-1.34)	1340 (2.0)	0.90 (0.50-1.63)	0.90 (0.48-1.68)	1315 (8.1)	1.01 (0.75-1.37)	0.99 (0.72-1.36)
**Europe/Diabetes**									
No	142 (9.9)	1.00 (Reference)	1.00 (Reference)	135 (6.7)	1.00 (Reference)	1.00 (Reference)	133 (12.8)	1.00 (Reference)	1.00 (Reference)
Yes	256 (12.5)	1.28 (0.66-2.50)	1.41 (0.71-2.82)	247 (4.9)	0.75 (0.29-1.89)	10.68 (0.27-1.77)	242 (11.2)	0.87 (0.46-1.69)	0.96 (0.49-1.88)
**Ghana/No diabetes**									
No	683 (9.1)	1.00 (Reference)	1.00 (Reference)	687 (4.1)	1.00 (Reference)	1.00 (Reference)	683 (9.8)	1.00 (Reference)	1.00 (Reference)
Yes	1447 (7.7)	0.84 (0.60-1.16)	0.82 (0.59-1.14)	1451 (3.1)	0.69 (0.42-1.13)	0.69 (0.42-1.13)	1442 (8.3)	0.82 (0.59-1.12)	0.80 (0.58-1.10)
**Ghana/Diabetes**									
No	49 (28.3)	1.00 (Reference)	1.00 (Reference)	49 (10.2)	1.00 (Reference)	1.00 (Reference)	49 (26.4)	1.00 (Reference)	1.00 (Reference)
Yes	148 (24.3)	0.78 (0.37-1.61)	0.72 (0.34-1.52)	150 (7.3)	0.55 (0.16-1.81)	0.57 (0.17-1.88)	148 (26.4)	0.93 (0.45-1.97)	0.90 (0.43-1.92)
**Discrimination**									
**Europe/No diabetes**									
No	1629 (7.9)	1.00 (Reference)	1.00 (Reference)	1609 (2.2)	1.00 (Reference)	1.00 (Reference)	1580 (8.3)	1.00 (Reference)	1.00 (Reference)
Yes	684 (6.7)	0.85 (0.60-1.21)	0.89 (0.62-1.28)	669 (1.5)	0.68 (0.33-1.40)	0.69 (0.32-1.47)	661 (7.0)	0.84 (0.59-1.20)	0.86 (0.59-1.25)
**Europe/Diabetes**									
No	270 (11.9)	1.00 (Reference)	1.00 (Reference)	258 (5.4)	1.00 (Reference)	1.00 (Reference)	252 (13.1)	1.00 (Reference)	1.00 (Reference)
Yes	126 (11.1)	0.87 (0.44-1.71)	0.89 (0.45-1.76)	122 (5.7)	1.29 (0.48-3.45)	1.25 (0.46-3.37)	121 (9.1)	0.68 (0.32-1.40)	0.69 (0.33-1.42)
**Ghana/No diabetes**									
No	1854 (8.4)	1.00 (Reference)	1.00 (Reference)	1865 (3.5)	1.00 (Reference)	1.00 (Reference)	1851 (9.0)	1.00 (Reference)	1.00 (Reference)
Yes	99 (8.1)	1.03 (0.49-2.17)	1.04 (0.49-2.19)	99 (2.0)	0.79 (0.19-3.33)	0.78 (0.18-3.29)	99 (7.1)	0.87 (0.39-1.93)	0.88 (0.39-1.95)

For peer review only

For peer review only - http://bmjopen.bmj.com/site/about/guidelines.xhtml

**Ghana/Diabetes**									
No	180 (26.7)	1.00 (Reference)	1.00 (Reference)	182 (7.7)	1.00 (Reference)	1.00 (Reference)	180 (27.2)	1.00 (Reference)	1.00 (Reference)
Yes	5 (0.0)	**** (***-***)	0.40 (0.05-3.11)	5 (0.0)	**** (***-***)	**** (***-***)	5 (0.0)	**** (***-***)	**** (***-***)
**Stress at home/work**									
**Europe/No diabetes**									
Never	1137 (7.9)	1.00 (Reference)	1.00 (Reference)	1127 (2.3)	1.00 (Reference)	1.00 (Reference)	1102 (8.0)	1.00 (Reference)	1.00 (Reference)
Some stress	860 (7.2)	0.90 (0.65-1.27)	0.96 (0.68-1.36)	850 (1.2)	0.49 (0.24-1.02)	0.49 (0.22-1.07)	835 (7.4)	0.94 (0.67-1.31)	0.99 (0.69-1.41)
Several/Permanent stresses	335 (8.4)	1.04 (0.67-1.62)	1.07 (0.68-1.70)	327 (2.1)	0.84 (0.36-1.95)	0.92 (0.39-2.17)	322 (9.6)	1.20 (0.77-1.85)	1.28 (0.82-1.99)
**Europe/Diabetes**									
Never	193 (9.8)	1.00 (Reference)	1.00 (Reference)	184 (8.2)	1.00 (Reference)	1.00 (Reference)	180 (11.7)	1.00 (Reference)	1.00 (Reference)
Some stress	142 (12.7)	1.22 (0.61-2.45)	1.38 (0.67-2.81)	134 (3.0)	0.43 (0.13-1.39)	0.40 (0.12-1.29)	133 (12.0)	1.04 (0.52-2.09)	1.13 (0.56-2.32)
Several/Permanent stresses	62 (12.9)	1.31 (0.54-3.16)	1.38 (0.56-3.41)	63 (3.2)	0.49 (0.10-2.29)	0.46 (0.10-2.23)	61 (9.8)	0.82 (0.31-2.13)	0.89 (0.34-2.35)
**Ghana/No diabetes**									
Never	628 (7.8)	1.00 (Reference)	1.00 (Reference)	634 (2.7)	1.00 (Reference)	1.00 (Reference)	628 (7.6)	1.00 (Reference)	1.00 (Reference)
Some stress	1163 (8.6)	1.06 (0.74-1.51)	0.97 (0.67-1.39)	1163 (3.7)	1.27 (0.71-2.27)	1.32 (0.74-2.34)	1158 (9.3)	1.15 (0.80-1.65)	1.10 (0.77-1.58)
Several/permanent stresses	339 (7.4)	0.88 (0.53-1.46)	0.76 (0.46-1.27)	341 (3.5)	1.24 (0.58-2.67)	1.35 (0.62-2.95)	339 (8.9)	1.07 (0.66-1.73)	1.00 (0.61-1.64)
**Ghana/Diabetes**									
Never	54 (38.9)	1.00 (Reference)	1.00 (Reference)	54 (11.1)	1.00 (Reference)	1.00 (Reference)	54 (37.0)	1.00 (Reference)	1.00 (Reference)
Some stress	116 (18.9)	**0.37 (0.37-0.77)**	**0.35 (0.17-0.75)**	116 (6.0)	0.47 (0.14-1.60)	0.45 (0.12-1.55)	116 (19.8)	**0.43 (0.21-0.89)**	**0.40 (0.19-0.86)**
Several/Permanent stresses	26 (25.0)	0.44 (0.44-1.29)	0.39 (0.13-1.19)	28 (10.7)	0.65 (0.13-3.21)	0.62 (0.12-3.23)	26 (30.8)	0.69 (0.25-1.93)	0.64 (0.22-1.87)
**Depressive symptoms**									
**Europe/No diabetes**									
No	2154 (8.0)	1.00 (Reference)	1.00 (Reference)	2122 (2.1)	1.00 (Reference)	1.00 (Reference)	2087 (8.1)	1.00 (Reference)	1.00 (Reference)
Yes	167 (5.4)	0.63 (0.32-1.25)	0.69 (0.34-1.38)	163 (2.0)	0.65 (0.15-2.72)	0.72 (0.17-3.04)	161 (6.8)	0.58 (0.25-1.35)	0.86 (0.46-1.64)
**Europe/Diabetes**									

For peer review only - http://bmjopen.bmj.com/site/about/guidelines.xhtml

No	351 (12.0)	1.00 (Reference)	1.00 (Reference)	335 (35.9)	1.00 (Reference)	1.00 (Reference)	329 (12.5)	1.00 (Reference)	1.00 (Reference)
Yes	39 (10.3)	0.79 (0.26-1.35)	0.73 (0.24-2.19)	39 (2.6)	0.49 (0.06-3.97)	0.49 (0.06-3.99)	38 (7.9)	0.89 (0.40-2.01)	0.60 (0.17-2.08)
**Ghana/No diabetes**									
No	2027 (8.3)	1.00 (Reference)	1.00 (Reference)	2035 (3.4)	1.00 (Reference)	1.00 (Reference)	2022 (8.8)	1.00 (Reference)	1.00 (Reference)
Yes	102 (4.9)	0.50 (0.20-1.25)	0.48 (0.19-1.19)	102 (2.9)	0.64 (0.19-2.13)	0.67 (0.20-2.21)	102 (7.8)	0.69 (0.32-1.46)	0.71 (0.34-1.51)
**Ghana/Diabetes**									
No	185 (26.5)	1.00 (Reference)	1.00 (Reference)	187 (8.7)	1.00 (Reference)	1.00 (Reference)	185 27.6)	1.00 (Reference)	1.00 (Reference)
Yes	12 (8.3)	0.27 (0.03-2.13)	0.25 (0.03-2.01)	12 (0.0)	*** (****-****)	*** (***-***)	12 (8.3)	0.42 (0.05-3.32)	0.27 (0.03-2.19)

Model 1, adjusted for age and sex; Model 2, adjusted for age, sex, and educational level for Ghanaians (SSA) and length of stay for those in Europe; Abbreviations: CI, confidence interval; ACR, albumin creatinine ration; eGFR, estimated glomerular filtration rate; CKD, chronic kidney disease; OR, odds ratio, n= total number of Ghanaians living in Ghana and Europe among the various levels of PS constructs; %, proportion of individuals with CKD among the various levels of PS constructs in Ghana and Europe.

For peer review only

For peer review only - http://bmjopen.bmj.com/site/about/guidelines.xhtml

S4: Association of psychosocial stressors (PS) indicators (negative life events, discrimination, stress at work/home and depression) with albuminuria, reduced eGFR and CKD risk for Ghanaians living in Ghana and those living in Europe stratified by hypertensive status

	Albuminuria (ACR ≥ 3 mg/mmol)			eGFR < 60 mL/min/1.73 m2			High to very high CKD risk (KDIGO, 2012)		
		OR (95% CI)			OR (95% CI)			OR (95% CI)	
	n (%)	Model 1	Model 2	n (%)	Model 1	Model 2	n cases (%)	Model 1	Model 2
**Negative events**									
**Europe/No hypertension**									
No	568 (6.9)	1.00 (Reference)	1.00 (Reference)	562 (1.3)	1.00 (Reference)	1.00 (Reference)	551 (7.1)	1.00 (Reference)	1.00 (Reference)
Yes	743 (5.0)	0.72 (0.45-1.15)	0.78 (0.48-1.27)	740 (0.8)	0.59 (0.19-1.77)	0.60 (0.20-1.83)	724 (5.1)	0.71 (0.44-1.13)	0.75 (0.46-1.22)
**Europe/Hypertension**									
No	560 (9.6)	1.00 (Reference)	1.00 (Reference)	544 (4.0)	1.00 (Reference)	1.00 (Reference)	539 (10.0)	1.00 (Reference)	1.00 (Reference)
Yes	872 (11.4)	1.22 (0.86-1.73)	1.20 (0.83-1.73)	847 (3.9)	0.95 (0.54-1.67)	0.89 (0.49-1.62)	833 (11.6)	1.19 (0.83-1.69)	1.13 (0.78-1.62)
**Ghana/No Hypertension**									
No	540 (6.3)	1.00 (Reference)	1.00 (Reference)	543 (2.2)	1.00 (Reference)	1.00 (Reference)	540 (6.9)	1.00 (Reference)	1.00 (Reference)
Yes	1118 (5.6)	0.88 (0.57-1.36)	0.84 (0.54-1.31)	1124 (1.9)	0.82 (0.39-1.69)	0.86 (0.41-1.79)	1115 (6.4)	0.93 (0.61-1.40)	0.90 (0.59-1.36)
**Ghana/Hyperten sion**									
No	192 (21.9)	1.00 (Reference)	1.00 (Reference)	193 (10.9)	1.00 (Reference)	1.00 (Reference)	192 (22.4)	1.00 (Reference)	1.00 (Reference)
Yes	476 (18.1)	0.78 (0.52-1.18)	0.79 (0.52-1.20)	476 (6.9)	0.58 (0.32-1.04)	0.58 (0.33-1.05)	474 (18.4)	0.77 (0.50-1.16)	0.76 (0.51-1.16)
**Discrimination**									
**Europe/No hypertension**									
No	916 (6.3)	1.00 (Reference)	1.00 (Reference)	907 (0.8)	1.00 (Reference)	1.00 (Reference)	885 (6.8)	1.00 (Reference)	1.00 (Reference)
Yes	379 (3.7)	0.60 (0.33-1.09)	0.65 (0.36-1.21)	378 (1.1)	1.35 (0.39-4.74)	1.37 (0.38-4.89)	374 (3.2)	**0.47 (0.25-0.89)**	**0.51 (0.27-0.97)**
**Europe/Hypertension**									
No	983 (10.5)	1.00 (Reference)	1.00 (Reference)	960 (4.4)	1.00 (Reference)	1.00 (Reference)	947 (10.9)	1.00 (Reference)	1.00 (Reference)
Yes	431 (10.7)	1.03 (0.71-1.49)	1.05 (0.72-1.54)	413 (3.2)	0.73 (0.38-1.39)	0.75 (0.38-1.48)	408 (11.0)	1.03 (0.71-1.49)	1.04 (0.71-1.53)
**Ghana/No hypertension**									
No	1427 (5.7)	1.00 (Reference)	1.00 (Reference)	1437 (2.2)	1.00 (Reference)	1.00 (Reference)	1424 (6.5)	1.00 (Reference)	1.00 (Reference)

For peer review only - http://bmjopen.bmj.com/site/about/guidelines.xhtml
For peer review only

Yes	81 (7.4)	1.30 (0.55-3.09)	1.30 (0.55-3.10)	81 (0.0)	**** (***-***)	**** (***-***)	81 (7.4)	1.21 (0.51-2.88)	1.22 (0.51-2.89)
**Ghana/Hypertension**									
No	606 (20.3)	1.00 (Reference)	1.00 (Reference)	609 (7.9)	1.00 (Reference)	1.00 (Reference)	606 (20.3)	1.00 (Reference)	1.00 (Reference)
Yes	23 (8.7)	0.38 (0.09-1.68)	0.40 (0.09-1.74)	23 (8.7)	1.27 (0.28-5.70)	1.20 (0.26-5.44)	23 (4.4)	0.19 (0.03-1.44)	0.19 (0.03-1.45)
**Stress at home/work**									
**Europe/No hypertension**									
Never	644 (6.1)	1.00 (Reference)	1.00 (Reference)	640 (1.3)	1.00 (Reference)	1.00 (Reference)	625 (5.8)	1.00 (Reference)	1.00 (Reference)
Some stress	468 (4.5)	0.74 (0.43-1.27)	0.81 (0.46-1.43)	464 (0.4)	0.37 (0.07-1.74)	0.41 (0.09-2.01)	457 (4.8)	0.84 (0.49-1.45)	0.91 (0.52-1.60)
Several/Permanent stresses	192 (6.8)	1.07 (0.56-2.05)	1.19 (0.61-2.29)	191 (1.6)	1.49 (0.38-5.78)	1.52 (0.39-5.98)	186 (8.1)	1.38 (0.74-2.60)	1.49 (0.79-2.84)
**Europe/Hypertension**									
Never	686 (10.2)	1.00 (Reference)	1.00 (Reference)	665 (5.4)	1.00 (Reference)	1.00 (Reference)	657 (11.1)	1.00 (Reference)	1.00 (Reference)
Some stress	534 (11.1)	1.03 (0.71-1.49)	1.05 (0.72-1.54)	520 (2.3)	**0.49 (0.25-0.96)**	**0.47 (0.23-0.95)**	511 (10.9)	0.97 (0.67-1.41)	0.99 (0.67-0.47)
Several/Permanent stresses	205 (11.2)	1.11 (0.67-1.82)	1.12 (0.67-1.87)	199 (3.0)	0.56 (0.23-1.37)	0.58 (0.24-1.43)	197 (11.2)	0.99 (0.59-1.64)	1.03 (0.61-1.74)
**Ghana/No hypertension**									
Never	495 (5.5)	1.00 (Reference)	1.00 (Reference)	498 (1.4)	1.00 (Reference)	1.00 (Reference)	495 (5.9)	1.00 (Reference)	1.00 (Reference)
Some stress	899 (5.9)	1.07 (0.66-1.73)	0.99 (0.61-1.60)	901 (2.2)	1.30 (0.54-3.15)	1.39 (0.57-3.37)	896 (6.5)	1.04 (0.65-1.65)	1.00 (0.63-1.59)
Several/permanent stresses	263 (5.7)	1.01 (0.53-1.95)	0.89 (0.46-1.72)	267 (2.6)	1.69 (0.58-5.01)	1.89 (0.63-5.65)	263 (7.6)	1.21 (0.67-2.20)	1.15 (0.63-2.11)
**Ghana/Hypertension**									
Never	187 (22.9)	1.00 (Reference)	1.00 (Reference)	190 (8.4)	1.00 (Reference)	1.00 (Reference)	187 (20.9)	1.00 (Reference)	1.00 (Reference)
Some stress	379 (18.2)	0.74 (0.48-1.14)	0.68 (0.43-1.05)	377 (7.9)	0.95 (0.50-1.80)	0.99 (0.51-1.91)	377 (19.4)	0.90 (0.58-1.40)	0.87 (0.55-1.36)
Several/Permanent stresses	102 (15.7)	0.62 (0.33-1.17)	0.54 (0.28-1.04)	102 (7.8)	0.92 (0.37-2.26)	0.97 (0.39-2.43)	102 (17.7)	0.79 (0.42-1.49)	0.76 (0.40-1.43)
**Depressive symptoms**									
**Europe/No hypertension**									

For peer review only

No	1195 (5.9)	1.00 (Reference)	1.00 (Reference)	1187 (1.1)	1.00 (Reference)	1.00 (Reference)	1162 (6.0)	1.00 (Reference)	1.00 (Reference)
Yes	101 (3.9)	0.59 (0.21-1.65)	0.64 (0.23-1.82)	99 (1.0)	1.18 (0.15-2.72)	1.27 (0.16-10.17)	98 (5.1)	0.77 (0.30-1.97)	0.84 (0.33-2.17)
**Europe/Hypertension**									
No	1310(10.9)	1.00 (Reference)	1.00 (Reference)	1270 (4.1)	1.00 (Reference)	1.00 (Reference)	1254 (11.2)	1.00 (Reference)	1.00 (Reference)
Yes	105 (8.6)	0.76 (0.37-1.53)	0.75 (0.37-1.55)	103 (1.9)	0.52 (0.12-2.18)	0.56 (0.13-2.38)	101 (8.9)	0.77 (0.38-1.58)	0.80 (0.39-1.63)
**Ghana/No hypertension**									
No	1575 (5.9)	1.00 (Reference)	1.00 (Reference)	1584 (2.1)	1.00 (Reference)	1.00 (Reference)	1572 (6.7)	1.00 (Reference)	1.00 (Reference)
Yes	82 (2.4)	0.36 (0.09-1.51)	0.35 (0.08-1.44)	82 (1.2)	0.39 (0059-2.95)	0.39 (0.05-2.97)	82 (3.7)	0.44 (0.13-1.42)	0.43 (0.13-1.40)
**Ghana/Hypertension**									
No	636 (19.5)	1.00 (Reference)	1.00 (Reference)	637 (8.2)	1.00 (Reference)	1.00 (Reference)	634 (19.6)	1.00 (Reference)	1.00 (Reference)
Yes	32 (12.5)	0.59 (0.20-1.72)	0.59 (0.20-1.74)	32 (6.3)	0.74 (0.17-3.25)	0.76 (0.17-3.33)	32 (18.8)	0.94 (0.0.37-2.37)	0.94 (0.38-2.37)

Model 1, adjusted for age and sex; Model 2, adjusted for age, sex, and educational level for Ghanaians (SSA) and length of stay for those in Europe; Abbreviations: CI, confidence interval; ACR, albumin creatinine ration; eGFR, estimated glomerular filtration rate; CKD, chronic kidney disease; OR, odds ratio, n= total number of Ghanaians living in Ghana and Europe among the various levels of PS constructs; %, proportion of individuals with CKD among the various levels of PS constructs in Ghana and Europe.

For peer review only

STROBE Statement—checklist of items that should be included in reports of observational studies

	Item No.	Recommendation	Page No.	Relevant text from manuscript
**Title and abstract**	1	(*a*) Indicate the study's design with a commonly used term in the title or the abstract	1 lines 1-3	We have included a commonly used term in the title and abstract
		(*b*) Provide in the abstract an informative and balanced summary of what was done and what was found	3 lines 87-116	Our study shows no convincing evidence for associations between stress as indicated by four PS constructs and prevalence of CKD. Further studies aimed at identifying potential factors driving the high prevalence of CKD among these populations are needed.
**Introduction**				
Background/rationale	2	Explain the scientific background and rationale for the investigation being reported	5 lines 180-226	The theoretical and scientific background as well as the rationale for conducting the study have been provided in the introduction section.
Objectives	3	State specific objectives, including any pre specified hypotheses	5 lines 222-226	We examined the association between PS and CKD prevalence among rural and urban Ghanaians and their migrants living in three European cities. We also

For peer review only - http://bmjopen.bmj.com/site/about/guidelines.xhtml

For peer review only

				assessed if the influence of PS on CKD is partially mediated by primary risk factors (hypertension and diabetes) of CKD.
**Methods**				
Study design	4	Present key elements of study design early in the paper	-6-7 lines 232-264	Details given in the methods
Setting	5	Describe the setting, locations, and relevant dates, including periods of recruitment, exposure, follow-up, and data collection	6-7 lines 232-264	Rural or urban Ghana and three (Amsterdam, Berlin, London) European cities (migrants)
Participants	6	(*a*) *Cohort study*—Give the eligibility criteria, and the sources and methods of selection of participants. Describe methods of follow-up<br>*Case-control study*—Give the eligibility criteria, and the sources and methods of case ascertainment and control selection. Give the rationale for the choice of cases and controls<br>*Cross-sectional study*—Give the eligibility criteria, and the sources and methods of selection of participants	Pg. 7 lines 254-264	A multi-centred cross sectional baseline data from the Research on Obesity and Diabetes among African Migrants (RODAM) study. A random sample of 5,659 adults (Europe, 3167, rural, 1,043, Urban Ghana, 1,449, Ghana) aged 25 to 70 years.
		(*b*) *Cohort study*—For matched studies, give matching criteria and number of exposed and unexposed<br>*Case-control study*—For matched studies, give matching criteria and the number of controls per case		
Variables	7	Clearly define all outcomes, exposures, predictors, potential confounders, and effect modifiers. Give diagnostic criteria, if applicable	Pg. 7-10 lines 266-357	The main outcomes have been clearly defined.
Data sources/ measurement	8*	For each variable of interest, give sources of data and details of methods of assessment (measurement). Describe comparability of assessment methods if there is more than one group	Pg. 7-10 lines 266-357	We defined each variable of interest in the methods

For peer review only - http://bmjopen.bmj.com/site/about/guidelines.xhtml
For peer review only

				accordingly
Bias	9	Describe any efforts to address potential sources of bias	Pg. 9 lines 315-331	Potential sources of bias have been reported in the methods sections.
Study size	10	Explain how the study size was arrived at	Pg. 7 lines 259-264	Given in the methods section and we have also referred to the RODAM study methods paper

Continued on next page

For peer review only

Quantitative variables	11	Explain how quantitative variables were handled in the analyses. If applicable, describe which groupings were chosen and why	Pg. 10-11 lines 368-388	Please see methods
Statistical methods	12	(*a*) Describe all statistical methods, including those used to control for confounding	Pg. 10-11 lines 367-387	Please see methods
		(*b*) Describe any methods used to examine subgroups and interactions	Pg. 10-11 lines 367-387	Please see methods
		(*c*) Explain how missing data were addressed	Pg. 7 lines 259-264	
		(*d*) *Cohort study*—If applicable, explain how loss to follow-up was addressed<br>*Case-control study*—If applicable, explain how matching of cases and controls was addressed<br>*Cross-sectional study*—If applicable, describe analytical methods taking account of sampling strategy	NA	We have reported non-response across sites
		(*e*) Describe any sensitivity analyses	NA	
**Results**				
Participants	13*	(a) Report numbers of individuals at each stage of study—eg numbers potentially eligible, examined for eligibility, confirmed eligible, included in the study, completing follow-up, and analysed	Pg. 7 lines 254-264	Non-response analysis was done to shed light on the differential response rates across sites
		(b) Give reasons for non-participation at each stage	Pg. 7 lines 259-264	
		(c) Consider use of a flow diagram	Pg. 6 lines 232-234	We have also referred to RODAM methods paper
Descriptive data	14*	(a) Give characteristics of study participants (eg demographic, clinical, social) and information on exposures and potential confounders	Pg. 7 lines 269-357	We have also referred to RODAM methods paper
		(b) Indicate number of participants with missing data for each variable of interest	Pg. 7 lines 260-264	We have also referred to RODAM methods paper
		(c) *Cohort study*—Summarise follow-up time (eg, average and total amount)	NA	
Outcome data	15*	*Cohort study*—Report numbers of outcome events or summary measures over time	*NA*	
		Case-control study—Report numbers in each exposure category, or summary measures of exposure	*NA*	

For peer review only - http://bmjopen.bmj.com/site/about/guidelines.xhtml

		Cross-sectional study—Report numbers of outcome events or summary measures	Pg. 11-13	Summary measures are given in the results section and in tables and figures
Main results	16	(*a*) Give unadjusted estimates and, if applicable, confounder-adjusted estimates and their precision (eg, 95% confidence interval). Make clear which confounders were adjusted for and why they were included	*Pg. 11-23 lines 406-521*	Unadjusted and adjusted estimates are given in the results section and in figures
		(*b*) Report category boundaries when continuous variables were categorized	*Pg. 11-23 lines 389-521*	We have provided mean and corresponding standard deviations for the continuous variables.
		(*c*) If relevant, consider translating estimates of relative risk into absolute risk for a meaningful time	NA	

Continued on next page

Other analyses	17	Report other analyses done—eg analyses of subgroups and interactions, and sensitivity analyses	NA	
**Discussion**				
Key results	18	Summarise key results with reference to study objectives	Pg. 24 lines 524--531	
Limitations	19	Discuss limitations of the study, taking into account sources of potential bias or imprecision. Discuss both direction and magnitude of any potential bias	Pg. 25 lines 584-596	Key limitations regarding study methods including differential response rates and sampling methods in the various study sites have been provided
Interpretation	20	Give a cautious overall interpretation of results considering objectives, limitations, multiplicity of analyses, results from similar studies, and other relevant evidence	Pg. 24-26 lines 522-603	Cautious overall interpretation of the key findings have been provided.
Generalisability	21	Discuss the generalisability (external validity) of the study results	Pg. 25 lines 584-596	
**Other information**				
Funding	22	Give the source of funding and the role of the funders for the present study and, if applicable, for the original study on which the present article is based	Pg. 26 lines 624-630	The funders had no role in study design, data collection and analysis, decision to publish, or preparation of the manuscript

*Give information separately for cases and controls in case-control studies and, if applicable, for exposed and unexposed groups in cohort and cross-sectional studies.

For peer review only - http://bmjopen.bmj.com/site/about/guidelines.xhtml

Note: An Explanation and Elaboration article discusses each checklist item and gives methodological background and published examples of transparent reporting. The STROBE checklist is best used in conjunction with this article (freely available on the Web sites of PLoS Medicine at http://www.plosmedicine.org/, Annals of Internal Medicine at http://www.annals.org/, and Epidemiology at http://www.epidem.com/). Information on the STROBE Initiative is available at www.strobe-statement.org.

For peer review only

For peer review only - http://bmjopen.bmj.com/site/about/guidelines.xhtml
